# Characterizing the Role of *TaWRKY13* in Salt Tolerance

**DOI:** 10.3390/ijms20225712

**Published:** 2019-11-14

**Authors:** Shuo Zhou, Wei-Jun Zheng, Bao-Hua Liu, Jia-Cheng Zheng, Fu-Shuang Dong, Zhi-Fang Liu, Zhi-Yu Wen, Fan Yang, Hai-Bo Wang, Zhao-Shi Xu, He Zhao, Yong-Wei Liu

**Affiliations:** 1 Institute of Genetics and Physiology, Hebei Academy of Agriculture and Forestry Sciences/Plant Genetic Engineering Center of Hebei Province, Shijiazhuang 050051, China; zhoushuobio@163.com (S.Z.); dongfushuang@126.com (F.-S.D.); wzy1800@126.com (Z.-Y.W.); nkywanghb@163.com (H.-B.W.); 2 College of Agronomy, Northwest A&F University, Yangling 712100, China; zhengweijun@nwafu.edu.cn; 3 Handan Academy of Agricultural Sciences, Handan 056001, China; hm4589@163.com; 4 College of Agronomy, Anhui Science and Technology University, Fengyang, Chuzhou 239000, China; zhengjiachengx2016@126.com; 5 Hebei Seed Station, Shijiazhuang 050031, China; 6 Institute of Crop Sciences, Chinese Academy of Agricultural Sciences (CAAS)/National Key Facility for Crop Gene Resources and Genetic Improvement, Key Laboratory of Biology and Genetic Improvement of Triticeae Crops, Ministry of Agriculture, Beijing 100081, China; xuzhaoshi@caas.cn

**Keywords:** stress responsive mechanisms, TaWRKY transcription factors, salt tolerance

## Abstract

The WRKY transcription factor superfamily is known to participate in plant growth and stress response. However, the role of this family in wheat (*Triticum aestivum* L.) is largely unknown. Here, a salt-induced gene *TaWRKY13* was identified in an RNA-Seq data set from salt-treated wheat. The results of RT-qPCR analysis showed that *TaWRKY13* was significantly induced in NaCl-treated wheat and reached an expression level of about 22-fold of the untreated wheat. Then, a further functional identification was performed in both *Arabidopsis* *thaliana* and *Oryza sativa* L. Subcellular localization analysis indicated that TaWRKY13 is a nuclear-localized protein. Moreover, various stress-related regulatory elements were predicted in the promoter. Expression pattern analysis revealed that *TaWRKY13* can also be induced by polyethylene glycol (PEG), exogenous abscisic acid (ABA), and cold stress. After NaCl treatment, overexpressed *Arabidopsis* lines of *TaWRKY13* have a longer root and a larger root surface area than the control (Columbia-0). Furthermore, *TaWRKY13* overexpression rice lines exhibited salt tolerance compared with the control, as evidenced by increased proline (Pro) and decreased malondialdehyde (MDA) contents under salt treatment. The roots of overexpression lines were also more developed. These results demonstrate that *TaWRKY13* plays a positive role in salt stress.

## 1. Introduction

Unlike animals, plants cannot move when exposed to stress. However, complex signaling network have been established to cope with stress [1]. Under stress, a series of responses are induced to prevent or minimize damage. These are accompanied by many physiological, biochemical and developmental changes [2]. Current research on plant stress response has reached the level of cells and molecules, and combined with genetics, we can explore the stress responsive mechanisms in order to improve plant growth under conditions of stress [3,4,5,6,7].

Many genes are induced by stress; the products of these genes both participate in stress response and regulate the expression of related genes involved in signal transduction pathways in order to avoid or reduce tissue damage [8,9,10]. Signaling via the hormone, liposome, SnRK2 (sucrose non-fermenting 1-related protein kinase 2) [11], MAPK (mitogen activated protein kinase) [12], ROS signal [13] and stomatal [14] pathways are the main networks by which plants respond to salt and drought stress. Plant adaptation to drought and other stresses depends on both the expression of stress-resistant related genes and the regulation of various signal pathways induced by stress [15]. Products of stress-related genes can be divided into two classes: the first class includes ion channel proteins [16], water channel proteins [17], osmotic regulators (sucrose, proline and betaine), synthases [18] and other products that directly function in stress response, while the products of the second type include proteins involved in stress-related signal transmission and regulators of gene expression, such as protein kinases (PKs) and transcription factors (TFs) [19,20].

Transcription factors play a crucial role in regulating the expression of stress-related genes in plants. When abiotic stress occurs, changes in the activity of transcription factors cause changes in the activity of target genes. Transcription factors involved in plant stress response are widely researched, such as the AP2/EREBP TF family [21], MYC/MYB TF family [22], HSE binding TFs [23], NAC TF family [24], and WRKY TF family [25]. Among them, WRKY TFs are extensively found in higher plants including *Arabidopsis thaliana*, *Oryza sativa*, *Setaria italica*, *Glycine max*, and *Triticum aestivum*, which indicates that WRKY TFs play a significant role in plant stress tolerance [26,27,28,29,30].

Although a large number of studies have shown that WRKY TFs in plants are mainly involved in disease resistance and defense response, some members of the WRKY TFs are involved in abiotic stress response. *TaWRKY1* mediates stomatal movement through an ABA-dependent pathway to improve plant tolerance to drought stress [31]. In addition, *TaWRKY10* acts as a positive regulator under drought, salt, cold, and hydrogen peroxide stress conditions and improves the stress tolerance in transgenic tobacco [32]. In *Arabidopsis*, WRKY proteins are involved in regulating ABA response factors, such as MYB2, DREB1a, DREB2a and Rab18 [33]. The overexpression of *ZmWRKY33* in *Arabidopsis* improved the salt-stress tolerance of transgenic plants [34]. These studies suggested that WRKY TFs play a significant role in plant stress response.

High salt stress is a major obstacle to plant growth and development. High salt conditions lead to increases in reactive oxygen species (ROS), metabolic toxicity, membrane disorganization, the inhibition of photosynthesis, and attenuated nutrient acquisition at different plant growth stages [35]. Recent reports claim that salinity affects about 20% of all irrigated arable land and is an increasing problem in worldwide agriculture (FAO Cereal Supply and Demand Brief. http://www.fao.org/worldfoodsituation/csdb/en/). 

Since wheat is rich in thiamine, fat, calcium, niacin, starch, protein, iron, riboflavin, minerals, and vitamin A and can provide abundant energy and protein for humans, wheat is regarded as one of the most important crops in the world [36]. However, wheat production is constrained by environmental conditions, such as drought, salinity, waterlogging, and extremes in temperature. Next in importance to drought stress, salinity affects crop yields worldwide. The improvement of stress tolerance in wheat by biotechnology and transgenic technology could contribute to increased production worldwide. However, the huge wheat genome has slowed progress [37,38]. Although many studies have investigated the roles of WRKY transcription factors in response to various stress conditions, the mechanisms underlying their function need further study. Here, RNA-Seq, real-time fluorescence quantification PCR (RT-qPCR), and several databases were used in a study of *TaWRKY13*. The results demonstrated that overexpression of *TaWRKY13* can improve salt tolerance in *Arabidopsis* and rice. 

## 2. Results

### 2.1. Identification and Genome Structure Analysis of WRKYs in Triticum aestivum

**Figure 1:**
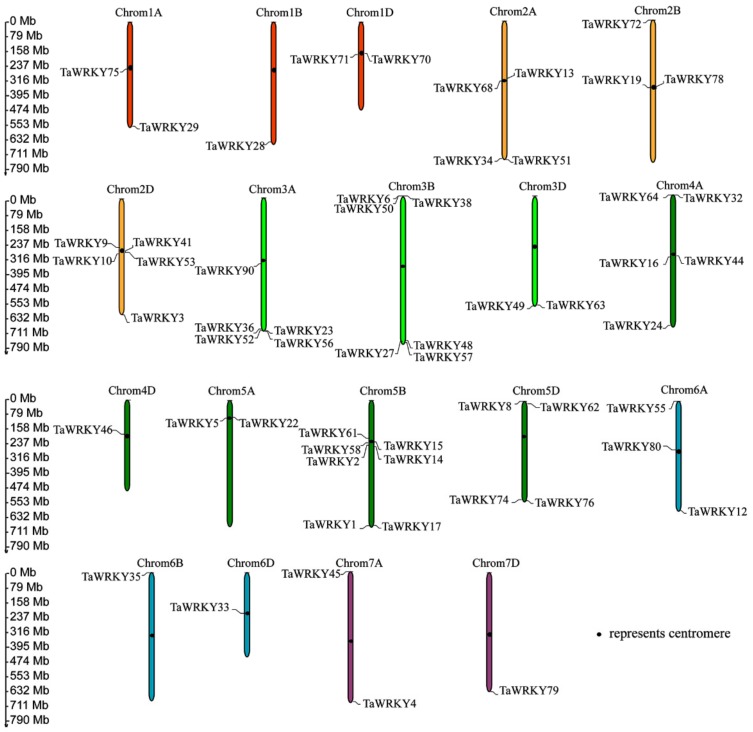
Chromosome location of TaWRKYs listed in Table 1.

**Figure 2:**
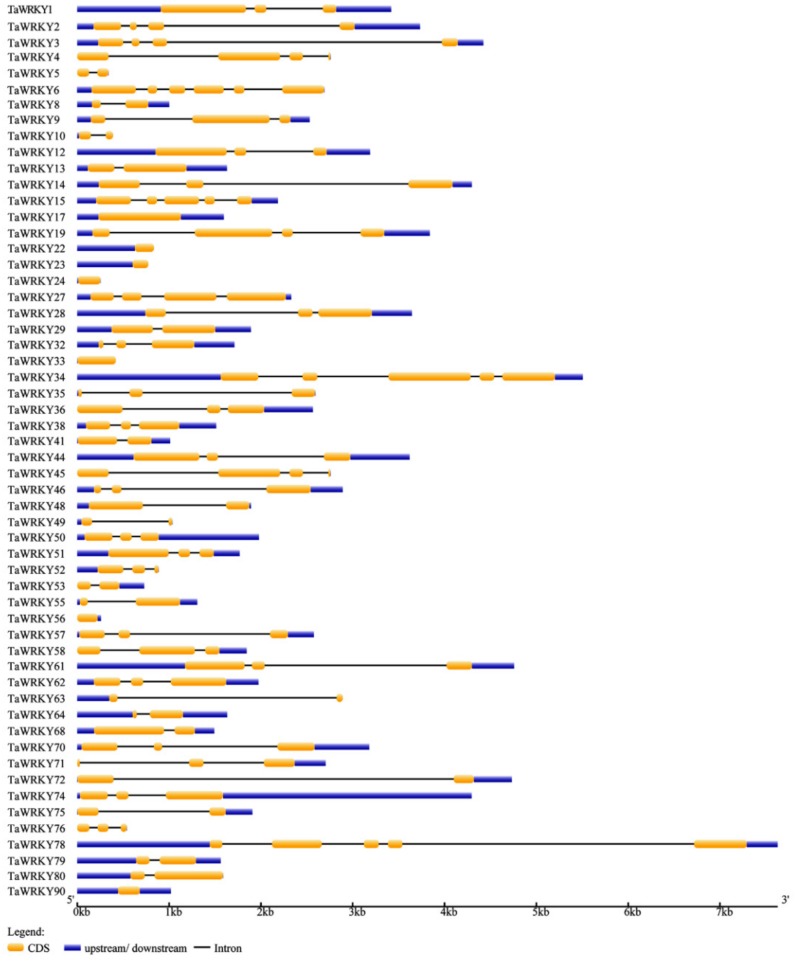
Gene structure analysis of TaWRKYs. Segments in yellow represent CDS, blue indicates upstream/downstream, and black lines represent introns.

According to the Plant Transcription Factor Database website (http://planttfdb.cbi.pku.edu.cn/index.php), wheat has 171 TaWRKYs, which are distributed across all chromosomes (1AL, 1BL, 1DL, 2AL, 2AS, 2BS, 2DL, 2DS, 3AL, 3B, 3DL, 4AL, 4AS, 4DS, 5AL, 5BS, 5BL, 5DL, 6AL, 6AS, 6BS, 6DS, 7AL, 7DL). Here, PF03106 was used as a key word to blast WRKYs in wheat on the Phytozome website (https://phytozome.jgi.doe.gov/pz/portal.html). Nucleic acid and amino acid sequences of 100 TaWRKYs that harbor at least one WRKY domain are shown in Supplementary Table S2. Based on the rule that the CDS of TaWRKYs were more than 300 base pairs [30], some TaWRKYs were removed, and then combined with the NCBI database (https://www.ncbi.nlm.nih.gov/pubmed), meaning that 57 TaWRKYs were identified with the annotation gene’s name, ID, transcript name and location (Table 1). The location of 57 TaWRKYs on chromosomes was analyzed by using the online website http://mg2c.iask.in/mg2c_v2.0/. From the map, we can see that the locations of TaWRKYs were different on each chromosome; for example, *TaWRKY6*, *38*, *50*, *27, 48*, and *57* were located at the end of chromosome 3B (forward or reverse), while *TaWRKY70* and *TaWRKY 71* were located near the centromere of chromosome 1D. Moreover, the distribution of TaWRKYs on 4A was the combination of both distributions described above (Figure 1). To further explore gene structure differences, a gene structure figure of 56 TaWRKYs is displayed in Figure 2. The TaWRKYs are all different in structure. Most TaWRKYs contain 1 to 5 different exons, which may contain different functional structures, such as zinc finger, leucine, kinase structure, exerting different biological functions. TaWRKY7, 22, 23, 24, 33, 56, and 90 do not harbor introns, only containing exons and/or an upstream structure.

### 2.2. Identification and Biological Analysis of TaWRKY13

**Figure 3:**
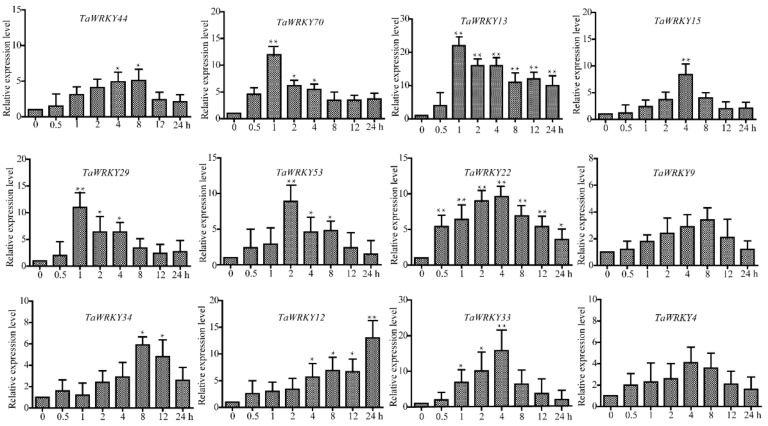
Real-time fluorescence quantification PCR of 12 TaWRKYs under salt treatment. The expression level of TaActin was used as a loading control. The data represent the means ± SD of three biological replications. The ANOVA demonstrated significant differences (* p < 0.05, ** p < 0.01).

To find wheat stress-responsive genes under salt stress, the roots of three-leaf wheat seedlings were immersed in 150 mM NaCl solution for 1 h. Control_Leaf represents the leaf tissue without NaCl treatment, NaCl_Leaf represents the leaf tissue treated as per the above description; each treatment involved two independent replicates which were then sampled for RNA-seq (Supplementary Table S1). Twelve TaWRKYs (TaWRKY4, 9, 12, 13, 15, 22, 29, 33, 34, 44, 53, and 70) were selected based on the rule log_2_ (NaCl_Leaf /Control_Leaf) > 2. As shown in Figure 3, *TaWRKY13* gave the highest relative expression in response to salt stress, peaking at more than 20-fold at 1 h. *TaWRKY13* (ID: 31962353, Traes_2AS_6269D889E.1) was selected for further investigation. *TaWRKY13* contained a 975 bp open reading frame (ORF) encoding 324 amino acids; the molecular weight of the protein was 81.02 kDa with pI 4.99 (https://web.expasy.org/protparam/). The predicted amino acid sequence showed that *TaWRKY13* only harbored one WRKY domain with a highly conserved WRKYGQK motif and a CX4-5CX22-23HXH zinc-finger motif.

### 2.3. Phylogenetic Analysis of AtWRKYs, OsWRKYs and TaWRKYs

**Figure 4:**
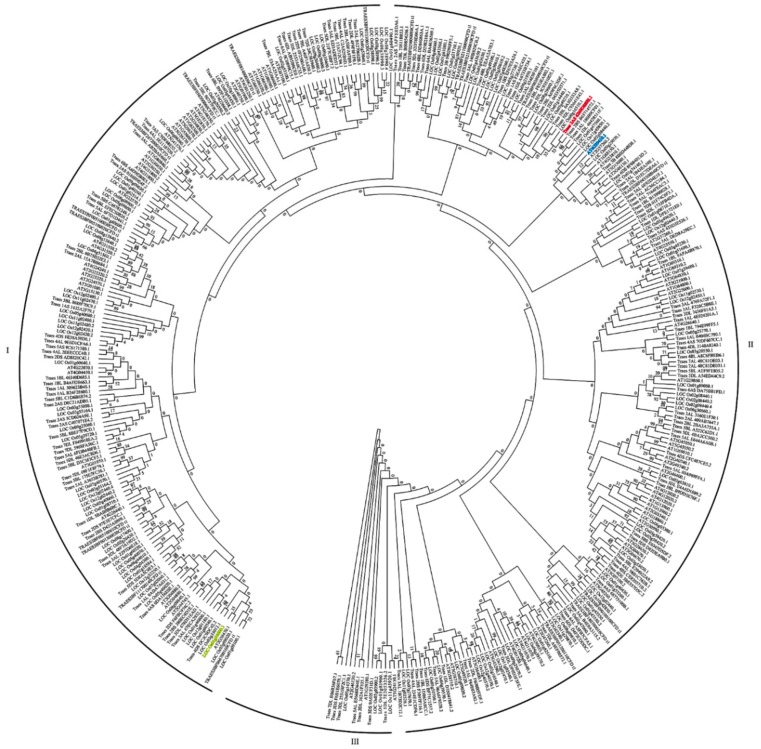
Phylogenetic analysis of AtWRKYs, OsWRKYs and TaWRKYs. The phylogenetic tree was produced using the aligned file with 1000 bootstrap replications in MEGA 6.0. *TaWRKY13, AtWRKY13* and *OsWRKY13* are highlighted in red, blue and yellow, respectively. The numbers at nodes are bootstrap values, and the length of branches represent evolutionary distance. Number of bootstrap replications: 1000.

Phylogenetic analysis is a useful method that can provide some clues to the possible functions of predicted or analyzed target genes. It would be useful to know the homologs of *Triticum aestivum* WRKYs (TaWRKYs), especially *TaWRKY13*, with WRKYs of *Arabidopsis thaliana* (AtWRKYs) and WRKYs of *Oryza sativa* (OsWRKYs) with reference to previous results. A phylogenic tree was constructed by the neighbor-joining method [39] to investigate the evolutionary relationships between AtWRKYs, OsWRKYs and TaWRKYs. There are 398 WRKYs for phylogenetic analysis (90 AtWRKYs, 128 OsWRKYs and 171 TaWRKYs) (Figure 4). According to Figure 4, AtWRKYs, OsWRKYs and TaWRKYs were scattered across different branches of the phylogenic tree, and all WRKYs were divided into three broad categories; among them, there were more WRKYs in groups I and II than in group III. *TaWRKY13* (ID: Traes_2AS_6269D889E.1) and *AtWRKY13* (ID: AT4G39410) were in group II, and *OsWRKY13* (ID: LOC-Os01g546600) belonged to group I. The results of phylogenetic analysis preliminarily indicated that *TaWRKY13* has a closer homology with *AtWRKY13* than *OsWRKY13*.

### 2.4. TaWRKY13 was Localized in the Nucleus

**Figure 5:**
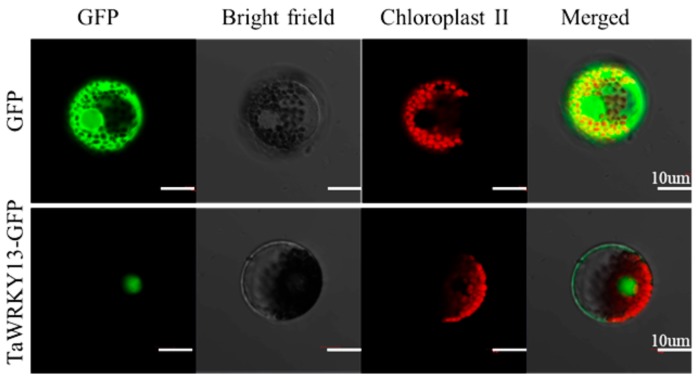
Subcellular localization of TaWRKY13. 35S::GFP and 35S::TaWRKY13-GFP constructs were transformed into wheat mesophyll protoplasts under the control of the Cauliflower Mosaic Virus 35S (CaMV35S) promoter. Wherein, green color represents fluorescence emitted by green fluorescent protein under confocal laser scanning microscope and the red color represents the fluorescence emitted by chloroplasts under confocal laser scanning microscope. Results were observed by a confocal laser scanning microscope (LSM700; CarlZeiss, Oberkochen Germany) after incubation in darkness at 22 °C for 18–20 h. Scale bars, 10 μm.

To investigate the biological activity of *TaWRKY13*, the coding sequence fused to the N-terminus of the green fluorescent protein (GFP) was inserted into wheat mesophyll protoplasts by the PEG-mediated method. As the control, 35S::GFP was transformed [40]. The fluorescence of the control GFP was distributed throughout the cells, whereas the fluorescence of 35S::TaWRKY13-GFP was specifically localized in the nucleus (Figure 5). Thus, TaWRKY13 is a nuclear-located protein. 

### 2.5. Tissue-Specific Expression of TaWRKY13

**Figure 6:**
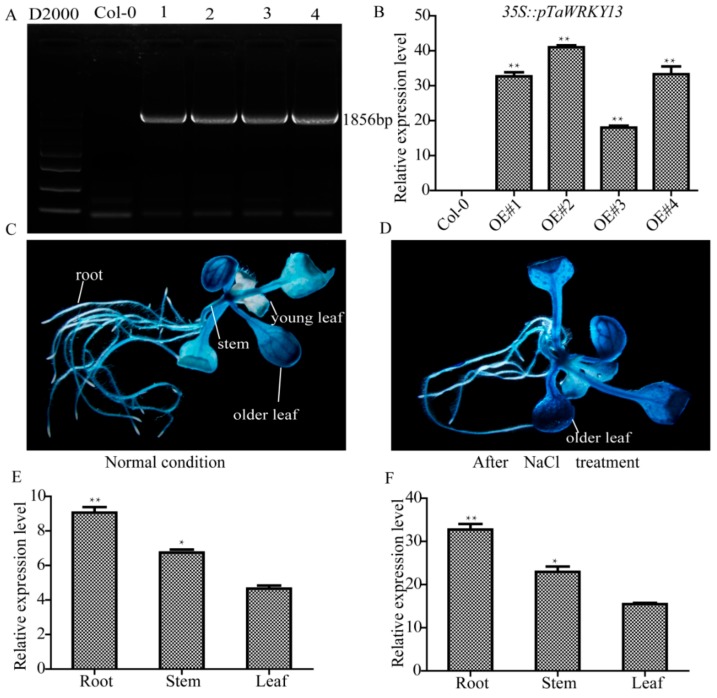
Tissue-specific expression analysis of *TaWRKY13*. (**A**) Identification of homozygous lines by agarose gel electrophoresis. (**B**) Three transgenic lines selected by RT-qPCR. (**C**) β-glucuronidase (GUS) staining of transgenic *Arabidopsis* under normal conditions. (**D**) GUS staining of transgenic *Arabidopsis* after NaCl treatment. (**E**) qRT-PCR for tissue-specific expression analysis of *TaWRKY13* under normal conditions. (**F**) qRT-PCR for tissue-specific expression analysis of *TaWRKY13* after NaCl treatment. All data are means ± SDs of three independent biological replicates. The ANOVA demonstrated significant differences (* *p* < 0.05, ** p < 0.01).

Studies of genes with a specific expression in different tissues are necessary to understand the regulatory mechanisms of plant growth and development and the relationship between cell type and function. Here, the promoter sequence of *TaWRKY13* was fused to the pCAMBIA1305 vector, which contains a β-glucuronidase (GUS) reporter gene in the N-terminus (Figure 6). The GUS reporter gene can preliminarily determine the tissue specificity of the gene by observing the tissue location with a blue color after staining [41]. qRT-PCR was used to further verify the relative expression level at the molecular level. *TaWRKY13* was expressed in the roots, stems and leaves of T_3_ generation transgenic *Arabidopsis* plants under normal and salt-stress conditions, with the relative expression in roots being higher than in leaves and stems. After NaCl treatment, the expression levels in roots, stems and leaves were significantly increased, indicating that *TaWRKY13* might be responsive to salt stress.

### 2.6. TaWRKY13 Is Involved in Various Stress Responses

**Figure 7:**
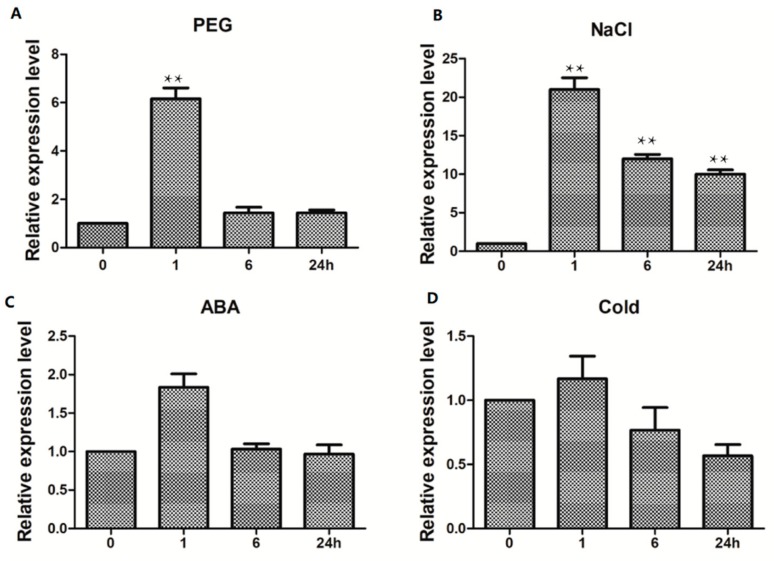
Expression patterns of *TaWRKY13* under (**A**) PEG, (**B**) NaCl, (**C**) exogenous abscisic acid (ABA), and (**D**) cold treatments. The ordinates are relative expression levels (fold) of *TaWRKY13* compared to the non-stressed control. The horizontal ordinate is the treatment time, at 0, 1, 6 and 24 h. The expression level of TaActin as a loading control. All experiments were repeated three times. Error bars represent standard deviations (SDs). All data are means ± SDs of three independent biological replicates. The ANOVA demonstrated significant differences (** p < 0.01).

WRKY proteins are reported to be involved in various biotic and abiotic stresses [25]. Expression pattern analyses were conducted to determine whether *TaWRKY13* was responsive to abiotic stresses. The results indicated that *TaWRKY13* participated in salt PEG, ABA and cold-stress responses (Figure 7). For PEG treatment, the relative expression level of *TaWRKY13* was rapidly induced at 1 h after the imposition of PEG stress (Figure 7A). After NaCl treatment for 1 h, *TaWRKY13* was highly induced at a maximum level of about 22-fold (Figure 7B). Exogenous ABA and cold stress also significantly affected the expression of *TaWRKY13* (Figure 7C,D). The rapid increase in relative expression levels of *TaWRKY13* following different stress treatments indicated an important role at the initial stages of stress response.

### 2.7. Stress-Related Regulatory Elements in the Promoter of TaWRKY13

The 1.856 kb promoter region upstream of the *TaWRKY13* ATG start codon was isolated to gain an insight into the regulatory mechanism. We searched for putative cis-acting elements in the promoter regions using the database PLACE (http://www.dna.affrc.go.jp/PLACE/). The results are shown in Table 2. Numerous stress-related regulatory elements were present, including a W-BOX, MYB element and TATA-BOX, which take part in the response to both drought and high-salt stress, as well as low-temperature responsive (LTR), ABA-responsive element (ABRE) and GT1, which mainly participate in salt-stress response. Moreover, there were various light, gibberellin, SA (salicylic acid) and high-temperature responsive elements, indicating that *TaWRKY13* is involved in abiotic stress response and plant hormone-related signal transduction.

### 2.8. Root System Analysis Indicates That Overexpression Lines Respond to Salt Stress in Arabidopsis

**Figure 8:**
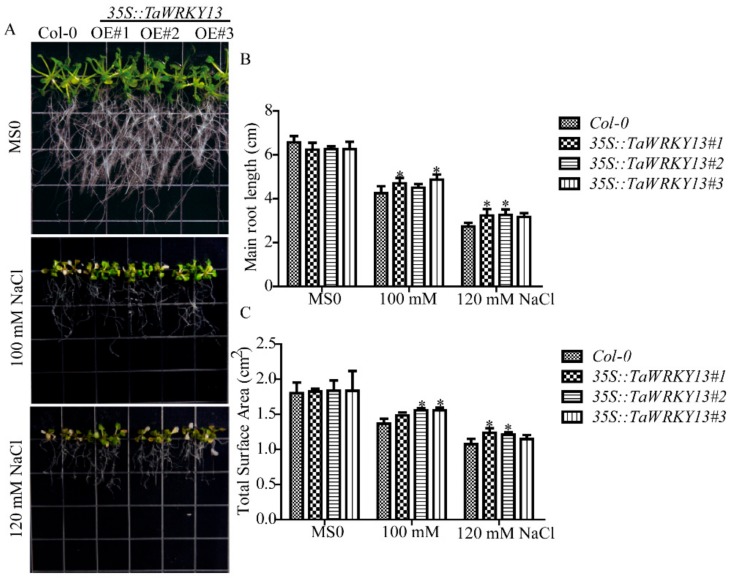
Root length phenotypes of *Arabidopsis* overexpression lines after NaCl treatment. (**A**) Image of the root length phenotype of transgenic lines grown in 0, 100 and 120 mM NaCl. (**B**) Analysis of the main root lengths of transgenic lines under NaCl treatment. (**C**) Analysis of total surface areas of transgenic lines under NaCl treatment. The main root length and total surface area of *Arabidopsis* roots were measured by the WinRHIZO system. All data are means ± SDs of three independent biological replicates. The ANOVA demonstrated significant differences (* *p* < 0.05).

To explore the mechanism of *TaWRKY13* under salt stress, a pCAMBIA1302-*TaWRKY13 (35S::TaWRKY13)* vector was constructed and transformed into *Arabidopsis* for root length assay [40]. The results of the identification of homozygotes by agarose gel electrophoresis (AGE) and the selection of three transgenic lines (*35S::TaWRKY13#1*, *#2*, *#3*) by RT-qPCR are available in Supplementary Figure S1. Seedlings of control (Columbia-0) and three T_3_ generation overexpression lines were first grown on MS (Murashige & Skoog) medium for one week and then transplanted to MS medium supplemented with various NaCl concentrations (0, 100, 120 mM) for salt treatment. As shown in Figure 8, the overexpression lines have an advantage in terms of the main root length and total surface area compared to Col-0 under NaCl treatment. 

### 2.9. TaWRKY13 Overexpression Response to Salt Stress in Oryza sativa

**Figure 9:**
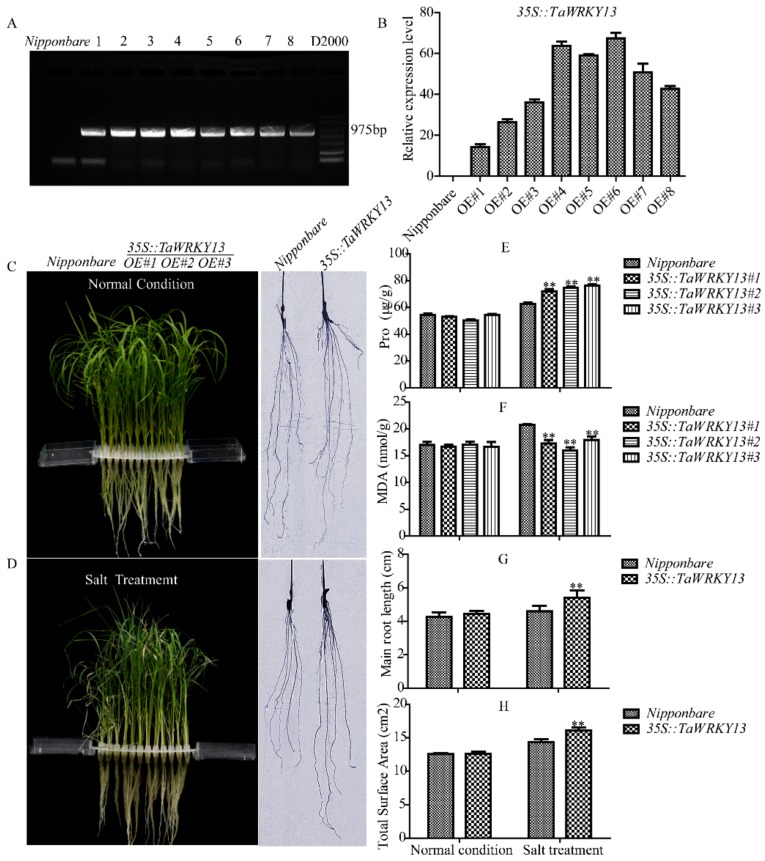
Phenotype identification of *TaWRKY13* transgenic rice under NaCl treatment. (**A**) Confirmation of homozygotes by agarose gel electrphoresis. (**B**) Selection of three transgenic lines by RT-qPCR. (**C**) Rice seedlings and root system diagram of Nipponbare and *35S::TaWRKY13* before treatment. (**D**) Rice seedlings and root system diagram of Nipponbare and *35S::TaWRKY13* after 150 mM NaCl treatment for 7 days. (**E**) Proline contents in Nipponbare and *35S::TaWRKY13* seedlings under normal conditions and NaCl treatment. (**F**) Malondialdehyde (MDA) contents in in Nipponbare and *35S::TaWRKY13* rice seedlings under normal growth conditions and NaCl treatment. (**G**) Root length measurements of Nipponbare and *35S::TaWRKY13* transformants with and without NaCl treatment. (**H**) Total surface areas of Nipponbare and *35S::TaWRKY13* with and without NaCl treatment. Main root lengths and total surface areas were measured by the WinRHIZO system (Hang xin, Guangzhou, China). All data are means ± SDs of three independent biological replicates. The ANOVA demonstrated significant differences (** *p* < 0.01).

Two-week-old T_3_ rice lines seedlings of the control (Nipponbare) and three overexpression lines (*35S::TaWRKY13#1*, *#2*, *#3*) were grown hydroponically in untreated control solution or in the same solution supplemented with 150 mM NaCl to explore the physiological tolerance of *TaWRKY13* overexpression rice lines to salt stress [42]. The verification of homozygotes and the selection of three transgenic lines were conducted by AGE and RT-qPCR, respectively (Figure 9A,B). As shown in Figure 9C, before NaCl treatment, both Nipponbare and the three transgenic lines showed similar growth patterns, with no or little difference in plant height, root length, and proline (Pro) and malondialdehyde (MDA) contents. After 7 days of NaCl treatment, both Nipponbare and the overexpression lines showed leaf shedding (Figure 9D). Compared with the transgenic lines, Nipponbare plants showed evidence of wilting, water loss and yellowing, whereas the transgenics lines showed less severe symptoms. Meanwhile, the overexpression of *TaWRKY13* increased the proline content and decreased MDA content under NaCl treatment (Figure 9E,F). The root length of Nipponbare was significantly lower than for transgenic plants; the surface areas of transgenic plants were higher than for Nipponbare (Figure 9G,H). These results indicated that the overexpression of *TaWRKY13* enhanced salt tolerance in rice.

## 3. Discussion

Regarded as one group among many important transcription factors in plants, WRKY TFs are represented by 90 members in *Arabidopsis* and more than 100 in rice [43]. The functions of WRKY TFs have been studied in detail in various plant species since their first discovery.

Since the application of transcriptome sequencing technology, researchers have sequenced the genome of wheat [44,45]. However, owing to the large and complex genome of heterohexaploid wheat, the task has posed many challenges [46]. Recently, transgenic *Arabidopsis* plants of *TaWRKY2* and *TaWRKY19* have shown improved stress tolerance, and the overexpression of *TaWRKY2* and *TaWRKY19* has exhibited salt, osmotic/dehydration and freezing stress tolerance [47]. More than 160 TaWRKYs were characterized according to their sequence alignment, motif type and phylogenetic relationship analysis by Sezer et al. [48]. Although the WRKY genes associated with stress can be identified by transcriptome sequencing and family analysis, functional identification and mechanism analysis in wheat is limited. Salt stress is one of the most serious stresses that cannot be reversed after damage [49].

Here, on the basis of the previous research, combining RNA-Seq, real-time quantitative PCR (RT-qPCR), and the latest wheat database, *TaWRKY13* was isolated from the wheat genome for further study. RNA-Seq was conducted first (Supplementary Table S1); meanwhile, using the wheat database, 57 TaWRKY genes were annotated (Table 1). The results showed that TaWRKYs were differently distributed (number and location) on wheat chromosomes (Figure 1). Studies of the genome structure and the phylogenetic analysis of TaWRKY genes were initially difficult, because the wheat genome was too complex for statistical analysis; there were 171 TaWRKY genes according to the database (https://phytozome.jgi.doe.gov/pz/portal.html). Based on the rule that the CDS of TaWRKYs were more than 300 base pairs, we removed redundant TaWRKY genes and, combined with the NCBI database (https://www.ncbi.nlm.nih.gov/pubmed), 56 TaWRKY genes were selected for the analysis of the gene structure (Figure 2). Major TaWRKY genes harbored different CDS and binding motifs responsible for special function; for example, *TaWRKY1* contained an N-terminal CUT domain and a C-terminal NL domain [30]. To further explore TaWRKY genes that respond to salt stress, 12 TaWRKY genes were chosen for verification by qRT-PCR (Figure 3). All 12 genes were up-regulated under salt stress, and *TaWRKY13* was chosen for further study due to its higher expression level under salt treatment. Phylogenetic analysis demonstrated that TaWRKY genes have different evolutionary relationships and homologies to WRKYs in *Arabidopsis* and rice (Figure 4); compared to *OsWRKY13, AtWRKY13* was closer to *TaWRKY13,* possibly indicating similar biological functions [50]. For *OsWRKY13,* the non-conservation of evolution may provide a basis for the subsequent functional identification of *TaWRKY13* in rice, in that the influence of rice itself in *OsWRKY13* was eliminated. Subcellular localization showed that TaWRKY13 is a nuclear protein (Figure 5) which may mainly be involved in nuclear signal transduction [51,52]. Although many cotton (*Gossypium hirsutum*) *WRKY* genes were expressed at low levels during development, a few *GhWRKYs* expressed highly in specific tissues such as roots, stems, leaves and embryos [53]. Our results showed that *TaWRKY13* was expressed in roots, stems and leaves in transgenic lines, the relative expression level of roots was higher than stems and leaves in transgenic lines, and under salt-stress conditions, the relative expression level was double that of the normal condition (Figure 6).

An increasing number of studies have shown that WRKY TFs play important roles in abiotic stress response; for instance, the overexpression of *GmWRKY21* improved cold tolerance in *Arabidopsis*, because of the regulation of DREB2A and STZ/Zat10. *GmWRKY54* conferred salt and drought tolerance; *GmWRKY13*, which was insensitive to ABA (abscisic acid) but markedly sensitive to salt and mannitol, may function in both lateral root development and the abiotic stress response [54]. Expression pattern analyses revealed that *TaWRKY13* was induced significantly by PEG, salt, low-temperature and ABA (Figure 7). Compared with PEG, low-temperature, and ABA stress, *TaWRKY13* achieved the highest relative expression level under salt treatment, which was in accordance with the following root length assay in *Arabidopsis* and the rice resistance assay. Products of WRKY TFs bind to specific cis-regulatory sequences such as the W-BOX in the promoter to induce the expression of downstream target genes [55]. Many regulatory cis-elements that are responsive to drought (W-BOX, MYB and TATA-BOX), high salt (LTR, ABRE and GT1), SA (salicylic acid, WRKY) and cold were recognized in the *TaWRKY13* promoter, showing that *TaWRKY13* is capable of responding to stress (Table 2). WRKY13 participated in various physiological processes; for example, a weaker stem phenotype, reduced sclerenchyma development, and altered lignin synthesis were observed in an *AtWRKY13* mutant, showing that it functioned in stem development [56]. When *AtWRKY13* was disturbed under short-day conditions, *AtWRKY13* promoted flowering [57]. Furthermore, WRKY13 was also involved in the cross talk between abiotic and biotic stress signaling pathways, and *OsWRKY13* displayed selective binding to different cis-elements to regulate various stress [58]. In this study, a root length assay of overexpression lines was conducted in *Arabidopsis* for an analysis of the stress tolerance of *TaWRKY13*; overexpression lines had longer root lengths and a higher total root area than Col-0 (Figure 8A–C). Additionally, the overexpression of *TaWRKY13* enhanced salt tolerance in transgenic rice (Figure 9). Under NaCl treatment, the transgenic lines of *TaWRKY13* grew vigorously, whereas Nipponbare seedlings were more wilted and yellow (Figure 9D); the transgenic lines also had higher proline (Pro) and reduced malondialdehyde (MDA) contents (Figure 8F and Figure 9E ) under NaCl treatment. In addition, the roots of transgenic lines were longer and more developed than Nipponbare (Figure 9G,H). These results all showed that *TaWRKY13* was responsive to salt stress, in agreement with data from other species [54,56,58]. In accordance with the present study, the results suggested that *TaWRKY13* has a potential role in improving salt tolerance in wheat. These results are only preliminary in exploring the putative role of *TaWRKY13* in salt tolerance; more researches about the role and regulation mechanism of *TaWRKY13* are still needed in wheat. For instance, based on the above findings, *TaWRKY13* was transformed into wheat for functional verification and mechanism analysis to further improve the role of *TaWRKY13* in wheat stress tolerance pathways.

## 4. Materials and Methods

### 4.1. De Novo Transcriptome Sequencing of Salt-Treated Wheat

Wheat (*Triticum aestivum* L. cultivar Jinhe 9123, from the Institute of Genetics and Physiology, Hebei Academy of Agriculture and Forestry Sciences, Shijiazhuang, China) was cultivated in a 10 cm × 10 cm pot (vermiculite:soil, 1:3) supplemented with Hoagland’s liquid medium at 22 °C under a 16 h light/8 h darkness photoperiod for 10 days. When the wheat seedlings were at the three-leaf stage, the pots were immersed in 150 mM NaCl solution and water (control) for 1 h, respectively [30], prior to the sampling of 0.1 g fresh leaf tissue. Samples were submerged immediately in liquid nitrogen and stored at −80 °C for RNA-Seq. The experiment was performed in three independent replications. In Supplementary Table S1, Control_Leaf means a sample without NaCl treatment, and NaCl_Leaf means a sample with salt treatment; each treatment involved three independent replicates, which were then sampled for RNA-Seq. Data are shown in Supplementary Table S1.

### 4.2. Identification and Annotation of TaWRKY Response to Salt Stress

According to the RNA-Seq data, the rule was adopted that the expression level was up-regulated and log_2_(NaCl_treat/Control_treat) > 2 to select TaWRKYs which responded to salt stress. Several databases—NCBI (https://www.ncbi.nlm.nih.gov/pubmed) PlantTFDB (http://planttfdb.cbi.pku.edu.cn/) and Phytozome (https://phytozome.jgi.doe.gov/pz/portal.html)—were used to annotate the gene name, ID, transcript name and localization.

### 4.3. Structure Analysis and Phylogenetic Analysis of TaWRKYs

According to the information listed in Table 1, the chromosome location of TaWRKYs was analyzed by using the online website http://mg2c.iask.in/mg2c_v2.0/. For gene structure analysis, the data of TaWRKYs that were identified in Section 4.2 were uploaded to GSDS (http://gsds.cbi.pku.edu.cn/*)* to obtain the map of the TaWRKYs’ structure. For phylogenetic analysis, a tree of WRKYs from wheat, rice and *Arabidopsis* was constructed using the neighbor-joining method in MEGA 6.0 with 1000 bootstrap replications [39]. Data for gene structure and the phylogenetic tree analysis were downloaded from PlantTFDB (http://planttfdb.cbi.pku.edu.cn/) and are shown in Supplementary Tables S2 and S3.

### 4.4. RNA Extraction of Stress Treatments and RT-qPCR Analyses

Wheat seeds were sown as previously described; vermiculite and soil were removed by water after being grown for 10 days, and the fresh leaf tissue of three-leaf-stage wheat seedlings were used for the RNA extraction of different stress treatments. For the identification of TaWRKY responses to salt stress, the seedlings roots were immersed in 150 mM NaCl solution, and 0.1 g of fresh leaf tissue was sampled at different times (0, 0.5, 1, 2, 4, 8, 12 and 24 h). For the expression pattern analyses, the roots of wheat seedlings were immersed in 10% PEG6000, 150 mM NaCl and 100 μmol·L^−1^ ABA solutions. Wheat seedlings for cold treatment were placed in a 10 h light/14 h darkness, 4/2 °C chamber and sampled at different periods (0, 1, 6 and 24 h) [30,59,60]. For specific tissue expression assays, T_3_ generation transgenic *Arabidopsis* (*35S::pTaWRKY13*) plants were surface-sterilized with 10% Chloros and washed three times with sterile water. Sterilized seeds were sown on MS (Murashige & Skoog) medium, vernalized in darkness for 3–4 days at 4 °C, then grown in a chamber at 22 °C and 75% humidity under a 16 h light/8 h darkness photoperiod for one week. The seedings were transplanted to soil (vermiculite:soil, 1:3), 0.1 g fresh roots, stems and leaves tissue of 10-day-old transgenic *Arabidopsis* seedlings with or without 150 mM NaCl treatment were sampled for RNA the extraction of different tissues [56].

All samples after collection were submerged immediately in liquid nitrogen and stored at −80 °C for RNA extraction using an RNA prep plant kit (TIANGEN, Beijing, China); cDNA was synthesized using a Prime Script First-Strand cDNA Synthesis Kit (TransGen, Beijing, China) following the manufacturer’s instructions. RT-qPCR was performed with Super Real PreMix Plus (TransGen, Beijing, China) on an ABI Prism 7500 system (Applied Biosystems, Foster city, CA, USA). Specific primers for TaActin, AtActin and TaWRKY4, 9, 12, 13, 15, 22, 19, 33, 34, 44, 53 and 70 for RT-qPCR are listed in Supplementary Table S4. Three biological replicates were used for RT-qPCR analysis, and the 2^−∆∆Ct^ method was used for quantification.

### 4.5. Gene Isolation and Subcellular Localization

The ORF (open reading frame) of *TaWRKY13* was amplified by PCR with specific primers from wheat cDNA (cultivar Jinhe 9123). The PCR product was fused into pZeroBack vector (TIANGEN, Beijing, China) and sequenced for further study. The correct sequencing plasmids were treated as templates, the segment with restriction sites was amplified by specific primers, and the PCR product was inserted into the N-terminus of the green fluorescent protein (GFP) containing the CaMV35S promoter for subcellular localization; the 35S::GFP vector was used as the control. Both 35S::GFP and 35S::TaWRKY13-GFP were transferred into wheat mesophyll protoplasts by the PEG-mediated method [29]. A confocal laser scanning microscope (LSM700; CarlZeiss, Oberkochen, Germany) was used to observe the fluorescence after incubation in darkness at 22 °C for 18–20 h. All primers are listed in Supplementary Table S4.

### 4.6. Tissue-Specific Expression of TaWRKY13 and GUS Staining

Tissue-specific expression analysis of *TaWRKY13* was conducted by two methods. In the first one, the CDS of *TaWRKY13* was amplified as described in Section 4.5, then cloned into the pCAMBIA1302 vector; then, the infected inflorescence of *Arabidopsis* was determined by the Agrobacterium-mediated method [61], grown as described in Section 4.4, until T_3_ generation transgenic *Arabidopsis* seeds were obtained. The identification of homozygotes and selection of three transgenic lines were conducted by agarose gel electrophoresis and RT-qPCR, respectively [59]. The transgenic *Arabidopsis* seedlings with or without NaCl (150 mM) treatment were used for RT-qPCR as described in Section 4.4. In the second method, promoter fragments of *TaWRKY13* (*pTaWRKY13*) were obtained from Ensemble Plants (plants.ensembl.org/index.html); the *pTaWRKY13* was amplified by PCR with specific primers from wheat cDNA (Jinhe 9123), and the PCR product was fused into pLB vector (TIANGEN, Beijing, China) and sequenced. The fragment of *TaWRKY13* promoter was cloned to the pCAMBIA1305 vector harboring a β-glucuronidase (GUS) tag, obtaining the T_3_ generation transgenic *Arabidopsis* seeds as per the previous description. T_3_ generation transgenic *Arabidopsis* (*35S::pTaWRKY13*) seeds were surface-sterilized, sown on MS medium, vernalized, and grown in a chamber at 22 °C and 75% humidity under a 16 h light/8 h darkness photoperiod for one week as described in Section 4.4. Ten-day-old transgenic *Arabidopsis* seedlings were submerged to 150 mM NaCl solution for 1 h. After salt treatment, the liquid was drained with filter paper, and the plant material was subjected to GUS staining solution supplemented with 5-bromo-4-chloro-3-indolylb-d-glucuronic acid (X-gluc) for 3 h; 70% (*vol*/*vol*) ethanol was used to remove the chlorophyll following the manufacturer’s protocol (Real-Times, Beijing, China) [56]. GUS staining was observed by a Leica microscope (Wetzlar, Germany). Primers are listed in Supplementary Table S4.

### 4.7. Cis-Acting Elements in the TaWRKY13 Promoter

A 1.856 kb promoter fragment upstream of the ATG start codon of *TaWRKY13* was obtained from the Ensemble Plants website (http://plants.ensembl.org/index.html). Cis-acting elements that respond to various stresses in the promoter region were analyzed by PLACE (http://www.dna.affrc.go.jp/PLACE/) [29].

### 4.8. Root Growth Assays of TaWRKY13 under Salt Stress in Arabidopsis

T_3_ generation transgenic *Arabidopsis* lines were obtained as previously described (Section 4.6). Seeds of Col-0 and transgenic lines (*35S::TaWRKY13#1, #2, #3*) were surface-sterilized, sown on MS medium, vernalized, grown in a chamber at 22 °C and 75% humidity under a 16 h light/8 h darkness photoperiod for one week as described above (Section 4.4). Three ten-day-old *Arabidopsis* seedlings (Col-0 and transgenic lines) were transferred to MS medium containing different concentrations of NaCl (0, 100, 120 mM) for one week [40].

### 4.9. Generation of Transgenic Rice and Stress Identification of TaWRKY13 to Salt Tolerance

Plant expression vector *pCAMBIA1305-TaWRKY13* was constructed and transformed to competent cells of EHA105 as previously described [30]. Genetic transformation was conducted by Dr Chuan-Yin Wu and colleagues at the Institute of Crop Science, Chinese Academy of Agricultural Sciences using the agrobacterium-mediated method, and Nipponbare was used as the control [62]. The selection of three transgenic lines was made by agarose gel electrophoresis and RT-qPCR, respectively, as previously described (Figure S1). T_3_ generation transgenics (*35S::TaWRKY13#1*, *#2*, *#3*) and Nipponbare were used for further study. Rice seeds were treated with 0.7% hydrogen peroxide for one day for surface sterilization, breaking dormancy and promoting germination, then replaced with 0.7% hydrogen peroxide with water and germinated at 37 °C for 3 days (changing the water once a day). When seeds showed white buds, bare seeds were transplanted to 96-well plates (24 seeds of Nipponbare and *35S::TaWRKY13#1,#2,#3*, respectively) and placed in a growth chamber at 28 °C and a 16 h light/8 h darkness photoperiod and 70% relative humidity for the hydroponic culture. Seedings were cultured in water for one week, then cultured in water supplemented with Hoagland’s hydroponic culture solution. The culture solution was replaced every 5 days, and the pH was set at 5.5 [63]. Three-leaf seedlings were treated. For salt treatment, the 96-well plates growing three-leaf stage seedlings were transferred to YS hydroponic culture solution and a YS hydroponic culture solution supplemented with 150 mM NaCl for several days until phenotypes appeared [62]. For each salt treatment, there were three independent replicates. Primers are listed in Supplementary Table S4.

### 4.10. Measurements of Proline (Pro) and Malondialdehyde (MDA) Contents

To better understand the function of *TaWRKY13* under salt treatment, proline and MDA contents were measured with Pro and MDA assay kits (Comin, Beijing, China) based on the manufacturer’s protocols. Main root lengths and total surface areas of *Arabidopsis* and rice roots were measured by the WinRHIZO system (Hang xin, Guangzhou, China). Measurements were made on all three biological replicates; means ± SD and statistically significant differences were based on the ANOVA (* *p* < 0.05, ** *p* < 0.01).

## 5. Conclusions

We identified the salt-induced WRKY gene *TaWRKY13* (ID: 31962353) from a wheat RNA-Seq database (https://phytozome.jgi.doe.gov/pz/portal.html) and real-time quantitative PCR (RT-qPCR). TaWRKY13 is a nuclear protein that was expressed in the roots, stems and leaves of transgenic *Arabidopsis. TaWRKY13* was responsive to PEG, salt, cold, and exogenous abscisic acid (ABA) treatment. The overexpression of *TaWRKY13* was responsive to salt stress in both *Arabidopsis* and rice, as evidenced by the promotion of root length and the total root surface area. These results provide a basis for further understanding the functions of *TaWRKY13* in wheat when subjected to salt stress.

## Figures and Tables

**Table 1:** Annotation of WRKY transcription factors in *Triticum aestivum.*

Name	ID	Transcript Name	Location
*TaWRKY28*	31740471	Traes_1BL_9AFA4B870.1	ta_iwgsc_1bl_v1_3809885:1110..4753 reverse
*TaWRKY15*	31742772	Traes_5BL_E294922A9.2	ta_iwgsc_5bl_v1_10867378:4708..6402 forward
*TaWRKY6*	31744736	Traes_3B_CDA5ADD75.1	ta_iwgsc_3b_v1_10758590:296..2989 forward
*TaWRKY62*	31745499	Traes_5DL_C93641E43.1	ta_iwgsc_5dl_v1_4576731:2459..4427 forward
*TaWRKY44*	31746115	Traes_4AL_2EEECCC4B.1	ta_iwgsc_4al_v2_7093101:3374..6818 forward
*TaWRKY74*	31747511	Traes_5DL_5C93510D5.1	ta_iwgsc_5dl_v1_4502975:3368..7661 reverse
*TaWRKY80*	31748920	Traes_6AS_DA75BB1FD.1	ta_iwgsc_6as_v1_4428654:1..1588 reverse
*TaWRKY53*	31752041	Traes_2DL_F600B5FDF.1	ta_iwgsc_2dl_v1_9719154:1..728 forward
*TaWRKY22*	31752743	Traes_5AL_6FDB440FB.1	ta_iwgsc_5al_v1_2705439:4..838 forward
*TaWRKY45*	31765470	Traes_7AL_48C81DE03.1	ta_iwgsc_7al_v1_4556343:539..3297 forward
*TaWRKY4*	31765472	Traes_7AL_48C81DE031.1	ta_iwgsc_7al_v1_4556343:3718..6476 reverse
*TaWRKY35*	31766778	Traes_6BL_EEAA2A7E3.1	ta_iwgsc_6bl_v1_4221964:3..2595 forward
*TaWRKY79*	31767242	Traes_7DL_B09854286.1	ta_iwgsc_7dl_v1_3393496:18..940 reverse
*TaWRKY46*	31768080	Traes_4DS_FE38A59D0.1	ta_iwgsc_4ds_v1_2280139:4533..7422 reverse
*TaWRKY57*	31782323	Traes_3B_41047D5E6.2	ta_iwgsc_3b_v1_10527462:3896..6406 reverse
*TaWRKY33*	31785825	Traes_6DS_8F684013D.1	ta_iwgsc_6ds_v1_1013038:1..419 reverse
*TaWRKY12*	31787421	Traes_6AL_BA4636569.1	ta_iwgsc_6al_v1_5754118:409..4140 reverse
*TaWRKY24*	31792629	Traes_4AL_C2A825B6D.1	ta_iwgsc_4al_v2_3841042:1..253 reverse
*TaWRKY63*	31793891	Traes_3DL_7456F61A3.1	ta_iwgsc_3dl_v1_5877113:2..2892 reverse
*TaWRKY68*	31798439	Traes_2AL_15A7BB684.1	ta_iwgsc_2al_v1_6374918:10015..11505 forward
*TaWRKY50*	31799212	Traes_3B_F45FCFE62.1	ta_iwgsc_3b_v1_10625585:4077..6054 forward
*TaWRKY58*	31811544	Traes_5BL_D3C383CF5.1	ta_iwgsc_5bl_v1_10787947:2038..3881 forward
*TaWRKY72*	31818595	Traes_2BS_F3097F116.1	ta_iwgsc_2bs_v1_5195103:6587..11319 forward
*TaWRKY8*	31823877	Traes_5DL_2553A6C33.1	ta_iwgsc_5dl_v1_4566006:8..1007 forward
*TaWRKY34*	31829399	Traes_2AL_409AB7647.1	ta_iwgsc_2al_v1_6334600:3412..8916 reverse
*TaWRKY9*	31836810	Traes_2DS_F6FBC974C.2	ta_iwgsc_2ds_v1_5331381:733..3264 reverse
*TaWRKY52*	31851405	Traes_3AL_AB2BAE660.1	ta_iwgsc_3al_v1_4270257:1..887 reverse
*TaWRKY3*	31853252	Traes_2DL_4F9F8F1F0.1	ta_iwgsc_2dl_v1_9906833:634..5055 reverse
*TaWRKY51*	31854913	Traes_2AL_434E9F101.1	ta_iwgsc_2al_v1_6367445:3985..5752 reverse
*TaWRKY27*	31865868	Traes_3B_990298FF5.1	ta_iwgsc_3b_v1_10750391:1..2331 reverse
*TaWRKY70*	31871499	Traes_1DL_DFE1721E0.1	ta_iwgsc_1dl_v1_2268423:4679..7857 forward
*TaWRKY41*	31872073	Traes_2DS_AD8820C42.1	ta_iwgsc_2ds_v1_5376167:6..1016 reverse
*TaWRKY14*	31872762	Traes_5BL_B9DD3E76F.1	ta_iwgsc_5bl_v1_10924584:9637..13927 forward
*TaWRKY17*	31875786	Traes_5BL_8688F70C9.1	ta_iwgsc_5bl_v1_10840877:2232..3827 reverse
*TaWRKY56*	31876237	Traes_3AL_DED8A29EC.1	ta_iwgsc_3al_v1_382150:704..961 reverse
*TaWRKY78*	31876678	Traes_2BS_D435A8999.1	ta_iwgsc_2bs_v1_5214231:8279..15893 reverse
*TaWRKY32*	31888413	Traes_4AS_70DF607CC.1	ta_iwgsc_4as_v2_352920:1884..3594 forward
*TaWRKY16*	31891223	Traes_4AL_98B1C762B.2	ta_iwgsc_4al_v2_7173949:3935..6881 forward
*TaWRKY48*	31892659	Traes_3B_B8BF316B8.2	ta_iwgsc_3b_v1_10433739:23..1890 reverse
*TaWRKY71*	31894510	Traes_1DL_46428511F.1	ta_iwgsc_1dl_v1_2235906:1905..4608 forward
*TaWRKY38*	31895081	Traes_3B_D6F86ABC3.2	ta_iwgsc_3b_v1_10762199:7310..8822 forward
*TaWRKY55*	31916438	Traes_6AS_68775100B.1	ta_iwgsc_6as_v1_4413209:7948..9254 forward
*TaWRKY76*	31917474	Traes_5DL_32D78D06A.1	ta_iwgsc_5dl_v1_4501324:900..1439 reverse
*TaWRKY19*	31924920	Traes_2BS_380EC4D1E.1	ta_iwgsc_2bs_v1_5227909:9257..13033 reverse
*TaWRKY29*	31938855	Traes_1AL_4E924201A.1	ta_iwgsc_1al_v2_3969710:4988..6878 reverse
*TaWRKY10*	31942345	Traes_2DL_362A1F535.1	ta_iwgsc_2dl_v1_9707610:58..446 forward
*TaWRKY36*	31942939	Traes_3AL_140B829CB.2	ta_iwgsc_3al_v1_4308486:3673..5708 reverse
*TaWRKY2*	31951792	Traes_5BL_17A712C94.1	ta_iwgsc_5bl_v1_10916210:5033..9621 forward
*TaWRKY13*	31962353	Traes_2AS_6269D889E.1	ta_iwgsc_2as_v1_5205891:13214..14843 reverse
*TaWRKY1*	31966248	Traes_5BL_AEF9FE805.1	ta_iwgsc_5bl_v1_10827243:3081..6424 reverse
*TaWRKY49*	31968771	Traes_3DL_2551BF2C1.1	ta_iwgsc_3dl_v1_6811598:1..1035 reverse
*TaWRKY23*	31977027	Traes_3AL_4769A72F1.1	ta_iwgsc_3al_v1_805190:2..774 reverse
*TaWRKY75*	31987126	Traes_1AL_0404BC790.1	ta_iwgsc_1al_v2_3912777:3..1909 forward
*TaWRKY5*	31988149	Traes_5AL_7164FEAC3.1	ta_iwgsc_5al_v1_2204788:3..342 forward
*TaWRKY64*	32002393	Traes_4AS_0DA136E0E.1	ta_iwgsc_4as_v2_5962726:2807..4440 forward
*TaWRKY90*	32002429	Traes_3AL_1B73D2C12.1	ta_iwgsc_3al_v1_4248344:659..1678 forward
*TaWRKY61*	32024774	Traes_5BS_C46781248.1	ta_iwgsc_5bs_v1_2248873:15934..19692 reverse

Annotations were according to Phytozome (https://phytozome.jgi.doe.gov/pz/portal.html), PlantTFDB (http://planttfdb.cbi.pku.edu.cn/index.php and NCBI (https://www.ncbi.nlm.nih.gov/pubmed).

**Table 2:** *Cis*-element analysis of the *TaWRKY13* promotor.

*Cis*-Element	Target Sequences	Number	Function
W-BOX	TTGAC/TGACT TGACY/CTCAY	27	Drought, high salt responsive elements
MYB	GGATA/WAACCA/TAACARA/ TAACAAA/CCWACC/GNGTTR	20	Drought, high salt responsive elements
LTR	CCGAC/CCGAAA	7	Low-temperature, salt responsive elements
ABRE	ACGTGKC	5	ABA-responsive elements
TATA-BOX	TATATAA	6	Drought, cold, high salt responsive elements
GTI	CAAAAA	3	Salt responsive elements
GATA-BOX	GATA	22	Light, gibberellin responsive elements
WRKY	TAGA	20	Light, salicylic acid responsive elements
HSP70A	SCGAYNR(N)_15_HD	7	High temperature responsive elements

“Number” corresponds to the number of each type of cis-element in the promoter.

## Supplementary Materials

Supplementary materials can be found at https://www.mdpi.com/1422-0067/20/22/5712/s1. Supplementary Table S1: RNA-Seq data of salt treated wheat. Supplementary Table S2: CDS and amino acid sequences of 100 TaWRKYs used for genome structure and phylogenetic analysis. Supplementary Table S3: Amino acid sequences of 90 AtWRKYs, 128 OsWRKYs and 171 TaWRKY used for phylogenetic analysis. Supplementary Table S4: Primers used in this study. Supplementary Figure S1: Overexpression rice lines. A: Identification of homozygotes by agarose gel electrophoresis B: Selection of three transgenic lines by RT-qPCR.

## Abbreviations

ROS	Reactive oxygen species
SnRK2	Sucrose non-fermenting 1-related protein kinase 2
MAPK	Mitogen activated protein kinase
PKs	Protein kinases
TFs	Transcription factors
CDS	Coding sequence
ORF	Open reading frame
SA	Salicylic acid
ABA	Abscisic acid
ABRE	ABA-responsive element
Pro	Proline
MDA	Malondialdehyde
PEG	Polyethylene glycol
GFP	Green fluorescent protein
RT-qPCR	Real-time quantitative PCR
LTR	Low-temperature responsive
GUS	β-glucuronidase
X-gluc	5-bromo-4-chloro-3-indolylb-d-glucuronic acid
AGE	Agarose gel electrophoresis
MS	Murashige & Skoog
