# Supplementary material for: Characterizing the Role of TaWRKY13 in Salt Tolerance
Source: Int J Mol Sci. 2019 Nov 14;20(22):5712. doi: 10.3390/ijms20225712 (PMC6888956; doi:10.3390/ijms20225712)
Supplement: Supplementary file 1 [file ijms-20-05712-s001.zip › Supplementary Table 2.docx]

CDS sequences of 100 *TaWRKYs*

>Traes_1AL_0404BC790 Org_Taestivumearly-release CDS: Traes_1AL_0404BC790.1 (1 of 5) PTHR31221:SF7 - WRKY TRANSCRIPTION FACTOR 24-RELATED (PAC:31987126)

ATGGTGAGCGGGGCGGCGCCGCCGCCGCCGCCGGAGAGTGGTGCAGGGAGCAGTAGTGGCGTGGGAAGGGAGGAGACGAAAGGGAAGGGCAGTGCGCGCGGGCGGGGGAGCAGGAAGGCGAGCCGGCCGCGGTTCGCGTTCCAGACCAAGAGCGAGAACGACGTCCTCGACGACGGCTACCGGTGGAGGAAGTACGGCCAGAAGGCCGTCAAGAACAGCGCCTTCCCCAGGAGCTACTACCGGTGCACGCACCACACATGCAACGTGAAGAAGCAGGTGCAGCGGCTGGCCAAGGACACGAGCATCGTGGTGACCACGTACGAGGGCGTCCACAACCACCCCTGCGAGAAGCTCATGGAGGCCCTCAACCCCATCCTCAGGCAGCTTCAGTTCCTCTCACAGCTCTAA

>Traes_1AL_4E924201A Org_Taestivumearly-release CDS: Traes_1AL_4E924201A.1 (1 of 9) PTHR32096:SF18 - DISEASE RESISTANCE PROTEIN-LIKE-RELATED (PAC:31938855)

ATGCGTGACCTGTTTTGGCTGTCGCCGGGCGAGCAAGGAGATCTCTCCGATGTCGTCAGGGCAAGCCTGCACCCGCCTCATCAGCTGCCAACCCCGGCTGCCGACGAGGAGGAGGAGGACGAGTACAGCTCGCTGCTGCTGGAAGGAGGAGGCGGTGGAGGCGGCCTGGTCGTCGGCCATGGCGATGAGCAGCTGGGAATGGTGGCCATGATGATGGGCGGCAATAATAGTAGCCGGCCTCCTTCGTCTGATCATCACGTGATCTCGCTGCATTCACCGCCGGCGACGACATATACGCGGCCGCATCCAGAGCCGCTGGCCGGGATGCTTCGTCGGCCGGGTTTCGAGAGGGAAGGCGACATGGTGGTCGGGCCGCCGCCGGAGATAGGCGACCGCCTGCAGCACATGTCGATCGCCCATCACCCTCGTGTGCCTACCGCAATGAAGCCAAGGAAGAGCCAGTCGAAGAAGGTGGTGTGCATCCCGGCGCCGACGGCGGCGCCGGGAGCGAGCGGGCGGCATAGCACGAGCGGCGAGGTGGTGCCGTCGGACCTGTGGGCGTGGAGGAAGTACGGGCAGAAGCCCATCAAGGGGTCGCCGTACCCGAGGGGCTACTACCGCTGCAGCAGCTCCAAGGGGTGCCCGGCGAGGAAGCAGGTGGAGCGCAGCCGCACCGACCCCAACATGCTCGTCATCACCTACACCTCCGACCACAACCACCCGTGGCCGACCCAGCGCAACGCCCTCGCCGGATCAACCCGCCCGTCATCCTCCTCCGCCGCCGCCGCCAAGATCGCCGCCTCCTCTTCCTCTTCATTGGCGGCCGCGGCAGCTCGTAACAGTAGCAACACCAACGTCGACGTCGACTGTGCTGGTGCTCACCATCAGCTGAAGCAAGAGAGCGACCTGGACCTGTTCGCGGACATGGACGCCCTCAGCGTCTTCTCCTCCATCGACAAGATCCAGGAAGATGACAGCAAGCAGCAGCTGTTTGATCCTTTCAGCTCCGGCTTCTGCGACTACATCTAA

>Traes_1AL_F64E07A92 Org_Taestivumearly-release CDS: Traes_1AL_F64E07A92.1 (1 of 5) PTHR31221:SF23 - WRKY TRANSCRIPTION FACTOR 23-RELATED (PAC:31871735)

ATGAAGGAGGGGAAGAGGGAGAAGAAGCCGCGTGGGTCCCGTGTGGCATTCGCAACCAAGAGTGCGGTCGACCACCTCGACGACGGCTATCGCTGGCGCAAGTACGGCCAGAAGGCAGTCAAGAACAGCTCCTTCCCAAGGAGCTACTACCGGTGCACGGCGGCGCAGTGCGGGGTGAAGAAGCTGGTGGAGCGGTCACAGCAGGACCCGTCCACGGTCGTCACCACTTACGAGGGTCGCCACGCGCACCCGAGCCCCATCGCCACCCACCGTGGCTCGCGCATGCTAATGGCCACTGGTGTCGACACTGTCTACTCACTCGACGTGCTCCAGCATCAACATCATGGATTTTTCCCGGCCGGTACCGACGTCTATGGGCGCATGTACGCACTGCCCAGTACCGACGCCTCGGTGGTGGCACACCGGTCGTCCGAGTACGGTGGCATGCAGGTGCATGCCGGTGTTCTTCCCGATGCCGTGATGAGTTATGAGCATGTTCATCGTTAA

>Traes_1AS_1432A2F79 Org_Taestivumearly-release CDS: Traes_1AS_1432A2F79.1 (1 of 169) PF03106 - WRKY DNA -binding domain (WRKY) (PAC:31973080)

GGAGCCGGGGATGACGAACATCGCGGCGAGAAGAAGATCAAGATCAGTGCGAGGGTGAGCTCAGGGAGGATCGGGTTCAGGACGAGGTCAGAGGTGGAAATCTTGGACGACGGGTTCAAGTGGAGGAAGTACGGGAAGAAGGCGGTGAAGAACAGCCCGAACCCGAGGAACTACTACCGGTGCTCGGCGGAGGGCTGCGGCGTCAAGAAGCGCGTGGAGAGGGACCGCGACGACCCCCGCTACGTCGTCACCACCTACGACGGCGTCCACAACCACGCAACGCCCGGCGCCGCCGCGCAGTACTACTGCTACAGCCCCCCGCGCAGCTCGCCGCCGGCAGCGTATTCCGCCGCTGGCCTACTGCAATTCTGA

>Traes_1BL_46340D685 Org_Taestivumearly-release CDS: Traes_1BL_46340D685.1 (1 of 169) PF03106 - WRKY DNA -binding domain (WRKY) (PAC:31924766)

ATGGCGATGCGGCCCATGTCCGAGATGTCGCCGCCGCCGGCGCCGTCTGACCAGAGAGACGCTGTCATAGAGGAGCTCCGGAAGGGCTCTCAGCTTGCGGAGTTCCTCCGGCAGCAGGTGGAGCTCATCCCGGAGGACAGCCGCCGTGACGCCGCGCTGGCCAACGTGAGCGACATCACCACGGCCCTGGCGTCGTCACTCTCTGTGCTCCAGTCGGAGAGAGAGCAGTACTACTGCTCCTCCTCCTCCTCCGACGCCGGCCATGCTTCCGGGGCCTCCGGTGGTGGCGGGGTGCGAAACGGCGTAGTCGCACGCACCAGGAACAGGAAGGCGAAGCATCGGCGAGGCACCTATGGCGAGGAGCTCCCAATCAAGGAGATACTAACCAAGGCACCAGAAAATGATCGATTCCACTGGAGGAAATATGGGGAGAAGAAGATCCTCCATGCTGATTTTCCAAGGTTATACTACAGATGCGGATATAGCGACGAGCACAAGTGCCCGGCGAAGAAGTACGTGCAGCAGCAAAACAGCAGCGACCCGCCCATGTTCTTGGTCACCCTCATCAACGACCACACATGCGATACTTTGTTCCCAGATGAAGACCAAGACCAACCACCCAGCAGCTCAAGCAGCGCTAATAACTCGCAGATGCTCGACTTCTCCAAAGCGTCGCTCTCTTCGGCTGTTGGTGTCTCGAGATTGAAGGAGGAGGAAGACGCGGACATGTCCGTGACCGTGCCCAGCTACAACTACACGTATGATGAGCTGTCTTCTTCCTCGCTGCCGTTCCTGTCGCCCAAGCAGTGGGAGATGGAAATGGAGGTCAAGTCACTTTTTCGTCGTCACTCTGGGGATGGTAACTAG

>Traes_1BL_9AFA4B870 Org_Taestivumearly-release CDS: Traes_1BL_9AFA4B870.1 (1 of 169) PF03106 - WRKY DNA -binding domain (WRKY) (PAC:31740471)

ATGCTGGCGAGCGACGGTGCCGCCGGCGCGGTTGTTGTGCCCGACGGCGGGCCGGGCGGGACGGCGCACAGCGTGTCGGTGTCGTCCACGTCGAGCGAGGCCGGTGTCGGTGGTGGCGGCGCGGTGGAGGACGAGGCCGGGAAGTGCAAGAAGGAGGAGGGCGAGGGCGACGACGAGAGCAAGGAGGCGGCGGCCGATGGGGAGGCGGACAAGACCAAGAAAGGGGCGGCGAAGGGGAAAGGGGCCGCCAAGGCCAAGGGGGAGAAGCGGCCGCGGCAGGCGCGGTTCGCCTTCATGACCAAGAGCGAGGTTGACCATCTGGAGGACGGCTACCGCTGGCGCAAGTACGGCCAGAAGGCCGTCAAGAACAGCCCATTCCCAAGATGCAGGAGCTACTACCGGTGCACGACGCAGAAGTGCCCGGTGAAGAAGCGGGTGGAGCGGTCGTACCAGGACGCCGCCGTGGTGATCACCACCTACGAGGGCAAGCACACCCACCCCATCCCGGCCACGCTGCGCGGCGCCAACCACCTCCTCGCCGCGCACGCGCACGCGCACGGCGGCCACGGGCTCATCCACCCAGGCATGTTCCGGATGCCGGCGCCGCCCGGTGCCTTCCGTCCCGGCGACGCGCTCGGCAGCTTCCTGCAGCAGCAACACGCAGCCATGCAGCACCAGCAGCAAGTCGCCGCGGCGGGGATGGCGATGCGGCAGGCGAACGCCATGGCGAGCAGCCACATGCAGCAGGCGCCTCCTGCTGACCGTGGCCTGGCCGCCGCCATGGCCGGTGGTACGACGGGGAACAGTACCCATACTGTCAGCAGCAGTAGTGGTACTGATCCGCTGCGGATGGAGCACCTGATGGCGCAGGACTACGGCCTGCTGCAGGACATGCTCATGCCGCCGTCCTTCGCCCACAGCGACGGCGCCACCAACAGCAATAACAACGCCCACAACCGCCATTGA

>Traes_1DL_46428511F Org_Taestivumearly-release CDS: Traes_1DL_46428511F.1 (1 of 5) PTHR31221:SF23 - WRKY TRANSCRIPTION FACTOR 23-RELATED (PAC:31894510)

GACGGATCGGCCGATCATGGCAGCTGCAGGAGCAACGAGAAGGAGAAAAAGAAGAAGGGGAAGGGGGAGAAGAAGGCGCACGGCTCCCGTGTGGCCTTTGCAACCAAGAGCGAGGTTGACCACCTCGACGATGGCTACCGCTGGCGCAAGTACGGCCAGAAGGCCGTCAAGAACAGTTCCTTCCCAAGGAGCTACTACCGGTGCACGGCCACACGGTGTGGGGTGAAGAAGCTGGTGGAGCGGTCGCAGCAAGACCCTTCCACGGTCGTCACCACCTACGAGGGCCGCCACGGGCACCCAAGCCCCGTCGCCACCCACCGTGGCCCACGGATGCTAATGGCCACCGGTGCCAACACTGCCTACGCGCTTGCCGCGCTTCAACATCAGCAACATTGCTTTTTTTCAGCTGGTGCTGACGTCTACGCTCCGTTGGTGGCACACCGATTGTCGGAGCACGGTGGCATGCAGTTCCATGCCGATCTTCTTCCCGATGCCGTGATGGGCTACCAGCAAGGGTATCGTTAA

>Traes_1DL_5BAB0B6BC Org_Taestivumearly-release CDS: Traes_1DL_5BAB0B6BC.1 (1 of 5) PTHR31221:SF7 - WRKY TRANSCRIPTION FACTOR 24-RELATED (PAC:32010216)

ATGGTGAGCGGCGTGACTGGGGCGGCCGCGCCGGAGAGTGGTGCCGGGAGCAGTAGTGGCGTGGGAAGGGAGGAGACGAAAGGGAAGGGCAGTGCGCGCGCCAGGGGGAGCAGGAAGGCGAGCCGGCCGCGGTTCGCGTTCCAGACCAAGAGCGAGAACGACGTCCTCGACGACGGCTACCGGTGGAGGAAGTACGGCCAGAAGGCCGTCAAGAACAGCGCCTTCCCCAGGAGCTACTACCGGTGCACGCACCACACGTGCAACGTGAAGAAGCAGGTGCAGCGGCTGGCCAAGGACACGAGCATCGTGGTGACCACGTACGAGGGCGTCCACAACCACCCCTGCGAGAAGCTCATGGAGGCACTCAACCCCATCCTCAGGCAGCTTCAGTTCCTCTCGCAGCTCTAA

>Traes_1DL_DFE1721E0 Org_Taestivumearly-release CDS: Traes_1DL_DFE1721E0.1 (1 of 169) PF03106 - WRKY DNA -binding domain (WRKY) (PAC:31871499)

ATATTCCCCAATGGCGATGCGGCCCAAGTCCGAGATGTCGCCGCCGCCGGCGCCCCCGTCGCCCTCCGACCAAAGAGACGCTGTCATAGAGGAGCTCCGGAAGGGCTCTCAGCTTGCGGAGTTCCTCCGGCAGCAGGTGGAGCTCATCCCGGAGGACAGCCGNGCCGCCGTGACGCCGCGCTGGCCAACGTGAGCGACATCACCACGGCCCTGGCGTCGTCGCTCTCTGTGCTCCAGTCGGAGAAAGAGCAGTACTCTTCCTCCTCCTCCTCCTATGATCCCGGCCATGCTTCCGGCCCCTCAGGTGGCGGGATGCGAAATGGCCCCGTGGCACGCTCCAGGAACAGGAAGGCGAAGCACCGGCGAGGCACCTACGGCGAGGAGCTTCCAATCAAGGAGATACTAACCGAGGCACCAGAAAATGATCGATTCCACTGGAGGAAATATGGCGAGAAGAAGATCCTCCATGCTGATTTTCCAAGGTTGTACTACAGATGCGGATACAGCGACGAGCACAAGTGCCCGGCCAAGAAGTACGTGCAGCAGCAGAACAGCCGCGACCCGCCCATGTTCTTGGTCACCCTCATCAACGACCACACATGCGATACTTTGTTCCCAGATGAAGACCAAGACCAACCACCCAGCAGCTCAAGCAGCCCTAATAACTCGCAGGTGCTCGACTTCTCCAAAGCGTCGCTCTCTTCGGCTGTTGGTGTCTCGAGGTTGAAGAAGGAGGAAGACGCGGACATGTCCGTGACCGTGCCCAGCTACAACTACACGTATGATGAGCTGTCTTCTTCCTCGCTGCCGTTCCTGTCGCCCAAGCAGTGGGAGATGGAAATGGAGGTCAAGTCACTTTTTCGTCGTCACTCTGGGGATGGTAGCTAG

>Traes_2AL_15A7BB684 Org_Taestivumearly-release CDS: Traes_2AL_15A7BB684.1 (1 of 4) PTHR31282:SF15 - WRKY TRANSCRIPTION FACTOR 11-RELATED (PAC:31798439)

ATGGCCGTGGACCTCATGGGCTGCTACACCCCTCGCCGCGCCGACGACCAGCTCGCCATCCAGGAGGCCGCCACCGCCGGCCTCCGCAGCCTGGAGCTCCTCGTCTCCTCCCTCTCCGGCGCGGCGCCGTCCAAGGCGCCGCAGCAGCACCCGCAGCAGCCGTTCGGCGAGATCGCCGACCAGGCCGTCTCCAAGTTCCGCAAGGTGATCTCCATCCTCGACCGCACCGGCCACGCCCGCTTCCGCCGCGGCCCCGTCCAGTCGCCTACCCCGCCTCCTCCGGCTCCGGTCGCTCCTCCGCCGCCCCCACCGCGCCCTCTGGCCGTCGTCGAGCCGGCCAGGCCCGCTCCCTTGACCGTCGTGGCGCCGGTGTCGGTGGCCGCCCCGGTCCCTCTCCCGCAGCCGCAGAGCCTGACGCTGGACTTCACCAAGCCGAACCTGACCATGTCAGGCGCGACGTCCGTGACGTCCACGTCCTTCTTCTCGTCGGTGACCGCCGGCGAGGGCAGCGTGTCCAAGGGCCGCAGCCTGGTCTCCGCCGGCAAGCCGCCGCTGTCCGGGCACAAGAGAAAGCCGTGCGCCGGCGCGCACTCGGAGGCCAACACCACCGGCAGCCGATGCCACTGCTCCAAGAGAAGGAAGAACCGCGTGAAGACGACGGTGAGGGTGCCCGCGGTGAGCGCGAAGATCGCCGACATCCCGCCGGACGAGTACTCGTGGAGGAAGTACGGCCAGAAGCCCATCAAGGGATCCCCTTACCCACGGGGCTACTACAAGTGCAGCACAGTGCGAGGGTGCCCGGCGCGGAAGCACGTGGAGCGCGCCCTGGACGACCCGGCGATGCTGGTGGTGACGTACGAGGGCGAGCACCGCCACTCGCCGGGGCCGATGCCGATGCAGATGGCGCCGTCGCCGGTGCCGATTCCGATGCCGATGGGCGCGCCCGTAGCCGTAGCTAGTGTGTCCGCCGGCAACGGGCACGTCTGA

>Traes_2AL_409AB7647 Org_Taestivumearly-release CDS: Traes_2AL_409AB7647.1 (1 of 3) K18835 - WRKY transcription factor 2 (WRKY2) (PAC:31829399)

ATGGACGGCCACACCCACCTAGCCGTGGAGTGGAAGGACCAGAGCCCAGGGGCAGACTGCAGCATGCTGCCGAGCTTCCTCACCGACCCGTTCCCCGCGGACCCCCTGGTGGAGGACTGCGACGGCGGCAACGACGGCTCCGAGGGGGCCGGGTTCGAGAGGCACGGCCTGTCGGTGGCCGTGGGCTCGCCTCAGGAGGAAGGGAAGCCGGCGACGCCGCACTTCGGCCAGAGGTCCAGCTCCAGCAGCAGCCTCTCCGAGCGGATGCAGGCCAGAGCTGGGTTCAGCGTCGCCAAGCTCAGCATGCCTGGCAGCGAATATTCAGGAGCCCAGTCGCCTTACCTGACCATCCCGCCCGGCCTCAGCCCGGCGTCGCTGCTGGAATCGCCCGTGTTCCTCTCCAATGCCATGGGCCAGTCCTCGCCAACCACGGGGAAGTTACTCATGCTTGGTGACACCAATAACAACAATAATACTAGGCTTGAACCTCCCTCAATTGAAGATCGTCCCGGTGCATTTTCTTTCAAGCCTTTGGACCTAAAATCATCACAGTACACTGCTGAAGGAAAGAAGGGATCTCTACCCAACAGTCAGCACCCATCAGCACCAAGCAGAGATGTTCCTGTCAAGACAGAAACCAACATTCAGACAACGACACGAGGCGCCATTCCGCCAGGCCATCTGAACCAAGCGCAGTTCAACAACGGGCAAGACCTGATGAAGTGCAGCTACCATGACTGCAATAACAAGCGCAACCGTCTCGCAGCTGACAGGACCACGGCAGGTGGCGACAACAACGACGGCCCACCGGTAACGGCGGCGGACAGCGAGGCGGCGAAGGGAGACTACCCTGCTGCGGTGGCCACAGCAGCGCCGGCCGAGGACGGGTACAGCTGGAGGAAGTACGGGCAGAAGCAGGTGAAGCACAGCGAGTACCCGCGGAGCTACTACAAGTGCACGCACCCCAGCTGCCAGGTGAAGAAGAAGGTGGAGCGGTCGCACGAGGGCCACGTCACGGAGATCATCTACAAGGGCACGCACAACCACCCCAGGCCCGCCGCCCAGGGCCGCCGACCTGCCGGCGGCGCGCAGGTTCACCCGTTCAATGACGCGCAGATGGACGCGCCCGCGGACAACAACAACAACGGCTACGGCAATGCTGGTGGCTCGCAGCCGAACGCGGAGGCGAGGTCGCTGTGGCACGCGGGCGTGGCTGTCCAGGACTGGCGCGGCGACGGGCTGGAGGCCACGTCGTCCCCCTCGGTGCCCGGCGAGCTCTGTGACTCGTCGGCTTCGATGCAGGTCCATGACGGCGCCGCCCGGTTCGAGTCCCCGGAGGGCGGCGTGGATGTCACGTCTGCCGTGTCTGACGAGGTGGACGGGGATGATAGGGTGGCCCATGGCAGCATGTCCCAGGGGCAGGGCGCTGCCGACACCACCGAGGGCGATGAACTGGAGTCCAAGAGAAGGAAGCTGGAGTCGTGCGCCATTGACATGAGCACTGCGTCCAGAGCTGTCCGCGAGCCTCGGGTCGTGATCCAGACGACCAGCGAGGTGGACATCCTGGACGACGGCTATCGCTGGCGCAAGTACGGGCAGAAGGTCGTCAAGGGCAACCCGAATCCAAGGAGCTACTACAAGTGCACGCACCCGGGGTGCTCGGTGCGGAAGCACGTGGAGCGCGCGTCGCACGACCTCAAGTCCGTGATCACCACGTACGAGGGCAAGCACAACCACGAGGTCCCGGCCGCGAGGAACGGCGGCCACGGGAGCTCGGCGGCGTCGGGCGGCACGGGGGCGTCGCAGCTGTCCCACGCGCGCAGGGCGGAGCCGCCGTCGGTGCAGGACGGCCTGATGAGGCTGGGCGGCTGTGGCGCGCCCTTCGGCCTGCCGCCGAGGGACCCGCTGGGGCCGATGAGCAACTACCCGTACTCGCTCGGCGGCGGCCACGCGTNNNNCCTGCCGATGCCGTCGGGGCTCGGCGCCGTGGAGGGGCTGAAGCTCCCCATGCTGTCCCCGTCGCTGCACTCGGTGTTCAGGCAGAGGCAGGCCATGGAGACGGCGGCGGGATTCAGGGTGCCCAAGGGCGAGGTGAAGGACGAAGCCGCCGGGGCTGGGGCTGGCGCTGGGGGCGGCGCGGCGGCGGCGGCGGCGTATCCGCAGACGATGATGAGCAGGCTGCCGCTGGGGCACCGGATGTAG

>Traes_2AL_434E9F101 Org_Taestivumearly-release CDS: Traes_2AL_434E9F101.1 (1 of 16) PF03106//PF10533 - WRKY DNA -binding domain (WRKY) // Plant zinc cluster domain (Plant_zn_clust) (PAC:31854913)

ATGATGACCATGGATCTGATTGGAGGATACGGGAGGGCGGACGAGCAGGTGGCCATCCAGGAGGCGGCGGCGGCGGGGCTGTGCGGGATGGAGCACCTCATCCTGCAGCTCTCCCGGACAGGCACCAGCGAGAGCTCGCCGGTTGGGTCGTCGGAGGCGCCGGAGCAGCAGGTAGACTGCCGGGAGATCACTGATATGACCGTGTCCAAGTTCAAGAAGGTGATTTCTATCCTCAACCACCGCACTGGCCACGCCAGGTTCCGGCGCGGGCCTGTGGTGGCGCAGTCCCAGGGCCCCGCCGTGTCCGAGCCGGCGCCGGTGAGGGCGTCTTCGTCGAGGTCCATGACCTTGGACTTCACCAAGGCGTCTTCCGGGTACGGAAACGACGCCGGGTTCAGCGTCTCGGCCGCGAGCTCATCCTTCATGTCGTCGGTGACCGGTGACGGGAGCGTGTCCAACGGACGCGGGGGCGGGTCCTCGCTGATGCTCCCGCCGCCACCTTCGGCCAGCTGCGGGAAACCGCCGCTGGCGTCCTCCGCGGCATCCACCGGCGCCGGTGCCGGGCAGAAGCGCAAGTGCCACGACCACGCGCACTCAGAGAACGTCGCCGGCGGAAAGTACGGCGCCTCCGGTGGCCGCTGCCACTGCTCCAAGCGCAGGAAATCCCGGGTTCGGCGGATGACTCGCGTGCCGGCGATCAGCTCGAAGGCGGCGGAGATCCCCGCGGACGACTTCTCGTGGCGCAAGTATGGCCAGAAGCCTATCAAGGGCTCCCCCTACCCACGAGGTTACTACAAGTGCAGCACGGTGCGCGGGTGCCCGGCGCGGAAGCACGTGGAGCGTGACCCCAGCGACCCCTCCATGCTCATCGTGACCTACGAGGGCGAGCACCGGCACACCCCCGCGGACCAGGAGCCGCTCGCCCCGCTACCGGAGCTCTGA

>Traes_2AL_B1270662B Org_Taestivumearly-release CDS: Traes_2AL_B1270662B.1 (1 of 169) PF03106 - WRKY DNA -binding domain (WRKY) (PAC:31953214)

ATGGAAGGGGGTAGCCAGCTGGGGGCGTGCCTTCCCAGCCTCTACGCGCTCGATCCGTACGCATCCCCTCCCCTCCTCGCTCCATTGCCGAACCAGCACAAGCTTCACCAGATGCCGCTGGTGCTCCAAGAGCAGCCCGGGAACCACGGCGTGATGTTCTCCTCGGACCATGGCGGGGGCCTGTACCCGCTGCTTCCGGGGATCCCCTTCTGCCACTCCGCCGCCGCCTGCGAGAAGCCCACCGGGTTCGCGCCCTTGGGCGGCACCGGCGAGGCGGGCACATCGGCGGCCAGAGCGGCCAACGAGTTTGCTAGTACTACTACTACCACCACAGCCAGCTGCCATGGTCCGAGCTCATGGTGGAAGGGGGCGGAGAAGGGGAAGATGAAGGTGAGGAGGAAGATGAGGGAGCCGCGGTTCTGCTTCCAGACCAGGAGCGAAGTGGACGTGCTGGACGACGGATACAAGTGGAGGAAGTACGGACAGAAGGTTGTCAAGAACAGCCTTCATCCCAGGAGCTACTACCGGTGCACCCACAGCAACTGCCGCGTGAAGAAGCGTGTGGAGAGGCTGTCCGAGGACTGCCGCATGGTGATCACCACCTACGAGGGCCGCCACACCCACACCCCCTGCAGCGACGACGACGCCGCCGGCGACCACACCGGCAGCTGCGCCTTCACCTCCTTCTGA

>Traes_2AS_0186B9E4F Org_Taestivumearly-release CDS: Traes_2AS_0186B9E4F.2 (1 of 169) PF03106 - WRKY DNA -binding domain (WRKY) (PAC:31765715)

ATGGTGGCACCATCTTCTTCCATTTTGAAAGCGTCACGGAGCCTGATTGACAACTTTGATGTGTTCCATGAACAAGACCTAGCACAGCTGGCAGCACAAGTAGCTCAAAAGAAAGAGTTGCGGGAAAAACAAGGGGCGGGATTGCATCACAAGATTGGACCTCAACTAGCTTTTTCGAAATACAGTATACTTGATCAAGTGGACAACTCTTCTTCTTTCTCATTGGCAACTTCAGTGCTGACACCTCAGCATGTCAGTTCTTCCGTAGGCGCGGCATCAATGCAGGGACAGACTTTGCCATCACACACTGGTAGTGGTAGTGTCAACACTGGACCAACTGGAGTTTTACAAGTGCTTCAAGATTCATCCACCACTCTGGACAGTATCAACACTGGATCGGCTGGAGTTCTGGAAGCACTCCAAGGTTCATCCATCACTCTGGATAAACCTGCTGATGATGGATACAATTGGCGTAAGTATGGACAAAAGGCAGTCAAGGGTGGGAAGTATCCAAAGAGCTATTACAAATGCACCCTGAACTGCCCGGTCAGGAAAAATGTAGAGCACTCTGCAGATGGACGAATTATTAAAATAATTTATAGAGGTCAGCACTGCCATGAACCTCCCTCAAAGAGGTTTAAAGATTGTGGTGATTTATTGAATGAGTTAGATGAATTCAATGATGCCAAGGATCCTTCAACTAGATCACAATTAGGTTGTCAAGGTTATTATGGAAAACCTATAACGCCAAATGGCACGATGGTGGATGGTTTATTGCCAACGAAGGAAGAGGGAGATGAGCAATTATCTAGTTTAAGTGATATCCGGGAAGGTGATGGTGAAATAAGAACTGTTGATGGAGATGTTGGTGATGCCGATGCAAATGAAAGGAATGCACCAGGTCAAAAGATCATCGTGAGTACAACGAGCGATGTTGATCTTTTGGACGACGGCTATAGGTGGCGCAAGTATGGACAGAAAGTGGTGAGAGGAAATCCTCACCCAAGGAGCTATTACAAGTGCACTTACCAAGGCTGCGACGTCAAGAAGCATATCGAGAGATCTTCCGAGGAACCACATGCTGTGATAACCACATACGAAGGGAAGCATACCCATGACGTGCCTGAGTCTAGGAACAGAAGCCAAGGCACAGGTCAACACCACTGCAAAGAGCAGACTTATTCAGAACAACCAGCTGCTAGCTTCTGCAGTAGCTCGGAAAAGAGAAAATACGGAACAGCCATTCTGAACGATCTCGCCTTCTAG

>Traes_2AS_1AFFE8DA6 Org_Taestivumearly-release CDS: Traes_2AS_1AFFE8DA6.1 (1 of 169) PF03106 - WRKY DNA -binding domain (WRKY) (PAC:31960625)

GATGAGTACCGCGGGAGAAACAACGACAAGCGATCAAGATCACTTGTAACAGTTGTTCCACATTATGATGGCCATCATTGGAGAAAATATGGGCAGAAGAACATTAATGGGAGGGAACATGCTAGGCACTAC

>Traes_2AS_6269D889E Org_Taestivumearly-release CDS: Traes_2AS_6269D889E.1 (1 of 169) PF03106 - WRKY DNA -binding domain (WRKY) (PAC:31962353)

ATGGAGAGCGTGGAGGGAAACGGCGCCGGCCGCGGGAACCTGCAGCTTGTGGTGTCGGAGCTGTGCCGCGTCCAGGAGCTGGTGCGGCAGCTGGAGCTGCACCTACACGCGCCGGACGCCTCCATCGACCTGTGCCGCGCCCTCACCGCCGAGATCTTCGCGCTCACCGACCGGTCCATCGGCTTCGTCGCCGCCGCACATTTCCCCGACGCCCCGACGACGCCCTCCAGCACGTCCAGCTCCCTCAGCGGCGTCTCCGACCAGCCCTTCAGGACCAACACCAAGAAGAGGAAGGCGACGACGAGGTGGACGAGCCAGGTGAGGGTGAGTGCGGCCGGCGGCGCGGAGTGGCCCGGCGACGACGGCCACAGCTGGAGGAAGTACGGGCAGAAGGACATCCTCGGCGCCAGGCACCCGAGGGCCTACTACCGCTGCACCCACCGCAACTCGCAGGGCTGCCCCGCCACCAAGCAGGTGCAGCGCGCCGACCAGGACCCCGCGCTCTTCGACATCGTCTACCACGGCCAGCACACCTGCAGGCCAGGCGGCGGCGGCGGCGGGNNNNNNNNNNNNNNNNNNNNNNNNNNNNNACCACAACCCGCACGCGGAGAGCCTGCTGCAGAGCCTCAGAGCCGGCCTGACCGTGGACGCGGACCACGGCGCCCTGAACAACACCTCAGTCTCACCGCCGTCGATGGCCTCGCCTGTGGAGTCCGGCTCCAACGGTGGCCTGACCATGTCACCGTACCCGGTGCCGGCCGGCGCGTACACGGAGTGGCCGCTGGACGGCGACCTCCAGGAGGTGGTGTCGGCGCTCACAGCCGTGTCGGCCCCGAGCATGGACTGCCTTTTCGAGTTCGACCCGACCTTCGGCCTTGGTGCGCCCAACTTCTTCATGTGAGGACGACGAACAGAGCATCGAAGTGGAATTATATGACACTACCCAGCCCCTGCCTGTCCTTGGTAAGAGGGTAG

>Traes_2AS_C407071E4 Org_Taestivumearly-release CDS: Traes_2AS_C407071E4.2 (1 of 169) PF03106 - WRKY DNA -binding domain (WRKY) (PAC:31882682)

ATGCAATTTGCAGGCTGTAGTGTTGTTGTACTTCCTATTTTGCGTTCACAAGTATTCAGAGGTCTTAAAGCGTGGTGGACACTGAAAATAGTTAACAAAAATTTCTTTCAGGTTGAACCTTCACCAACAACTGGTAGTCTGAGCATGGCTGCAATTATGCACAAGAGTGCTCATCCAGACATGCCTTCGCCACGGGATAAGTCCATTCGAGCCCATGAAGATGGGGGTTCTAGGGATTTTGAATTCAAGCCTCATCTGAATTCGTCTTCTCAATCAGTGGCTCCTGCTATGAGTGATCTAAAAAAACACGAGCATTCTATGCAAAATCAGAGTATGAATCCCAGCTCATCATCTAGCAATATGGTGAATGAAAACAGACCTCCCTGTTCACGCGAGTCAAGTCTTACAGTGAATGTAAGTGCTCCGAACCAACCTGTTGGAATGGTTGGTTTGACTGACAGCATGCCTGCTGAAGTTGGTACTTCTGAGCCGCAGCAGATGAATAGTTCTGACAATGCCATGCAAGAGCCGCAGTCTGAAAATGTTGCTGACAAGTCAGCAGATGATGGCTACAACTGGCGCAAATATGGGCAGAAGCATGTCAAGGGAAGTGAAAACCCTAGAAGTTATTACAAGTGCACACATCCTAATTGTGAAGTAAAAAAGCTATTGGAGCGTGCGGTTGATGGTCTGATCACGGAAGTTGTCTATAAGGGGCGCCATAATCATCCTAAGCCCCAGCCTAATAGGAGGTTAGCTGGTGGTGCAGTTCCTTTGAACCAGGGTGAAGAACGATATGATGGTGCGGCAGCTGCTGATGATAAATCTTCCAATGCTCTTAGCAACCTTGCTAATGCGGTAAATTCGCCTGGCATGGTTGAGCCTGTTCCAGTTTCAGTTAGTGATGATGACATAGATGCTGGAGGTGGAAGATCCTACCCTGGGGATGATGGTACTGAGGAGGAGGATTTAGAGTCAAAACGCAGGAAAATGGAGTCTGCTGGTATTGATGCTGCTCTGATGGGTAAACCTAACCGTGAGCCCCGTGTTGTCGTTCAAACTGTAAGTGAGGTTGACATCTTGGATGATGGGTATCGTTGGCGGAAATATGGACAGAAAGTTGTCAAAGGAAACCCCAATCCACGGAGTTACTACAAATGCACAAGCACAGGATGCCCTGTGAGGAAGCATGTTGAGAGAGCATCGCATGATCCTAAATCAGTGATAACAACGTATGAAGGAAAACATAACCATGAAGTCCCTGCTGCGAGGAATGCAATCCATGAGATGTCCGCGCCTCCCATGAAGAATGTCGTGCATCAGATTAACAGCAATATGCCCAGCAGCATTGGCGGCATGATGCGAGCATGTGAAGTCAGGAACTTCAGCAACCAATATTCTCAAGCCGCTGAAACCGACAATGTCAGTCTTGACCTTGGTGTTGGGATCAGCCCGAACCACAGCGACGCCACAAACCAAATGCAGTCTACAGGTCCTGATCAGATGCAGTATCAAATGCAACCGATGGCTTCGATGTATAGCAACATGAGACATCCATCAATGGCAATGCCAACGGTACAAGGGAACTCTGCTGGCCGCATGTATGGTTCCAGAGAAGATAAAGGTAGCGAAGGGTTTACTTTCAGAGCCACACCGATGGACCATTCAGCTAACCTATGCTATAGCGGTGCTGGGAACTTGGTCATGGGTCCATGA

>Traes_2AS_D0C21ADB5 Org_Taestivumearly-release CDS: Traes_2AS_D0C21ADB5.1 (1 of 3) PTHR31221:SF25 - WRKY TRANSCRIPTION FACTOR 45-RELATED (PAC:31912163)

ATGGCAGGAGCGAGCAGCCATGACCATCTACATCACCATGGTCAAGCAGCTGGCAATAACACCGGCGGCGGCGGCGGCTTGGGCCAAGGCCTCTTCTCGGGGTCCAAGCAGGAGGATCCCTCCGAATCAAAGGATGGTGGCGATGATCGTGCAGGAAGTAGCTCACAGGGCGGTGGCGAGGCGGACGTGGTCGTCGGGAAGAAGAAAGGCGAGAAGAGGGAGCGGCGGCCGCGGTTCGCGTTCCAGACGCGCAGCCAGGTCGACATCCTCGACGACGGCTACCGGTGGAGGAAGTACGGGCAGAAGGCCGTCAAGAACAACAACTTCCCCAGGTCTGTAAATCCGTGCCCTAATTTCTCATATGACACCTTGACTTGCTGA

>Traes_2BL_6B75B32E3 Org_Taestivumearly-release CDS: Traes_2BL_6B75B32E3.1 (1 of 4) PTHR31282:SF15 - WRKY TRANSCRIPTION FACTOR 11-RELATED (PAC:31983429)

ATGGCCGTGGACCTCATGGGCTGCTACACCCCTCGCCGCGCCGACGACCAGCTCGCCATCCAGGAGGCCGCCACCGCCGGCCTCCGCAGCCTGGAGCTCCTGGTCTCCTCCCTCTCCGGCGCGGCGCCGTCCAAGGCGCCCCAGCAGCACCCGCAGCAGCCGTTCGGCGAGATCGCCGACCAGGCCGTCTCCAAGTTCCGCAAGGTGATCTCCATCCTCGACCGCACCGGTCACGCCCGCTTCCGCCGCGGCCCCGTCCAGTCGGCGCCGCCGCCGCCGCCTCCTCCAGCACCGGTCGCTCCACCTCCTCCCCCACCTTTGACCGTCGTGGCGCCGGTGTCGGTGGCCGCCCCGCTCCCGCAGCCGCAGAGCCTGACGCTGGACTTCACCAAGCCGAACCTGACCATGTCGGGCGCGACGTCCGTGACATCCACGTCCTTCTTCTCTTCGGTGACCGCCGGCGAGGGCAGCGTGTCCAAGGGCCGGAGCCTGGTCTCCGCCGGCAAGCCGCCGCTGTCCGGGCACAAGAGAAAGCCCTGCGCCGGCGCGCACTCCGAGGCCAACACCACCGGCAGCCGATGCCACTGCTCCAAGAGAAGGAAGAACCGCGTGAAGACGACGGTGAGGGTGCCGGCGGTGAGCGCGAAGATCGCCGACATCCCGCCGGACGAGTACTCGTGGAGGAAGTACGGCCAGAAGCCCATCAAGGGATCCCCTTACCCACGGGGCTACTACAAGTGCAGCACAGTGCGAGGGTGCCCTGCCCGGAAGCACGTGGAGCGCGCCCTGGACGACCCGGCGATGCTGGTGGTGACTTACGAGGGCGAGCACCGCCACTCGCCGGGGCCGATGCCGATGCAGATGGCGCCGTCGCCGATGCCAATGCCGATGGGCGCTCCCGTAGCCGTAGCTAGTGTGTCCGCCGGCAACGGGCACGTCTGA

>Traes_2BL_A5BFA97B9 Org_Taestivumearly-release CDS: Traes_2BL_A5BFA97B9.1 (1 of 169) PF03106 - WRKY DNA -binding domain (WRKY) (PAC:31892717)

ATGGAAGGGGGTAGCCAGCTGGGGGCGTGCCTTCCCAGCCTCTACGCGCTCGATCCGTACGCATCCCCTCCCCTCCTCGCTCCATCGCCGAACCAGCACAAGCTTCACCAGCTGCCGCCGGTGCTCCAAGAACAGCCCGGGGTCCACGGCGTGATGTTCTCCTCGGACCATGGCGGGGGCCTGTACCCGCTGCTTCCGGGGATCCCCTTCTGCCACTCCGCCGCCGCCTGCGAGAAGCACACCGGGTTCGCGCCCTTGGGCAGCACCGGCGAGGCTGGCACATTGGCGGCCAGACAAGGCAACGAGATTGCTAGTGCTACTACTACCACCACAGCCAGCTGCCATGGCCCGAGCTCATGGTGGAAGGGGGCAGAGAAGGGGAAGATGAAGGTAAGGAGGAAGATGAGGGAGCCGCGGTTCTGCTTCCAGACCAGGAGCGAAGTGGACGTGCTGGACGACGGATACAAGTGGAGGAAGTACGGCCAGAAGGTTGTCAAGAACAGCCTTCATCCCAGGAGCTACTACCGGTGCACCCACAGCAACTGCCGCGTGAAGAAGCGCGTGGAGCGGCTGTCGGAGGACTGCCGCATGGTGATCACCACCTACGAGGGCCGCCACACCCACACCCCCTGCAGCGACGACGACGTCGGCGGCGACCACACGGGCAGCTGCGCCTTCACCTCCTTCTGA

>Traes_2BS_380EC4D1E Org_Taestivumearly-release CDS: Traes_2BS_380EC4D1E.1 (1 of 169) PF03106 - WRKY DNA -binding domain (WRKY) (PAC:31924920)

ATGGCGGCGGGGCAGTGGTCAGGCATCGGCGACGGCGGCGGCCTCTGGGCCCCGCCCGCGCTCGACAGCCTCTTCCCCGACGACCAGCCGTCGCCGGCCGCCTCGGCGCTGGGCTTCTTCGGTGGATCCCTCGCGCAGCTCCCTTCCCCTCCGCCGCTCTGCGGCACCGCGCTCCTCGGGTACCCCCAGGACAACTTTGATGTGTTCCATGAACAAGACCTAGCACAGCTGGCAGCACAAGTAGCTCAAAAGAAAGAGTTGCGGGAAAAACAAGGGGCGGGATTGCATCACAAGATTGGACCTCAACTAGCTTTTTCTAAATACAGTATACTTGATCAAGTGGACAACTCTTCTTCTTTCTCATTGGCAACTTCAGTGCTGACACCTCAGCATGTCAGTTCTTCCGTAGGCGCGGCATCAATGCAGGGACAGACTTTGCCATCACACACTGGTAGTGGTAGTGTCAACACTGGACCAACTGGAGTTTTACAAGTGCTCCAAGATTCATCCACCACTCTGGACAGTATCAACACTGGATCAACTGGAGTTCTGGAAGCACTCCAAGGTTCATCCATCACTCTGGATAGACCTGCTGATGATGGATACAACTGGCGTAAGTATGGACAAAAGGCAGTCAAGGGTGGGAAGTATCCAAGGAGCTATTACAAATGTACCCTGAATTGCCCGGTCAGGAAAAATGTAGAGCACTCTGCAGATGGACGAATTATTAAAATAATTTATAGAGGTCAGCACTGCCATGAACCCCCCTCAAAGAGGTTTAAAGATTGTGGTGATTTATTGAATGAGTTAAATGATTTCAATGATGCCAAGGAGCCTTCAACTAAATCACAATTAGGTTGTCAAGGTTATTATGGAAAACCTATAACGCCAAATGGAATGATGACGGATGTTTTATTGCCAACGAAGGAAGAGGGGGATGAGCAATTATCTAGTTTAAGTGATATCCGGGAAGGTGATGGTGAAATAAGAACTGTTGATGGAGATGATGGTGATGCCGATGCAAATGAAAGGAATGCACCAGGTCAAAAGATTATCGTGAGTACAACGAGCGATGTTGATCTTTTGGACGACGGCTATAGGTGGCGCAAGTATGGACAGAAAGTGGTGAGAGGAAATCCTCACCCAAGGAGCTATTACAAGTGCACTTACCAAGGATGCGACGTCAAGAAGCATATCGAGAGATCTTCCGAGGAACCACATGCTGTGATAACTACATACGAAGGGAAGCATACGCATGACGTGCCTGAGTCTAGGAACAGAAGCCAAGCCACAGGTCAACACCACTGCAAAGAGCAGACTTATTCAGAACAATCAGCTGCAAGCTTCTGCAGTAGCTCGGAAAAGAGAAAATATGGAACAGCCATTCTGAACGATCTCGCCTTCTAG

>Traes_2BS_D435A8999 Org_Taestivumearly-release CDS: Traes_2BS_D435A8999.1 (1 of 169) PF03106 - WRKY DNA -binding domain (WRKY) (PAC:31876678)

ATGGCTGCAATTATGCACAAGAGTGCTCATCCAGACATACTGCCTTCGCCACGGGATAAGTCTATTCGAGCCCATGAAGATGGGGGTTCTAGGGATTTTGAATTCAAGCCTCATCTGAATTCGTCTTCTCAATCACTGGCTCCTGCTATGAGTGATCTAAAAAAACACGAGCATTCTATGCAAAATCAGAGTATGAATCCCAGCTCATCATCTAGCAATATGGTGAATGAAAACAGACCTCCCTGTTCACGCGAGTCAAGTCTTACAGTGAATGTAAGTGCTCCGAACCAACCTGTTGGAATGGTTGGTTTGACTGACAACATGCCTGCTGAAGTTGGTACATCTGAGCCGCAGCAGATGAATAGTTCTGACAATGCCATGCAAGAGCCGCAGTCTGAAAATGTTGCTGACAAGTCAGCAGATGATGGCTACAACTGGCGCAAATATGGGCAGAAGCATGTCAAGGGAAGTGAAAACCCTAGAAGTTATTACAAGTGCACACATCCTAATTGTGAAGTAAAAAAGCTATTGGAGCGTGCGGTTGATGGTCTGATCACGGAAGTTGTCTATAAGGGGCGCCATAATCATCCTAAGCCCCAGCCTAATAGGAGGTTAGCTGGTGGTGCAGTTCCTTCGAACCAGGGTGAAGAACGATATGATGGTGCGGCAGCTGCTGATGATAAATCTTCCAATGCTCTTAGCAACCTTGCTAATCCGGTAAATTCGCCTGGCATGGTTGAGCCTGTTCCAGTTTCAGTTAGTGATGATGACATAGATGCTGGAGGTGGAAGACCCTACCCTGGGGATGATGCTACAGAGGAGGATTTAGAGTCGAAACGCAGGAAAATGGAGTCTGCAGGTATTGATGCTGCTCTGATGGGTAAACCTAACCGTGAGCCCCGTGTTGTCGTTCAGACTGTAAGTGAGGTTGACATCTTGGATGATGGGTATCGTTGGCGGAAATATGGACAGAAAGTTGTCAAAGGAAACCCCAATCCACGGAGTTACTACAAATGCACAAGCACAGGATGCCCTGTGAGGAAGCATGTTGAGAGAGCATCGCACGATCCTAAATCAGTGATAACAACGTATGAAGGAAAACATAACCATGAAGTCCCTGCTGCGAGGAATGCAACCCATGAGATGTCCGCGCCTCCCATGAAGAATGTCGTGCATCAGATTAACAGCAGTATGCCCAGCAGCATTGGCGGCATGATGAGAGCATGTGAAGCCAGGAACTTCAGCAACCAATATTCTCAAGCCGCTGAAACCGACAATGTCAGTCTTGACCTTGGTGTTGGGATCAGCCCGAACCACAGCGATGCCACAAACCAAATGCAGTCTTCAGGTCCTGATCAGATGCAGTACCAGATGCAATCCATGGCTTCGATGTACGGCAACATGAGACATCCATCATCAATGGCAGTGCCAACGGTACAAGGAAACTCTGCTGGCCGCATGTATGGTTCCAGAGAAGAGAAAGGTAACGAAGGGTTTACTTTCAGAGCCACACCGATGGACCATTCAGCTAACCTATGCTATAGCGGTGCTGGGAACTTGGTCATGGGTCCATGA

>Traes_2BS_F3097F116 Org_Taestivumearly-release CDS: Traes_2BS_F3097F116.1 (1 of 3) PTHR31221:SF25 - WRKY TRANSCRIPTION FACTOR 45-RELATED (PAC:31818595)

GAGAATTACCCCATTTTCTTTGGGACGCAGCCCTCATCTTCCACCTCCAATCCATACCACTTCGTGGCAGGAGCGAGCAGCCATGACCATCTCCATCACCATGGTCAAGCAGCTGGCAATAACACCGGCGGCGGCGGCTTGAATCAAGGCCTCTTCCTGGGGTCCAAGCAGGAGGAGCCCTCTGAATCAAAGGATGGTGGCGATGATGGTGCAGGAAGTAGTTCACAGGGCGGTGGCGGGGAGGCGGACGTTGTCGTCGGGAAGAAGAAAGGCGAGAAGAGGGAGCGGCGGCCGCGGTTCGCGTTCCAGACGCGCAGCCAGGTCGACATCCTCGACGACGGCTACCGGTGGAGGAAGTACGGGCAGAAGGCCGTCAAGAACAACAACTTCCCCAGGAGCTACTACCGGTGCACGCACCAAGGATGCAACGTGAAGAAGCAGGTGCAACGGCTATCACGGGACGAAGGTGTGGTGGTAACGACATACGAAGGCACCCACACGCACCCAATCGAGAAGTCCAATGACAACTTCGAGCACATACTCACCCAGATGCAGGTCTACTCAGGCATCAACAACGTCTCCCAAACCTTTGGCAACCAACACATGTTTCAATGA

>Traes_2DL_04535D371 Org_Taestivumearly-release CDS: Traes_2DL_04535D371.1 (1 of 6) PTHR31221:SF8 - WRKY TRANSCRIPTION FACTOR 57-RELATED (PAC:31903007)

ATGGCCGGTGTCGAGTGCGGCGGTGGGGACTGGCCCTTCTCCGCCGAGGAAGCGTACGCCGATTCCTCTGCGCTGTTGGCGGAGATCGGCTGGGCGGCCGGTTTTGTCGACGACGGCTGCGCCGGGGAGCTGCTTCCGCCGCTGGATCCGCCTCCGGCCACACCAACGGGGTCCATGGAAGGGGCCGGCGCCTCGTCGAGCTCCACCGATGACGGTGCCACGCGGGAGGCTGCAGACGCCGACGGCAGGCCGGCCGCCGCGACAGAGGCAGCGAGCAAGCCGGCGCCGGCGCTGGCCCCGGGGAAGACGATGAAAAAGCAGAAGCGGGCGCGGCAGCCACGGTTCGCGTTCATGACCAAGACGGAGATAGACCACCTCGAGGACGGATACCGCTGGAGGAAGTACGGACAGAAGGCCGTCAAGAACAGTCCTTTCCCAAGGAGCTACTACCGGTGCACCAACAACAAGTGCACGGTGAAGAAGCGCGTGGAGCGCTGCTCCGACGACCCCTCCGTTGTCATCACCACCTACGAGGGCCAGCACTGTCACCACACCGTCACCTTCCCCCGCGGCGCCGGCGCCGCCACCCTCGCCAGCCAGATGGCCTTCTCGGCACACCACCACCACCTCATGTACAACGACTTGCCGGCGCTGCACTCGCCGACCACTCAAAACCCACTCTTCAGCGTGCCGGCGATGTCGTCGTCGCTGCTCCAGCCGCTACACTGCAACCGACAGGAGCTGCAACTTGCAAGCTACACAACCCAGGCATCGTCCATCTCGTCGCCAGGGAGTGTTCCCGCCGTCGATAAGGGGCTTCTGGATGACATGGTGCCTCCAGCGATGAGGCACGGATAG

>Traes_2DL_362A1F535 Org_Taestivumearly-release CDS: Traes_2DL_362A1F535.1 (1 of 169) PF03106 - WRKY DNA -binding domain (WRKY) (PAC:31942345)

AATATCTTCTTTCTTGGGCGTTGCAGGACCCAGGATTCGTCCGTCGTGACGAAGAACATGAAGAGCTTGGAGGACGGGCAGACCTGGCGCAAGTACGGGCAGAAGGAGATACAGAACTCCAAGCACTCAAAGGCCTACTTCCGGTGCACGCACAAGTACGACCAGCAGTGCATGGCGCGGCGGCAGGCCCAGCGCTGCGACGACGACCCG>Traes_2DL_4F9F8F1F0 Org_Taestivumearly-release CDS: Traes_2DL_4F9F8F1F0.1 (1 of 169) PF03106 - WRKY DNA -binding domain (WRKY) (PAC:31853252)

ATGGAAGGGGGTAGCCAGCTGGGGGCGTGCCTTCCCAGCCTCTACGCGCTCGATCCGTACGCATCCCCTCCCCTCCTCGCTCCATTGCCGAACCAGCACAAGCTTCACCAGCTGCCGCTGGTGCTCCAAGAGCAGCCAGGGAACCACGGCGTGATGTTCTCCTCGGACCATGGCGGAGGCCTGTACCCGCTGCTTCCGGGGATCCCCTTCTGCCACTCCGCCGCCGCCTGCGAGAAGTCCACCGGGTTCGCGCCCTTGGGCGGCACCGGCGAGGCGGGCACATCGGCGGCCAGAGCGGGCAACGAGTTTGCTAGTGCTACTACTACCACCACAGCCAGCTGCCATGGTCCGAGCTCATGGTGGAAGGGGGCGGAGAAGGGAAAGATGAAGGTGAGGAGGAAGATGAGGGAGCCGCGGTTCTGCTTCCAGACCAGGAGCGAAGTGGACGTGCTGGACGACGGATACAAGTGGAGGAAGTACGGCCAGAAGGTTGTCAAGAACAGCCTTCATCCCAGGAGCTACTACCGGTGCACCCACAGCAACTGCCGCGTGAAGAAGCGTGTGGAGCGGCTGTCGGAGGACTGCCGCATGGTGATCACCACCTACGAAGGCCGCCACACCCACACCCCCTGCAGCGACGACGACGCCGGCGGCGACCACACGGGCAGCTGCGCCTTCACTTCCTTCTGA

>Traes_2DL_F600B5FDF Org_Taestivumearly-release CDS: Traes_2DL_F600B5FDF.1 (1 of 4) PTHR31282:SF15 - WRKY TRANSCRIPTION FACTOR 11-RELATED (PAC:31752041)

TGCCACTGCTCCAAGAGAAGGAAGAACCGCGTGAAGACGACGGTGAGGGTGCCGGCGGTGAGCGCGAAGATCGCCGACATCCCGCCGGACGAGTACTCGTGGAGGAAGTACGGCCAGAAGCCCATCAAAGGATCCCCTTACCCACGGGGCTACTACAAGTGCAGCACAGTGCGGGGGTGCCCGGCGCGGAAGCACGTGGAGCGCGCCCTGGACGACCCGGCGATGCTGGTGGTGACCTACGAGGGCGAGCACCGCCACTCGCCGGGGCCGATGCCGATGCAGATGGCGCCGTCGCCTATGCCGATGCCGATGGGCGCTCCCGTCGCCGTAGCTAGTGTGTCCGCCGGCAACGGGCACGTCTGA

>Traes_2DS_97E3E7CFC Org_Taestivumearly-release CDS: Traes_2DS_97E3E7CFC.1 (1 of 169) PF03106 - WRKY DNA -binding domain (WRKY) (PAC:32007262)

ATGGCTGCAATCATGCACAAGAGTGCTCATCCAGACATACTGCCTTCGCCACGGGATAAGTCTATTCGAGCCCATGAAGATGGGGGTTCTAGGGATTTTGAATTCAAGCCTCATCTGAATTCGTCTTCTCAATCACTGGCTCCTGCTATGAGTGATCTAAAAAAACACGAGCATTCTATGCAAAATCAGAGTATGAATCCCAGCTCATCATCTAGCAATATGGTGAATGAAAACAGACCTCCCTGTTCACGCGAGTCAAGTCTTACAGTGAATGTAAGTGCTCCGAACCAACCTGTTGGAATGGTTGGTTTGACTGACAACATGCCTGCTGAAGTTGGTACATCTGAGCCGCAGCAGATGAATAGTTCTGACAATGCCATGCAAGAGCCGCAGTCTGAAAATGTTGCTGACAAGTCAGCAGATGATGGCTACAACTGGCGCAAATATGGGCAGAAGCATGTCAAGGGAAGTGAAAACCCTAGAAGTTATTACAAGTGCACACATCCTAATTGTGAAGTAAAAAAGCTATTGGAGCGTGCGGTTGATGGTCTGATCACGGAAGTTGTCTATAAGGGGCGCCATAATCATCCTAAGCCCCAGCCTAATAGGAGGTTAGCTGGTGGTGCAGTTCCTTCGAACCAGGGTGAAGAACGATATGATGGTGCGGCAGCTGCTGATGATAAATCTTCCAATGCTCTTAGCAACCTTGCTAATCCGGTAAATTCGCCTGGCATGGTTGAGCCTGTTCCAGTTTCAGTTAGTGATGATGACATAGATGCTGGAGGTGGAAGACCCTACCCTGGGGATGATGCTACAGAGGAGGAGGATTTAGAGTTGAAACGCAGGAAAATGGAGTCTGCAGGTATTGATGCTGCTCTGATGGGTAAACCTAACCGTGAGCCCCGTGTTGTCGTTCAAACTGTAAGTGAGGTTGACATCTTGGATGATGGGTATCGTTGGCGGAAATATGGACAGAAAGTTGTCAAAGGAAACCCCAATCCACGGAGTTACTACAAATGCACAAGCACAGGATGCCCTGTGAGGAAGCATGTTGAGAGAGCATCGCATGATCCTAAATCAGTGATAACAACGTATGAAGGAAAACATAACCATGAAGTCCCTGCTGCGAGGAATGCAACCCATGAGATGTCTGCACCTCCCATGAAGAATGTCGTGCATCAGATCAACAGCAATATGCCCAGCAGCATTGGGGGCATGATGAGAGCATGTGAAGCCAGGAACTTCACCAACCAATATTCTCAAGCGGCTGAAACCGACACCGTCAGTCTTGACCTTGGTGTTGGGATCAGCCCGAACCACAGCGACGCCACAAACCAAATGCAGTCTTCAGGTCCTGATCAGATGCAGTATCAGATGCAATCCATGGCTTCGATGTACGGCAACATGAGACATCCATCATCAATGGCAGTGCCAACGGTACAAGGAAACTCTGCTGGCCGCATGTATGGTTCCAGAGAAGAGAAAGGTAACGAAGGGTTTACTTTCAGAGCCACACCGATGGACCATTCAGCTAACCTATGCTATAGCGGTGCTGGGAACTTGGTCATGGGTCCATGA

>Traes_2DS_AD8820C42 Org_Taestivumearly-release CDS: Traes_2DS_AD8820C42.1 (1 of 169) PF03106 - WRKY DNA -binding domain (WRKY) (PAC:31872073)

ATGGCTCGTCTCCCCGCCTCTCACCACCAGTCCACCACCTCCTCTCCTGCACCTCGCGATCGAGAGATATCGCTGATCACTTGCTCGCTTCCTTCCTTCTCCTCCGCCAATCGAACGTGTTCTTCCGTGTCCGCGGCAATGGAGAGCGTGGAGGGGAACGGCACCGGCCGCGGGAACCTGCAGCTTGTGGTGTCGGAACTGTGCCGAGTCCAGGAGCTGGTCCGGCAGCTGGAGCTGCACCTACACGCACCGGACGCCTCCATCGACCTGTGCCGCGCCCTCACCGCCGAGATCTTCGCGCTCACCGACCGGTCCATCGGCTTCGTCGCCGCCGCACATTTCCCCGACGCCCCGACCACGCCCTCCAGCACCTCCAGCTCCCTCAGCGGCGTCTCCGACCAGCCCTTCAGGACCAACACCAAGAAGAGGAAGGCGACGGCGAGGTGGACGAGCCAGGTGAGGGTGAGTGCGGCCGGCGGCGCGGAGGGGCCTGGCGACGACGGCCACAGCTGGAGGAAGTACGGGCAGAAGGACATCCTCGGCGCCAAGCACCCGANCCTACTACCGCTGCACCCACCGCAACTCGCAGGCCTGCCCCGCCACCAAGCAGGTGCAGCGCGCCGACCAGGACCCCGCGCTCTTCGACGTCGTCTACCACGGCCAGCACACCTGCAGGCCAACGGGCGGTAG

>Traes_2DS_F6FBC974C Org_Taestivumearly-release CDS: Traes_2DS_F6FBC974C.2 (1 of 169) PF03106 - WRKY DNA -binding domain (WRKY) (PAC:31836810)

ATGGCGGCGGGGCAGTGGTCAGGCATCGGCGACGGCGGCGGCCTCTGGGCCCCGCCCGCGCTCTACAGCCTCTTCCCCGACGAGCAGCCGGCGCTCGGCTTCTACGGGGGATCCCTCGCGCAGCTCCCTTCCCCTCCGCCGCTCCTCGGGTACCCCCAGGACAACTTTGATGTGTTCCATGAACAAGACCTAGCACAGCTGGCAGCACAAGTAGCTCAAAAGAAAGAGTTGCGGGGAAAACAAGGGGCGGGATTGCATCACAAGATTGGACCTCAACTAGCTTTTTCGAAATACAGTATACTTGATCAAGTGGACAACTCTTCTTCTTTCTCATTGGCAACTTCAGTGCTGACACCTCAGCATGTCAGTTCTTCCGTAGGCGCGGCATCAATGCAGGGACAGACTTTGCCATCACACACTGGTAGTGGTAGTGTCAACACTGGACCAACTGGAGTTTTACAAGTGCTCCAAGATTCATCCACCACTCTGGACAGTATCAACACTGGATCAGCTGGAGTTCTGGAAGCACTCCAAGGTTCATCCATCACTCTGGATAGACCTGCTGATGATGGATACAATTGGCGTAAGTATGGACAAAAGGCAGTCAAGGGTGGGAAGTATCCAAAGAGCTATTACAAATGCACCCTGAACTGCCCGGTCAGGAAAAATGTAGAGCACTCTGCAGATGGACGAATTATTAAAATAATTTATAGAGGTCAGCACTGCCATGAACCTCCCTCGAAGAGGTTTAAAGATTGTGGTGATTTATTGAATGAGTTAGATGATTTCAATGATGCCAAGGAGCCTTCAACTAGATCACAATTAGGTTGTCAAGGTTATTATGGAAAACCTATAACGCCAAATGGCACGATGGTGGATGGTTTATTGCCAACGAAGGAAGAGGGAGATGAACAATTATCTAGTTTAAGTGATATCCGGGAAGGTGATTGTGAAATAAGAATTGTTGATGCAGATGTTGGTGATGCTGATGCAAATGAAAGGAATGTACCAGGTCAAAAGATTATCGTGAGTACAACGAGCGACGTTGACCTTTTGGACGACGGCTATAGGTGGCGCAAGTATGGACAGAAAGTGGTGAGAGGAAATCCTCACCCAAGGTAA

>Traes_3AL_140B829CB Org_Taestivumearly-release CDS: Traes_3AL_140B829CB.2 (1 of 1) PTHR31221:SF37 - WRKY TRANSCRIPTION FACTOR 71-RELATED (PAC:31942939)

ATGTCTTCCGGTGGTGGCGGGGGAGGGGATCAGGGCCGTCATGGCCTCTACCACCAGCATGGCCACGGCCAACTCACCCGCTACGATGGCGCCGGCGGCTACGAGCTCAGCAACGACGACATGGAGAGCTTCTTCTTCAGCCAGCCTGAGGGCGTCGGCGGTGGCGTGCGCGCCGACGAGATCGCGCCGTACTCGAGCATCACGAGCTACCTGCAGGGGTTCTTGGACCCCACCGGGCTAGCTCGGCATCTCGACGTGCCGGCCAAGCACGAGCTGTCGGTCGACGTTAGGAGCCATGACCAAGACAGCCAGGGCACCGGCAGCGCTGCTGGAGAAAGCGCGGCGCTGCTAACACCCAACTCATCGGTATCTTTCTCGTCCGGAGGCGGGGACGGTGAGGGAAAGTCTCGCCGGAGCAAGAAGGGCCGGGCGCAGGAGCCGGATGACCAGGAGGATGGGAAGGACCATGAAGATGGGGAAAGTTCCAAGACAGCGAATAACAAACCCAAAAAGAAAGCCGAGAAGAGGCCGCGGCTGCCCCGCGTCTCCTTCCTCACCAAGAGCGAGGTCGATCACCTCGAGGACGGCTACCGCTGGCGCAAATACGGCCAGAAGGCCGTCAAGAACAGCCCTTATCCAAGGAGCTACTACCGGTGCACTACGCCCAAGTGCGGCGTGAAGAAGCGGGTGGAGCGGTCGTACCAGGACCCGTCGACGGTGATCACCACGTACGAAGGGCAGCACACGCACCACAGCCCCGCCAGCCTCCGGGGAAGCGCCGCGCACCTCTTTATGCCCCCCGGACTCCACGGGCTCCCGCCGCCGCACCTCATACCGCCGGGGGTGTTCCACCCAGAGCTGATGAGCATGATGCGCATGCCCTACCCAAGCCCTAACATGCACCTGCTGAGCGTGCCACCGCCTCCCCATCATCATCCAACCTCTCATCCAATGGCGGGAACTCTCCAGCAGTACCATTTCACTGACTACGCGCTATTGCAAGACCTCTCCACTTCCACAATGCCCAACAACCCCTGA

>Traes_3AL_1B73D2C12 Org_Taestivumearly-release CDS: Traes_3AL_1B73D2C12.1 (1 of 169) PF03106 - WRKY DNA -binding domain (WRKY) (PAC:32002429)

CCTAAGATTTTATTTAATCTAAATTTCATTTATTATCACTGCAGGAACTACTACCGGTGTTCAACGGAGGGGTGCAGCGTGAAGAAGAGGGTAGAGAGAGACAAAGACGACGCAAACTATGTAGTGACGATGTATGAGGGGGTTCACAACCATGCGAGCCCTGGCACAATTTACTATGCTGCTCAAGATCCTGCTTCAGGCCGCTTCTTTGTTACTGGGACGCATCAACTAGCTCCTTGA

>Traes_3AL_2297D6E18 Org_Taestivumearly-release CDS: Traes_3AL_2297D6E18.1 (1 of 169) PF03106 - WRKY DNA -binding domain (WRKY) (PAC:31939858)

ATGGCGTCACCGCCTCCGAAGGGGGAGTCGTTTGACTTCGAGGATCCACGCGCGCAGGAAGCCATGGGTTCTGCGTCCGCGTCCTACAGCGCTCCCGGGGGCGTCTTCGGCCTCTCCCCGCCGGAGTCCTCGCGCCGCGATAGCCGAAAGAGAAGGAAAGACAGACCTTCATGGGTCAAACATACGTTCACACCTCATTTTGACGGTCACCTGTGGAGAAAGTATGGCCAGAAAAACATCAAGGACTCTGTCTTCCCAAGGCTTTATTACAGATGCTCTTACCGTGAAGACAAGCAATGCCTTGCCTCAAAGCTGGTGCAACAGGAGAACCACGAGGACCCACCACTGTTCAAGGTCACCTACACGTACGAGCACACGTGCAACACCGCGCCCGTCCCAACTCCAGATGTCGTGGCCGAGCTGCCGGCGCCGGCAACTGGCGACGCACTATTTCTGAGGTTCGATTCCACCGGCGCAGGCCACCGGGACGCGCACCGGATGGAGCAGGAACGGCATTACCAGCAGCCTGCGGCACCTGGGTGGCCGTCCATGATGCTGAGCTTTGATTCCAATAGCCAGCAGCACGAACAGTGTACGTTCCCTTCCGAGCTGCCGCCAGCTGCGTCGTCGTCGTCGTTTTCGACCGAGGGGCTGCCAGCGCCGCCGTCTACAACCGATGGCGGAGGCGACGGGTTCTCGACGTGGGACTCGTTGAGATACGGATTAAATGACCATGTGCACTTCGGCGACAATTCGTATCTCCCAAACAGTGGTAATGATGGTGACGATAACTACTGA

>Traes_3AL_3160E1F30 Org_Taestivumearly-release CDS: Traes_3AL_3160E1F30.1 (1 of 169) PF03106 - WRKY DNA -binding domain (WRKY) (PAC:31939797)

ATGGACGGGTACAGGTGGAGGAAGTACGGGCAGAAGTTCATCAAGAACAACCCTCATCCCAGGAGCTACTACAAGTGCACCAGCGCACGGTGCAGCGCCAAGAAGCACGTCGAGAAGTCCACCGACGACCCGGAGATGCTCATCGTCACCTACGAGGGGTCGCACCTCCACGGCCCGCAGACGACG

>Traes_3AL_4769A72F1 Org_Taestivumearly-release CDS: Traes_3AL_4769A72F1.1 (1 of 5) PTHR31221:SF7 - WRKY TRANSCRIPTION FACTOR 24-RELATED (PAC:31977027)

AGGAGCTACTACCGGTGCACCCATCCCACATGCAACGTGAAGAAGCAGGTGCAGCGCCTGGCCAAGGACACGGCCATCGTGGTGACCACGTACGAGGGTGTGCACAACCACCCCTGCGAGAAGCTCATGGAGGCGCTCGGCCCCATCCTGAAGCAGCTCCAGTTCCTCTCC

>Traes_3AL_67ECA2932 Org_Taestivumearly-release CDS: Traes_3AL_67ECA2932.1 (1 of 169) PF03106 - WRKY DNA -binding domain (WRKY) (PAC:31827594)

GCGGCGAGCGGCAAGATCGCGTTCCGGACTAGGTCGGAGGAGGAGATACTGGAAGACGGCTACAAGTGGAGGAAGTACGGCAAGAAGTCTGTCAAGAACAGCCCTAACCCAAGGTATCCAAAACGAACGAATTCATTCACTTTTCTATAG

>Traes_3AL_AB2BAE660 Org_Taestivumearly-release CDS: Traes_3AL_AB2BAE660.1 (1 of 169) PF03106 - WRKY DNA -binding domain (WRKY) (PAC:31851405)

ATGTCCTCCTACTCGTCCCTCCTCTCCGTGAGCCCCGGCGAGCAGATCGGTGGGTATGCGGACGGAGGTGACCACGACGACATGGCGGCCGCCGCCAACTACCTATCATCATTCTGCTTTGATTTCGGCGAGGAGTATTATTCCCTGGCGGAGGCAGCAACAGCCTCCTACCCCTTGCACGCACAGCAGCAGCAGCAGCCGCCAACCCAGGCCGACAGCCACCACAGCGGCAAAGCAGCAAGTACTACTAGTAGTTCACAAGGACTCGACAATATCAACACGAGCTTGACCAGCAGCGACGCGAGGAGCAAAGGCAGCAAGATCGCGTTCAAGACGAGGTCGGAGGTGGAGGTGCTGGACGACGGGTACCGCTGGAGGAAGTACGGCAAGAAGATGGTCAAGAACAGCCCAAACCCAAGGAACTACTACCGTTGCTCCAGCGAGGGGTGCCGCGTGAAGAAGCGG

>Traes_3AL_DED8A29EC Org_Taestivumearly-release CDS: Traes_3AL_DED8A29EC.1 (1 of 5) PTHR31221:SF7 - WRKY TRANSCRIPTION FACTOR 24-RELATED (PAC:31876237)

AGCGGGGAGAACGACGGCGAGGCGGGTGGGAGCGGAAGTGGTAACAAGGAGAAAGCGAAGGGAAGCGCCGGGAGATCGGGGAAGAAGAAGGCGAGCAAGCCGCGGTTCGCGTTCCAGACGAGGAGCGAGAACGATATCCTGGACGACGGCTACCGCTGGAGGAAGTACGGGCAGAAGGCCGTCAAGAACAGCTCCAACCCTAGGTATGGTTCGTCCAATTAA

>Traes_3B_0C5417706 Org_Taestivumearly-release CDS: Traes_3B_0C5417706.2 (1 of 169) PF03106 - WRKY DNA -binding domain (WRKY) (PAC:31922736)

ATGCAGACGCAGTCCCGTCTCATCATGAACCCCAACGGCGGCGTAACCGGCTACGAGCCCGCGGCGACCGACGAGCAGCACGAGGCGGTGCTGAGGGAGCTGGCGCACGGGCACGAGCTGACGGCGCACCTGCAGGCGGAGGCGCTGCGGGCGCTGCACGGGCAGGGCCAGACCGAGGCCACCGCCGCATTAATCCTGCAGGAGGTGTCCCGCGCCTTCACCGTCTGCATCAACATCATGGGCGGCTCCGCCCCGGCCGCGACCCCGACCACGCCGCCACCGGACGCGGCGGCCGTCGTCGTCACCGGCGCCGCGAGCGCGCGCCGTCCTAGAGACGACGGCGTCCCGAGAAAAGTAACAGTTACTTCCTCGCCCTACTCGGACGGGTACCAGTGGAGGAAATACGGCCAGAAGAGGATCATGAGGACAAGCTTCCCAAGGTGCTACTACCGATGCTGCTACCATCGCGAGCGCAGCTGCCCGGCCACGAAGCTCGTGCAGCAGCAGCCGCCGCAGCAGCACAGCGACGGCGACCAGACGATGTACACCGTCACCTACGTCCACGAGCACACGTGCCATAACATGGCGCCGGCCGAGCCCGAGGCGGCGGCACGTAGCTCCACGCCCGACCCGCTCGGCTTCTCTGCCGGGATGCAGCCGAGGCAGCAGCAGCGGGGCGGCGCCGGCCTGGACCGCGGCTCCAAGGAGGAGCTCGAGCGGCAGGCGCTCGTGTCGTCCCTCGCCTGCGTGCTGCAGGGCCACCACCAGAGCTACACCGGGTCCGGGGCCGGCACGCCGGACGGATCGCCCTCGCAGGGCCGCGTCGGCGACGGCCCGTCTGCGTCAGGTCTTTCGCTCGATACGTCTGACGACTTGGGGTTGGACGTCATGGACTACGGCGTGACGGACGCGCTGTATTTCGCTGCGTCCTCATCGTATGGCCCAGGCGGCGACGGCATGATTCCTTGA

>Traes_3B_41047D5E6 Org_Taestivumearly-release CDS: Traes_3B_41047D5E6.2 (1 of 169) PF03106 - WRKY DNA -binding domain (WRKY) (PAC:31782323)

ATGGCGGTAGCTGGAGCCGGGGCAGCGTATCGTTTCCACCCGCACGGCGCCGGCTCGATGGCGTTTCCCCGGCCGCCGGGATCCGGCTGCCCGTACAGCTCCGGAGCCCCGTTGAGCTCTCCCGCCTTCGGTGGGGCCACCGGGCCCGGGGTGCTTCAGCAGCAGCTGGACGTCCTGGACTACTTGTCCGACGACGGCGGGGTGCCCGGCACGGTCGGCGCGCCGCTGCCGGTTGAGGCGGCGGTCGTGCCGGATGTTGGCTACTGTGATCATACGAGGGCGGCCGCGGTGGCGGCGAGCGGCAAGATCGCGTTCCGGACGAGGTCGGAGGAGGAGATACTGGACGATGGCTACAAGTGGAGGAAGTACGGCAAGAAGTCTGTCAAGAACAGCCCTAACCCAAGGAACTACTACCGGTGTTCAACGGAGGGGTGCAGCGTGAAGAAGAGGGTAGAGAGAGACAAAGACGATGCAAACTATGTAGTGACGATGTATGAGGGGGTTCACAACCATGCGAGCCCTGGCACAGTTTACTATGCTTCTCAAGATCCTGCTTCGGGTCGCTTCTTTGTTACTGGGACCCATCACCTAGCTCCTTGA

>Traes_3B_8B0D448D8 Org_Taestivumearly-release CDS: Traes_3B_8B0D448D8.1 (1 of 169) PF03106 - WRKY DNA -binding domain (WRKY) (PAC:31890613)

ATGAGGGGGAGCAACATGCTCAGCTCCAGTGGGAGCAACAAAAGGGCGCTGCAGCAGGATTGCAGTGGCGGCAGCCATGCCCAGGAGCACACCAAGAGGAAGTCTCGCATTGGCATGAGAACAGACTACACATATGCACCGTATCATGATGGATTCCAGTGGAGAAAATATGGGCAGAAGGTGATCCGGGGCAATGCCTTCCCAAGGTGCTACTACAGGTGCACGTACCACCAGGATCATGGCTGTTCGGCGAGCAAGCACGTGGAGCAGCACAACTCGGCGGACCCGCCGCTGTTCCGCGTGGTCTACACGAACGATCACACATGCAGCGGCGCTGCTGCTTCCGCATCGGACTACATGGCCTCATCGATGCAAATCCAGCAGATCGCTGACGCCTCTCTGAGAAAGGCCGACACGGAAGCGGAAAGGCCGCCGCGCCCGCAGCAGCCTCGCTCCGGCGGTAGCTATGCCGCGGCGATAAAAGAGGAGAAAGACGCCATCGTCTCCTCCCTGCTCACCGTCATCAGAGGCAGCTGTGACGTTGTGAAATCTGACACTGCGCACGAGGGCTACAGCAGTGCGTCGCTGGCTACTAACTGCTACGCGATGTCATCCCCGTCGGTGGCCGGAGGTAGCCGTGAGGGTAGTAGCAGCTCTTCGGTTTCGCCAGTGGTGCTGCCGGCGCCAGATGACATGGGATTGGGACTGGACTTCATGGTGGAGTCCCACTGGTTCGAGCCTTTGGATTTGGGTTGGTTCGTAGAATAG

>Traes_3B_990298FF5 Org_Taestivumearly-release CDS: Traes_3B_990298FF5.1 (1 of 1) PTHR31221:SF1 - WRKY TRANSCRIPTION FACTOR 1-RELATED (PAC:31865868)

ATGACAACCTCGTCGTCAGGGAGCGTGGAGACGTCGGCCAATTCGAGGCCCGGCTCCTTCTCCTTCGGGAGCGCGAGCTTCACTGAAATGCTGGGCGGCTCCGCCGCGGCCGGCGGCGCGTCCGGGTACAAGGCCCTGACCCCGCCTTCCCTGCCGCTCTCGCCTTCGCTCATGTCGCCGTCGTCCTTCTTCAGCATGCCCGCCGGCATGAACCTCGCCGACTTCCTCGACTCCCCGGTGCTCCTCACCTCCAGTATCTTCCCGTCACCCACGACGGGCGCGTTCGGGTCCCAGTTCAACTGGCGGCCGGAGGCGCCGACGCCGAGCGCCGCCGAGCAGGGCGGCAAGGAGGAGCAGAGGCAGCCCTACTCCGACTTCTCGTTCCAGACGGCGCCAGCGAACAGCGAGGAGGCCGCGCGTGCGACGATGACGACTAGCTTGCAGCCACCGGTTGCTGTGGCGTCACAGGGGGAGGAAGCGTACACAGGCCAGCAGCAGCAGGCATGGGGGTACGGCCAGCAACAAGAAGGCATGAACGCGAGTGCCGCCAACCCGGCGAGCTTCAGCGCGCCGGCGCTCCAGGCGACCTCGTCGGAGATGGCGCCTGCGGGCGCGTACAGGCAGACGCACTCGCAGAGGCGGTCGTCGGACGACGGGTACAACTGGCGCAAGTACGGGCAGAAGCAGGTGAAGGGGAGCGAGAACCCCCGCAGCTACTACAAGTGCACCTTCCCCAACTGCCCCACCAAGAAGAAGGTGGAGACGTCGCTGGAGGGCCAGATCACCGAGATCGTGTACAAGGGCACGCACAACCACGCCAAGCCGCTCAACACGCGCAGGGGCTCCGGCGGCGGCGCGGCGGCGGCCCAGGTGCTGCAGATCGGCGGCGACGCGTCTGAGAATTCCTTCGGAGGGATGGTCACCACGCCCGAGAACTCGTCGGCGTCCTTCGGGGACGACGACAACGGTGTAAGCTCGCCGCGCTCTGGCAACGTCGGCCGTAACGACAACGACGATGATGAGCCGGATTCCAAGAGGAGGAGAGACAGTGGTGACGGCGAGGGAATAAACATGGCCGGCAACCGTACGGTGCGGGAGCCGAGGGTGGTCGTCCAGACCATGAGCGACATCGACATCCTCGACGACGGCTACCGGTGGAGGAAGTACGGGCAGAAGGTGGTGAAGGGCAACCCGAACCCGCGGAGCTACTACAAGTGCACCACGGTGGGGTGCCCCGTGCGGAAGCACGTGGAGCGCGCGTCCCACGACCTGCGCGCCGTGATCACCACGTACGAGGGCAAGCACAACCACGACGTGCCCGCCGCCAGGGGAAGCGCCGCGCTCTACCGCCCCGCGCCCCGGGCGGCGGACAGCACCGCGAGCACCGGCCACTACCTTAACCCGCAGCCGTCCGCCATGGCGTACCAAGCCAGCGCCGCCCCCAACGTGGCCGGGACACAGCAGTACGCGCCGAGGCCCGACGGGTTCGGCGGCCAGAACCCGGGCTCGTTCGGCTTCAACGGCAACTTCGGCTTCTCCGGGGCCGGTTTTGACAACCCGACGGCGTCGTACATGAGCCAGCACCAGGAGCAGCAGAGGCAGAACGACGCGATGCACGCATCGAGCGCCAAGGAGGAGCCGAGGGAGGAGGACATGTTCTTCCAGAACTCCCAGTACTGA

>Traes_3B_B8BF316B8 Org_Taestivumearly-release CDS: Traes_3B_B8BF316B8.2 (1 of 2) PTHR31221:SF42 - WRKY TRANSCRIPTION FACTOR 49-RELATED (PAC:31892659)

ATGGAGGAGATGGGGGAGGAGAGCAGCAGGTATCCATGGCAGGACTACGACCTGGGCTTCGGGGAGGAGCTCATGAGGGAGCTCCTCGACCAGACGACGACGGCGCCAACGCCATCGCCAGCAGCAATGGCGGCCGGCGCCGCGAGCGCTGATAACTCTTCTTCTTCCGATAAGGGGATCGGTGACGAGGAGGAAGGGGCGGCGGGGCGCCGGGAGTCCATGGAAAACAGGCTCATGTCGACGGTCTACTCCGGGCCCACCCTCAGCGACATCGAGAGCGCCCTCTCCTTCACCGGCGCCGGCGCCGGCGACCCGCTGGACGGCCGCAGCAAGTACCACTACAGCCCCTCCAGCCCAGTGTACGTCCCTCGTATCTATGCCAACAACAACCATATACAATTCTCCGTAGATGCAAGCTCAATTTGGACATCTGTGTGGAAATTCAGGGTTTTCTCGCCGGAGAAGGTGCTGGGCAAGATGGAGAACAAGTACACGATGAAGATCAAGAGCTGCGGCAACGGGCTCGCCGACGATGGGTACAAGTGGAGGAAATACGGCCAGAAAGCCATCAAGAACAGCCCCAACCCAAGGAGTTACTACCGTTGCACGAACCCGCGGTGCAACGCGAAGAAGCAGGTGGAGCGCGCCGTCGACGAGCCGGACACGCTCGTCGTCACCTACGAAGGCCTCCACCTCCACTACACCTACTCCCACTTCCTCCAGCAGCAGACCAACCCTCCACCCGCCGCCGCCGCCGCCGCCTCAAGCTCCAAGAAGCCCAAGCTGCACCCCACCGCCGGCGCCATTACAGTTACAGACTCCCACCACGGGAGCACCCCTGTG

>Traes_3B_CDA5ADD75 Org_Taestivumearly-release CDS: Traes_3B_CDA5ADD75.1 (1 of 169) PF03106 - WRKY DNA -binding domain (WRKY) (PAC:31744736)

ATGTCAGATCTGGGCCTCCCAGCCAGCTCGCCCTTTTTTATACGCGCCCCGTCCTACCGGGGAGAGACTTCAGACTCCTCCCTCTTTCGGTCTCTACGACACGTTTCTTTCTCTCTCCCTCTCCCCTCGGCTACCAAGGCGTCTCTCTCTTCTCCAAGGAGGCTCTCGATTTGCATGGACAAGGGCCACCTCGGTGGTGGCGGAGGAGGAGGAGGACTGCTAGCCCTGGACGCCTCGCCGCGGCAGCTCGGCTTCTTGAACTTGCTATCGCCGGCGCCGTTCCATAGGAGCATGGAGGCGGACGACGGCGGAGGCGGTGGCGGCGGCGGCAGGGGGAGGAGGTCCATCGAGGTTGACTTCTTCTCCGACGAGAAGAAGAACATGAAGAAGAGCCGGGCTTCGGCCGGGGCCGACGCGGAGGACCACAAGGATCAAGCCTCCGCCGCCGGACTCGCCATCAAGAAGGAGGACCTCACCATTAACCTCCTTCCCGGGAACAACACGAGGAGCGACCGGTCCATGGTGGTCGACGACGACGGAGCGTCCCGGGCCGACCAAGACAGGAACGGCAGGAACACCGGCGAGCTGGCGGTGATACAGGCCGAGCTGAGCCGCATGAACGAGGAGAACCAGCGGCTGCGAGGGATGTTGACCCAGGTCAACAACAGCTACCATGCGCTGCAGATGCATCTCGTCGCGCTCATGCAGCAAAGGACCCAGATGCCTCCGGTCCAGCCGCAACAACCTCCGACCCACGAGGATGGCAAGAACGAGAGTGCCATCGTGCCGAGGCAGTTCCTGGGTCTGGGCCCGTCGGGAGCCAGCGCTGACGTGGCGGAAGAGCCCTCCAACTCGTCCACGGAGGTCGGGAGCCCGCGGCGGTCGTCGTCCAACGGAAACGAGGACCCTGAGCGCGGCGAAAACCCTGACGGCCCGTCCACCGCGGGGTGGTTGCCCGGACGCGGGATGAGCCAGCAGCAGCAGCAGCAGCTGGGGGCGGCGGCGAAGGGCCACGACCAGCAAGCGCAGGAGGCCACCATGAGGAAGGCCCGTGTCTCCGTTCGCGCCCGATCCGAAGCCCCGATTATCGCCGATGGATGCCAATGGAGGAAGTACGGTCAGAAGATGGCCAAGGGAAACCCTTGCCCACGCGCCTACTACCGGTGCACCATGGCCACCGGCTGCCCGGTGCGCAAGCAGGTGCAACGATGCGCCGAGGACCGGACCATCCTCATCACCACGTACGAGGGCACGCACAACCACCCGCTCCCGCCGGCCGCCATGGCCATGGCGTCTACCACGTCGGCCGCTGCGTCCATGCTGCTCTCCGGCTCCATGCCCAGCGCCGACGGCGCGGGGCTCATGAGCTCCAACTTCCTCGCGCGCACCGTGCTTCCGTGCTCGTCCAGCATGGCCACCATCTCGGCGTCCGCGCCGTTCCCGACGGTCACGCTCGACCTCACCCACGCCCCGCCCGGGGCGCCCAACGCCATGCCGCTAAACGTCGCCCGACCGCATGCACCGGGGCAGTTCCATGTTCCGATGCCCGGCGGTGGGATGGCCCCGGCCTTCGCGATGCCGCCGCACATGCTGTACAACCAGTCCAAGTTCTCTGGGCTGCAGATGTCCTCCGACTCGGTGGACGCCGGGCAGTTC

>Traes_3B_D6F86ABC3 Org_Taestivumearly-release CDS: Traes_3B_D6F86ABC3.2 (1 of 169) PF03106 - WRKY DNA -binding domain (WRKY) (PAC:31895081)

ATGGCGCTGGCCACCCCGACCGCCGTGGTGCTGGAGCTGATGACCATGGGGCAGCAGTCCGCGGCGCACCTCGGGGACCTGCTCAGGGCGGCGTCCCCGCCGGTGCGGGCGGAGCACCAGGCCCTCGCCGCCGAGATCCTCCGGTGCTGCGACCGGGTGATCGCCGCGGTGAGCGCGGGCGCCTCCGATAAAAAGAGGAAGATGACGGACCCTGGCGCCACGACCTGCCATCCTCCCGCGGCCGCGATGCCATCCAAAAGAAGGGTGCGTGGCGCGGAGGCGCACAGAGAGGTCCATGCCGACACGACGGCGGACGGGTTCGTGTGGAGGAAGTACGGGCAAAAGGACATCAACGGAAGCAACCACCCGAGGCTCTACTACCGCTGCGCGTTCAGAGGCGAGGGCTGCGCTGCGACCCGGCGGGTGCAGCGGTCGCAGGAGGAGCCCGCGGCGTTCGTGATCGCCTACTACGGCGAGCACACCTGCGGAGCCGCCTTCTCTCAGCAGAGGGCGGAGCCACAGCCTCCTACTGTCGTCGACTCCGGCTCAAACGCTTGGGGAGTCTTCGGTGCCGTCGACCGGAACAGGGGCTCGCCTCTGATGCCGTCGCTCGCAGCCGAGCACGATGTGCGGCGTCACGGCGAGGCGCCCCGTGACACGTCGCAGCGATGGTCGTCGCCGTCGTCCTCCTCGTCCTACTCCGAGGTGGAACTTGGTGCTTCTCCTGTCGAGGGGTTCCTCGATGGCAACTTCGACTGGGAGTGGGAGACCGTCGTCAACTCGCTCAGATTCGGCGATCTGCTTCACTAG

>Traes_3B_F45FCFE62 Org_Taestivumearly-release CDS: Traes_3B_F45FCFE62.1 (1 of 169) PF03106 - WRKY DNA -binding domain (WRKY) (PAC:31799212)

ATGGAGGAGAGGTGCGCCCTGGCCACGGAGCTGGCGCAGGTGCTCGACACGGTGAGGCAGCTGGAGGCGCACATGGGCGTGAAGGGCGGCGCTGACGGAGGGGAGACGTGCCGGACGCTAGTGTCGAGCATGCGCTCCTCCGTCGACAGGTCCATCCACATCGCCATGTCGTCGTGCTGCGTGGTCCTCGGAGCGCCGGAGTCGCCGCCGTCTGCCGGCGGGNGGAGCCCCCGCAGCGGCGGCTCTGACCAGGCTGCCGACTCCCCTTGCCGCGGCGCTCATGCAGCTGGGCAGTCCAAGAAGAGGAAAACACAGCCCAAGTGGAGCACGCAGGTGAGGGTGAACTCCGTGGAGGACGTCGGCCCCCTCGACGACGGCATCAGCTGGAGGAAGTACGGTCAGAAGGACATCCTCGGAGCCAAGTACCCAAGGGCCTACTTCCGGTGCACCCACCGGCACACGCAGGGCTGCTACGCCAGCAAGCAGGTGCAACGTGCTCACGGCGACCCGCTGCTCTTCGACGTCGTGTACCACGGGAACCACACGTGCGCGCAGGGCAAGCACTACAACAGCCAGAGGCCACAGCCCGTAGCAAGCGGAGAGCACCGNAGGAGCGGATCTCCGTGGGACTGA

>Traes_3DL_2551BF2C1 Org_Taestivumearly-release CDS: Traes_3DL_2551BF2C1.1 (1 of 169) PF03106 - WRKY DNA -binding domain (WRKY) (PAC:31968771)

TTGTGTGAGGTCCATTTTTCCTTCAACAGAGTAACAGTGACTTCCTCGCCGTACTCCGACGGGTACCAATGGAGGAAATACGGCCAGAAGAGGATCATGAGGACAAGCTTCCCAAGGTGCTACTACCGATGCTGCTACCATCGCGAGCGCAGCTGC

>Traes_3DL_678D51EAD Org_Taestivumearly-release CDS: Traes_3DL_678D51EAD.1 (1 of 169) PF03106 - WRKY DNA -binding domain (WRKY) (PAC:31874088)

CATGCAGCTGGGCAGTCCAAGAAGAGGAAAACACAGCCCAAGTGGAGCACACAGGTGAGGGTGAATTCCGTGGAGGACGTCGGCCCCCTCGACGACGGCTTCAGCTGGAGGAAGTATGGTCAGAAGGACATCCTCGGTGCCAAGTACCCAAGGGCCTACTTCCGGTGCACCCACCGCCACACGCAGGGCTGCTACGCCAGCAAG

>Traes_3DL_7456F61A3 Org_Taestivumearly-release CDS: Traes_3DL_7456F61A3.1 (1 of 169) PF03106 - WRKY DNA -binding domain (WRKY) (PAC:31793891)

ATGAGAACAGACTACACATATGCACCGTATCATGATGGATTCCAGTGGAGAAAATATGGGCAGAAGGTGATCCGGGGCAATGCCTTCCCAAGGTGCTACTACAGGTGCACGTACCACCAGGATCATGGCTGTTCGGCGAGCAAGCACGTGGAGCAGCAC

>Traes_3DL_DF0D3F3FE Org_Taestivumearly-release CDS: Traes_3DL_DF0D3F3FE.1 (1 of 169) PF03106 - WRKY DNA -binding domain (WRKY) (PAC:31886335)

GAGGAGGAGATACTGGACGATGGCTACAAGTGGAGGAAGTACGGCAAGAAGTCTGTCAAGAACAGCCCTAACCCAAGGAACTACTACCGGTGTTCAACGGAGGGGTGCTACGTGAAGAAGAGGGTAGAGAGAGATAAAGACGACGCAAACTATGTAGTGACGATGTATGAGGGGGTTCACAACCATGCGAGCCCTGGCACAGTTTACTATGCTGCTCAAGATCCTGCTTCGGGGCGCTTCTTTGTTACTGGGACGCATCACCTAGCTCCTTGA

>Traes_4AL_234E1CDF6 Org_Taestivumearly-release CDS: Traes_4AL_234E1CDF6.1 (1 of 2) PTHR31221:SF16 - WRKY TRANSCRIPTION FACTOR 62-RELATED (PAC:31959259)

ATGCATGCCCCTATTTTCTTAGTCGAATTAAAGTGCTCGTTCATCATTTATGTGATCGATGAGTTATATTTGTATGTATTGGTTTGCAGAGGGAGCGGAGAAGGTGAGCGCGGTCATCATGGCGACGAGGAGGAGCAGCAGCAGGCAGCTTGGGCGGAGGAGGCAGCCGGCGTTCAACCGCTGGTGATGCCGGAGGACGGGTACCAGTGGAAGAAGTACGGCCAGAAGTTCATCAAGAACATCCAGAAAATCAGGAGCTACTTCCGGTGCCGCGACAAGCGGTGCGGCGCCAAGAAGAAGGTGGAGTGGCAGCCGGGCGACCCCAGCCTTCGCATCGTCTACGACGGCGCGCACCAGCACGGCTCCCCAGCCTCAAACGGCGGTGGTCAGGACGGCGACGGCGCCGCCAACCGGTACGATCTCAGCACCCAGTACTTCGGCGGGGCCGGCGCCCCCACGCCGCAGACGCGGTGA

>Traes_4AL_2EEECCC4B Org_Taestivumearly-release CDS: Traes_4AL_2EEECCC4B.1 (1 of 169) PF03106 - WRKY DNA -binding domain (WRKY) (PAC:31746115)

ATGGCAAATTTTGAAATGTCCCATCAGCAAGCGTTAGCCCAAGTAACAGCACAAGCAGTCCATTCTCAGTATACTGTAGGCAGTCAAGCAGATTACTCACTCCCTTTCTCATCGGCAACAACATCAGCTTTGACATCACAGTTCATCAACTCTTCTGCAAATGTGACATCAATGAAGGAGACAGCAACTCTGCCACTGCATACAGTTAATGATAACCTTAAGTCAAATGAGGTTTCACAAGGATTCCAAACTTCGGCCCTCACCGTGGATAAACCTGCTGATGATGGGTACAATTGGCGAAAGTATGGCCAGAAGGCAGTTAAGGGTGGAGAATATCCAAGGAGCTACTATAAATGCACCCAGGCGAGCTGTCCAGTTAAGAAGAAAGTGGAGCACTCAGCATATGGACAGATTACTCAAATAATTTATAGAGGCCAGCATAACCACCAGCGTCCACCAAAAAGGAGGTCCAAAGATGGTGGCAATTTACTAAATGAAGATGATTTCCCTGAGAACAGAGACGCTTTGACTCGATCAGAACCGGGCTCTCAAGATCATTCTGGAAAAGTTGAGGTATCAAATGATGGCATCACAGGGCCTTCAGTGTCTAAGAGGAGAGGCGGAGGTGATCAATCGTCTGGCTCAAGTGATACAGAGGAGGACAATGATGAAGCAGGTGATGACAATGGAGATGCTGGTATTGTGAATGCAAACAAAAGGCATGTGCCGGCCCCAGCTCAAAGAATCATCGTGCAAACAACCAGTGAAATTGATCTTTTGGATGATGGCTACCGGTGGCGCAAGTACGGGCAGAAAGTGGTAAAAGGGAATCCTCATCCAAGGAGTTACTACAAATGCACGTACCAGGGATGCGATGTAAAGAAGCATATCGAAAGATGTTCGCAAGACCCAACGGCGGTGATAACTACATATGAAGGCAAGCATAGCCATGATGTGCCAGCAGCTAGGAGCAGCGTGGCTGCTGCTGCCAGTGCCAATGCATCCTCTTCCATTAGCTTACTACATAGAGGGCAGAAGGCAGCATCCAGTAGCCAACGGGTGTTGCCCAGAGCAGCACTACATACATCAGATTCTTCCTTGCAGCTAAAAGAAGAAAATGAGATAACATAA

>Traes_4AL_98B1C762B Org_Taestivumearly-release CDS: Traes_4AL_98B1C762B.2 (1 of 16) PF03106//PF10533 - WRKY DNA -binding domain (WRKY) // Plant zinc cluster domain (Plant_zn_clust) (PAC:31891223)

ATGGAGGAGGTGGAGGAGGCCAACAGGATGGCCGTGGAGAGCTGCCACAGAGTGCTCGGCCTGCTCGCCCAGACCCAGGACCCGGCGCAGCTCAGGAGCATAGCGCTGGGGACGGACGAAGCCTGTGCCAAGTTCAGGAAGGTGGTCTCCCTCCTCGGCAACGGCAACGGCAACGGCAACGAAGGAGGGGGAACCCACCATCCTAGAGCCAAGCTGGTGAGCAGAAGGCAGACCCCGGGGTTCTTGAGCCAGAAGAGCTTCCTGGACAACAACACCCCGGTGGTGGTGCTGAACAGCGCGCACCCTTCGACCAGCTCGGCTCAGGTGTATCCTAGCAGCAGAAACAGCATTCTGGATTCATCACAGGCCGCGCACCCGATCGGAGGGCCTCCCAAGCTGGTGCAGCCGTTGTCCGCGCACTTCCAGTTCGGCGACTCATCGCGGTACAACCAGTTCCAGCAGCAGCATCAGCACCAGCAGCAGAAGATGCGGGCCGAGATGTTCAAGAGAAGCAACAGTGGGATCAACCTCAAGTTTGACAGCCCCAGTGGCACCGGGACGATGTCGTCCGCGAGGTCCTTCATGTCATCTTTGAGCATGGACGGCAGTGTGGCCAGCCTGGATGCCAAGTCTTCCTCCTTCCATCTGATCGGTGGGCCTGCTATGAGCGACCCGGTGAACGCGCAGCAGGCGCCGAGGAGGCGGTGCTCGGGACGTGGGGAGGATGGAAATGGCAAGTGTGCTGCAACTGGCAGGTGCCATTGTTCTAAGAGAAGCAGGAAGTTGCGGGTGAAGAGGACGATTAAAGTTCCCGCAATTAGTAATAAAATTGCTGATATACCTCCAGACGAATACTCCTGGAGGAAGTATGGGCAGAAGCCAATTAAGGGTTCCCCTCATCCCAGGGGGTACTACAAATGCAGCAGTGTGAGGGGTTGCCCTGCGCGGAAGCATGTTGAGCGTTGCGTGGATGATCCATCGATGCTCATTGTGACATACGAGGGTGAACATAACCATACACGAATGCCAACTCAGTCTGCGCAAGCTTAG

>Traes_4AL_C2A825B6D Org_Taestivumearly-release CDS: Traes_4AL_C2A825B6D.1 (1 of 169) PF03106 - WRKY DNA -binding domain (WRKY) (PAC:31792629)

ATGGCACAGGTCTCTTTCGCCGGCGCCGGGGATGACAAACATCGCAGCGAGAAGACGATCAAAATCAGTGCGAGGGTGAGCGCAGGGAGGATCGGGTTCAGGACGAGGTCAGAGGTGGAAATCTTGGACGACGGGTTCAAGTGGAGGAAGTACGGGAAGAAGGCGGTGAAGAACAGCCCGAACCCGAGGAACTACTACCGGTGCTCGGCGGAGGGCTGCGGCATCAAGAAGCGCGTGGAGAGG

>Traes_4AS_0DA136E0E Org_Taestivumearly-release CDS: Traes_4AS_0DA136E0E.1 (1 of 169) PF03106 - WRKY DNA -binding domain (WRKY) (PAC:32002393)

ATGGCGGAAGATGGGCAGAAAGAAATCCAAAACTCAACTCATCCGAGGAGCTATTACAGGTGTACGCACAAATCAGACCAAGGCTGCAACGCCAAGAGGCAAGCCCAGATCTGCGAGACCCATCCAATCAAGTACGACATCACGTACTACGGCGAGCACACCTGCAAGCCTCCCTCCAATACCCCAATGATCATTGTCGCTGCGAGCGACGACCGTGCAGAAAACCTCGTCAGTTTTGCCCCGACGTTTCCTCATTTGGCCCCGGGCAGTGCACCAGCGTTGACGACGTGTTCAGCTCCTCGTCCGATCCATTCTTGCAGGCGGACGAGCTCGCCGCCTTCGTGGGATCGGCCGGGAGGACGTCCTCGATGGTGGGGTCGGTGCCGGACCACAGCGGGAGTGGGATAG

>Traes_4AS_70DF607CC Org_Taestivumearly-release CDS: Traes_4AS_70DF607CC.1 (1 of 169) PF03106 - WRKY DNA -binding domain (WRKY) (PAC:31888413)

ATGTCTCCCGTGCCGAGCCCAAATCAATCACACCTTCTAGGCCATGGATCAAGGAAAGAGAAGCGCATGAGGAAGGTGGATACCTTTGCGCCGCACAACGACGGCCACCAATGGAGGAAGTACGGCGAGAAGAAGATCAACAACTGCAACTTTCCCAGGTACTACTACAGATGCACCTACAAAGACAACATGAACTGCCCGGCCACCAAGCAGATTCAGCAGAAAGATCACAGCGACCCCCCATTGTACCAAGTCACATACTACAACGAGCACTCATGCAACAGCGCCTTCCTTGCCCTCACTCCCACAGAGTTCCAGCTGCAGACCGCATCTGGAAAGGCAGTCTCCATCTGCTTTGATTCATCCGGGGCTCAGGAGCCGGGAGCCAATGCGAGCTCGCCTTCTTCGAGTGCGGCGCCACGCGCCACGCCTTCGGAGAGCAAGAACAAGCCCCTTGCGCTGCGTTCAGAGGCGCTTTCTTCCTGGGCCCCCGGCGTTGTGGAGCAAAAGACGGCCTGTGCTGATCTCCAGTCCTGCAGCACCGAGTGCCAAGATGCATACATTTCAGAAGACATAGATGCAGGGAGATTCGGTTCTATCAGATTCTTCCATTTTTTGTAA

>Traes_4BL_EFEC50B26 Org_Taestivumearly-release CDS: Traes_4BL_EFEC50B26.2 (1 of 169) PF03106 - WRKY DNA -binding domain (WRKY) (PAC:31827464)

ATGTCCCATCAGCAAGCGTTAGCCCAAGTAACAGCACAAGCAGTCCATTCTCAGTATACTGTTGGCAGTCAAGCAGATTACTCACTCCCTTTCTCATCGGCAACATCAGCTTTGACATCACAGTTCATCAAATCTTCTGCAAATGTGACATCAATGAAGGAGACAGCAACTCTGCCACTGCATACAGTTAATGATAACCTTAAGTCAAATGAGGTTTCACAAGGATTCCAAACTTTGGCCCTCACCGTGGATAAACCTGCTGATGATGGGTACAATTGGCGAAAGTATGGCCAGAAGGCAGTTCAGGGTGGAGAGTATCCAAGGAGCTACTATAAATGCACCCATGCGAGCTGTCCAGTTAAGAAGAAAGTGGAGCACTCAGCATATGGACAGATTACTCAAATAATTTATAGAGGCCAGCATAACCACCAGCGTCCACCAAAAAGGAGGTCCAAAGATGGTGGCAATTTACTAAATGAAGATGATTTCCCTGAGAACAGAGACGCGTTGACTCGATCAGAACCAGGCTCTCAAGATCATTCTGGAAAAGTTGAGGTATCAAATGATGGCATCACAGGGCTTTCAATGTCTAAGAGGAGAGACGGAGGTGATCAATCGTCTGGCTCAAGTGATAGAGAGGAGGACAATGATGAAGCAGGTGATGATAATGGAGATGCTGGTATTGTGAATGCAAACAAAAGGCATGTGCCGGTGCCAGCTCAAAGAATCATCGTGCAAACAACCAGTGAAATTGATCTTTTGGATGATGGCTACCGGTGGCGCAAGTACGGGCAGAAAGTGGTAAAAGGGAATCCTCATCCAAGAATGGTGTTTGAAAAAGTGGATTCTAGCTTTCATCCTGGAATTTTGGACCTTTCTCACCTCCACACCCCTTGTTGTATTCTGGTTGTCAAGGGCAGAGGGGTTGGGAGGTGA

>Traes_4DS_3BE557D5C Org_Taestivumearly-release CDS: Traes_4DS_3BE557D5C.4 (1 of 16) PF03106//PF10533 - WRKY DNA -binding domain (WRKY) // Plant zinc cluster domain (Plant_zn_clust) (PAC:31752217)

ATGGAGGAGGTGGAGGAGGCCAACAGGATGGCCGTGGCGAGCTGCCACAGAGTGCTTGGCCTGCTCGCCCAGACCCAGGACCCGGCGCAGCTGAGGAGCATAGCTCTGGGCACGGACGAAGCCTGTGCAAAGTTCAGGAAGGTGGTCTCCCTCCTCGGCAACGGCAACGAAGGAGGGGGAACCCATCCTAGAGCCAAGCTTGTGAGCAGAAGGCAGACCCCCGGGTTCTTGAGCCAGAAGAGCTTCTTGGACAACAACACCCCGGTGGTGGTGCTGAACAGTGCGCACCCCTCGACCAGCTCTGCCCAGGTGTATCCCAGAAACAGCAGCATTCTGGATTCACAGCCCGCGCACCCAATCGGGGGGCCTCCCAAGCTGGTCCAGCCGTTGTCCGCGCATTTCCAGTTCGGCGACTCGTCGCGGTATAATCAGTTCCAGCAGCATCAGCACCAGCAGCAGAAGATGCGGGCCGAGATGTTCAAGAGAAGCAACAGTGGGATCAACTTGAAGTTTGACAGCCCCAGTGGCACGGGGACGATGTCGTCCGCGAGGTCCTTCATGTCGTCTTTGAGCATGGATGGCAGCGTGGCCAGCCTGGATGCCAAGTCTTCCTCCTTCCATTTGATCGGTGGGCCTGCCATGAGCGACCCGGTGAATGCGCAGCAGGCGCCAAGGAGGCGATGCTCAGGGCGTGGGGAGGATGGAAATGGCAAGTGTGCTGCAACTGGCAGGTGCCATTGTTCTAAGAGAAGCAGGAAGTTGCGGTTGAAGAGGACGATTAAAGTTCCCGCAATTAGTAATAAAATTGCTGATATACCTCCAGATGAATACTCCTGGAGGAAGTATGGGCAGAAGCCAATTAAGGGCTCCCCTCATCCCAGGGGGTACTACAAATGCAGCAGTGTGAGGGGCTGCCCTGCGCGGAAGCATGTTGAACGTTGCGTGGATGATCCGTCGATGCTCATTGTGACATACGAGGGCGAACATAACCATACGCGAATGCCAACTCAGTCTGCGCAAGCTTAG

>Traes_4DS_CFC487CE5 Org_Taestivumearly-release CDS: Traes_4DS_CFC487CE5.2 (1 of 2) PTHR31221:SF16 - WRKY TRANSCRIPTION FACTOR 62-RELATED (PAC:31752593)

ATGTACACGATGATCCATGAGAGTTATATTTCTATGTATCGTTGGTTAATTTGCAGAGGGAACGAAGGAGGTGAGCGAGGTCATCATGGCGACGAGGAGGAGCAGCAGCAGGGAGCTTGGGCGGAGGCAGCCGGCGGCCAGCCGCTGGTGATGCCGGAGGACGGGTACCAGTGGAAGAAGTACGGCCAGAAGTTCATCAAGAACATCCAGAAAATCAGGAGCTACTTCCGGTGCCGCGACAAGCGGTGCGGCGCCAAGAAGAAGGTGGAGTGGCAGCCGGGCGACCCCAACCTCCGCGTCGTCTACGACGGCGCCCACCAGCACGGCTCCCCGTCGTCAAACGGCGGTGGTCAGGACGCCGACGGCGCCGCCAACCGGTACGATCTCAGCACCCAGTACTTCGGCGGGGCCGGCGCCCCCACGCCGCAGACGCAGTGA

>Traes_4DS_DC3C9DC42 Org_Taestivumearly-release CDS: Traes_4DS_DC3C9DC42.3 (1 of 169) PF03106 - WRKY DNA -binding domain (WRKY) (PAC:31879714)

ATGGCGGCACACGAGGCCTCTGCCGGCGGCGGCGAGGGCGCGCGCTGCACTCCTCCGCGCCCCGCGCTCTCGCTACCCCCGCGCTCCGCCGTCGAATCCTTCTTCGGCTCTGGCGCCACCGCCGCGTCCTTTGCCGAGACCAGCCCCGGCCCGTTCACCCTCGCCGCCGCGCTCTTCCCGGACATGCCCTCCTCGGCCTTCCACGGCTCCTTCACCCAGCTCCTTGTCGGCGCCATGGGCTCCCCAGCCGCACCCCCTTCCCCTCCCTCACCGTTCGCCGTGCCACCGGGGCTCAGCCCCACCGCTCTCGTCGGCCCCTTCCCCCCTACGGGGAATTTTGAAATGTCCCATCAGCAAGCGTTAGCCCAAGTAACAGCACAAGCAGTCCATTCTCAGTATACTGTAGGCAGTCAAGCAGATTACTCACTCCCTTTCTCATCGGCAACAACATCAGCTTTGACATCACAGTTCATCAACTCTTCTGCAAATGTGACATCAATGAAGGAGACAGCAACTCTGCCACTGCATACAGTTAATGATAACCTTAAGTCAAATGAGGTTTCACAAGGATTCCAAACTTCGGCCCTCACCGTGGATAAACCTGCTGATGATGGGTACAATTGGCGAAAGTATGGCCAGAAGGCAGTTAAGGGTGGAGAGTATCCAAGGAGCTACTATAAATGCACCCAGGCGAGCTGTCCAGTTAAGAAGAAAGTGGAGCACTCAGCATATGGACAGATTACTCAAATAATTTATAGAGGCCAACATAACCACCAGCGTCCACCAAAAAGGAGGTCCAAAGATGGTGGCAATTTACTAAATGAAGATGATTTCCCTGAGAACAGAGACACTTTGACTCGATCAGAACCAGGCTCTCAAGATCATTCTGGAAAAGTTGAGGTATCAAATGATGGCATCACAGGGTCTTCAATTTCTAAGAGGAGAGACGGAGGTGATCAATCGTCTGGCTCAAGTGATCGAGAGGAGGACAATGATGAAGCAGGTGATGACAATGGAGATGCCGGTATTGTGAATGCAAACAAAAGGCATGTGCCGGCGCCAGCTCAAAGAATCATCGTGCAAACAACCAGTGAAATTGATCTTTTGGATGATGGCTACCGGTGGCGCAAGTACGGGCAGAAAGTGGTAAAAGGGAATCCTCATCCAAGGAGTTACTACAAATGCACGTACCAGGGATGCGATGTAAAGAAGCATATCGAAAGATGTTCGCAAGACCCAACGGCGGTGATAACTACATATGAAGGCAAGCATAGCCATGATGTGCCAGCAGCTAGGAGCAGCGTGGCTGCTGCTGCCAGTGCCAATGCATCCTCTTCCATTAGCTTACTACATAGAGGGCAGAAGGCAGCATCTAGTAGCCAACGGGTGTTGCCCAGAGCAGCACTACATACATCAGATTCTTCCTTGCAGCTAAAAGAAGAAAACGAGATAACATAA

>Traes_4DS_FE38A59D0 Org_Taestivumearly-release CDS: Traes_4DS_FE38A59D0.1 (1 of 169) PF03106 - WRKY DNA -binding domain (WRKY) (PAC:31768080)

ATGGCCCTGGACTCCGTCCCTTCCTACCCCAGCGACCTGGGATCCAGCGGCAGGGCAACCAGGACCCAGCAAAGAATCCGGAAGGAGGAGCGAACGTGGACGGCGGACACCTACGCGCCGTACGACGACGGGCACCAGTGGAGGAAGTACGGCGAGAAGAAGCTCTCCAACTCCAACTTCCCGAGGTTCTACTACAGATGTACCTACAAGACTGACCTGAAGTGCCCTGCGACAAAACAAGTCCAACAGAAGGACATGAGCGACCCACCATTGTTCACGGTCACTTACTTCAACCATCACAGCTGCAACACCACCTCGAGGCCCATCGGCAGCGCCCCAGACACCACTGAGCAGTCGTCCTCGAGGAGGGCAGTGTCCATCTGCTTCGGGTCGCATGCCACCGGCGAGCAGCCGACATTCCTAACGTCGCCGGGCACACTGCAGTCACCAGCGAGCACAACCAACCAACAGAACGACAGAGGCGCTTACGGTCACCAGTTTCAGTGGACAGACACATCACCGTCGGCAGGCGATGCCCCAGTCAAGATGGAGACCGACAGTCTCGCCGGAACAGGCGCTTCGTCTGGCGCTGCTAGCGGCCATGCTCTGTCGAGGACACTGCTGCCGATCGGCCAGTCGAGATGCATCGAGTACTTTCAGTTCTTGTGA

>Traes_5AL_06A6F9328 Org_Taestivumearly-release CDS: Traes_5AL_06A6F9328.2 (1 of 6) PTHR31221:SF8 - WRKY TRANSCRIPTION FACTOR 57-RELATED (PAC:31972770)

ATGAAGCCGACGACGGCGGGGAAGAAGGGGCAGAAGCGGGCGCGGCAGCAGCGGTTTGCCTTCGTGACCAAGAGCGAAGTTGATCACCTCGAGGATGGGTACAGATGGAGGAAGTATGGGCAGAAAGCCGTCAAGAACAGCCCTTTCCCAAGGAGCTACTACCGGTGCACCAACAGCAAGTGCACCGTGAAGAAGCGGGTGGAGCGGTCGTCGGAGGACCCCTCCGTGGTGATCACCACCTACGAGGGCCAGCACTGCCACCACCAGACCTCCTTCCAGCGCGGTGNNNNNNNNNNNNNNNNNNNNNNNNNNNNNNNNNNNNNNNNNNNNNNNNNNNNNNNNNNNNNNNNNNNNNNNNNNNNNNNNNNNNNNNNNNNNNNNNNNNNNNNNNNNNNNNNNNNNNNNNNNNNNNNNNCGCCGTCTTTGCAGCAGCTCAACGGCGGCGACGAACTGCGAAGGTCGACTAGCTATAGCCCCATGGCCTCCGCGACGCAGACACCGTCCTCGTTGGTGCCTCCAGATGTTTCGTTTGACATGGGACTGCTTGGCGACATCGTGCCTCCAGGAGTAAGAAATGGATGA

>Traes_5AL_6FDB440FB Org_Taestivumearly-release CDS: Traes_5AL_6FDB440FB.1 (1 of 169) PF03106 - WRKY DNA -binding domain (WRKY) (PAC:31752743)

ATGGCAATTTGCAGGAATTACTACCGGTGCACCAACAGCACGAACCAGGGCTGCCCGGCCAAACGAACGGTGCAGCGCAATGACGATGACGGCAGCGACGATGGACGGCCGAAGTATACGGTGGTGTACATCTCGGAGCACAGCTGCAAGGCGACCGAGTCGGCGGCCGTGCCGGTGATCCTCGAGACCACCGTCCGCACCGACACC

>Traes_5AL_7164FEAC3 Org_Taestivumearly-release CDS: Traes_5AL_7164FEAC3.1 (1 of 6) PTHR31429:SF3 - WRKY TRANSCRIPTION FACTOR 40-RELATED (PAC:31988149)

AGCAACAGGAACGTAGGCACGGGCGAGGCCGAGCATGTCGATGTGGACAGCCCGCTGAGCAACGGCACTTGCCGGAGAATCAAGGTCAAGAAGGTCTGCACCAGGATCGACCCATCGGACACGAGCCTCGTGGTGAAAGATGGGTATCAATGGCGGAAGTACGGGCAGAAGGTGACACGGGACAACCCGTCCCCAAGGGCCTACTTCCGATGCGCCTTCGCGCCTTCCTGCCCTGTCAAGAAGAAGGTG

>Traes_5AL_A3653B781 Org_Taestivumearly-release CDS: Traes_5AL_A3653B781.1 (1 of 16) PF03106//PF10533 - WRKY DNA -binding domain (WRKY) // Plant zinc cluster domain (Plant_zn_clust) (PAC:31919715)

ATGCAGCAGAGGAGGAGGTGTGCCGGCAAGGAGGATGGGAGTGGACGCTGTGCAACTGGGAGCAGGTGTCACTGTGCAAAGAAAAGGAAACTAAGGATAAGGAGGTCTATCAAAGTCCCTGCAATCAGCAATAAGGTGGCCGACATCCCGGCCGATGAATTCTCGTGGCGGAAGTATGGCCAGAAGCCAATAAAGGGATCCCCGCATCCTAGGGGTTACTACAAGTGTAGCAGCGTGAGGGGCTGCCCGGCCAGGAAGCATGTCGAGAGGTGCGTCGACGACCCCGCGATGTTGATCGTTACCTACGAGGGCGATCACAACCACAACCACAACCAAGCTGCGGCAGCCCAGCCAGCCTGA

>Traes_5AL_A39A63F43 Org_Taestivumearly-release CDS: Traes_5AL_A39A63F43.2 (PAC:31882179)CGCACGGCGTCCGGTTGGATATGTTCCGAAAGGAGCTGCTCCTATTGGACGAGTGATGGCGTGATGATAGTACTTTGCGCTAGTAGTAATTTTGTTCTGGCTTCAGAATCGCAAACTTTTCTGAGAAAAGGAAAAGAGTTCAGGGCTGAGAACTGGACGTGCCGCCATGGTGTTCTTTTTTGCGCCGTGCTTGACCTGGATCGTGCACAATTTTGCAGGGCATACTACCGTTGCACGTACCAGAAGACCCAGGGATGCGCGGCGACGAAGCAG

>Traes_5AL_B4E8A3115 Org_Taestivumearly-release CDS: Traes_5AL_B4E8A3115.3 (1 of 1) PF00931//PF03106//PF12796 - NB-ARC domain (NB-ARC) // WRKY DNA -binding domain (WRKY) // Ankyrin repeats (3 copies) (Ank_2) (PAC:31838967)

ATGGGTGACAGTGCGCTCCACCTTGCAGCAAGGGCCGGAAATGTTGCCCTTGTGCAGAAGATCTTTGCGGACTGCGATCCGGAGCTGGTCGCGGAATTGACTGACCATCAGAACCAGGACGGTGAGACAGCGCTGTATGTTTCCGCGGAGATGGGGCATGTCGAGGTTGTGTGTGAAATTCTGAAGGTTTGTGATCTGCATTCAGCATTTCTCAAGGCACACAACAGCTTTGATGCATTCCATATTGCAGCAAAGCAGGGCCATCTAGTTGTTTTGCAGGAGCTACTGAAGGCTTTTCCTGCATTAGCTATGACAACAAATTCAGTAAATGCCACAGCTTTATACACTGCCGCACTTCACCGCCACATTGGTATTGTCAATCTTCTACTGGATACAGACCCAAGACTTGCCAGGATTGCAAGAAATAATGGGAAGACAGCTCTGCATATAGCAGCAAGACTGGGCAATGTGGAGGTGGTAGTGTTGTTGTTGAATAAAGATCCGGCGACTGTTTTCAGAATAGACAGGAAGGGACAAACAGCAGTGCACATGGCTTCCAAAGGCCACAATGCTGAAATTCTGCTTGAGCTACTGAAGCCCGATGTCTCAGTAATCCATTTGGAAGATAACAAGGGGGACAGGCCACTGCATGTTGCAACGCGGAAGGGAAAAACCATTATAGTTCAGACGCTAATATCCATTGAAGAGATTGATATCAATGCAATCAATGGAGCTGGAGAGACCGCTTTTGCCATTGCAGAGAAACTGGGTAATGAAGAGCTTGTAAACATCCTGAGGGAGGCTGGTGGAGTAACCGCAGAAGAGCAAGTAAATCCTCCGAAATCAATCAAGCGTTTTAAGCAAACACATGATGTCCAATCGCAGATCAAGCAAAAGCGTCGGACAAATATGCATTTCCACACGATCAGGAAGAGTAGTCAAAAGCTCCACACTGAGGCTCCAGTCTGCGCTTTGGCAGACGCCATGTTCAGACTTCCTGCAAAGCTTGATGAGCTACTGATTAGCCACGTCCACATGCTTCCTAGGGGTGCGGAGGATGAGATACCTCTCATCAAGCAAGATCTGGAAGAGATAATGGCCATTCTGCAGGAGCACGACCACCCAGGGAGAGCGGAAGACCGTGCTATGACGAGCAAGTGCCTGACCAAGGAGGTGCGCGAGCTGTCATACGACATGGAGGATAGCGTCGACCAGTACGTGCACGCCGTCGACACCAAGAGAAGGATTGTTCCTCGCCGTAAAAAGTACAAGATCACCTGTCGTAGGGGCAAGACCACTGCGCGGCTCCCGGAGAAGCTTAAGTGGCGTATATGGATGGCCAACAAGATCAGGGAGTTCAGTGTGCGCTCGCAAGAGGCGCTGCAGCGGTACAGCCTATTTAACCACCCTGGTGCTCATGGCATCAGCACGTCTGCTACTTCTACGAGACATGATGTGTGTTTTGGCTCTTGGTATCCCACACCGTGTGGGGAGCTTGTCGGTATAGATGGACATTTGAATACTCTTGAAGCGTGGTTGGGTAAGGATGGGGAGCAGCAGCTCAAGGTGGTATCTGTTGTTGGATCTGGAGGGGTTGGTAAGACCACACTTTCCAAAGAGCTGTACCGTAGAATCAGAGGGCAATTCGAGTGCCAGGCATTTGTGAGGACGTCCCGGAAGCCCGACATCAGGAGGCTTCTCATCAGCTTGCTCTCACAAGTCCGGCCACACCAAACCCCTCACACTTGGAAATTGCATAGTCTAATTGCCGATATCAGGACACATCTCCACGATAAGAGGTACTTGATCGTCATTGATGATGTATGGGCTACACAAACATGGGATATCATTAATCGTGCTTTGCCGGCTGGTAATCTTTGCAGTAGAATTCTAATAACGACAGAAGTCGAAGATGTAGCTCTGAAATGTTGTGGTTATGACTCTAGGCATGTTCTTATGGTGAAACCACTTGGTTACGATGATTCAAGCAAATTATTTTTCAGCACAGCTTTTGGACTACAATATGAATGTCCTCCAGAACTCTGTGACGCTGCACACAACATTGTGAGGAAATGTGCTGGTTCACCACTAGCAATGGTTACTGTTGCTAGTCTTTTAGTAAGCCAGATTGGCAAACCAGAGAAATGGGATTATGTAAATGAAATCTTTGGTCACGGTTTGAGCACATATCCTAGCTCGGAAGGAATGAAACAAGTACTAAACCTTAGTTACAACAATCTTCCTCATTATTTGAAGGCATGTGTGATGTATCTCAGTATATATGAAGAGGACTACATAATTCAGAAAGATGATTTGGTAAAGCAATGGATAGCTGAAGGTCTTATCCTAGCAACAGAAGAGAAAGACAAAGAGGAAATATCAAGGAGATATTTTGATGAGCTTATCAGTAGCAGAATGATCCTACCTGTGTATACAAATGACAACGATGATGTTTTGTCCTGCACACTGCATCACATGGTACTTGATTTTATCAAACACAAGTCCTTAGAAGAGAATTTTGTCATCGCAATAGATCATAGTCAGACAACTGCACCACTCGCAGACAAGGTTCGTCGACTGTCTCTCCACTTTGGTAATGCAGAAGCAACGCCACCAACAAATATGAGACTATCACAAGTTCGGACTCTCGCATTTTTCGGGGTCATTGAGTGTTTGCCTTCCGTTATAGAGTTTCGGCTTCTTCAAGTCCTAATCCTACATCTTTTTGGCGATGATGAAAGTGTCAGTTTTGATCTCACTGGAATATCTGAGCTTTTTCGGTTGAGATATTTGCATGTCACATGTAATGCCACCTTAGAAGTACCACAAACTCAGATGCGAGGTTTACAATATTTGGAGACACTGAAAATAGATGCAAGAGTAAGTGCAGTTCCGTCGGACATTGTTCATTTGCCGAGCTTGTTGCACCTCAGTCTTCCTGTTGGGACAAATCTACCAAATGGTATTGACCATATGACATCGCTTTGCACACTTGAATATTTTGATATAAATGTTAACTCAATGGAGAATGTGCACAGCCTTGGTGAGCTGACCAATCTTCAGGATCTTCGGCTCACATGTTCTACAGTTCCTTCTTCTTACTTAAAGAGTAAAATCGATAGTATGGGCTCTATTCTTGCGAACCTCAGCAACCTCAGGTCTGTAACTCTGAAGTCTTCAGGTATTCTGGAGAGTGAACCTTACAGCATGATCATTTCCTGTGATGGCTTGAGCAGCGTTTCCTCTCCTCCAGCTCTTCTTCAAAGATTTGAGTGGTTGCCACGCATTTGTACCTTCTCCAGCATCCCTAAGTGGATTAGCCATCTCAACAAGCTCTGCATTTTAAAGATTGGGCTTAGGGAATTAGTGAGCAATGATGTCGCTGCTCTGAGAGGATTGCCTGCACTAACTGTTTTGTCGTTATATGTCCGAGCAAAGCCCGCAGAAAAAATTGTCTTTACTAGGGCAGGATTCTTGGTTCTCAAGTGCTTCAAGTTCAGGTGCAGTGTACCTTGGCTGGAATTTGAGGTGGATGCAATGCCTAATCTCTTGAAACTCAAGCTAAGTTTTGATGCCCATGGAGTAGATCAACATCGTACTATACCTGTCGGCATGGTGCACTTAACAGGCCTTAAGGAAATCTCTGCAAAAATTTGGGGTGCTGGTGCCAATGAAAGAAGGGCTGCAAAATCAGCGCTGATTGATGCTATAAAAATGCATTCGGGATGTCCCACCTCCAGCATACAGTGTTTAGATGGGATGTTCAGTGGTAAGGATGATAATAATAGCGGGATACAAGAGGAAGAACACTTGACTCTGCAAAAGCAATACAATATCAAGGAGGAAGACTCCAAGAAACAGCATGATCTTCCAAAGGACTACATGGATGTTGCATACAAACAAACTTCCAGCAGCAACAATCATAGGAAGTCCAAGCGGATCACACAGGTGAGGATGCAGGTGAGGGTGGGATCGGTGCAGGACAACAGCGCCCTCGAGGATGGCTTTAGCTGGAGGAAGTACGGCCAGAAGGATATCATCGGCTCCATGCACCCAAGAGCTTATTTCCGGTGCACGCACAGGCACGTTAAGGGCTGCCCAGTGACCAAGCAGGTGCAGCGCACGTCTACTGACCCGCTGCTCTTTGACGTCGTGTATCACGGGGAGCACACGTGCTTGGACTCTGTCGGATCCCCTGCGACGTCTTGTGGCCATGTCGCCGGCGTGGAGGTGATGAGCAGAAGTAGGCCCGGAGTTGGATTCGTGTCCCAGTCCCAGGCGGCGTGCAGTAGCCAGGTTATGTCCTCCGAGGTGGTAAGCGGAAGCGGGAGTACGGCGGGACTTTGGGGTGACGAGATTGACATGCCGGACCCGGACCGCGATGACACCGGCATCAGTGCTGACTACCTCGGTGGCTACGAATTTGATGTCAGCGCGTTTTTTGCCTAG

>Traes_5AL_E566BD64E Org_Taestivumearly-release CDS: Traes_5AL_E566BD64E.1 (1 of 169) PF03106 - WRKY DNA -binding domain (WRKY) (PAC:31856791)

CAGAAAGTGGTCAAGGGCAACCCCCGTCCGAGGAGCTACTACAAGTGCACCGCCGAGAATTGCAATGTGCGCAAGCAGATCGAGAGGGCATCCACTGACCCTAGGTGCGTCCTGACGACATACACCGGGCGGCACAACCATGACCCACCGGGCAGGGGAGCTGGAGNNNNNNNNNNNNNNNNNNGGCGGCTCCTCCTCTGA

>Traes_5AL_E644A6A0B Org_Taestivumearly-release CDS: Traes_5AL_E644A6A0B.1 (1 of 169) PF03106 - WRKY DNA -binding domain (WRKY) (PAC:31750780)

ATGCAAAGTCAAGAGAAGATAACACCTGTGAAGCCTGTAGCCTCCAGGCCTTTCTCCAGTTTCACTTCCTTCTCGAAGCTCTTGAAAGACTTCACTGCGACTGGTTCTGCGAAGATCACCTCTCCAGGAGAGACTGTTATAGTTAGGCGGCCGAAGGTAACACGTTTCGCACCGCCACCAAGTGATCTATCTGCAGGAGTTGCTGCAAGCATGTTACAGGATGCTGGTTTAGATACCACACGTGAGAAAATGGTCATCGATCCAGAACAAGTAGTCTCCTGTGACCAGATGACGACATTCCACGATATCAACAAACCAATTCACAGTGTGAAAACCCGTCTGTCCTATGATGGCTACAATTGGAGGAAGTATGGGCAGAAGCAAGTGAAAGGGAGCGAGTTCCCGCGGAGCTACTACAAGTGCACTCACCCAACCTGCCCTGTCAAGAGGAAAGTGGAGACAACAGTAGATGGCCAAATTGCGGAAATTGTGTACAACGGTGAACACAACCATCCCCAGCCCCATCCACCAAAAAAGCCAGCGTCATCGGCAAGTACAGAAGTTGTGGTCCCTGACGCCCATGGCAGCAATGATGCTGGAGCAGAAAGCCAGCTAGGAGGGTGCAACCTCGCTCTTGTTTCAGATCCTGTTGCCGCCGCATTCAAAAGCAGCTGTTATTACGTCGATGAATTTGGAAACACTAGTCCGGTCTATCACTGGAACACAAGCCGAAAGGAGAAGCAATCAAGCATCGCAAATGGCCTGACTTCTGGTGAGGCTGCCCCTGCATTCCAGTCTCCAACAGAATGCGGGTCGTCTGGGGATGCAGCATTCCGTTGGCGCAAGTATGGTCAGAAGGCTGTTAATGGTAATTCATTTCCAAGGAGCTACTACAGATGCAGCACAGCAAGATGCAACGCACGCAAGTTTGTGGAGCGTTCATCGGACAATTCGCTGGTAACCACATATGAGGGAAAGCACAACCATGTGCAACTTCAATGA

>Traes_5BL_0A3D332A8 Org_Taestivumearly-release CDS: Traes_5BL_0A3D332A8.1 (1 of 169) PF03106 - WRKY DNA -binding domain (WRKY) (PAC:31834520)

ATGGAGAGCGTGGATGAAAATGGAGGAAGCCGCCTTGTGGTGACCGAGCTGGGCTACATCAAGGAGCTGGTGAGGCAGCTGGACGTGAACCTGGGAGGCTGCCCCGACCACTGCAAGCGCCTGGCCGCCCAGATCTTCGCTGTGACCGAGAGGTCCATCGGCATGATCAGGTCCGGGCACTTCGACAGCCGGAAGCGCTCCGCCGCCGGCCTCGACTCGCCGCCGTTCTCCGCGACGCCCAGCCCCCTGAGCGACGTTTCGGGCATGCCTTTCCATACCAACAACAAGAAGAGGAAAACAATGGAGAAACGGAAGCATCAGGTCAGGGTGAGCTCGGAGGGAGGAGGAGCAGAGACCCCAGTCGACGACGGCCACAGCTGGAGGAAGTACGGCCAGAAGGACATTCTTGGAGCCAAGCACCCAAGGGGGTACTACCGCTGCACGCACCGCAAGTCCCAGGGATGCGCGGCGACCAAGCAGGTGCAGCGCGCCGACGAGGACCCGGCGCTCTTCGACGTGATCTACCACGGCGAGCACACCTGCGTTCATAAGACGGTGGCGGCCGCGGCGGCCATGGTGCAGCCGGCGGAGGAGAACCCGGACGCGCGTAGGCATCTGCAGAACCTGAGCACGAGCCTGACGGTGAACACCGAGGGGCTCACGGCGGGTCATCAGGGCTGCAGCACCACCACGTCCTTCTGCTTCTCCTCGCAGGCGGCGGGCGTGCTGACGATGCCGCAAGAGCACTACCCGTTCTCCATGCCGTCGACGCCGGAGAACTGCTTTGGGCAAGGCGCGTCGCTGTCGACGTCCCTTGAGCCCTCGCCGGTGACCTCGGACTCGAACCGCTTCTCCATGAGCCCGTTCCAGGCGGAGTGGAGGGCGCGGTCTGAGTACGACGAGGTGGTGTCCGCGCTCGTAGCAGCGGGGACGATGCCGGCGCTCACCATGGAGATGGAGGAGGAGACCGCCTTCTCGCTGGACGAGTTTGAGTTTGACGTTTCTTGCTTCCTTGCATGA

>Traes_5BL_17A712C94 Org_Taestivumearly-release CDS: Traes_5BL_17A712C94.1 (1 of 3) K18835 - WRKY transcription factor 2 (WRKY2) (PAC:31951792)

ATGGCCGGCACCAGCGATCGTGGATCCCTCATGGAGGACTGGATGGCCATGCCGCCGACACCCAGCCCGAGAACGCTCATGTCAAGCTTCTTGAATGAAGACTTCAGCTCTGGTCAATTCTCCAATTTTTTCGGCGAACATGTGAGCAACAAGCCCCATGATCAATCAGAGAAGAGAGGAGAGCTTGTGGATTTGAGGGAGCAAGTGCCTGCTCAGTCAGCTACAGATACAGCTACACCCCAAAAGGATTTTTCCCTGCAACCAAATTCGTTCAATGCTAATCAGAAATCAAACCCACAGGGATCTCTAGCTGAGCGCAGGGCTTCGAGAGCTGGTTTCAGTATCCCCAAGATTGATACATCTCGTGTGGGTTCATCCACAGTTATTCGATCACCCATAGCAATTCCACCTGGTCTTAGTCCAACTACTCTTCTTGAGTCACCGGTCTTTCTCTACAATGCTATGGCACAGCCTTCTCCAACCACTGGCAAACTGTTCGTTGCTTCAGAGGCTAACTCAACAATGCCACCAGATAGCACGTTCAGTAACGATGTTTTTTCCTTCCAACCCCACTCTGGCCCAACAAGTTACTCAAATGTGGAAAAGGGTTACACTGTTTGCCACCAAAACCAGTCGTTGTCAAATATTCATCAGCAGGGATCCAGTCTTCAGTCAAGCTTTACTGCAGCCAAGGACAGTGCCGACGAAACAATCGTTAAACCGAAGACATCTGACTCTGTGTTCAGTGATAATCACTCTTCTGAAGAACAGGAAGATGACGAGGGAGACCAAAATGAAGAATACTCTTCTGCCACAAATAGCAACCCAGCTGAAGATGGATATAACTGGAGAAAATATGGACAGAAGCAAGTTAAGAGCAGTGAGCATCCAAGGAGCTATTACAAGTGCACACACCCAGATTGCCCTGTCAAGAAAAAGGTGGAACGCTCTCAAGATGGTCAGATAACAGAGATAGTCTACAAGAGTTCTCACAATCACCCTTTGCCGCCTCCAAACCGCCGCTCAGGTATCCCTTCGTTGCAAATTAATGATCCACAAGTCCATCTTCTAGAGAAACCTGGTTTGCACACAGGGGTCAACACTGCATCTTTGTGGGAAAATGGTAAAAGTGAGTGCATTCAAGATATGCAAGGTGTTGAGGGAAGACCAGCTGCTGGCCCTCCTGTATCTGCATATGGTGATACATCTATCATGGAGTCCCAAGATGCAGCTGATGTCTCGTCAACGCTGTCCAATGAGATTGATAGAGCAACACAAGGCACCATTTCTTTAGACTGTGATGTAGGTGAAGATGAGACTGAATCCAAAAGAAGGAAACTGGATGCTTTAGCTGCTGTTACCATTCCTACTGCGACCACCACCAGTTCAATTGACATGGTGGCTGCAGCATCAAGAGCTGTCCGGGAGCCTCGTGTTGTGGTTCAGACAACAAGCGAGGTTGACATCCTTGATGATGGTTATCGCTGGCGCAAGTATGGTCAGAAGGTTGTTAAAGGAAACCCAAATCCAAGGAGCTACTACAAATGTACGCACCAGGGCTGTTCAGTGCGCAAGCACGTGGAGAGAGCGTCGCATGATCTGAAATCCGTGATCACGACATACGAGGGGAAGCACAACCATGAAGTTCCAGCAGCCAGAAATAGCGGGAACGCGGGCAGTGCTCCAGCATCTGCGCCGCAGGCCAATCTCTCACACCGCAGGCAAGAACAAGCGCAAGGCAGCTATTCTCAGTTTGGTGGCGCGTCTCCCTTCGGTTCCTTCGGCCTTCCGCCTAGAGGCCATCTGGGAGCGGCAGGCAACTTCCACTTCGGGATGGCCCCTCCAGGCATGTCGATGCCGCCGATGCCCGCCGCTCGCCATCCGTCAATGATGCAGGGCTACCCGGGGCTCATGATGCAGGAAGGACAGATGATGCAGGAAGGACAGATGAAGGCGGAGCCAGACCAGCAGTCTGGCTTCGCAGCGTCATCGGCTTACCAACAGATGATGGGCAGGCCCCCCTTTGGTCCCCAGATGTAA

>Traes_5BL_8688F70C9 Org_Taestivumearly-release CDS: Traes_5BL_8688F70C9.1 (1 of 16) PF03106//PF10533 - WRKY DNA -binding domain (WRKY) // Plant zinc cluster domain (Plant_zn_clust) (PAC:31875786)

ATGGCCGTCGACTTCGTGGGACGCGGCCACGCCCCCCGTGGCCTCGCCCTGGCGGGCGGGCAGCAGCAGCTGGCCTTCCACGAGGCCGCCGCGGCGGGGCTCAGCAGCCTCGAGCTCCTCGTCTCGGCGCTCTCCCCCCGCGCTGACTGCGCACCGCCGCCGCTCGGGGAGATCGCCGACCAAGCGTTATCGGGGTTCCGCCGGGTCATCGACATCCTCGGCCGCACCGGTCACGCCCGCTTCCGCCGCGGCCCTGTTGGAGGAGGCGCCGCCTCGTTGACTCCCCCTCCTGTCTCTTCTCCTCCTCGGATGCCGGCCCGGCCACCGGCACCGGCAGCCTCGCAGCAGTTGGCGCCCCAGAAAAGCCTGACGCTGGACTTCACCAAGCCTTCGAAGACGCCGGCAGCGGCAGCCGCTGCTTCGGTGACGTCGACGTCTTTCTTCTCGTCGGTGACGGCGGGGGGCGAGGGCAGCGTCTCCAAGGGCCCGAGCCAGCTGGTGTCCTCCGGAAAGCCGCCGCTCGCGGCTGGCACCAAACGCAAGCAACAGCAGCAGCAGACGCCCTGCGCGAGCGCCGCGCACTCCGATGCCGCTGCCGCCGCCGGTGGCCGGTGCCACTGCTCGAAGAAGCGCAAGCACCGGGTGAAATACACCACGCGCGTGCCCGCGGTAAGCTCGCGCACGGCGGACATCCCCGGCGACGACTACTCGTGGCGCAAGTACGGGCAGAAGCCCATCAAGGGGTCCCCTTACCCCCGCTGCTACTATAGGTGCAGCACCGCCAAGGGCTGCCCGGCGCGGAAGCACGTCGAACGCGCCACCGACGACCCCGCCATGCTCATCGTCACCTACGAGGGTGACCACCGCCACGACACTTTGCCGCCGGCCGCCGCAAATTGA

>Traes_5BL_90757F0CC Org_Taestivumearly-release CDS: Traes_5BL_90757F0CC.1 (1 of 6) PTHR31429:SF3 - WRKY TRANSCRIPTION FACTOR 40-RELATED (PAC:31986586)

ATGTGTTGCTTCTGGACCATGGGCACGGCGCCGGTCTGCCTCGACCTCATGGTCGGGCGTCCCATGGACCACGAGCCCTCTCCGGTGAGGTGCACCGGCGTGAGAACCGAGGCCGACGTTGCTAGCTCGGCCTGTGACAGAGCACCTCCCATGACCAACGACGAGGCTAAGATCCTGGAGGCGAAGCTGGCGCAGGTGAGCGAGGAGAACCGGAAGCTGACTGAAATGATCGCCTACCTGTACGGCAACCAGGTCTCGCGCCAGAGCCCCGACGGCGAGGGCCAGCAGCGCGCCCGCACGGCCGCGTCGCCGACGCCGCCGGCAGGCAAGAAGAGGAGCCAGGAGAGCATGGACGCCTCGCATTCTTGCGATGTGGAAATTAGCAACAGGAACGTAGGCACGGGCGAGGCCGAGCATGTCGATGTGGACAGCCCGCTGAGCAACGGCACTTGCCGGAGAATCAAGGTCAAGAAGGTCTGCACCCGGATCGACCCATCGGACACGAGCCTCGTGGTGAAAGATGGGTATCAATGGCGGAAGTACGGGCAGAAGGTGACACGGGACAACCCGTCCCCAAGGGCCTACTTCCGATGCGCCTTCGCGCCTTCCTGCCCTGTCAAGAAGAAGGTGCAGAGGAGCGCGGAGGACAGCTCGGTGGTGGAGGCGACGTACGAGGGCGAGCACAACCACCCGCACCCCACGCGGGCCGGCGAGCTGCCGAGCTACGCGGCGCGGAGCGGCGGCTCGGTGCCGTGCTCCATCTCCACCAACTCCTCCGGCCCAACAATCACGCTGGACCTCACCAAGAACGGGGGAGGCGTGCAGGTCTTGGATGCGGGGGAGGCGCAGCCGGACATGAAGAAGGTGTGTCGGGCGGTCGCGTCCCCAGAATTCCAGAGGGCGTTGGTGGAGCAGATGGCTCGCGAGCTCACCGGCGACCAAAAGTTTACCGACGCGCTCGCCGCGGCGATCTTGCGGAAGCTGCCGGATTATTAG

>Traes_5BL_AEF9FE805 Org_Taestivumearly-release CDS: Traes_5BL_AEF9FE805.1 (1 of 16) PF03106//PF10533 - WRKY DNA -binding domain (WRKY) // Plant zinc cluster domain (Plant_zn_clust) (PAC:31966248)

ATGGATGGCATGGTGGAATCCAACAGGGAGGCGGTGCAGAGCTGCCACAAGGTGCTGGACCTCCTCTCCAACCCCCATGGCCAGCTCGTCCCCCACAAGGACCTCCTGGAGGCCACGGGCGCCGCCGTCGCCAAGTTCGGCTCCCTAGCTTCCAAGATCGGCAACGGCAATGGTGGACGACAAGGCCATGCTAGGTTCAGACAAAGGATCAAGAAGCCCATGCCTCTCTTCGACAGCAACCTCTTCCGGGACAGCCCCGCGTCGGCTGCGGCTGCCGATGCTGCTGCTGCAGCACCACCCAAGACATCCAGTCCTGGCCCGAGCACCAGCCTCCAGCTGTTTCCGAGGTACCAGCAGATGGAGGCCTCCTCCTCCAAGGACCCTGTCAGGATCCCAGCAGCCCAGTTCCCCCAGAGGATGGTTGTGGAGAACCCGTCGGTCGGTTCGAACGGCCCGGCTCGCGGGCCGCCGCTCCACCTCGTCCAGCCGGTGTCTGTTGCGCCACCGGCGGGGACGCCGGCACCGGCATTGCCGGCGGCGCATCTCCATTTCATCCAGCAGCAGCAGAGCTACCAGAGGTTCCAGCTCATGCATCAGATGAAGCTGCAGAGCGAGATGATGAAGAGGGGCGGCCATGGTGATCATCAGGGTGGCAGCACTGGTGCTGGCAAGGGTGTCAACCTCAAGTTTGATGGCTCTAACTGTACGGGGTCATCCTCCCGTTCGTTCCTGACATCTCTGAGCATGGAGGGGAGCATGGCGAGCTTGGACGGCAGCCGCTCCAGCCGTCCCTTCCAGCTAGTTAGTGGCTCGCAGACGTCGAGCACCCCGGAGATGGGCCTCATGCAGCAGAGGAGGAGGTGTATCGGCAAGGAGGATGGGAGTGGACGCTGTGCAACTGGGAGCAGGTGTCACTGTGCAAAGAAAAGGAAACTAAGGATAAGGAGGTCTATCAAAGTCCCTGCAATCAGCAATAAGGTGGCCGACATCCCGGCTGATGAATTCTCGTGGCGGAAGTATGGCCAGAAGCCAATAAAGGGATCCCCGCATCCTAGGGGTTACTACAAGTGTAGCAGCGTGAGGGGCTGCCCGGCGAGGAAGCATGTCGAGAGGTGCGTCGACGACCCCGCGATGTTGATCGTTACCTACGAGGGCGATCACAACCACAACCGAGCTGCGGCGGCCCAGCCACAGCCAGCCTGA

>Traes_5BL_B9DD3E76F Org_Taestivumearly-release CDS: Traes_5BL_B9DD3E76F.1 (1 of 169) PF03106 - WRKY DNA -binding domain (WRKY) (PAC:31872762)

ATGGATATGGAGGAGCAGGCCAACGCCGCCGCCACTGCAGCGCGGGAAGGCGACCTCGCCGATGTCGTGGCCCGTGCCAACTCAATGCCCTACTCCGCCGGAGCCCGTCGCCAAGCACCACCACCACCTCCCCCTTCTGCGGCAGCTCGCGTCATGATCCCCTACGAGGAGGAGAGACAACGGCGGCCTGCGAACGTCGCCTGCGGCGGCGGCGGCGGCCAGGTTACCTTTGAGGCCCCGCCGTCAACCGTTGTGGTCGACCCGTACCTTCTGGCGGCGGCTGGTGGATATGGGCTGCCGCAGCAGCAGCAGCACCAGCACCAGCAACTGCTGGCTTTCCAGATCTCTGAGCATGCGTGCTGCGCCGCCGCCGACAGCGACGACCCCATGAGGATCTCGCCACCACCACCACCACCACCACCGGCTCCTCATCATCAGATGATCACCAGTTATTGCGGCATGGCTTGCACGCATATACCATACTGCAGAAAGAACGATGTGAGGAAGGTGGTGTGCATCCCGGCGCCGCCGGTGATGAGCAACCGGGCAGGAGGGGGAGGGGAGGTGATTCCATCTGATCTATGGGCATGGAGGAAGTATGGCCAGAAACCCATCAAGGGCTCTCCTTATCCAAGGGGTTACTACAGATGCAGCAGCTCCAAGGGGTGCCTAGCCCGGAAGCAGGTGGAGCGCAGCCGCAGCGACCCCAACATGCTGGTTATCACCTACACGGCGGAGCACAACCACCCATGGCCTATGCAGCGCAACGTCCTTGCTGGATACGCTCGTGCTCACACGCACGCCGCTGCCAAGAAGCAGCAGAAGATCAGCAGCAGTAGCTCCGCTGATAATGCCGCGAGCTCGTCCTCCAGCAACAGTTTCCACGTCGAGCAGATCAATCCGATCTGCGGCGACCAGCTGCCCGTCAGTTGCAAGATGCCGGATAGCACGGCCACCGCCGGAGATGGTGGTGGCCTGTTGTTTGAAGGCATCCAGCCTGACGAGGTCTTTGCAGAGCTGGAGGAATTGGAGACTGATAATAATCCGATGATGACAAGTGCAAACGTCTACGGATCCAGGGGGGTAAGTAGTAACTACGAGTGGCACAAATTCTAA

>Traes_5BL_D3C383CF5 Org_Taestivumearly-release CDS: Traes_5BL_D3C383CF5.1 (1 of 169) PF03106 - WRKY DNA -binding domain (WRKY) (PAC:31811544)

ATGGCCGATCGGCGAGGCGACGGCATGCGCCAGCAGCCCCCTTACTCGTCGGGCCATCAGGAGCGGGTCTTCGACGGCGGCGGCGGCAGCAGCGGAGGCCCGGCGTTCGGGAACGACTACGATCCTGGTTCATCCTACATGTCGCTTCTTGGCTCCGGCGTCAACCCCCAGCAGTTGCTGCCGGCACCGCCGGCATGGGCGGTCGAGGAGGTGGCCCCGCCGACGATTAATCTCACTCCTCAGTTCTCCATGGCAAACTATGTGCCGACGTCGTCCTACCAGCAACAGCAGCACCAGACGGCTGCCTCCTTCGTGGCACCCCTCGCCGCCAATCTCCACCCCTACCAGTCGTCGTCGTCGTCCTACTTCCAGGCCGACCCGCTGCCGCAATGGCCTCCGCGGGCAATGGCGCCGTCGCCCTCCTCCTCCCTGCTGCCCCGCAACTTCACGCTCCACCAAACGCCGGCGTACCCGCACCACCACGAGCAGCAGATGCACATGCAGCTGCTGCGCGCGGCGGCGCTGGGCGGCCCGCACGCGGCGCCGGCGCCCCCCATCGAGCAGCCGGCCAAGGACGGCTACAACTGGCGCAAGTACGGGCAGAAGCAGCTCAAGGACGCCGAGTCGCCGCGGAGCTACTACAAGTGCACCCGGGACGCCTGCCCCGTCAAGAAGATCGTGGAGCGCTCCTTCGACGGCTGCATCAAGGAGATCACCTACAAGGGCCGGCACACCCACCCGCGGCCCCCCGAGCCCCGGCGCGCCGGAGCGGAGGACGTCGCCGCCCCCGGCAGCGCTCATCAGGAGGACGAGCTGAGCGACGACGAGGATGACGGCGAGGAGGGCCACGACATTGGCAGCGGCGCGGGTGGTCCGGCGGGGCAGAGGGTGGTGAGGAAGCACAAGATCATACTGCAGACGCCGAGCGAGGTTGATCTGCTGGACGACGGCTACCGGTGGCGCAAGTACGGCCAGAAAGTGGTCAAGGGCAACCCCCGTCCGAGGTCTTACATTTCGGGATGG

>Traes_5BL_E294922A9 Org_Taestivumearly-release CDS: Traes_5BL_E294922A9.2 (1 of 3) PTHR31282:SF33 - WRKY TRANSCRIPTION FACTOR 15-RELATED (PAC:31742772)

ATGGAGGGGGTGGAGGAGGCCAACCGGGCGGCGGTCGTGAGCTGCAAGAGGCTGGTCGCGCGCCTCTCGCTGTCTGCCGGCGACCCGTTCCGGCTCGCCGCCGTCGCGGCCGAGACCGAGGAGGCCGTGTCCCGGTTCAGCAAGGTGGTTAACATCCTCGGCAATAGGGTTGGCCATGCGAGGGCGAGGGTCGGCCGGAGGAGCTCGCCGGCGGGCGACCCGATTGCGAGGTGCCTCCTCGAGTACCACCCTCCCCCGCCGGTGCCGTACTGCCCACCTGCCAGTGCCCCCCAACTCCATGGGAGTAGCAGTAGCACTCCTGCGCCGCCGACGCCGCTGAAGCAGATGGCGGTGCCGGTGGCGGCAGCGGCGGCTCCTTGTGCTACAGACAGGGACATGTTCTTCCAGACGCCGCTCCTAGATTTGAGTGGGTGCAGCGTTACTCCCGCCTCCATGCCGCCGTGCAGATCAACGGCTCGAGAGTTTCCGCAGCAGCAGCCGGCGCCGCCGCAGAAGAGGATGCTCGAGCAGCAGCAGAGGCCGGCCAGCAGCGACAACAAGAGGTTCCACTTCGAGCCGAAGCCGGCGAGCGAGAAGCCGTTCCACATCGAGATCCCGGCGGCAAGGAGCGGCAAGGAGCCGGAGGTGATCACCTTCAGCTTCGACAACTCGGTGTGCACCTCGTCGGCGGCCACCTCCTTCTTCACCAACATGAGCAGCCAGCTGATCAGCATGTCGGAGACCTCCGCCTGCGCGCCGGCGTCCAGGAAGGCGGCGCACAAAGCCGACGACGACGGCAAATGCCACTGCCCGAAGAAAAAGTCGGTGCCGGTCGCATACGCCATTGATTTATTGAAGCCGAGGGAGAAGAGGGTGGTGAGGATGCCGGCGGTGAGCGACAAGGTGGCCGATATACCTTCGGACAGCTACTCGTGGAGGAAGTACGGGCAGAAACCCATCAAAGGCTCTCCACACCCAAGGGGATACTACCGGTGCAGCAGCATCAAGGACTGCCCGGCGAGGAAGCACGTGGAGCGGTGTCGCGGCGACGCCGGGATGCTCATCGTCACCTACGAGAACGACCACAACCACGCGCAGCCGCTCGACCTCGCCACGCTCACCGCCAACTCCGAAGTCTGA

>Traes_5BS_C46781248 Org_Taestivumearly-release CDS: Traes_5BS_C46781248.1 (1 of 3) PTHR31221:SF22 - WRKY TRANSCRIPTION FACTOR 3-RELATED (PAC:32024774)

ATGTCGCATCAACAGGCTCTTGCCCAAGTCACAGCCCAAGCAAGCCATTCTCCACTCAGAATGTTTGATCACACTGAACAGACATCGTTTTCAGCAGCTGCAACATCATCTGGAGCTCTACAGAATATGAGCTCTGCAGCCAATGTGGCTGAGATGTCAGAGATGGCGACAACCATATCGAACAATGAGCATGCAGCCTTCCAATCTGCTGAGGCTTCTCACAGGTACCAAGTTCCTGCCCCAGTTGATAAGCCTGCTGATGATGGCTATAATTGGCGGAAGTATGGTCAGAAGGTGGTGAAGGGCAGTGATTGCCCAAGGAGCTACTACAAATGTACTCATCCCAGTTGTCCTGTGAAGAAAAAAGTAGAGCACGCAGAAGATGGCCAAATATCTGAAATAATATATAAGGGAAAACACAATCACCAACGTCCACCAAATAAGCGAGCAAAAGATGGCAGCTCTTTAGCAGCCGAGCAAAATGAACAATCCAATGACACAGCATCTGGTTTGTCAGGTGTTAGGCGAGATCAGGAAGCTGTATATGGAATGTCTGAGCAATTATCTGGTCTAAGTGATGGAGATGATAAGGATGATGGTGAATCTCGGCCAAATGAAGCTGATGATAGAGAGAGTGACTGCAAAAGAAGGAATATACAAATTTCTTCACAGAAGGCCCTGACAGAGTCTAAGATCATTGTACAAACAACCAGTGAGGTTGACCTTTTGGATGATGGTTATAGATGGCGCAAGTATGGGCAGAAGGTGGTCAAAGGAAATCCTCATCCAAGGAGTTACTACAAATGCACGTTTGCTGGGTGCAATGTTAGGAAGCACATTGAGAGGGCCTCGTCAGACCCAAAGGCTGTCATCACAACTTATGAAGGAAAACATAACCATGAACCACCGGTTGGTAGAGGCAGCAACCAGAATGGAGGAAATTCCAACCGGTCACAACAGAAAGGGCCGAATAGCATGTCTAGCAATCAAGCTTCGCATACAAGAACAGACCTCGGCAACGTTAACCAGGGGCAGATCGGGGTCTTGCAGTTTAAAAGGGAAGAATAG

>Traes_5DL_1733FB4DA Org_Taestivumearly-release CDS: Traes_5DL_1733FB4DA.1 (1 of 1) PF00931//PF03106 - NB-ARC domain (NB-ARC) // WRKY DNA -binding domain (WRKY) (PAC:31895273)

ATGCTCTCACAAGTCCGGCCACACCAAACCTCTCACACTTGGAAATTGCATAGTCTAATTGCCGACATCAGGACACATCTCCAAGATAAGAGGTACTTGATTGTAATTGATGATGTATGGGCTACGCAAACTTGGGATATAGTTAGCCGTGCCTTGCCAGATGGTAATCTTTGCAGTGGAGTACTTATAACTACAGAAATAGATGATGTAGCTCTGAAATGTGGTGGTTATGACTCTAAGTATGTTCTTCCGATGAAACCACTTGGTCACGATGATTCAAGCAAATTATTTTTCAGAACAGCTTTTGGTCCACAATATGAATGTCCTCCAGAACTCAGTGATGTTGCAAATAATATAATAAGGAAATGTGCTGGTTTCCCATTAGCAGTGGTTACTGTTGCTGGTCTTTTAGTAAACCAGATGGGCAAACCAGAGCAGTGGGATTTTGTGAATAAATCCTTAGGTTATGGTTTGAGGAAAAATCCTGCTCCAGAGGGGATGAAACAAGTACTAAACCTTAGTTACAACAATCTTCGTCTGCATTTGAAGGCATGCCTGATGTATCTCAGTATATATGAAGAGGACTACATAATTCAGAAGAATGATTTGGTAAAGCAATGGATAGCTGAAGGATTTATCCATGCGACAGAAGAGAAAGATATGGTGGAAATATCAAGGATATGTTTTGATGAGCTCATCAGTAGCAGAATGATCGAACCTGTACATATAAATGACACCGGTGATGTTTTGTCTTGCACAGTGCATCACATGGTACTTGATTTTATTACACACAAGTCCTTGGAAGAGAATTTCGTCACTGCAATAGATCATTGTCAGACAACTGCACGACTCGCAGACAAAGTTCGTCGGCTGTCTCTTCACTTTGGTAATGCAGAAGCGACGCCACCAACAAATATGAGACTATCACAAGTTCGCACTCTCGCATATTTCGGGGTAATCAAGTGTTTGCCTTCCATTGTGGAGTTTGGACTTCTTCAAATTCTAATCCTGCATCTTTGGGGCGATGATGATAGCATCAGTTTTGATCTCACTGGAATATCTGAGCTTTTTCGGCTGAGATATTTACACGTCACATGTAATGCCACCTTGGAAGTACCACAAACTCAGATACGAGGTCTACGATATTTGGAGACACTGAAAATAGATGCAAGAGTAAGTGCTGTTCCATCGGACATTGTTCATTTGCCGGGCTTATTGCACCTAAGTCTTCCTGTTGAGATAAATCTGCCCAATGGTATTGGCCGCATGACATCACTTTGCACACTTGAATGTTTTGATATAAGTGTTAACTCAGTAGAGAATGTGCACAGCCTTGGTGAGCTGACAAATCTTCATGATCTTCGACTCACCTGTTCTACAGTACATTCTTGTTATCTAACGAGCAAAATGGATAGTATGTGTTCCATTCTGACGAAACTCAGCAACCTCAGGTCTCTAACTCTGGAGCCTTCAAGTATTCTGGATGTTGGACCTTCAAGCATGAGCATTTCCTGTGACGGGTTGAGCAGTGTATCCTCTCCTCCAGCGTGTCTTCAGACGTTTGAGTGGTTGCCGCGCATTTGCACCTTCTCCAGCCTCCCCAAGTGGATTGGACGTCTCAGCAAGCTCTGCATTTTAAAGATTGGGGTTAGGAAACTAGCGAACAATGATTTTGATATTCTCAGAGGATTGCCTGCACTCACAGTTTTGTCGCTGCATATCCGAACAAAGCCTGCAAAAAGGATTCTCTTCAATAAGATAGGATTCTCAGTTCTCAAGTACTTCAAGTTCAGGTGCCGTGCACCTTGGCTGGAATTTGAGGTGGACGCAATGCCTAATCTTTTGAAACTCAAGCTACGTTTTGATGCCCATGGAGTAGATCAACATGGTACTATACCTGTTGGCATCGTGCACTTAACAGGCCTGAAGGAAATGTCTGCAAAAATTGGGGGTGCTGGTGCCAATGACCCTGATAGAAGGGCTGCAGAATCAGCTTTGATTGATGCTATTAAGATGCATCCGGCATGTCCCACCTTGAGCATACATTGTTTAGATGCGATGTTCAGTGGTGAAGATGATGATATCAAGAAGGAAGACTCAATTGAACACATGACTCTGCAAAAGCAATACGATGTCAAGAAGGAAGACTCAATTGAACACATGACTCTGCAAAAACAATATGGTATCAAGAAGGAAGACTCAATTGAACACACGACTCTGCAAGAACAATATGATATCAAGAACGAAGACTTAATTGAGCACATGACACTGCAAATACAAAATGATATCAAGAAGGAAGACTCCTATAAACAGCACGGATTTCTTCAAAAGGACTACAGGAAGACCCTGCCCAAGTGGAGCACACAGGTAAGGGTGAGCTCCTTGCAGGATATTGAGGGTCACGATGATGGCTTCAGCTGGAGGAAGTATGGTCAGAAGGATATCCTCGGCTCCAGGAACCCAAGAGGTTACTACCGATGCACGCACCATAACACACGGGGCTGCCAAGCATTGAAGCAGCTGCAGGCCACAGATGGCGACCCACTCTTATTTAATGCCATCTATGTCGGGAACCACACATGCACTCAGGGCGCGAACTCGCAGCCACAGCCTGGGTATGAGCAGAGCTCCATCTCTGTGGGGGACAAGGCTGAGGGGAGCATTCAGAGGCTTGAGAAGATGCCGCCACGGAGGTCCAAGCGAAGCATACAGGTGAGGGTGAGGTCCATGCAGGACGACTATCCGGCAGATGACGGCTATAGCTGGAGTAAGTACGGCCAGAAGGACATTCTCGGGTCCAAGCACCCAAGAGGTTACTACCGGTGCGTGCACAGGCCCGAAAAGGGCTGCGAGGCAACCAAGCAGGTGCAGCGATCGGACAGCGACACACAGCTCTTTGACGTCGTGTACCACGGGGAGCACACATGCGCTGAGAATGTGCATTCGCGGGGTGAGAGTGCGCGTTCACTGCCGCATCATGTCTCTGTCTCCGCGGGGGTCATACCTCCAGCGACGTCTGAGAGCCAGGTTACCTATGAGGCGGTCAGCAGCGGAAGTACAGCCGGGATCCACTTCATGTCCCCGGCAACGTCTGCGGGTAGCCAGGTTACCTACGAGAGTGGCAGCAGAAGTACGACAACGGGTCGATTCATCTCCCCGGGGATGTCAGAGAGCCAGGTTGCCTACGCCGAATTTTGGACTTGGCCCAATAATGTTGACTTTATGCTGAACTCCCCGATAAACGAAAGGCTGGACCTGAATGCTGATTTTGTGGATGAAACTGGCCCTTCTGACTTTGACTGA

>Traes_5DL_2553A6C33 Org_Taestivumearly-release CDS: Traes_5DL_2553A6C33.1 (1 of 3) PTHR31282:SF33 - WRKY TRANSCRIPTION FACTOR 15-RELATED (PAC:31823877)

ATGCCGGCGGTGAGCGACAAGGTGGCCGATATACCTTCCGACAGCTACTCGTGGAGGAAGTACGGGCAGAAACCCATCAAAGGCTCTCCACACCCAAGGGGATACTACCGGTGCAGCAGCATCAAGGACTGCCCGGCGAGGAAGCACGTGGAGCGGTGTCGCGGCGACGCCGGGATGCTCATCGTCACCTACGAGAACGACCACAACCACGCGCAGCCGCTCGACCTCGCCACGCTCACCGCCAACTCCGAANNNNNNNNNNNNNNNNNNNNNNNNNNNNNNNNNNNNNNNNNNNNNNNNNNNNNATGGAACATGCCATTCAGCCTGACCAAATCTGAATGCAAGTGA

>Traes_5DL_32D78D06A Org_Taestivumearly-release CDS: Traes_5DL_32D78D06A.1 (1 of 6) PTHR31429:SF3 - WRKY TRANSCRIPTION FACTOR 40-RELATED (PAC:31917474)

ATTAGCAACAGGAACGTAGGCACGGGCGAGGCCGAACATGTCGATGTGGACAGCCCTCTGAGCAATGGCACTTGCCGGAGAATCAAGGTCAAGAAGGTCTGCACCAGGATCGACCCATCGGACACGAGCCTCGTTGTGAAAGATGGATATCAATGGCGGAAGTACGGGCAGAAGGTGACACGGGACAACCCGTCCCCAAGGGCCTACTTCCGATGCGCCTTCGCACCGTCCTGCCGTGTCAAGAAGAAGGTCCAGAGAAGCGCGGAGGACAGCTCGGTGGTGGAGGCGACGTACGAGGGCGAGCACAACCACCCG

>Traes_5DL_46E3AC8D6 Org_Taestivumearly-release CDS: Traes_5DL_46E3AC8D6.1 (1 of 169) PF03106 - WRKY DNA -binding domain (WRKY) (PAC:31972964)

ATGGCCGATCGCCGTGGCGACGCCATGCGCCAGCAGCCGCCTTACTCGTCGGGCCATCAGGAGCGGGTCTTCGACGGCGGTGGCGGCCCGGCGTTCGGGAACGACTACGATACTGCTGGATCATCCTACATGGCGCTTCTTGGCTCCGGCGTCAACCCCCAGCAGTCGCTGCCGCCGCAGCAGGCGTGGGGGGTCGACGAGGTGACCCCACCGACGATCAATCTCACTCCTCAGTTCTCCATGGCAAACTATGCGCCGACGTCGTCCTACCAGCAGCACCAGACGACTGCATCCTTCGTCTCACCCCTCGCCGCCAACCTCCACCCCTACCCGTCGTCGTCGTCGTCGTCCTACTTCCAGGCCGATCTGCCGCCGCAATGGCCTCCGCGGGCAATGGCGCCGTCGCCCTCCTCCTCCCTGCTGCCCCGCAACTTCACGGTCCACCAAACGCCGGCGTACCCGCACCACCACGAGCAGCAGATGCACATGCAGCTGCTGCGCGCGGCGGCGCTGGGCGGCCCGCACGCGGCGCCGGCGCCCCCCATCGAGCAGCCGGCCAAGGACGGCTACAACTGGCGCAAGTACGGGCAGAAGCAGCTCAAGGACGCCGAGTCGCCGCGGAGCTACTACAAGTGCACCCGGGACGCCTGCCCCGTCAAGAAGATCGTGGAGCGCTCCTTCGACGGCTGCATCAAGGAGATCACTTACAAGGGCCGGCACACCCACCCGCGCCCCCCCGAGCCCCGGCGCGCCGGAGCGGAGGACGCCGCCGCCCCCAGCAGCGCCGTCGGCGCTCATCAGGAGGACGAGCTGAGCGACGACGAGGATGACGGCGAGGAGGGCCACGACATTGTCAGCGGCGCGGGTGGTCCGGCGGGGCAGAGGGTGGTGAGGAAGCACAAGATCATACTGCAGACGCCGAGCGAGGTTGATCTGCTGGACGACGGCTACCGGTGGCGCAAGTACGGCCAGAAAGTGGTCAAGGGCAACCCCCGTCCGAGATCATATTATCAAAAAACGTCTTTCATTTGGGGACGG

>Traes_5DL_4BA2CC560 Org_Taestivumearly-release CDS: Traes_5DL_4BA2CC560.1 (1 of 169) PF03106 - WRKY DNA -binding domain (WRKY) (PAC:31924879)

ATGCAAAGTCAAGAGAAGATAACACCTGTGAAGCCTGTAGCCTCCAGGCCTTTCTCCAGTTTCACTTCCTTCTCGAAGCTCTTGAAAGACTTCACTGCGACTGGTTCCGCGAAGATCACCTCTCCAGGAGAGACTGTTATAGTTAGGCGGCCGAAGGCAACACGTTTCGCACCGCCACCAAGTGATCTATCTGCAGGAGTTGCTGCAAGCATGTTACAGGATGCTGGTTTAGATACCACACGTGAAAAAATGGTCATCGATCCAGAACAAGTAGTCTCCTGTGACCAGATGACGGCATTCCACGATATCAACAAACCAATTCACAGTGTGAAAAACCGTCTGTCCTATGATGGCTACAATTGGAGGAAGTATGGGCAGAAGCAAGTGAAAGGGAGCGAGTTCCCGCGGAGCTACTACAAGTGCACTCACCCAACCTGCCCTGTCAAGAGGAAAGTGGAGACAACAGTAGATGGCCAAATTGCGGAAATTGTGTACAACGGTGAACACAACCATCCCCAGCCCCATCCACCAAAAAAGCCAGTGTCATCGGCAAGTACAGAAGTTGTGGTCCCTGACGCCCATGGCAACAATGATGCTGGAGCAGAAAGCCAGCTAGGAGAGTGCAACCTCGCTCTTGTTTCAGATCCTGTTGCCGCGGCATTCAAAAGCAGCTGTGATTACGTCGATGAATTTGGAAACACTGGTCCGGTCTATCACTGCAACACAAGCCCAAAGGAGAAGCAATCAAGTATCGCAAATGGCCTGCCTTCTTCTGGTGAGGCTGCTCCTGCATTCCAGCCTCCAACAGAATGCAGGTCGTCTGGGGATGCAGCATTCCGTTGGCGCAAGTATGGTCAGAAGGCTGTTAATGGTAATTCATTTCCAAGGAGCTACTACAGATGCAGCACAGCAAGATGCAACGCACGCAAGTTCGTGGAGCGTTCATCGGACAATTCGCTGGTAACCACATATGAGGGAAAGCACAACCATGTGCAACTTCGATGA

>Traes_5DL_5C93510D5 Org_Taestivumearly-release CDS: Traes_5DL_5C93510D5.1 (1 of 169) PF03106 - WRKY DNA -binding domain (WRKY) (PAC:31747511)

ATGGAGGCCGTGCATGAGGGCAATGGAGGAGGGAGCGGCCTGGTGGTGACGGAGCTGAGCCACATCAAGGAGCTGGTGAAGCAGCTGGACGTGCACCTGGGGGGCTCGCCCGACCTCTGCAAGCTCCTCGCCCAGCAGATCTTCGCCGTCACCGAGAGGTCCATCGGCATGATCAGGTCCGGCCACTTCAACGGCCCCAAGCGCCCGGCCGCCGGCGCCGGCCTCGACTCGCCGCCGCTGTCCCCCACGCCCAGCCCCCTCAGCGGCGTCTCCAACACGCCGTTCAAGCCCAACAAGAAGAGGAAGACGTCGGAGAAAGGGGGGCGTCAGATCAGGGTGAGCTCGGCGGCGGGAGGGGCGGACGCCCCCGCCGACGACGGCCGCAGCTGGAGGAAGTACGGCCAGAAGGACATCCTCGGAGCCCAGCACCCAAGGGCGTACTACCGTTGCACGTACCAAAAGACTCAGGGATGCGCGGCGACGAAGCAGGTGCAGCGCGCCGACGAGGACCCGGCGCTCTTCGACGTGATCTACCACGGCGAGCACACCTGCGTCCATAAGACGGCGGCGGCGGCGGTGCAGCCGGCCGGGCAGAACCCGGGCGCGGAAAGCCTTCTGCAGAGCCTGAGCTCGAGCCTGACGGTGAAGACCGAGGGGCTCACGGCGGCGGGAGCGCAGGGCTGGAGCGCCACCACGCCCTTCAGCTTCTCCTCGCCGGCGGTGAGCGGCATGACGCCGCCGGAGCACCACCCGTTCTCCACGCCGTCGACGCCGGAGAACTGCTTCGTGTCGATGCCCACGTCGCTCGAACCCTCGCCGGCGACCTCGGGCTCGAACCACATGTGCATGACCCCGTTCCATGCGCAGTCCGAGCTCCAAACAATGGTGTCAGCGCTCGTGGAGGCGACGAGCATGCCGGCGGCCGGCACGGAGGAGGCTGCCTTCTACACATTCCAGTCCGATTGGAGCTTCGACGATTCTGCCTTGGACGTTAACAACTTCGACGTTTCTGCCTTGGACGTCAATAACTTCGACGTTTCTGTCTTCTTGGCATGA

>Traes_5DL_A54ED44C9 Org_Taestivumearly-release CDS: Traes_5DL_A54ED44C9.1 (1 of 16) PF03106//PF10533 - WRKY DNA -binding domain (WRKY) // Plant zinc cluster domain (Plant_zn_clust) (PAC:31821176)

ATGGATGGCATGGTGGAATCCAACAGGGAGGCGGTGCAGAGCTGCCACAAGGTGCTGGACCTCCTCTCCAACCCCCATGGCCAGCTCGTTCCCCACAAGGACCTCGTGGAGGCCACGGGCGCCGCCGTCGCCAAGTTCGGCTCCCTAGCTTCCAAGATCAGCAGCGGCAATGGCCGACAGGGCCATGCTAGGTTCAGACAAAGGATCAAGAAGCCCATGCCTCTCTTTGACAGCAACCTCTTCCGGGACAGCCCCGCATCGGCATCGGCTGCCGATGCTGCTGCCGCTGCACCCAAGACGTCCAGTCCTGGCCCGAGCACCGGTCTCCAGCTGTTTCCGAGGTACCAGCAGATGGAGGCCTCCTCCTCCAAGGACCCTGTCAGGATCCCAGCAGCCCAGTTCCCCCAGAGGATGGTTGTGGAGAACCCGTCGGTCGGTTCGAACGGGCCGCCGCTCCAGCTCGTCCAGCCGGTGTCTGTCGCGCCCCCGGCGGGGACGCCGGCACCGGCATTGCCGGCAGCGCATCTCCATTTCATCCAGCAGCAGCAGAGCTACCAGAGGTTCCAGCTCATGCATCAGATGAAGCTGCAGAGCGAGATGATGAAGAGGGGCGGCCATGGTGATCATCAGGGTGGCAGCACTGGTGCTGGCAAGGGTGTCAACCTCAAGTTTGATGGCTCTAACTGTACGGGCTCATCCTCCCGTTCGTTCCTGACATCTCTGAGCATGGAGGGGAGCATGGCGAGCTTGGACGGTAGCCGCTCCAGCCGTCCCTTCCAGCTAGTTAGTGGCTCGCAGACGTCGAGCACCCCGGAGTTGGGCCTCATGCAGCAGAGGAGGAGGTGCGCCGGCAAGGAGGATGGGAGTGGACGCTGTGCAACTGGGAGCAGGTGTCACTGTGCAAAGAAAAGGAAACTAAGGATAAGGAGGTCTATCAAAGTCCCTGCAATCAGCAATAAGGTCGCCGACATCCCGGCTGATGAATTCTCGTGGCGGAAGTATGGCCAGAAGCCAATAAAGGGATCCCCGCATCCTAGGGGTTACTACAAGTGTAGCAGCGTGAGGGGCTGCCCAGCGAGGAAGCATGTCGAGAGGTGCGTCGACGACCCCGCGATGTTGATCGTTACCTACGAAGGCGATCACAACCACAACCGAGCTGCGGCAGCCCAGCCACAGCCAGCCTGA

>Traes_5DL_C93641E43 Org_Taestivumearly-release CDS: Traes_5DL_C93641E43.1 (1 of 169) PF03106 - WRKY DNA -binding domain (WRKY) (PAC:31745499)

ATGGAGAGCGTGGATGAAAATGGAGGAAGCCGCCTTGTGGTGACCGAGCTGGGCTACATCAAGGAGCTGGTGAGGCAGCTGGACGTCAACCTGGGAGGTTGCCCCGACCACTGCAAGCGCCTGGCCGCCCAGATCTTCGCCCTGACCGAGAGATCCATCGGCATGATCAGGTCCGGGCACTACGACTGCCGGAAGCGCTCCGCCGCCGGCCTCGACTCGCCGCCGTTCTCCGCGACGCCCAGCCCCCTGAGCGACGTTTCCGGCATGCCTTTCCATAACAACAAGAAGAGGAAGACAATGGAGAAACGGAAGCATCAGGTCAGGGTGAGCTCGGAGGGAGGAGGAGCAGAGACCCCAGTCGATGACGGCCACAGCTGGAGGAAGTACGGCCAGAAGGACATCCTTGGAGCCAAGCACCCAAGGGGATACTACCGCTGCACGCACCGCAAATCCCAGGGATGCGCGGCGACGAAGCAGGTGCAGCGCGCCGACGAGGACCCGGCGCTCTTCGACGTGATCTACCACGGCGAGCACACCTGCGTCCATAAGACGGTGGCGACCGCGGCGGCGGCCATGGCGCAGCCGGCGGAGGAGAACCCGGACGCGCGTAGGCATCTGCAGAACCTGAGCACGAGCCTGACGGTGAACACCGAGGGACTCACGGCGACGGCGGGTCATCAGGGCTGCGGCACCACCACGTCCTTCTGCTTCTCCTCGCAGGCGGCGGGCGTGCTGACGACGCCGCAAGAGCACTACCCGTTCTCCATGCCGTCGACGCCGGAGAACTGCTTTGGGCAGCGCGCATCGCTGTCGACGTCCCTTGAACCCTCGCCGGTGACCTCGGATTCGAACCGCTTCTCCATGAACCCGTTCCAGGCGGAGTGGAGGGCGCAGTCTGAGTACGACGAGGTGGTATCCGCGCTCGTCGCTGCGGGGACGATGCCGGCGCTCGCCATGGAGATGGAGGAGGAGACCGCCTTCTCGCTGGACGAGTTTGAGTTTGACGTTTCTAGCTTCCTTGCCTGA

>Traes_5DL_E4A6D1889 Org_Taestivumearly-release CDS: Traes_5DL_E4A6D1889.2 (1 of 169) PF03106 - WRKY DNA -binding domain (WRKY) (PAC:31898234)

ATGGATATGAAGGAGCAGGCCAACGCCGCCGCCACTGCTGCGCGAGAAGGCGACCTCGCCGACGTCGTGGCCCGTGCCAACGCAATGGCCTACTCCACCGGAGCCCGTCGCCAAGCGCCACCACCTCCCCCTTCTGCGGCAGCTCGCGTCATGATCCCCTACGAGGAGGAGAGACAACGGCGGCCTGCGAACGTCGCCTGCGGCGGCGGCGAGGTTACCTTTGAGGCACCGCCGTCAACCGTTGTGGTCGACCCGTACCTTCTGGCGGCGGCTGGTGGATATGGGCTGCCACAGCAGCACCAGCACCAGCACCAGCAACTGCTCGCTTTCCAGATCTCTGAGCATGCGTGCTGCGCCGCCGCCGACAGCGACGACCCCATGAGGATCTCGCCACCACCACCACAACCGGCTCCTCATCATCAGATGATCACCAGTTATTGCGGCATGGCTTGCACGCATATACCATACTGCAGAAAGAACGATGTGAGGAAGGTGGTCTGCATCCCGGCGCCACCGGTGATGAGCAACCGGGCAGGAGGGGGAGGGGAGGTGATTCCATCTGATCTATGGGCATGGAGAAAGTATGGCCAGAAACCCATCAAGGGCTCTCCTTATCCAAGGGGTTACTACAGATGCAGCAGCTCCAAGGGGTGCCTAGCCCGGAAGCAGGTGGAGCGCAGCCGCAGCGACCCCAACATGCTGGTTATCACCTACACGGCGGAGCACAACCACCCATGGCCAATGCAGCGCAACGTTCTTGCTGGATACGCTCGTGCTCACACGCACGCCGCTGCCAAGAAGCAGCAGAAGATCAGCAGCAGTAGCTCCGCTGATAATGCCGCGAGCTCGTCCTCCATCAACAGTTTCCACGTCGAGCAGATCAATCCGATCTGCGTCGACCAGCTGCCCGTCAGTTGCAAGATGCCGGATAGCACGGCCACTGCCGGAGATGGTGGTGGCCTGTTGTTTGAAGGCATCCAGCCTGACGAGGTCTTTGCAGAGCTGGAGGAGTTGGAGACTCATAATAATCCCGTGATGACAAGTGCAAACGTCTACGGATCCAGGGGGGTAAGTAGTAACTACGAGTGGCACAAATTCTAA

>Traes_6AL_BA4636569 Org_Taestivumearly-release CDS: Traes_6AL_BA4636569.1 (1 of 169) PF03106 - WRKY DNA -binding domain (WRKY) (PAC:31787421)

ATGCATATATGCATGGATCAAGGGAGCCAATTGGGGATGGCTTACTGTCTTCCTAACCTCAGTGTGCCAGATCACTACTACACCACACCTGTTCCTCTTTCTCCACTTCAACTACCCTTCCATCCCAAGCCTCTCCAGATGCCATTCGACCAGGAAGAAGCCTTGATGCTCTCTTCTGACCATTGTGGGCTATACCCGCTGCCGGCGCTTCCGCTCGGCAGCGGCCACTCCGCCGGCGCGCCTGCCATCGTCTGCGAGAAGCCCACGGTCGGTTTCATGCCTAATATTGGGGCTGAGGAGGTGGGCACGTCGGTGACGGCAAGAGTTGGCTATGAGGGTGCCACTGCCTGTAATGGTTATTCTAGTAATACATGGTGGAGGGGCTCGACGATGTTGGCGGGGGAGAAGGGGAAGATGAAGGTGAGGAGGAAGATGAGGGAGCCGAGGTTTTGCTTCCAGACCAGAAGCGACGTGGATGTACTTGACGATGGCTACAAGTGGAGGAAATACGGGCAGAAGGTTGTCAAGAACAGTCTGCATCCCAGGAGCTACTTCAGGTGCACGCACAGCAACTGCCGCGTGAAGAAGCGCGTGGAGCGGCTGTCGACGGACTGCCGCATGGTGATCACCACGTACGAGGGCCGCCACACGCACCCCCCCTGCGACGACAATTCCTCCTCCTCCGGCGACAACACCACCACCTGCTTCTGA

>Traes_6AS_68775100B Org_Taestivumearly-release CDS: Traes_6AS_68775100B.1 (1 of 1) PTHR31282:SF1 - WRKY TRANSCRIPTION FACTOR 38-RELATED (PAC:31916438)

ATGGGCAACATCGAGCTTCCACCGGACGACGGTTACACGTGGCGCAAGTACGGCCAGAAGGACATCCTCGGCTCTAGGTTCCCAAGGAGCTACTACCGGTGCACCCACAAGAACTACTACGGGTGCGACGCCAAGAAGAAGGTGCAGCGCCTGGACGACGACCCCTTCATGTACGAGGTCACCTACTGCGGCAGCCACAGCTGCCTCACCTCCACCACCCCGCTGCTCAACTTTCCCACCGCCACCGCCACAGCCACCGCTACCAACTCTCCGACCGCCGCGACAGGCTCCGGCCTGGCCCCTGCGGACCACTTCATGGCGCCCACCGAGCAGGCGGCCGTGTCGACATCAATGCACCTCGGCGTCGGCTGGATGCCGGCAAGCTTCCAGGGCGTTGTGGCGGGCTCCGGTGCCGGCGGGGGGAGTAGCGCCGGCATGCAGACGAGCGTGTCCACCGCAGCAAGGGACACGGATTACCCGGCGCTGGACCTTGCCGACGTCATGTTCAACTCCGGCGGTAGCGTCGGCATGGACGGCATCTTCTCTTCTCATCATCGACGTGATAGCTAG

>Traes_6AS_DA75BB1FD Org_Taestivumearly-release CDS: Traes_6AS_DA75BB1FD.1 (1 of 169) PF03106 - WRKY DNA -binding domain (WRKY) (PAC:31748920)

ATGGATCCATGGGTCAGCAGCCAGCCTTCGCTGAGCCTCGATCTGCACGTCGGCCTCCCGCCGATGGGGCACCACCAGGCGGCGCCGATGGTGGCGCTGGCCAAGCCCAAGGTCCTCGTCGAGGAGAACTTCATGCAGCTCAAGAAGGACCCTGAGGTTGCGGTTCTTGAGTCGGAGCTACAGCGTGTGAGCGAGGAGAACCGCCGCCTGGGCGAGATGCTCAGGGAGGTGGCCTCCAAGTACGAGGCCCTGCAGGGCCAGTTCACCGACATGGTCACGGCCGGCGCCCACGCCGGCGGCAACAACAGCCACTACAACAACCAGCCGTCCTCCGCGTCGGAGGGCGGGTCGGTGTCGCCGTCGAGGAAGCGCAAGAGCGAGGAGAGCCTCGGCACGCCGCCGCGGCCGTCGCAGCACCAGCAGCAGCACTACGCCGGCGGCCTCGCGTACGCGGCGGCGCCGGACCAGGCGGAGTGCACGTCGGGCGAGCCGTGCAAGCGCATCCGGGAGGAGTGCAAGCCCGTCGTCTCCAAGCGCTACGTCCACGCCGACCCCTCCGACCTCAGCCTGGTGGTGAAGGACGGGTACCAATGGCGCAAGTACGGGCAGAAGGTGACCAAGGACAACCCCTGCCCCCGAGCCTACTTCCGCTGCTCCTTCGCCCCCGGCTGCCCCGTCAAGAAGAAGGTGCAGAGGAGCGCCGAGGACAAGACCATACTGGTGGCGACGTACGAGGGCGAGCACAACCACACCCAGCCCCCGCCGTCGCAGCCGCAGCAGCAGAACGACGGCTCCGGCGCGGGCAAGAACGCTGGGAAGCCGCCCCAGGCGCCGACTGCCACGCCTCACCACCCGCAGCAGCAGCACAAGCAGGAAGCGGCAGCGGCCGCCGTCAGCGGC

>Traes_6BL_B92FA1D38 Org_Taestivumearly-release CDS: Traes_6BL_B92FA1D38.1 (1 of 5) PTHR31429:SF1 - WRKY FAMILY TRANSCRIPTION FACTOR-RELATED (PAC:31835704)

ATGAACGACGGGTGCCAATGGCGGAAGTACGGCCAGAAGGTCGCCAAGGGGAACCCGTGCCCCAGAGCCTACTACCGGTGCACCGTCGCCCCGGCCTGCCCAGTGCGGAAGCAGGTGCAGAGATGTCAGGAGGACATGTCGATCCTGATCACCACGTACGAGGGCACGCACAAC

>Traes_6BL_DD840863A Org_Taestivumearly-release CDS: Traes_6BL_DD840863A.1 (1 of 169) PF03106 - WRKY DNA -binding domain (WRKY) (PAC:31938936)

ATGCATATATGCATGGATCAAGGGAGCCAATTGGAGATGGCTTACTGTCTTCCTAACCTTAGTGTGCCAGATCACTACTACACCACCCCTGTTCCTCTTTCTCCACTTCAACTACCCTTCCATCCCAAGCCTCTCCAGATGCCATTCGACCAGGAAGAAGCCTTGATGCTCTCTTCTGACCATTGTGGGCTATACCCGCTGCCGGCGCTTCCGTTCGGCGGCCATTCCGCCGCCGCGCCTGCCACCGTCTGCGACAAGCCCACGGTCGGTTTCATGCCTAGTATTGGGGCTGAGGAGGTGGGCACGTCAGTGACGGCAAGAGTTGGCTATGAGGGTGCCACTGCCTGTAATGGTTATTCTAGTAATACATGGTGGAGGGGCTCAACGATGTTGGCGGGGGAGAAGGGGAAGATGAAGGTGAGGAGGAAGATGAGGGAGCCGAGGTTTTGCTTCCAGACCAGAAGCGACGTGGATGTGCTGGACGATGGCTACAAGTGGAGGAAATACGGGCAGAAGGTTGTCAAGAACAGTCTCCATCCCAGGAGCTACTTCAGGTGCACGCACAGCAACTGCCGCGTGAAGAAGCGCGTGGAGCGGCTGTCGACGGACTGCCGCATGGTGATCACCACGTACGAGGGCCGCCACACGCACCCCCCCTGCGACGACAACTCCTCCTCCTCCGGCGACAACACCACCACCTGCTTCTGA

>Traes_6BL_EEAA2A7E3 Org_Taestivumearly-release CDS: Traes_6BL_EEAA2A7E3.1 (1 of 9) PTHR32096:SF18 - DISEASE RESISTANCE PROTEIN-LIKE-RELATED (PAC:31766778)

ATGTCGTCCTCACCGCGTGGCGGGGGCATCAAGCGCAGGAAGAACCAGGCAAGGAAGGTGGTGTGCATCCCAGCACCTGCAGCAGCAGTGGCCGGGAAGACCACTGGGGAGGTTGTTCCTTCTGATCTCTGGGCTTGGAGGAAGTATGGCCAGAAGCCTATCAAAGGCTCCCCCTACCCAAGAGGGTACTACAGATGCAGTAGCTCCAAGGGGTGCCCTGCACGGAAGCAAGTGGAGCGCAGCCGGACGGACCCCAACATGCTGGTCATCACCTACACCTCGGAGCACAACCACCCGTGGCCGACGCAGCGCAACGTGCTCGCCGGCTCCACGCGGTCCCACTACGCCAAGAATAGCAGCAGCAACACAGCTGCAGCTGCGTCCAAGAACAGCAAGAACTGCTCGCGCAATCAGCACAAGCCAGTCGTCAAGGCAGAA

>Traes_6DL_D4F2CDDDC Org_Taestivumearly-release CDS: Traes_6DL_D4F2CDDDC.1 (1 of 9) PTHR32096:SF18 - DISEASE RESISTANCE PROTEIN-LIKE-RELATED (PAC:31827697)

ATGTGCGACTACTTCTTGAAGAGGGCTGATGGCGACCAGCAGGCCGGAGACCTCACCGACATCGTCCGAGCCGGCGGAGCCATGCCGGCTGGCTCTACGGATCCCCCGTCGACGGCCACCGAGTGGCTGCAGCTGCCGGCTGACCCGATCCTCTTCCCGCTGCCCCAGACGTCGTCCTCGGACGGTGCTGGGCCGAGCAGCGCAGACGCTCTCGGTGACCCGTTCTCCGGCCTCCCGGATGCCTTCAGCACCGACTACCCCTCCTCCTCCGGCAGCGCCGCCGCCGACTTCTTCGACGCCGTCCAGGACGCGATGGGTGTCGGCATGGCCAAGCAGGTCGGCTTCGTCGACACGACCGGCTGCGGCGGCGGTGGAACAACTGTTGGTGCTGGTGGCGGGTTCCTGGACATGAGGAATCACCATATGTTTCCAGGGGAGATGCCGATGCGCGTGCTGTCGCCGTACGCGCTGATGGGCGGTGGCGCGGCGAAGCTCGGCGTGCCGATGGCCGGGCACGGGCAGGCGGCTGGCCCGTGCGCGTTCGACGCCGTCGCAGGGCTGCAGATGTCGTCCTCACCGCGTGGCGGGGGGATCAAGCGCAGGAAGAACCAGGCAAGGAAGGTGGTGTGCATCCCAGCACCTGCAGCAGCAGTGGCCGGGAAGACCACTGGGGAGGTTGTTCCTTCTGATCTCTGGGCTTGGAGGAAGTATGGCCAGAAGCCTATCAAAGGCTCCCCCTACCCAAGAGGGTACTACAGATGCAGTAGCTCCAAGGGGTGCCCTGCACGGAAGCAAGTGGAGCGCAGCCGGACGGACCCCAACATGCTGGTCATCACCTACACCTCGGAGCACAACCACCCATGGCCGACGCAGCGCAATGTGCTCGCCGGCTCCACTCGGTCCCACTACGCGAAGAACAGCAGCAACACAGATGCAGCTTCGTCCAAGAACAGCAAGAACTCCTCGCGCAACCAGCACAAGCCAGTCGTCAAGGCAGAATCAAAGGATCAATCCGCCGCCACCCCGGCCGCCACGAGCACGACCACCACGGCGACCACAAGTACCGGCAACAACACACCTCCGATGGCTGTGAAAGAGGAGGCAGAAATGGAGAGAAGGATAGGCGGTGATACTACAGCAACGGTGGGTTATTACAGTGACCATCTCCTGCAGCAGATGTTCAGCCAGAGCTACAGGCCGATGATGCCCGAGGAGGCCGGCGGCTATCACCACCAGGACGACTTCTTCGCCGACCTCACTGAGCTGGACTCCGACCCTGTCAGCCTCATCTTCTCCACGGAGTACATGGAGGCCAGACCTGGCAAGGAGAAGGCAGCAGCCAAGGACGACGTGGATTCATTATTCATGATGGACTGGGCGCCCGCTAGTGCTGCTGTTACTACTTCTGCAGGGAGCGCGCTTGAGCAAGGGGACATGGGTTTATGA

>Traes_6DS_8F684013D Org_Taestivumearly-release CDS: Traes_6DS_8F684013D.1 (1 of 169) PF03106 - WRKY DNA -binding domain (WRKY) (PAC:31785825)

GAGGGCGGGTCGGTGTCGCCGTCGAGGAAGCGCAAGAGCGAGGAGAGCCTCGGCACGCCGCCGCCCTCGCACCAGCAGCACTACGCCGCCGGCCTCGCGTACGCGGCGGCGCCGGACCAGGCGGAGTGCACGTCCGGCGAGCCGTGCAAGCGCATCCGGGAGGAGTGCAAGCCCGTCGTCTCCAAGCGCTACGTCCACGCGGACCCCGCCGACCTCAGCCTGGTGGTCAAGGACGGGTACCAATGGCGCAAGTACGGGCAGAAGGTGACCAAGGACAACCCCTGCCCCAGAGCCTACTTCCGGTGCTCCTTCGCCCCCGGCTGCCCCGTCAAGAAGAAGGTGCAGAGGAGCGCCGAGGACAAGACCATACTCGTGGCGACGTACGAGGGCGAGCACAACCACTCCCAGCCCCCGCCG>Traes_7AL_48C81DE03 Org_Taestivumearly-release CDS: Traes_7AL_48C81DE03.1 (1 of 169) PF03106 - WRKY DNA -binding domain (WRKY) (PAC:31765470)

CTTTCGAGCTTCCCATGGCCGACGGCNNNNNNNNNNNNNNNNNNNNNNNNNNNNNNNNNNNNNNNNNNNNNNNNNNNNNNNNNNNNNNNNNNNNNNNNNNNNNNNNNNNNNNNNGAGACGAAGCCAGAGAAGAAGGCGGTGACGGAGACGCAGGTGAAGGAGGTGAGCAAGTCGGGGCCGAAGGAGATAGAGAAAGAGGTGAAGGTGAAAGTGGAGAAGGAGAACGAAAATGTGGAGATCGAGGCGACGCTGCGGCCGACCGGAGCTGGGACGGAGGCTCCCCCCATCCTTGCGGTGCCGATGTTGGCCGTGCCGTGCTTCATAGCTCCTCCAGGGTTTGCGGGCCAGTTTGCAATGAGCCATCAAGCAGCTTTGGCAAGTGTTACAGCTCAGGCACATATACAATTGCAATCACCAGCCTCATCTGCATATTCAGAAGGGCTGCCGAGTCCATTTCCACATCCTATAACACCTAAAGCTATTCGGCCACTGCAGCAAGCACCATCAGTAACTCAAGGAAGTATTGGAAGACCAATTGCAGAGAGGCCATCCTCATCTGAGTCAAAATTGCAACATCATGCAGCTGTAAATATTGTGGGTGATGGCTTCAATTGGAGGAAATATGGTCAGAAGCAAGTGAAAAGTAGTGATAATTCTAGGAGCTACTATAGATGCACAAATTCAAGTTGTTTGGCCAAAAAGAAAGTTGAGCATTGTCCTGATGGCCGTGTTATAGAAATCATATATAGAGGAGCACACAGTCATGAACCACCACAGAAGACCAGATTTGTAAAAGAGAGGTCGCTTCATATTTATGTTCCCCCCATAGGTGATGGAACTCTGCAACTTGTGAACACTGAAATTGTGGAATCCCGTACACCGACATGTAAATTAAACCAGAGTGCTGCCATCGAAAATTCTGAACAGCAGCTCTTTTGCTCAAGTGATTGTGAAGGGGATGTTGGTAACAAGTCAGAAGATGAACATCGTAGTGCAGAGTCGCAGCCAAAGCGAAGGATAGTTGAAGCCACAACATCTAACTTAACTCCAGTTCTCCGAACTGTCAGGGAGAGAAAGATTATCGTGCAGGCTGGGAAGATGAGCGATGGGTACAGATGGCGTAAGTATGGGCAGAAAATTGTGAAGGGAAATCCAAACCCCAGTTCTAATTGCGAAGCTAGCAAT

>Traes_7AL_48C81DE031 Org_Taestivumearly-release CDS: Traes_7AL_48C81DE031.1 (1 of 169) PF03106 - WRKY DNA -binding domain (WRKY) (PAC:31765472)

CTTTCGAGCTTCCCATGGCCGACGGCNNNNNNNNNNNNNNNNNNNNNNNNNNNNNNNNNNNNNNNNNNNNNNNNNNNNNNNNNNNNNNNNNNNNNNNNNNNNNNNNNNNNNNNNGAGACGAAGCCAGAGAAGAAGGCGGTGACGGAGACGCAGGTGAAGGAGGTGAGCAAGTCGGGGCCGAAGGAGATAGAGAAAGAGGTGAAGGTGAAAGTGGAGAAGGAGAACGAAAATGTGGAGATCGAGGCGACGCTGCGGCCGACCGGAGCTGGGACGGAGGCTCCCCCCATCCTTGCGGTGCCGATGTTGGCCGTGCCGTGCTTCATAGCTCCTCCAGGGTTTGCGGGCCAGTTTGCAATGAGCCATCAAGCAGCTTTGGCAAGTGTTACAGCTCAGGCACATATACAATTGCAATCACCAGCCTCATCTGCATATTCAGAAGGGCTGCCGAGTCCATTTCCACATCCTATAACACCTAAAGCTATTCGGCCACTGCAGCAAGCACCATCAGTAACTCAAGGAAGTATTGGAAGACCAATTGCAGAGAGGCCATCCTCATCTGAGTCAAAATTGCAACATCATGCAGCTGTAAATATTGTGGGTGATGGCTTCAATTGGAGGAAATATGGTCAGAAGCAAGTGAAAAGTAGTGATAATTCTAGGAGCTACTATAGATGCACAAATTCAAGTTGTTTGGCCAAAAAGAAAGTTGAGCATTGTCCTGATGGCCGTGTTATAGAAATCATATATAGAGGAGCACACAGTCATGAACCACCACAGAAGACCAGATTTGTAAAAGAGAGGTCGCTTCATATTTATGTTCCCCCCATAGGTGATGGAACTCTGCAACTTGTGAACACTGAAATTGTGGAATCCCGTACACCGACATGTAAATTAAACCAGAGTGCTGCCATCGAAAATTCTGAACAGCAGCTCTTTTGCTCAAGTGATTGTGAAGGGGATGTTGGTAACAAGTCAGAAGATGAACATCGTAGTGCAGAGTCGCAGCCAAAGCGAAGGATAGTTGAAGCCACAACATCTAACTTAACTCCAGTTCTCCGAACTGTCAGGGAGAGAAAGATTATCGTGCAGGCTGGGAAGATGAGCGATGGGTACAGATGGCGTAAGTATGGGCAGAAAATTGTGAAGGGAAATCCAAACCCCAGTTCTAATTGCGAAGCTAGCAAT

>Traes_7DL_B09854286 Org_Taestivumearly-release CDS: Traes_7DL_B09854286.1 (1 of 169) PF03106 - WRKY DNA -binding domain (WRKY) (PAC:31767242)

GCCGTCAGCGTCAACGTCGGCCCCGACCAGGCGGAGTGCACGTCCGTCCACGAGCCGTGCAACAGCAAGCGCGTCCGCGCCGACGAGTGCAAGGCCAGCAGGGTATCCAAGCTGTATGTCCACGCCGATCCCTCGGACCTCAGCCTCGTCGTGAAGGATGGGTACCAATGGCGAAAGTACGGGCAGAAGGTGACCAAGGACAACCCTTGCCCGAGGGCCTACTTCCGGTGCTCGTTCGCGCCGTCGTGCCAGGTGAAGAAGAAGGTGCAGCGCAGCGCCGAGGACAGGACCGTCCTGGTCGCCACGTACGAGGGCGAGCACAACCACGCACAGCCGCCGAAGATGCAAGGCTCTGGCGGTAGGAAGTCCGCCNCCCAGCAACAGCCGAAGCAGCAATCGATGACGGAGGCGGGATCGGCCGCGGACAGGAAGAACCTGGCGGAGCAGATGGCAGCGACGCTGACGAGGGACCCAGGATTCAAGGCGGCGCTCGTCTCCGCGCTCTCCGGCCGGATCCTCGAGCTCTCGCCGTCCGATAGTTGA

>Traes_7DL_F849918EA Org_Taestivumearly-release CDS: Traes_7DL_F849918EA.2 (1 of 169) PF03106 - WRKY DNA -binding domain (WRKY) (PAC:32005351)

CTCGCCGCGGCCGGCAGCAGCGGCCGCTTCGCCGTCAGCGTCACCGTCGGCCCTGACCAGGCGGAGTGCACGTCCGTCCACGAGCCCTGCAATAGCAAGCGCGTCCGCGCCGACGAGTGCAAGGCCAGCAGGGTCTCCAAGCTCTACGTCCACGCAGACCCCTCCGACCTCAGCCTCGTGGTGAAGGATGGGTACCAATGGCGGAAGTACGGGCAGAAGGTGACCAAGGACAACCCGTGTCCGAGGGCCTATTTCCGGTGCTCGTTTGCGCCGTCGTGCCATGTGAAGAAGAAGGTGCAGCGCAGCGCCGAGGACAAGGCCGTGCTTGTGGCCACGTACGACGGCGACCACAACCACGCACCGCCGCCCAAGCAACAAGGCTCGGGTGGCAGGAAGAGCGGCGANCGCAGCCGCCGTACGCGTATCAGCTGCGCCGGTGCTTGTCCAGCAGCAGCGGAAGCAAGAACCTTCGACGGCGGAGCAGGTGGACGATAG

>Traes_7DS_24C563960 Org_Taestivumearly-release CDS: Traes_7DS_24C563960.1 (1 of 169) PF03106 - WRKY DNA -binding domain (WRKY) (PAC:31926597)

ATGTTGGCCGTGCCGTGCTTCATAGCTCCTCCAGGGTTTGCGGGCCAGTTTGCAATGAGCCATCAAGCAGCTTTGGCAAGTGTTACAGCTCAGGCACATATACAATTGCAATCACCAGCCCCATCTGCATATTCAGAAGGGCTGCCGAGTCCATTTCCACATCCTATAACACCTAAAGCTATTCGGCCACTGCAGCAAGCACCATCAGTAACTCAAGGAAGTGTTGGAAGACCGATTGCAGAGAGGCCATCCTCATCTGAGTCAAAATTGCAACATCATGCAGCTGTAAATATTGTGGGTGATGGCTTCAATTGGAGGAAATATGGTCAGAAGCAAGTGAAAAGTAGTGATAATTCTAGGAGCTACTATAGATGCACAAATTCAAGTTGTTTGGCCAAAAAGAAAGTTGAGCATTGTCCTGATGGCCGTGTTATAGAAATCATATATAGAGGAACACACAGTCATGAACCACCACAGAACACCAGATTTGTAAAAGAGAGGTCGCCTCATATTTATGTCCCCCCCATAGGTGATGGAACTCTGCAACTTGTGAACACTGAAATTGTGGAATCCCGTACACCGACATGTAAATTAAACCAGAGTGCTGCCATCGAAAATTCTGAACAGCAGCTCTTTTGCTCAAGTGATTGTGAAGGGGATGTTGGTAACAAGTCAGAAGATGAACATCGTAGTGCAGAGTCGCAGCCAAAGCGAAGGATAGTTGAAGCCACAACATCTAACTTAACTCCAGTTCTCCGAACTGTCAGGGAGCAAAAGATTATCGTGCAGGCTGGGAAGATGAGCGATGGGTACAGATGGCGTAAGTATGGGCAGAAAATTGTGAAGGGAAATCCAAACCCCAGGTGTGTGTTGCTATTGCTTACCCATGGTTACTTCAGTGAATCTATTAAGCATTAA

Amino acid sequences of 100 *TaWRKYs*

>Traes_1AL_0404BC790 Org_Taestivumearly-release peptide: Traes_1AL_0404BC790.1 (1 of 5) PTHR31221:SF7 - WRKY TRANSCRIPTION FACTOR 24-RELATED (PAC:31987126)

MVSGAAPPPPPESGAGSSSGVGREETKGKGSARGRGSRKASRPRFAFQTKSENDVLDDGYRWRKYGQKAVKNSAFPRSYYRCTHHTCNVKKQVQRLAKDTSIVVTTYEGVHNHPCEKLMEALNPILRQLQFLSQL*

>Traes_1AL_4E924201A Org_Taestivumearly-release peptide: Traes_1AL_4E924201A.1 (1 of 9) PTHR32096:SF18 - DISEASE RESISTANCE PROTEIN-LIKE-RELATED (PAC:31938855)

MRDLFWLSPGEQGDLSDVVRASLHPPHQLPTPAADEEEEDEYSSLLLEGGGGGGGLVVGHGDEQLGMVAMMMGGNNSSRPPSSDHHVISLHSPPATTYTRPHPEPLAGMLRRPGFEREGDMVVGPPPEIGDRLQHMSIAHHPRVPTAMKPRKSQSKKVVCIPAPTAAPGASGRHSTSGEVVPSDLWAWRKYGQKPIKGSPYPRGYYRCSSSKGCPARKQVERSRTDPNMLVITYTSDHNHPWPTQRNALAGSTRPSSSSAAAAKIAASSSSSLAAAAARNSSNTNVDVDCAGAHHQLKQESDLDLFADMDALSVFSSIDKIQEDDSKQQLFDPFSSGFCDYI*

>Traes_1AL_F64E07A92 Org_Taestivumearly-release peptide: Traes_1AL_F64E07A92.1 (1 of 5) PTHR31221:SF23 - WRKY TRANSCRIPTION FACTOR 23-RELATED (PAC:31871735)

MKEGKREKKPRGSRVAFATKSAVDHLDDGYRWRKYGQKAVKNSSFPRSYYRCTAAQCGVKKLVERSQQDPSTVVTTYEGRHAHPSPIATHRGSRMLMATGVDTVYSLDVLQHQHHGFFPAGTDVYGRMYALPSTDASVVAHRSSEYGGMQVHAGVLPDAVMSYEHVHR*

>Traes_1AS_1432A2F79 Org_Taestivumearly-release peptide: Traes_1AS_1432A2F79.1 (1 of 169) PF03106 - WRKY DNA -binding domain (WRKY) (PAC:31973080)

GAGDDEHRGEKKIKISARVSSGRIGFRTRSEVEILDDGFKWRKYGKKAVKNSPNPRNYYRCSAEGCGVKKRVERDRDDPRYVVTTYDGVHNHATPGAAAQYYCYSPPRSSPPAAYSAAGLLQF*

>Traes_1BL_46340D685 Org_Taestivumearly-release peptide: Traes_1BL_46340D685.1 (1 of 169) PF03106 - WRKY DNA -binding domain (WRKY) (PAC:31924766)

MAMRPMSEMSPPPAPSDQRDAVIEELRKGSQLAEFLRQQVELIPEDSRRDAALANVSDITTALASSLSVLQSEREQYYCSSSSSDAGHASGASGGGGVRNGVVARTRNRKAKHRRGTYGEELPIKEILTKAPENDRFHWRKYGEKKILHADFPRLYYRCGYSDEHKCPAKKYVQQQNSSDPPMFLVTLINDHTCDTLFPDEDQDQPPSSSSSANNSQMLDFSKASLSSAVGVSRLKEEEDADMSVTVPSYNYTYDELSSSSLPFLSPKQWEMEMEVKSLFRRHSGDGN*

>Traes_1BL_9AFA4B870 Org_Taestivumearly-release peptide: Traes_1BL_9AFA4B870.1 (1 of 169) PF03106 - WRKY DNA -binding domain (WRKY) (PAC:31740471)

MLASDGAAGAVVVPDGGPGGTAHSVSVSSTSSEAGVGGGGAVEDEAGKCKKEEGEGDDESKEAAADGEADKTKKGAAKGKGAAKAKGEKRPRQARFAFMTKSEVDHLEDGYRWRKYGQKAVKNSPFPRCRSYYRCTTQKCPVKKRVERSYQDAAVVITTYEGKHTHPIPATLRGANHLLAAHAHAHGGHGLIHPGMFRMPAPPGAFRPGDALGSFLQQQHAAMQHQQQVAAAGMAMRQANAMASSHMQQAPPADRGLAAAMAGGTTGNSTHTVSSSSGTDPLRMEHLMAQDYGLLQDMLMPPSFAHSDGATNSNNNAHNRH*

>Traes_1DL_46428511F Org_Taestivumearly-release peptide: Traes_1DL_46428511F.1 (1 of 5) PTHR31221:SF23 - WRKY TRANSCRIPTION FACTOR 23-RELATED (PAC:31894510)

DGSADHGSCRSNEKEKKKKGKGEKKAHGSRVAFATKSEVDHLDDGYRWRKYGQKAVKNSSFPRSYYRCTATRCGVKKLVERSQQDPSTVVTTYEGRHGHPSPVATHRGPRMLMATGANTAYALAALQHQQHCFFSAGADVYAPLVAHRLSEHGGMQFHADLLPDAVMGYQQGYR*

>Traes_1DL_5BAB0B6BC Org_Taestivumearly-release peptide: Traes_1DL_5BAB0B6BC.1 (1 of 5) PTHR31221:SF7 - WRKY TRANSCRIPTION FACTOR 24-RELATED (PAC:32010216)

MVSGVTGAAAPESGAGSSSGVGREETKGKGSARARGSRKASRPRFAFQTKSENDVLDDGYRWRKYGQKAVKNSAFPRSYYRCTHHTCNVKKQVQRLAKDTSIVVTTYEGVHNHPCEKLMEALNPILRQLQFLSQL*

>Traes_1DL_DFE1721E0 Org_Taestivumearly-release peptide: Traes_1DL_DFE1721E0.1 (1 of 169) PF03106 - WRKY DNA -binding domain (WRKY) (PAC:31871499)

IFPNGDAAQVRDVAAAGAPVALRPKRRCHRGAPEGLSACGVPPAAGGAHPGGQPXRRDAALANVSDITTALASSLSVLQSEKEQYSSSSSSYDPGHASGPSGGGMRNGPVARSRNRKAKHRRGTYGEELPIKEILTEAPENDRFHWRKYGEKKILHADFPRLYYRCGYSDEHKCPAKKYVQQQNSRDPPMFLVTLINDHTCDTLFPDEDQDQPPSSSSSPNNSQVLDFSKASLSSAVGVSRLKKEEDADMSVTVPSYNYTYDELSSSSLPFLSPKQWEMEMEVKSLFRRHSGDGS*

>Traes_2AL_15A7BB684 Org_Taestivumearly-release peptide: Traes_2AL_15A7BB684.1 (1 of 4) PTHR31282:SF15 - WRKY TRANSCRIPTION FACTOR 11-RELATED (PAC:31798439)

MAVDLMGCYTPRRADDQLAIQEAATAGLRSLELLVSSLSGAAPSKAPQQHPQQPFGEIADQAVSKFRKVISILDRTGHARFRRGPVQSPTPPPPAPVAPPPPPPRPLAVVEPARPAPLTVVAPVSVAAPVPLPQPQSLTLDFTKPNLTMSGATSVTSTSFFSSVTAGEGSVSKGRSLVSAGKPPLSGHKRKPCAGAHSEANTTGSRCHCSKRRKNRVKTTVRVPAVSAKIADIPPDEYSWRKYGQKPIKGSPYPRGYYKCSTVRGCPARKHVERALDDPAMLVVTYEGEHRHSPGPMPMQMAPSPVPIPMPMGAPVAVASVSAGNGHV*

>Traes_2AL_409AB7647 Org_Taestivumearly-release peptide: Traes_2AL_409AB7647.1 (1 of 3) K18835 - WRKY transcription factor 2 (WRKY2) (PAC:31829399)

MDGHTHLAVEWKDQSPGADCSMLPSFLTDPFPADPLVEDCDGGNDGSEGAGFERHGLSVAVGSPQEEGKPATPHFGQRSSSSSSLSERMQARAGFSVAKLSMPGSEYSGAQSPYLTIPPGLSPASLLESPVFLSNAMGQSSPTTGKLLMLGDTNNNNNTRLEPPSIEDRPGAFSFKPLDLKSSQYTAEGKKGSLPNSQHPSAPSRDVPVKTETNIQTTTRGAIPPGHLNQAQFNNGQDLMKCSYHDCNNKRNRLAADRTTAGGDNNDGPPVTAADSEAAKGDYPAAVATAAPAEDGYSWRKYGQKQVKHSEYPRSYYKCTHPSCQVKKKVERSHEGHVTEIIYKGTHNHPRPAAQGRRPAGGAQVHPFNDAQMDAPADNNNNGYGNAGGSQPNAEARSLWHAGVAVQDWRGDGLEATSSPSVPGELCDSSASMQVHDGAARFESPEGGVDVTSAVSDEVDGDDRVAHGSMSQGQGAADTTEGDELESKRRKLESCAIDMSTASRAVREPRVVIQTTSEVDILDDGYRWRKYGQKVVKGNPNPRSYYKCTHPGCSVRKHVERASHDLKSVITTYEGKHNHEVPAARNGGHGSSAASGGTGASQLSHARRAEPPSVQDGLMRLGGCGAPFGLPPRDPLGPMSNYPYSLGGGHAXXLPMPSGLGAVEGLKLPMLSPSLHSVFRQRQAMETAAGFRVPKGEVKDEAAGAGAGAGGGAAAAAAYPQTMMSRLPLGHRM*

>Traes_2AL_434E9F101 Org_Taestivumearly-release peptide: Traes_2AL_434E9F101.1 (1 of 16) PF03106//PF10533 - WRKY DNA -binding domain (WRKY) // Plant zinc cluster domain (Plant_zn_clust) (PAC:31854913)

MMTMDLIGGYGRADEQVAIQEAAAAGLCGMEHLILQLSRTGTSESSPVGSSEAPEQQVDCREITDMTVSKFKKVISILNHRTGHARFRRGPVVAQSQGPAVSEPAPVRASSSRSMTLDFTKASSGYGNDAGFSVSAASSSFMSSVTGDGSVSNGRGGGSSLMLPPPPSASCGKPPLASSAASTGAGAGQKRKCHDHAHSENVAGGKYGASGGRCHCSKRRKSRVRRMTRVPAISSKAAEIPADDFSWRKYGQKPIKGSPYPRGYYKCSTVRGCPARKHVERDPSDPSMLIVTYEGEHRHTPADQEPLAPLPEL*

>Traes_2AL_B1270662B Org_Taestivumearly-release peptide: Traes_2AL_B1270662B.1 (1 of 169) PF03106 - WRKY DNA -binding domain (WRKY) (PAC:31953214)

MEGGSQLGACLPSLYALDPYASPPLLAPLPNQHKLHQMPLVLQEQPGNHGVMFSSDHGGGLYPLLPGIPFCHSAAACEKPTGFAPLGGTGEAGTSAARAANEFASTTTTTTASCHGPSSWWKGAEKGKMKVRRKMREPRFCFQTRSEVDVLDDGYKWRKYGQKVVKNSLHPRSYYRCTHSNCRVKKRVERLSEDCRMVITTYEGRHTHTPCSDDDAAGDHTGSCAFTSF*

>Traes_2AS_0186B9E4F Org_Taestivumearly-release peptide: Traes_2AS_0186B9E4F.2 (1 of 169) PF03106 - WRKY DNA -binding domain (WRKY) (PAC:31765715)

MVAPSSSILKASRSLIDNFDVFHEQDLAQLAAQVAQKKELREKQGAGLHHKIGPQLAFSKYSILDQVDNSSSFSLATSVLTPQHVSSSVGAASMQGQTLPSHTGSGSVNTGPTGVLQVLQDSSTTLDSINTGSAGVLEALQGSSITLDKPADDGYNWRKYGQKAVKGGKYPKSYYKCTLNCPVRKNVEHSADGRIIKIIYRGQHCHEPPSKRFKDCGDLLNELDEFNDAKDPSTRSQLGCQGYYGKPITPNGTMVDGLLPTKEEGDEQLSSLSDIREGDGEIRTVDGDVGDADANERNAPGQKIIVSTTSDVDLLDDGYRWRKYGQKVVRGNPHPRSYYKCTYQGCDVKKHIERSSEEPHAVITTYEGKHTHDVPESRNRSQGTGQHHCKEQTYSEQPAASFCSSSEKRKYGTAILNDLAF*

>Traes_2AS_1AFFE8DA6 Org_Taestivumearly-release peptide: Traes_2AS_1AFFE8DA6.1 (1 of 169) PF03106 - WRKY DNA -binding domain (WRKY) (PAC:31960625)

DEYRGRNNDKRSRSLVTVVPHYDGHHWRKYGQKNINGREHARHY

>Traes_2AS_6269D889E Org_Taestivumearly-release peptide: Traes_2AS_6269D889E.1 (1 of 169) PF03106 - WRKY DNA -binding domain (WRKY) (PAC:31962353)

MESVEGNGAGRGNLQLVVSELCRVQELVRQLELHLHAPDASIDLCRALTAEIFALTDRSIGFVAAAHFPDAPTTPSSTSSSLSGVSDQPFRTNTKKRKATTRWTSQVRVSAAGGAEWPGDDGHSWRKYGQKDILGARHPRAYYRCTHRNSQGCPATKQVQRADQDPALFDIVYHGQHTCRPGGGGGGXXXXXXXXXXPQPARGEPAAEPQSRPDRGRGPRRPEQHLSLTAVDGLACGVRLQRWPDHVTVPGAGRRVHGVAAGRRPPGGGVGAHSRVGPEHGLPFRVRPDLRPWCAQLLHVRTTNRASKWNYMTLPSPCLSLVRG*

>Traes_2AS_C407071E4 Org_Taestivumearly-release peptide: Traes_2AS_C407071E4.2 (1 of 169) PF03106 - WRKY DNA -binding domain (WRKY) (PAC:31882682)

MQFAGCSVVVLPILRSQVFRGLKAWWTLKIVNKNFFQVEPSPTTGSLSMAAIMHKSAHPDMPSPRDKSIRAHEDGGSRDFEFKPHLNSSSQSVAPAMSDLKKHEHSMQNQSMNPSSSSSNMVNENRPPCSRESSLTVNVSAPNQPVGMVGLTDSMPAEVGTSEPQQMNSSDNAMQEPQSENVADKSADDGYNWRKYGQKHVKGSENPRSYYKCTHPNCEVKKLLERAVDGLITEVVYKGRHNHPKPQPNRRLAGGAVPLNQGEERYDGAAAADDKSSNALSNLANAVNSPGMVEPVPVSVSDDDIDAGGGRSYPGDDGTEEEDLESKRRKMESAGIDAALMGKPNREPRVVVQTVSEVDILDDGYRWRKYGQKVVKGNPNPRSYYKCTSTGCPVRKHVERASHDPKSVITTYEGKHNHEVPAARNAIHEMSAPPMKNVVHQINSNMPSSIGGMMRACEVRNFSNQYSQAAETDNVSLDLGVGISPNHSDATNQMQSTGPDQMQYQMQPMASMYSNMRHPSMAMPTVQGNSAGRMYGSREDKGSEGFTFRATPMDHSANLCYSGAGNLVMGP*

>Traes_2AS_D0C21ADB5 Org_Taestivumearly-release peptide: Traes_2AS_D0C21ADB5.1 (1 of 3) PTHR31221:SF25 - WRKY TRANSCRIPTION FACTOR 45-RELATED (PAC:31912163)

MAGASSHDHLHHHGQAAGNNTGGGGGLGQGLFSGSKQEDPSESKDGGDDRAGSSSQGGGEADVVVGKKKGEKRERRPRFAFQTRSQVDILDDGYRWRKYGQKAVKNNNFPRSVNPCPNFSYDTLTC*

>Traes_2BL_6B75B32E3 Org_Taestivumearly-release peptide: Traes_2BL_6B75B32E3.1 (1 of 4) PTHR31282:SF15 - WRKY TRANSCRIPTION FACTOR 11-RELATED (PAC:31983429)

MAVDLMGCYTPRRADDQLAIQEAATAGLRSLELLVSSLSGAAPSKAPQQHPQQPFGEIADQAVSKFRKVISILDRTGHARFRRGPVQSAPPPPPPPAPVAPPPPPPLTVVAPVSVAAPLPQPQSLTLDFTKPNLTMSGATSVTSTSFFSSVTAGEGSVSKGRSLVSAGKPPLSGHKRKPCAGAHSEANTTGSRCHCSKRRKNRVKTTVRVPAVSAKIADIPPDEYSWRKYGQKPIKGSPYPRGYYKCSTVRGCPARKHVERALDDPAMLVVTYEGEHRHSPGPMPMQMAPSPMPMPMGAPVAVASVSAGNGHV*

>Traes_2BL_A5BFA97B9 Org_Taestivumearly-release peptide: Traes_2BL_A5BFA97B9.1 (1 of 169) PF03106 - WRKY DNA -binding domain (WRKY) (PAC:31892717)

MEGGSQLGACLPSLYALDPYASPPLLAPSPNQHKLHQLPPVLQEQPGVHGVMFSSDHGGGLYPLLPGIPFCHSAAACEKHTGFAPLGSTGEAGTLAARQGNEIASATTTTTASCHGPSSWWKGAEKGKMKVRRKMREPRFCFQTRSEVDVLDDGYKWRKYGQKVVKNSLHPRSYYRCTHSNCRVKKRVERLSEDCRMVITTYEGRHTHTPCSDDDVGGDHTGSCAFTSF*

>Traes_2BS_380EC4D1E Org_Taestivumearly-release peptide: Traes_2BS_380EC4D1E.1 (1 of 169) PF03106 - WRKY DNA -binding domain (WRKY) (PAC:31924920)

MAAGQWSGIGDGGGLWAPPALDSLFPDDQPSPAASALGFFGGSLAQLPSPPPLCGTALLGYPQDNFDVFHEQDLAQLAAQVAQKKELREKQGAGLHHKIGPQLAFSKYSILDQVDNSSSFSLATSVLTPQHVSSSVGAASMQGQTLPSHTGSGSVNTGPTGVLQVLQDSSTTLDSINTGSTGVLEALQGSSITLDRPADDGYNWRKYGQKAVKGGKYPRSYYKCTLNCPVRKNVEHSADGRIIKIIYRGQHCHEPPSKRFKDCGDLLNELNDFNDAKEPSTKSQLGCQGYYGKPITPNGMMTDVLLPTKEEGDEQLSSLSDIREGDGEIRTVDGDDGDADANERNAPGQKIIVSTTSDVDLLDDGYRWRKYGQKVVRGNPHPRSYYKCTYQGCDVKKHIERSSEEPHAVITTYEGKHTHDVPESRNRSQATGQHHCKEQTYSEQSAASFCSSSEKRKYGTAILNDLAF*

>Traes_2BS_D435A8999 Org_Taestivumearly-release peptide: Traes_2BS_D435A8999.1 (1 of 169) PF03106 - WRKY DNA -binding domain (WRKY) (PAC:31876678)

MAAIMHKSAHPDILPSPRDKSIRAHEDGGSRDFEFKPHLNSSSQSLAPAMSDLKKHEHSMQNQSMNPSSSSSNMVNENRPPCSRESSLTVNVSAPNQPVGMVGLTDNMPAEVGTSEPQQMNSSDNAMQEPQSENVADKSADDGYNWRKYGQKHVKGSENPRSYYKCTHPNCEVKKLLERAVDGLITEVVYKGRHNHPKPQPNRRLAGGAVPSNQGEERYDGAAAADDKSSNALSNLANPVNSPGMVEPVPVSVSDDDIDAGGGRPYPGDDATEEDLESKRRKMESAGIDAALMGKPNREPRVVVQTVSEVDILDDGYRWRKYGQKVVKGNPNPRSYYKCTSTGCPVRKHVERASHDPKSVITTYEGKHNHEVPAARNATHEMSAPPMKNVVHQINSSMPSSIGGMMRACEARNFSNQYSQAAETDNVSLDLGVGISPNHSDATNQMQSSGPDQMQYQMQSMASMYGNMRHPSSMAVPTVQGNSAGRMYGSREEKGNEGFTFRATPMDHSANLCYSGAGNLVMGP*

>Traes_2BS_F3097F116 Org_Taestivumearly-release peptide: Traes_2BS_F3097F116.1 (1 of 3) PTHR31221:SF25 - WRKY TRANSCRIPTION FACTOR 45-RELATED (PAC:31818595)

ENYPIFFGTQPSSSTSNPYHFVAGASSHDHLHHHGQAAGNNTGGGGLNQGLFLGSKQEEPSESKDGGDDGAGSSSQGGGGEADVVVGKKKGEKRERRPRFAFQTRSQVDILDDGYRWRKYGQKAVKNNNFPRSYYRCTHQGCNVKKQVQRLSRDEGVVVTTYEGTHTHPIEKSNDNFEHILTQMQVYSGINNVSQTFGNQHMFQ*

>Traes_2DL_04535D371 Org_Taestivumearly-release peptide: Traes_2DL_04535D371.1 (1 of 6) PTHR31221:SF8 - WRKY TRANSCRIPTION FACTOR 57-RELATED (PAC:31903007)

MAGVECGGGDWPFSAEEAYADSSALLAEIGWAAGFVDDGCAGELLPPLDPPPATPTGSMEGAGASSSSTDDGATREAADADGRPAAATEAASKPAPALAPGKTMKKQKRARQPRFAFMTKTEIDHLEDGYRWRKYGQKAVKNSPFPRSYYRCTNNKCTVKKRVERCSDDPSVVITTYEGQHCHHTVTFPRGAGAATLASQMAFSAHHHHLMYNDLPALHSPTTQNPLFSVPAMSSSLLQPLHCNRQELQLASYTTQASSISSPGSVPAVDKGLLDDMVPPAMRHG*

>Traes_2DL_362A1F535 Org_Taestivumearly-release peptide: Traes_2DL_362A1F535.1 (1 of 169) PF03106 - WRKY DNA -binding domain (WRKY) (PAC:31942345)

NIFFLGRCRTQDSSVVTKNMKSLEDGQTWRKYGQKEIQNSKHSKAYFRCTHKYDQQCMARRQAQRCDDDP

>Traes_2DL_4F9F8F1F0 Org_Taestivumearly-release peptide: Traes_2DL_4F9F8F1F0.1 (1 of 169) PF03106 - WRKY DNA -binding domain (WRKY) (PAC:31853252)

MEGGSQLGACLPSLYALDPYASPPLLAPLPNQHKLHQLPLVLQEQPGNHGVMFSSDHGGGLYPLLPGIPFCHSAAACEKSTGFAPLGGTGEAGTSAARAGNEFASATTTTTASCHGPSSWWKGAEKGKMKVRRKMREPRFCFQTRSEVDVLDDGYKWRKYGQKVVKNSLHPRSYYRCTHSNCRVKKRVERLSEDCRMVITTYEGRHTHTPCSDDDAGGDHTGSCAFTSF*

>Traes_2DL_F600B5FDF Org_Taestivumearly-release peptide: Traes_2DL_F600B5FDF.1 (1 of 4) PTHR31282:SF15 - WRKY TRANSCRIPTION FACTOR 11-RELATED (PAC:31752041)

CHCSKRRKNRVKTTVRVPAVSAKIADIPPDEYSWRKYGQKPIKGSPYPRGYYKCSTVRGCPARKHVERALDDPAMLVVTYEGEHRHSPGPMPMQMAPSPMPMPMGAPVAVASVSAGNGHV*

>Traes_2DS_97E3E7CFC Org_Taestivumearly-release peptide: Traes_2DS_97E3E7CFC.1 (1 of 169) PF03106 - WRKY DNA -binding domain (WRKY) (PAC:32007262)

MAAIMHKSAHPDILPSPRDKSIRAHEDGGSRDFEFKPHLNSSSQSLAPAMSDLKKHEHSMQNQSMNPSSSSSNMVNENRPPCSRESSLTVNVSAPNQPVGMVGLTDNMPAEVGTSEPQQMNSSDNAMQEPQSENVADKSADDGYNWRKYGQKHVKGSENPRSYYKCTHPNCEVKKLLERAVDGLITEVVYKGRHNHPKPQPNRRLAGGAVPSNQGEERYDGAAAADDKSSNALSNLANPVNSPGMVEPVPVSVSDDDIDAGGGRPYPGDDATEEEDLELKRRKMESAGIDAALMGKPNREPRVVVQTVSEVDILDDGYRWRKYGQKVVKGNPNPRSYYKCTSTGCPVRKHVERASHDPKSVITTYEGKHNHEVPAARNATHEMSAPPMKNVVHQINSNMPSSIGGMMRACEARNFTNQYSQAAETDTVSLDLGVGISPNHSDATNQMQSSGPDQMQYQMQSMASMYGNMRHPSSMAVPTVQGNSAGRMYGSREEKGNEGFTFRATPMDHSANLCYSGAGNLVMGP*

>Traes_2DS_AD8820C42 Org_Taestivumearly-release peptide: Traes_2DS_AD8820C42.1 (1 of 169) PF03106 - WRKY DNA -binding domain (WRKY) (PAC:31872073)

MARLPASHHQSTTSSPAPRDREISLITCSLPSFSSANRTCSSVSAAMESVEGNGTGRGNLQLVVSELCRVQELVRQLELHLHAPDASIDLCRALTAEIFALTDRSIGFVAAAHFPDAPTTPSSTSSSLSGVSDQPFRTNTKKRKATARWTSQVRVSAAGGAEGPGDDGHSWRKYGQKDILGAKHPXLLPLHPPQLAGLPRHQAGAARRPGPRALRRRLPRPAHLQANGR*

>Traes_2DS_F6FBC974C Org_Taestivumearly-release peptide: Traes_2DS_F6FBC974C.2 (1 of 169) PF03106 - WRKY DNA -binding domain (WRKY) (PAC:31836810)

MAAGQWSGIGDGGGLWAPPALYSLFPDEQPALGFYGGSLAQLPSPPPLLGYPQDNFDVFHEQDLAQLAAQVAQKKELRGKQGAGLHHKIGPQLAFSKYSILDQVDNSSSFSLATSVLTPQHVSSSVGAASMQGQTLPSHTGSGSVNTGPTGVLQVLQDSSTTLDSINTGSAGVLEALQGSSITLDRPADDGYNWRKYGQKAVKGGKYPKSYYKCTLNCPVRKNVEHSADGRIIKIIYRGQHCHEPPSKRFKDCGDLLNELDDFNDAKEPSTRSQLGCQGYYGKPITPNGTMVDGLLPTKEEGDEQLSSLSDIREGDCEIRIVDADVGDADANERNVPGQKIIVSTTSDVDLLDDGYRWRKYGQKVVRGNPHPR*

>Traes_3AL_140B829CB Org_Taestivumearly-release peptide: Traes_3AL_140B829CB.2 (1 of 1) PTHR31221:SF37 - WRKY TRANSCRIPTION FACTOR 71-RELATED (PAC:31942939)

MSSGGGGGGDQGRHGLYHQHGHGQLTRYDGAGGYELSNDDMESFFFSQPEGVGGGVRADEIAPYSSITSYLQGFLDPTGLARHLDVPAKHELSVDVRSHDQDSQGTGSAAGESAALLTPNSSVSFSSGGGDGEGKSRRSKKGRAQEPDDQEDGKDHEDGESSKTANNKPKKKAEKRPRLPRVSFLTKSEVDHLEDGYRWRKYGQKAVKNSPYPRSYYRCTTPKCGVKKRVERSYQDPSTVITTYEGQHTHHSPASLRGSAAHLFMPPGLHGLPPPHLIPPGVFHPELMSMMRMPYPSPNMHLLSVPPPPHHHPTSHPMAGTLQQYHFTDYALLQDLSTSTMPNNP*

>Traes_3AL_1B73D2C12 Org_Taestivumearly-release peptide: Traes_3AL_1B73D2C12.1 (1 of 169) PF03106 - WRKY DNA -binding domain (WRKY) (PAC:32002429)

PKILFNLNFIYYHCRNYYRCSTEGCSVKKRVERDKDDANYVVTMYEGVHNHASPGTIYYAAQDPASGRFFVTGTHQLAP*

>Traes_3AL_2297D6E18 Org_Taestivumearly-release peptide: Traes_3AL_2297D6E18.1 (1 of 169) PF03106 - WRKY DNA -binding domain (WRKY) (PAC:31939858)

MASPPPKGESFDFEDPRAQEAMGSASASYSAPGGVFGLSPPESSRRDSRKRRKDRPSWVKHTFTPHFDGHLWRKYGQKNIKDSVFPRLYYRCSYREDKQCLASKLVQQENHEDPPLFKVTYTYEHTCNTAPVPTPDVVAELPAPATGDALFLRFDSTGAGHRDAHRMEQERHYQQPAAPGWPSMMLSFDSNSQQHEQCTFPSELPPAASSSSFSTEGLPAPPSTTDGGGDGFSTWDSLRYGLNDHVHFGDNSYLPNSGNDGDDNY*

>Traes_3AL_3160E1F30 Org_Taestivumearly-release peptide: Traes_3AL_3160E1F30.1 (1 of 169) PF03106 - WRKY DNA -binding domain (WRKY) (PAC:31939797)

MDGYRWRKYGQKFIKNNPHPRSYYKCTSARCSAKKHVEKSTDDPEMLIVTYEGSHLHGPQTT

>Traes_3AL_4769A72F1 Org_Taestivumearly-release peptide: Traes_3AL_4769A72F1.1 (1 of 5) PTHR31221:SF7 - WRKY TRANSCRIPTION FACTOR 24-RELATED (PAC:31977027)

RSYYRCTHPTCNVKKQVQRLAKDTAIVVTTYEGVHNHPCEKLMEALGPILKQLQFLS

>Traes_3AL_67ECA2932 Org_Taestivumearly-release peptide: Traes_3AL_67ECA2932.1 (1 of 169) PF03106 - WRKY DNA -binding domain (WRKY) (PAC:31827594)

AASGKIAFRTRSEEEILEDGYKWRKYGKKSVKNSPNPRYPKRTNSFTFL*

>Traes_3AL_AB2BAE660 Org_Taestivumearly-release peptide: Traes_3AL_AB2BAE660.1 (1 of 169) PF03106 - WRKY DNA -binding domain (WRKY) (PAC:31851405)

MSSYSSLLSVSPGEQIGGYADGGDHDDMAAAANYLSSFCFDFGEEYYSLAEAATASYPLHAQQQQQPPTQADSHHSGKAASTTSSSQGLDNINTSLTSSDARSKGSKIAFKTRSEVEVLDDGYRWRKYGKKMVKNSPNPRNYYRCSSEGCRVKKR

>Traes_3AL_DED8A29EC Org_Taestivumearly-release peptide: Traes_3AL_DED8A29EC.1 (1 of 5) PTHR31221:SF7 - WRKY TRANSCRIPTION FACTOR 24-RELATED (PAC:31876237)

SGENDGEAGGSGSGNKEKAKGSAGRSGKKKASKPRFAFQTRSENDILDDGYRWRKYGQKAVKNSSNPRYGSSN*

>Traes_3B_0C5417706 Org_Taestivumearly-release peptide: Traes_3B_0C5417706.2 (1 of 169) PF03106 - WRKY DNA -binding domain (WRKY) (PAC:31922736)

MQTQSRLIMNPNGGVTGYEPAATDEQHEAVLRELAHGHELTAHLQAEALRALHGQGQTEATAALILQEVSRAFTVCINIMGGSAPAATPTTPPPDAAAVVVTGAASARRPRDDGVPRKVTVTSSPYSDGYQWRKYGQKRIMRTSFPRCYYRCCYHRERSCPATKLVQQQPPQQHSDGDQTMYTVTYVHEHTCHNMAPAEPEAAARSSTPDPLGFSAGMQPRQQQRGGAGLDRGSKEELERQALVSSLACVLQGHHQSYTGSGAGTPDGSPSQGRVGDGPSASGLSLDTSDDLGLDVMDYGVTDALYFAASSSYGPGGDGMIP*

>Traes_3B_41047D5E6 Org_Taestivumearly-release peptide: Traes_3B_41047D5E6.2 (1 of 169) PF03106 - WRKY DNA -binding domain (WRKY) (PAC:31782323)

MAVAGAGAAYRFHPHGAGSMAFPRPPGSGCPYSSGAPLSSPAFGGATGPGVLQQQLDVLDYLSDDGGVPGTVGAPLPVEAAVVPDVGYCDHTRAAAVAASGKIAFRTRSEEEILDDGYKWRKYGKKSVKNSPNPRNYYRCSTEGCSVKKRVERDKDDANYVVTMYEGVHNHASPGTVYYASQDPASGRFFVTGTHHLAP*

>Traes_3B_8B0D448D8 Org_Taestivumearly-release peptide: Traes_3B_8B0D448D8.1 (1 of 169) PF03106 - WRKY DNA -binding domain (WRKY) (PAC:31890613)

MRGSNMLSSSGSNKRALQQDCSGGSHAQEHTKRKSRIGMRTDYTYAPYHDGFQWRKYGQKVIRGNAFPRCYYRCTYHQDHGCSASKHVEQHNSADPPLFRVVYTNDHTCSGAAASASDYMASSMQIQQIADASLRKADTEAERPPRPQQPRSGGSYAAAIKEEKDAIVSSLLTVIRGSCDVVKSDTAHEGYSSASLATNCYAMSSPSVAGGSREGSSSSSVSPVVLPAPDDMGLGLDFMVESHWFEPLDLGWFVE*

>Traes_3B_990298FF5 Org_Taestivumearly-release peptide: Traes_3B_990298FF5.1 (1 of 1) PTHR31221:SF1 - WRKY TRANSCRIPTION FACTOR 1-RELATED (PAC:31865868)

MTTSSSGSVETSANSRPGSFSFGSASFTEMLGGSAAAGGASGYKALTPPSLPLSPSLMSPSSFFSMPAGMNLADFLDSPVLLTSSIFPSPTTGAFGSQFNWRPEAPTPSAAEQGGKEEQRQPYSDFSFQTAPANSEEAARATMTTSLQPPVAVASQGEEAYTGQQQQAWGYGQQQEGMNASAANPASFSAPALQATSSEMAPAGAYRQTHSQRRSSDDGYNWRKYGQKQVKGSENPRSYYKCTFPNCPTKKKVETSLEGQITEIVYKGTHNHAKPLNTRRGSGGGAAAAQVLQIGGDASENSFGGMVTTPENSSASFGDDDNGVSSPRSGNVGRNDNDDDEPDSKRRRDSGDGEGINMAGNRTVREPRVVVQTMSDIDILDDGYRWRKYGQKVVKGNPNPRSYYKCTTVGCPVRKHVERASHDLRAVITTYEGKHNHDVPAARGSAALYRPAPRAADSTASTGHYLNPQPSAMAYQASAAPNVAGTQQYAPRPDGFGGQNPGSFGFNGNFGFSGAGFDNPTASYMSQHQEQQRQNDAMHASSAKEEPREEDMFFQNSQY*

>Traes_3B_B8BF316B8 Org_Taestivumearly-release peptide: Traes_3B_B8BF316B8.2 (1 of 2) PTHR31221:SF42 - WRKY TRANSCRIPTION FACTOR 49-RELATED (PAC:31892659)

MEEMGEESSRYPWQDYDLGFGEELMRELLDQTTTAPTPSPAAMAAGAASADNSSSSDKGIGDEEEGAAGRRESMENRLMSTVYSGPTLSDIESALSFTGAGAGDPLDGRSKYHYSPSSPVYVPRIYANNNHIQFSVDASSIWTSVWKFRVFSPEKVLGKMENKYTMKIKSCGNGLADDGYKWRKYGQKAIKNSPNPRSYYRCTNPRCNAKKQVERAVDEPDTLVVTYEGLHLHYTYSHFLQQQTNPPPAAAAAASSSKKPKLHPTAGAITVTDSHHGSTPV

>Traes_3B_CDA5ADD75 Org_Taestivumearly-release peptide: Traes_3B_CDA5ADD75.1 (1 of 169) PF03106 - WRKY DNA -binding domain (WRKY) (PAC:31744736)

MSDLGLPASSPFFIRAPSYRGETSDSSLFRSLRHVSFSLPLPSATKASLSSPRRLSICMDKGHLGGGGGGGGLLALDASPRQLGFLNLLSPAPFHRSMEADDGGGGGGGGRGRRSIEVDFFSDEKKNMKKSRASAGADAEDHKDQASAAGLAIKKEDLTINLLPGNNTRSDRSMVVDDDGASRADQDRNGRNTGELAVIQAELSRMNEENQRLRGMLTQVNNSYHALQMHLVALMQQRTQMPPVQPQQPPTHEDGKNESAIVPRQFLGLGPSGASADVAEEPSNSSTEVGSPRRSSSNGNEDPERGENPDGPSTAGWLPGRGMSQQQQQQLGAAAKGHDQQAQEATMRKARVSVRARSEAPIIADGCQWRKYGQKMAKGNPCPRAYYRCTMATGCPVRKQVQRCAEDRTILITTYEGTHNHPLPPAAMAMASTTSAAASMLLSGSMPSADGAGLMSSNFLARTVLPCSSSMATISASAPFPTVTLDLTHAPPGAPNAMPLNVARPHAPGQFHVPMPGGGMAPAFAMPPHMLYNQSKFSGLQMSSDSVDAGQF

>Traes_3B_D6F86ABC3 Org_Taestivumearly-release peptide: Traes_3B_D6F86ABC3.2 (1 of 169) PF03106 - WRKY DNA -binding domain (WRKY) (PAC:31895081)

MALATPTAVVLELMTMGQQSAAHLGDLLRAASPPVRAEHQALAAEILRCCDRVIAAVSAGASDKKRKMTDPGATTCHPPAAAMPSKRRVRGAEAHREVHADTTADGFVWRKYGQKDINGSNHPRLYYRCAFRGEGCAATRRVQRSQEEPAAFVIAYYGEHTCGAAFSQQRAEPQPPTVVDSGSNAWGVFGAVDRNRGSPLMPSLAAEHDVRRHGEAPRDTSQRWSSPSSSSSYSEVELGASPVEGFLDGNFDWEWETVVNSLRFGDLLH*

>Traes_3B_F45FCFE62 Org_Taestivumearly-release peptide: Traes_3B_F45FCFE62.1 (1 of 169) PF03106 - WRKY DNA -binding domain (WRKY) (PAC:31799212)

MEERCALATELAQVLDTVRQLEAHMGVKGGADGGETCRTLVSSMRSSVDRSIHIAMSSCCVVLGAPESPPSAGGXSPRSGGSDQAADSPCRGAHAAGQSKKRKTQPKWSTQVRVNSVEDVGPLDDGISWRKYGQKDILGAKYPRAYFRCTHRHTQGCYASKQVQRAHGDPLLFDVVYHGNHTCAQGKHYNSQRPQPVASGEHRRSGSPWD*

>Traes_3DL_2551BF2C1 Org_Taestivumearly-release peptide: Traes_3DL_2551BF2C1.1 (1 of 169) PF03106 - WRKY DNA -binding domain (WRKY) (PAC:31968771)

LCEVHFSFNRVTVTSSPYSDGYQWRKYGQKRIMRTSFPRCYYRCCYHRERSC

>Traes_3DL_678D51EAD Org_Taestivumearly-release peptide: Traes_3DL_678D51EAD.1 (1 of 169) PF03106 - WRKY DNA -binding domain (WRKY) (PAC:31874088)

HAAGQSKKRKTQPKWSTQVRVNSVEDVGPLDDGFSWRKYGQKDILGAKYPRAYFRCTHRHTQGCYASK

>Traes_3DL_7456F61A3 Org_Taestivumearly-release peptide: Traes_3DL_7456F61A3.1 (1 of 169) PF03106 - WRKY DNA -binding domain (WRKY) (PAC:31793891)

MRTDYTYAPYHDGFQWRKYGQKVIRGNAFPRCYYRCTYHQDHGCSASKHVEQH

>Traes_3DL_DF0D3F3FE Org_Taestivumearly-release peptide: Traes_3DL_DF0D3F3FE.1 (1 of 169) PF03106 - WRKY DNA -binding domain (WRKY) (PAC:31886335)

EEEILDDGYKWRKYGKKSVKNSPNPRNYYRCSTEGCYVKKRVERDKDDANYVVTMYEGVHNHASPGTVYYAAQDPASGRFFVTGTHHLAP*

>Traes_4AL_234E1CDF6 Org_Taestivumearly-release peptide: Traes_4AL_234E1CDF6.1 (1 of 2) PTHR31221:SF16 - WRKY TRANSCRIPTION FACTOR 62-RELATED (PAC:31959259)

MHAPIFLVELKCSFIIYVIDELYLYVLVCRGSGEGERGHHGDEEEQQQAAWAEEAAGVQPLVMPEDGYQWKKYGQKFIKNIQKIRSYFRCRDKRCGAKKKVEWQPGDPSLRIVYDGAHQHGSPASNGGGQDGDGAANRYDLSTQYFGGAGAPTPQTR*

>Traes_4AL_2EEECCC4B Org_Taestivumearly-release peptide: Traes_4AL_2EEECCC4B.1 (1 of 169) PF03106 - WRKY DNA -binding domain (WRKY) (PAC:31746115)

MANFEMSHQQALAQVTAQAVHSQYTVGSQADYSLPFSSATTSALTSQFINSSANVTSMKETATLPLHTVNDNLKSNEVSQGFQTSALTVDKPADDGYNWRKYGQKAVKGGEYPRSYYKCTQASCPVKKKVEHSAYGQITQIIYRGQHNHQRPPKRRSKDGGNLLNEDDFPENRDALTRSEPGSQDHSGKVEVSNDGITGPSVSKRRGGGDQSSGSSDTEEDNDEAGDDNGDAGIVNANKRHVPAPAQRIIVQTTSEIDLLDDGYRWRKYGQKVVKGNPHPRSYYKCTYQGCDVKKHIERCSQDPTAVITTYEGKHSHDVPAARSSVAAAASANASSSISLLHRGQKAASSSQRVLPRAALHTSDSSLQLKEENEIT*

>Traes_4AL_98B1C762B Org_Taestivumearly-release peptide: Traes_4AL_98B1C762B.2 (1 of 16) PF03106//PF10533 - WRKY DNA -binding domain (WRKY) // Plant zinc cluster domain (Plant_zn_clust) (PAC:31891223)

MEEVEEANRMAVESCHRVLGLLAQTQDPAQLRSIALGTDEACAKFRKVVSLLGNGNGNGNEGGGTHHPRAKLVSRRQTPGFLSQKSFLDNNTPVVVLNSAHPSTSSAQVYPSSRNSILDSSQAAHPIGGPPKLVQPLSAHFQFGDSSRYNQFQQQHQHQQQKMRAEMFKRSNSGINLKFDSPSGTGTMSSARSFMSSLSMDGSVASLDAKSSSFHLIGGPAMSDPVNAQQAPRRRCSGRGEDGNGKCAATGRCHCSKRSRKLRVKRTIKVPAISNKIADIPPDEYSWRKYGQKPIKGSPHPRGYYKCSSVRGCPARKHVERCVDDPSMLIVTYEGEHNHTRMPTQSAQA*

>Traes_4AL_C2A825B6D Org_Taestivumearly-release peptide: Traes_4AL_C2A825B6D.1 (1 of 169) PF03106 - WRKY DNA -binding domain (WRKY) (PAC:31792629)

MAQVSFAGAGDDKHRSEKTIKISARVSAGRIGFRTRSEVEILDDGFKWRKYGKKAVKNSPNPRNYYRCSAEGCGIKKRVER

>Traes_4AS_0DA136E0E Org_Taestivumearly-release peptide: Traes_4AS_0DA136E0E.1 (1 of 169) PF03106 - WRKY DNA -binding domain (WRKY) (PAC:32002393)

MAEDGQKEIQNSTHPRSYYRCTHKSDQGCNAKRQAQICETHPIKYDITYYGEHTCKPPSNTPMIIVAASDDRAENLVSFAPTFPHLAPGSAPALTTCSAPRPIHSCRRTSSPPSWDRPGGRPRWWGRCRTTAGVG*

>Traes_4AS_70DF607CC Org_Taestivumearly-release peptide: Traes_4AS_70DF607CC.1 (1 of 169) PF03106 - WRKY DNA -binding domain (WRKY) (PAC:31888413)

MSPVPSPNQSHLLGHGSRKEKRMRKVDTFAPHNDGHQWRKYGEKKINNCNFPRYYYRCTYKDNMNCPATKQIQQKDHSDPPLYQVTYYNEHSCNSAFLALTPTEFQLQTASGKAVSICFDSSGAQEPGANASSPSSSAAPRATPSESKNKPLALRSEALSSWAPGVVEQKTACADLQSCSTECQDAYISEDIDAGRFGSIRFFHFL*

>Traes_4BL_EFEC50B26 Org_Taestivumearly-release peptide: Traes_4BL_EFEC50B26.2 (1 of 169) PF03106 - WRKY DNA -binding domain (WRKY) (PAC:31827464)

MSHQQALAQVTAQAVHSQYTVGSQADYSLPFSSATSALTSQFIKSSANVTSMKETATLPLHTVNDNLKSNEVSQGFQTLALTVDKPADDGYNWRKYGQKAVQGGEYPRSYYKCTHASCPVKKKVEHSAYGQITQIIYRGQHNHQRPPKRRSKDGGNLLNEDDFPENRDALTRSEPGSQDHSGKVEVSNDGITGLSMSKRRDGGDQSSGSSDREEDNDEAGDDNGDAGIVNANKRHVPVPAQRIIVQTTSEIDLLDDGYRWRKYGQKVVKGNPHPRMVFEKVDSSFHPGILDLSHLHTPCCILVVKGRGVGR*

>Traes_4DS_3BE557D5C Org_Taestivumearly-release peptide: Traes_4DS_3BE557D5C.4 (1 of 16) PF03106//PF10533 - WRKY DNA -binding domain (WRKY) // Plant zinc cluster domain (Plant_zn_clust) (PAC:31752217)

MEEVEEANRMAVASCHRVLGLLAQTQDPAQLRSIALGTDEACAKFRKVVSLLGNGNEGGGTHPRAKLVSRRQTPGFLSQKSFLDNNTPVVVLNSAHPSTSSAQVYPRNSSILDSQPAHPIGGPPKLVQPLSAHFQFGDSSRYNQFQQHQHQQQKMRAEMFKRSNSGINLKFDSPSGTGTMSSARSFMSSLSMDGSVASLDAKSSSFHLIGGPAMSDPVNAQQAPRRRCSGRGEDGNGKCAATGRCHCSKRSRKLRLKRTIKVPAISNKIADIPPDEYSWRKYGQKPIKGSPHPRGYYKCSSVRGCPARKHVERCVDDPSMLIVTYEGEHNHTRMPTQSAQA*

>Traes_4DS_CFC487CE5 Org_Taestivumearly-release peptide: Traes_4DS_CFC487CE5.2 (1 of 2) PTHR31221:SF16 - WRKY TRANSCRIPTION FACTOR 62-RELATED (PAC:31752593)

MYTMIHESYISMYRWLICRGNEGGERGHHGDEEEQQQGAWAEAAGGQPLVMPEDGYQWKKYGQKFIKNIQKIRSYFRCRDKRCGAKKKVEWQPGDPNLRVVYDGAHQHGSPSSNGGGQDADGAANRYDLSTQYFGGAGAPTPQTQ*

>Traes_4DS_DC3C9DC42 Org_Taestivumearly-release peptide: Traes_4DS_DC3C9DC42.3 (1 of 169) PF03106 - WRKY DNA -binding domain (WRKY) (PAC:31879714)

MAAHEASAGGGEGARCTPPRPALSLPPRSAVESFFGSGATAASFAETSPGPFTLAAALFPDMPSSAFHGSFTQLLVGAMGSPAAPPSPPSPFAVPPGLSPTALVGPFPPTGNFEMSHQQALAQVTAQAVHSQYTVGSQADYSLPFSSATTSALTSQFINSSANVTSMKETATLPLHTVNDNLKSNEVSQGFQTSALTVDKPADDGYNWRKYGQKAVKGGEYPRSYYKCTQASCPVKKKVEHSAYGQITQIIYRGQHNHQRPPKRRSKDGGNLLNEDDFPENRDTLTRSEPGSQDHSGKVEVSNDGITGSSISKRRDGGDQSSGSSDREEDNDEAGDDNGDAGIVNANKRHVPAPAQRIIVQTTSEIDLLDDGYRWRKYGQKVVKGNPHPRSYYKCTYQGCDVKKHIERCSQDPTAVITTYEGKHSHDVPAARSSVAAAASANASSSISLLHRGQKAASSSQRVLPRAALHTSDSSLQLKEENEIT*

>Traes_4DS_FE38A59D0 Org_Taestivumearly-release peptide: Traes_4DS_FE38A59D0.1 (1 of 169) PF03106 - WRKY DNA -binding domain (WRKY) (PAC:31768080)

MALDSVPSYPSDLGSSGRATRTQQRIRKEERTWTADTYAPYDDGHQWRKYGEKKLSNSNFPRFYYRCTYKTDLKCPATKQVQQKDMSDPPLFTVTYFNHHSCNTTSRPIGSAPDTTEQSSSRRAVSICFGSHATGEQPTFLTSPGTLQSPASTTNQQNDRGAYGHQFQWTDTSPSAGDAPVKMETDSLAGTGASSGAASGHALSRTLLPIGQSRCIEYFQFL*

>Traes_5AL_06A6F9328 Org_Taestivumearly-release peptide: Traes_5AL_06A6F9328.2 (1 of 6) PTHR31221:SF8 - WRKY TRANSCRIPTION FACTOR 57-RELATED (PAC:31972770)

MKPTTAGKKGQKRARQQRFAFVTKSEVDHLEDGYRWRKYGQKAVKNSPFPRSYYRCTNSKCTVKKRVERSSEDPSVVITTYEGQHCHHQTSFQRGXXXXXXXXXXXXXXXXXXXXXXXXXXXXXXXXXXXXXXXXXXXXPSLQQLNGGDELRRSTSYSPMASATQTPSSLVPPDVSFDMGLLGDIVPPGVRNG*

>Traes_5AL_6FDB440FB Org_Taestivumearly-release peptide: Traes_5AL_6FDB440FB.1 (1 of 169) PF03106 - WRKY DNA -binding domain (WRKY) (PAC:31752743)

MAICRNYYRCTNSTNQGCPAKRTVQRNDDDGSDDGRPKYTVVYISEHSCKATESAAVPVILETTVRTDT

>Traes_5AL_7164FEAC3 Org_Taestivumearly-release peptide: Traes_5AL_7164FEAC3.1 (1 of 6) PTHR31429:SF3 - WRKY TRANSCRIPTION FACTOR 40-RELATED (PAC:31988149)

SNRNVGTGEAEHVDVDSPLSNGTCRRIKVKKVCTRIDPSDTSLVVKDGYQWRKYGQKVTRDNPSPRAYFRCAFAPSCPVKKKV

>Traes_5AL_A3653B781 Org_Taestivumearly-release peptide: Traes_5AL_A3653B781.1 (1 of 16) PF03106//PF10533 - WRKY DNA -binding domain (WRKY) // Plant zinc cluster domain (Plant_zn_clust) (PAC:31919715)

MQQRRRCAGKEDGSGRCATGSRCHCAKKRKLRIRRSIKVPAISNKVADIPADEFSWRKYGQKPIKGSPHPRGYYKCSSVRGCPARKHVERCVDDPAMLIVTYEGDHNHNHNQAAAAQPA*

>Traes_5AL_A39A63F43 Org_Taestivumearly-release peptide: Traes_5AL_A39A63F43.2 (PAC:31882179)

RTASGWICSERSCSYWTSDGVMIVLCASSNFVLASESQTFLRKGKEFRAENWTCRHGVLFCAVLDLDRAQFCRAYYRCTYQKTQGCAATKQ

>Traes_5AL_B4E8A3115 Org_Taestivumearly-release peptide: Traes_5AL_B4E8A3115.3 (1 of 1) PF00931//PF03106//PF12796 - NB-ARC domain (NB-ARC) // WRKY DNA -binding domain (WRKY) // Ankyrin repeats (3 copies) (Ank_2) (PAC:31838967)

MGDSALHLAARAGNVALVQKIFADCDPELVAELTDHQNQDGETALYVSAEMGHVEVVCEILKVCDLHSAFLKAHNSFDAFHIAAKQGHLVVLQELLKAFPALAMTTNSVNATALYTAALHRHIGIVNLLLDTDPRLARIARNNGKTALHIAARLGNVEVVVLLLNKDPATVFRIDRKGQTAVHMASKGHNAEILLELLKPDVSVIHLEDNKGDRPLHVATRKGKTIIVQTLISIEEIDINAINGAGETAFAIAEKLGNEELVNILREAGGVTAEEQVNPPKSIKRFKQTHDVQSQIKQKRRTNMHFHTIRKSSQKLHTEAPVCALADAMFRLPAKLDELLISHVHMLPRGAEDEIPLIKQDLEEIMAILQEHDHPGRAEDRAMTSKCLTKEVRELSYDMEDSVDQYVHAVDTKRRIVPRRKKYKITCRRGKTTARLPEKLKWRIWMANKIREFSVRSQEALQRYSLFNHPGAHGISTSATSTRHDVCFGSWYPTPCGELVGIDGHLNTLEAWLGKDGEQQLKVVSVVGSGGVGKTTLSKELYRRIRGQFECQAFVRTSRKPDIRRLLISLLSQVRPHQTPHTWKLHSLIADIRTHLHDKRYLIVIDDVWATQTWDIINRALPAGNLCSRILITTEVEDVALKCCGYDSRHVLMVKPLGYDDSSKLFFSTAFGLQYECPPELCDAAHNIVRKCAGSPLAMVTVASLLVSQIGKPEKWDYVNEIFGHGLSTYPSSEGMKQVLNLSYNNLPHYLKACVMYLSIYEEDYIIQKDDLVKQWIAEGLILATEEKDKEEISRRYFDELISSRMILPVYTNDNDDVLSCTLHHMVLDFIKHKSLEENFVIAIDHSQTTAPLADKVRRLSLHFGNAEATPPTNMRLSQVRTLAFFGVIECLPSVIEFRLLQVLILHLFGDDESVSFDLTGISELFRLRYLHVTCNATLEVPQTQMRGLQYLETLKIDARVSAVPSDIVHLPSLLHLSLPVGTNLPNGIDHMTSLCTLEYFDINVNSMENVHSLGELTNLQDLRLTCSTVPSSYLKSKIDSMGSILANLSNLRSVTLKSSGILESEPYSMIISCDGLSSVSSPPALLQRFEWLPRICTFSSIPKWISHLNKLCILKIGLRELVSNDVAALRGLPALTVLSLYVRAKPAEKIVFTRAGFLVLKCFKFRCSVPWLEFEVDAMPNLLKLKLSFDAHGVDQHRTIPVGMVHLTGLKEISAKIWGAGANERRAAKSALIDAIKMHSGCPTSSIQCLDGMFSGKDDNNSGIQEEEHLTLQKQYNIKEEDSKKQHDLPKDYMDVAYKQTSSSNNHRKSKRITQVRMQVRVGSVQDNSALEDGFSWRKYGQKDIIGSMHPRAYFRCTHRHVKGCPVTKQVQRTSTDPLLFDVVYHGEHTCLDSVGSPATSCGHVAGVEVMSRSRPGVGFVSQSQAACSSQVMSSEVVSGSGSTAGLWGDEIDMPDPDRDDTGISADYLGGYEFDVSAFFA*

>Traes_5AL_E566BD64E Org_Taestivumearly-release peptide: Traes_5AL_E566BD64E.1 (1 of 169) PF03106 - WRKY DNA -binding domain (WRKY) (PAC:31856791)

QKVVKGNPRPRSYYKCTAENCNVRKQIERASTDPRCVLTTYTGRHNHDPPGRGAGXXXXXXXRLLL*

>Traes_5AL_E644A6A0B Org_Taestivumearly-release peptide: Traes_5AL_E644A6A0B.1 (1 of 169) PF03106 - WRKY DNA -binding domain (WRKY) (PAC:31750780)

MQSQEKITPVKPVASRPFSSFTSFSKLLKDFTATGSAKITSPGETVIVRRPKVTRFAPPPSDLSAGVAASMLQDAGLDTTREKMVIDPEQVVSCDQMTTFHDINKPIHSVKTRLSYDGYNWRKYGQKQVKGSEFPRSYYKCTHPTCPVKRKVETTVDGQIAEIVYNGEHNHPQPHPPKKPASSASTEVVVPDAHGSNDAGAESQLGGCNLALVSDPVAAAFKSSCYYVDEFGNTSPVYHWNTSRKEKQSSIANGLTSGEAAPAFQSPTECGSSGDAAFRWRKYGQKAVNGNSFPRSYYRCSTARCNARKFVERSSDNSLVTTYEGKHNHVQLQ*

>Traes_5BL_0A3D332A8 Org_Taestivumearly-release peptide: Traes_5BL_0A3D332A8.1 (1 of 169) PF03106 - WRKY DNA -binding domain (WRKY) (PAC:31834520)

MESVDENGGSRLVVTELGYIKELVRQLDVNLGGCPDHCKRLAAQIFAVTERSIGMIRSGHFDSRKRSAAGLDSPPFSATPSPLSDVSGMPFHTNNKKRKTMEKRKHQVRVSSEGGGAETPVDDGHSWRKYGQKDILGAKHPRGYYRCTHRKSQGCAATKQVQRADEDPALFDVIYHGEHTCVHKTVAAAAAMVQPAEENPDARRHLQNLSTSLTVNTEGLTAGHQGCSTTTSFCFSSQAAGVLTMPQEHYPFSMPSTPENCFGQGASLSTSLEPSPVTSDSNRFSMSPFQAEWRARSEYDEVVSALVAAGTMPALTMEMEEETAFSLDEFEFDVSCFLA*

>Traes_5BL_17A712C94 Org_Taestivumearly-release peptide: Traes_5BL_17A712C94.1 (1 of 3) K18835 - WRKY transcription factor 2 (WRKY2) (PAC:31951792)

MAGTSDRGSLMEDWMAMPPTPSPRTLMSSFLNEDFSSGQFSNFFGEHVSNKPHDQSEKRGELVDLREQVPAQSATDTATPQKDFSLQPNSFNANQKSNPQGSLAERRASRAGFSIPKIDTSRVGSSTVIRSPIAIPPGLSPTTLLESPVFLYNAMAQPSPTTGKLFVASEANSTMPPDSTFSNDVFSFQPHSGPTSYSNVEKGYTVCHQNQSLSNIHQQGSSLQSSFTAAKDSADETIVKPKTSDSVFSDNHSSEEQEDDEGDQNEEYSSATNSNPAEDGYNWRKYGQKQVKSSEHPRSYYKCTHPDCPVKKKVERSQDGQITEIVYKSSHNHPLPPPNRRSGIPSLQINDPQVHLLEKPGLHTGVNTASLWENGKSECIQDMQGVEGRPAAGPPVSAYGDTSIMESQDAADVSSTLSNEIDRATQGTISLDCDVGEDETESKRRKLDALAAVTIPTATTTSSIDMVAAASRAVREPRVVVQTTSEVDILDDGYRWRKYGQKVVKGNPNPRSYYKCTHQGCSVRKHVERASHDLKSVITTYEGKHNHEVPAARNSGNAGSAPASAPQANLSHRRQEQAQGSYSQFGGASPFGSFGLPPRGHLGAAGNFHFGMAPPGMSMPPMPAARHPSMMQGYPGLMMQEGQMMQEGQMKAEPDQQSGFAASSAYQQMMGRPPFGPQM*

>Traes_5BL_8688F70C9 Org_Taestivumearly-release peptide: Traes_5BL_8688F70C9.1 (1 of 16) PF03106//PF10533 - WRKY DNA -binding domain (WRKY) // Plant zinc cluster domain (Plant_zn_clust) (PAC:31875786)

MAVDFVGRGHAPRGLALAGGQQQLAFHEAAAAGLSSLELLVSALSPRADCAPPPLGEIADQALSGFRRVIDILGRTGHARFRRGPVGGGAASLTPPPVSSPPRMPARPPAPAASQQLAPQKSLTLDFTKPSKTPAAAAAASVTSTSFFSSVTAGGEGSVSKGPSQLVSSGKPPLAAGTKRKQQQQQTPCASAAHSDAAAAAGGRCHCSKKRKHRVKYTTRVPAVSSRTADIPGDDYSWRKYGQKPIKGSPYPRCYYRCSTAKGCPARKHVERATDDPAMLIVTYEGDHRHDTLPPAAAN*

>Traes_5BL_90757F0CC Org_Taestivumearly-release peptide: Traes_5BL_90757F0CC.1 (1 of 6) PTHR31429:SF3 - WRKY TRANSCRIPTION FACTOR 40-RELATED (PAC:31986586)

MCCFWTMGTAPVCLDLMVGRPMDHEPSPVRCTGVRTEADVASSACDRAPPMTNDEAKILEAKLAQVSEENRKLTEMIAYLYGNQVSRQSPDGEGQQRARTAASPTPPAGKKRSQESMDASHSCDVEISNRNVGTGEAEHVDVDSPLSNGTCRRIKVKKVCTRIDPSDTSLVVKDGYQWRKYGQKVTRDNPSPRAYFRCAFAPSCPVKKKVQRSAEDSSVVEATYEGEHNHPHPTRAGELPSYAARSGGSVPCSISTNSSGPTITLDLTKNGGGVQVLDAGEAQPDMKKVCRAVASPEFQRALVEQMARELTGDQKFTDALAAAILRKLPDY*

>Traes_5BL_AEF9FE805 Org_Taestivumearly-release peptide: Traes_5BL_AEF9FE805.1 (1 of 16) PF03106//PF10533 - WRKY DNA -binding domain (WRKY) // Plant zinc cluster domain (Plant_zn_clust) (PAC:31966248)

MDGMVESNREAVQSCHKVLDLLSNPHGQLVPHKDLLEATGAAVAKFGSLASKIGNGNGGRQGHARFRQRIKKPMPLFDSNLFRDSPASAAAADAAAAAPPKTSSPGPSTSLQLFPRYQQMEASSSKDPVRIPAAQFPQRMVVENPSVGSNGPARGPPLHLVQPVSVAPPAGTPAPALPAAHLHFIQQQQSYQRFQLMHQMKLQSEMMKRGGHGDHQGGSTGAGKGVNLKFDGSNCTGSSSRSFLTSLSMEGSMASLDGSRSSRPFQLVSGSQTSSTPEMGLMQQRRRCIGKEDGSGRCATGSRCHCAKKRKLRIRRSIKVPAISNKVADIPADEFSWRKYGQKPIKGSPHPRGYYKCSSVRGCPARKHVERCVDDPAMLIVTYEGDHNHNRAAAAQPQPA*

>Traes_5BL_B9DD3E76F Org_Taestivumearly-release peptide: Traes_5BL_B9DD3E76F.1 (1 of 169) PF03106 - WRKY DNA -binding domain (WRKY) (PAC:31872762)

MDMEEQANAAATAAREGDLADVVARANSMPYSAGARRQAPPPPPPSAAARVMIPYEEERQRRPANVACGGGGGQVTFEAPPSTVVVDPYLLAAAGGYGLPQQQQHQHQQLLAFQISEHACCAAADSDDPMRISPPPPPPPPAPHHQMITSYCGMACTHIPYCRKNDVRKVVCIPAPPVMSNRAGGGGEVIPSDLWAWRKYGQKPIKGSPYPRGYYRCSSSKGCLARKQVERSRSDPNMLVITYTAEHNHPWPMQRNVLAGYARAHTHAAAKKQQKISSSSSADNAASSSSSNSFHVEQINPICGDQLPVSCKMPDSTATAGDGGGLLFEGIQPDEVFAELEELETDNNPMMTSANVYGSRGVSSNYEWHKF*

>Traes_5BL_D3C383CF5 Org_Taestivumearly-release peptide: Traes_5BL_D3C383CF5.1 (1 of 169) PF03106 - WRKY DNA -binding domain (WRKY) (PAC:31811544)

MADRRGDGMRQQPPYSSGHQERVFDGGGGSSGGPAFGNDYDPGSSYMSLLGSGVNPQQLLPAPPAWAVEEVAPPTINLTPQFSMANYVPTSSYQQQQHQTAASFVAPLAANLHPYQSSSSSYFQADPLPQWPPRAMAPSPSSSLLPRNFTLHQTPAYPHHHEQQMHMQLLRAAALGGPHAAPAPPIEQPAKDGYNWRKYGQKQLKDAESPRSYYKCTRDACPVKKIVERSFDGCIKEITYKGRHTHPRPPEPRRAGAEDVAAPGSAHQEDELSDDEDDGEEGHDIGSGAGGPAGQRVVRKHKIILQTPSEVDLLDDGYRWRKYGQKVVKGNPRPRSYISGW

>Traes_5BL_E294922A9 Org_Taestivumearly-release peptide: Traes_5BL_E294922A9.2 (1 of 3) PTHR31282:SF33 - WRKY TRANSCRIPTION FACTOR 15-RELATED (PAC:31742772)

MEGVEEANRAAVVSCKRLVARLSLSAGDPFRLAAVAAETEEAVSRFSKVVNILGNRVGHARARVGRRSSPAGDPIARCLLEYHPPPPVPYCPPASAPQLHGSSSSTPAPPTPLKQMAVPVAAAAAPCATDRDMFFQTPLLDLSGCSVTPASMPPCRSTAREFPQQQPAPPQKRMLEQQQRPASSDNKRFHFEPKPASEKPFHIEIPAARSGKEPEVITFSFDNSVCTSSAATSFFTNMSSQLISMSETSACAPASRKAAHKADDDGKCHCPKKKSVPVAYAIDLLKPREKRVVRMPAVSDKVADIPSDSYSWRKYGQKPIKGSPHPRGYYRCSSIKDCPARKHVERCRGDAGMLIVTYENDHNHAQPLDLATLTANSEV*

>Traes_5BS_C46781248 Org_Taestivumearly-release peptide: Traes_5BS_C46781248.1 (1 of 3) PTHR31221:SF22 - WRKY TRANSCRIPTION FACTOR 3-RELATED (PAC:32024774)

MSHQQALAQVTAQASHSPLRMFDHTEQTSFSAAATSSGALQNMSSAANVAEMSEMATTISNNEHAAFQSAEASHRYQVPAPVDKPADDGYNWRKYGQKVVKGSDCPRSYYKCTHPSCPVKKKVEHAEDGQISEIIYKGKHNHQRPPNKRAKDGSSLAAEQNEQSNDTASGLSGVRRDQEAVYGMSEQLSGLSDGDDKDDGESRPNEADDRESDCKRRNIQISSQKALTESKIIVQTTSEVDLLDDGYRWRKYGQKVVKGNPHPRSYYKCTFAGCNVRKHIERASSDPKAVITTYEGKHNHEPPVGRGSNQNGGNSNRSQQKGPNSMSSNQASHTRTDLGNVNQGQIGVLQFKREE*

>Traes_5DL_1733FB4DA Org_Taestivumearly-release peptide: Traes_5DL_1733FB4DA.1 (1 of 1) PF00931//PF03106 - NB-ARC domain (NB-ARC) // WRKY DNA -binding domain (WRKY) (PAC:31895273)

MLSQVRPHQTSHTWKLHSLIADIRTHLQDKRYLIVIDDVWATQTWDIVSRALPDGNLCSGVLITTEIDDVALKCGGYDSKYVLPMKPLGHDDSSKLFFRTAFGPQYECPPELSDVANNIIRKCAGFPLAVVTVAGLLVNQMGKPEQWDFVNKSLGYGLRKNPAPEGMKQVLNLSYNNLRLHLKACLMYLSIYEEDYIIQKNDLVKQWIAEGFIHATEEKDMVEISRICFDELISSRMIEPVHINDTGDVLSCTVHHMVLDFITHKSLEENFVTAIDHCQTTARLADKVRRLSLHFGNAEATPPTNMRLSQVRTLAYFGVIKCLPSIVEFGLLQILILHLWGDDDSISFDLTGISELFRLRYLHVTCNATLEVPQTQIRGLRYLETLKIDARVSAVPSDIVHLPGLLHLSLPVEINLPNGIGRMTSLCTLECFDISVNSVENVHSLGELTNLHDLRLTCSTVHSCYLTSKMDSMCSILTKLSNLRSLTLEPSSILDVGPSSMSISCDGLSSVSSPPACLQTFEWLPRICTFSSLPKWIGRLSKLCILKIGVRKLANNDFDILRGLPALTVLSLHIRTKPAKRILFNKIGFSVLKYFKFRCRAPWLEFEVDAMPNLLKLKLRFDAHGVDQHGTIPVGIVHLTGLKEMSAKIGGAGANDPDRRAAESALIDAIKMHPACPTLSIHCLDAMFSGEDDDIKKEDSIEHMTLQKQYDVKKEDSIEHMTLQKQYGIKKEDSIEHTTLQEQYDIKNEDLIEHMTLQIQNDIKKEDSYKQHGFLQKDYRKTLPKWSTQVRVSSLQDIEGHDDGFSWRKYGQKDILGSRNPRGYYRCTHHNTRGCQALKQLQATDGDPLLFNAIYVGNHTCTQGANSQPQPGYEQSSISVGDKAEGSIQRLEKMPPRRSKRSIQVRVRSMQDDYPADDGYSWSKYGQKDILGSKHPRGYYRCVHRPEKGCEATKQVQRSDSDTQLFDVVYHGEHTCAENVHSRGESARSLPHHVSVSAGVIPPATSESQVTYEAVSSGSTAGIHFMSPATSAGSQVTYESGSRSTTTGRFISPGMSESQVAYAEFWTWPNNVDFMLNSPINERLDLNADFVDETGPSDFD*

>Traes_5DL_2553A6C33 Org_Taestivumearly-release peptide: Traes_5DL_2553A6C33.1 (1 of 3) PTHR31282:SF33 - WRKY TRANSCRIPTION FACTOR 15-RELATED (PAC:31823877)

MPAVSDKVADIPSDSYSWRKYGQKPIKGSPHPRGYYRCSSIKDCPARKHVERCRGDAGMLIVTYENDHNHAQPLDLATLTANSEXXXXXXXXXXXXXXXXXXWNMPFSLTKSECK*

>Traes_5DL_32D78D06A Org_Taestivumearly-release peptide: Traes_5DL_32D78D06A.1 (1 of 6) PTHR31429:SF3 - WRKY TRANSCRIPTION FACTOR 40-RELATED (PAC:31917474)

ISNRNVGTGEAEHVDVDSPLSNGTCRRIKVKKVCTRIDPSDTSLVVKDGYQWRKYGQKVTRDNPSPRAYFRCAFAPSCRVKKKVQRSAEDSSVVEATYEGEHNHP

>Traes_5DL_46E3AC8D6 Org_Taestivumearly-release peptide: Traes_5DL_46E3AC8D6.1 (1 of 169) PF03106 - WRKY DNA -binding domain (WRKY) (PAC:31972964)

MADRRGDAMRQQPPYSSGHQERVFDGGGGPAFGNDYDTAGSSYMALLGSGVNPQQSLPPQQAWGVDEVTPPTINLTPQFSMANYAPTSSYQQHQTTASFVSPLAANLHPYPSSSSSSYFQADLPPQWPPRAMAPSPSSSLLPRNFTVHQTPAYPHHHEQQMHMQLLRAAALGGPHAAPAPPIEQPAKDGYNWRKYGQKQLKDAESPRSYYKCTRDACPVKKIVERSFDGCIKEITYKGRHTHPRPPEPRRAGAEDAAAPSSAVGAHQEDELSDDEDDGEEGHDIVSGAGGPAGQRVVRKHKIILQTPSEVDLLDDGYRWRKYGQKVVKGNPRPRSYYQKTSFIWGR

>Traes_5DL_4BA2CC560 Org_Taestivumearly-release peptide: Traes_5DL_4BA2CC560.1 (1 of 169) PF03106 - WRKY DNA -binding domain (WRKY) (PAC:31924879)

MQSQEKITPVKPVASRPFSSFTSFSKLLKDFTATGSAKITSPGETVIVRRPKATRFAPPPSDLSAGVAASMLQDAGLDTTREKMVIDPEQVVSCDQMTAFHDINKPIHSVKNRLSYDGYNWRKYGQKQVKGSEFPRSYYKCTHPTCPVKRKVETTVDGQIAEIVYNGEHNHPQPHPPKKPVSSASTEVVVPDAHGNNDAGAESQLGECNLALVSDPVAAAFKSSCDYVDEFGNTGPVYHCNTSPKEKQSSIANGLPSSGEAAPAFQPPTECRSSGDAAFRWRKYGQKAVNGNSFPRSYYRCSTARCNARKFVERSSDNSLVTTYEGKHNHVQLR*

>Traes_5DL_5C93510D5 Org_Taestivumearly-release peptide: Traes_5DL_5C93510D5.1 (1 of 169) PF03106 - WRKY DNA -binding domain (WRKY) (PAC:31747511)

MEAVHEGNGGGSGLVVTELSHIKELVKQLDVHLGGSPDLCKLLAQQIFAVTERSIGMIRSGHFNGPKRPAAGAGLDSPPLSPTPSPLSGVSNTPFKPNKKRKTSEKGGRQIRVSSAAGGADAPADDGRSWRKYGQKDILGAQHPRAYYRCTYQKTQGCAATKQVQRADEDPALFDVIYHGEHTCVHKTAAAAVQPAGQNPGAESLLQSLSSSLTVKTEGLTAAGAQGWSATTPFSFSSPAVSGMTPPEHHPFSTPSTPENCFVSMPTSLEPSPATSGSNHMCMTPFHAQSELQTMVSALVEATSMPAAGTEEAAFYTFQSDWSFDDSALDVNNFDVSALDVNNFDVSVFLA*

>Traes_5DL_A54ED44C9 Org_Taestivumearly-release peptide: Traes_5DL_A54ED44C9.1 (1 of 16) PF03106//PF10533 - WRKY DNA -binding domain (WRKY) // Plant zinc cluster domain (Plant_zn_clust) (PAC:31821176)

MDGMVESNREAVQSCHKVLDLLSNPHGQLVPHKDLVEATGAAVAKFGSLASKISSGNGRQGHARFRQRIKKPMPLFDSNLFRDSPASASAADAAAAAPKTSSPGPSTGLQLFPRYQQMEASSSKDPVRIPAAQFPQRMVVENPSVGSNGPPLQLVQPVSVAPPAGTPAPALPAAHLHFIQQQQSYQRFQLMHQMKLQSEMMKRGGHGDHQGGSTGAGKGVNLKFDGSNCTGSSSRSFLTSLSMEGSMASLDGSRSSRPFQLVSGSQTSSTPELGLMQQRRRCAGKEDGSGRCATGSRCHCAKKRKLRIRRSIKVPAISNKVADIPADEFSWRKYGQKPIKGSPHPRGYYKCSSVRGCPARKHVERCVDDPAMLIVTYEGDHNHNRAAAAQPQPA*

>Traes_5DL_C93641E43 Org_Taestivumearly-release peptide: Traes_5DL_C93641E43.1 (1 of 169) PF03106 - WRKY DNA -binding domain (WRKY) (PAC:31745499)

MESVDENGGSRLVVTELGYIKELVRQLDVNLGGCPDHCKRLAAQIFALTERSIGMIRSGHYDCRKRSAAGLDSPPFSATPSPLSDVSGMPFHNNKKRKTMEKRKHQVRVSSEGGGAETPVDDGHSWRKYGQKDILGAKHPRGYYRCTHRKSQGCAATKQVQRADEDPALFDVIYHGEHTCVHKTVATAAAAMAQPAEENPDARRHLQNLSTSLTVNTEGLTATAGHQGCGTTTSFCFSSQAAGVLTTPQEHYPFSMPSTPENCFGQRASLSTSLEPSPVTSDSNRFSMNPFQAEWRAQSEYDEVVSALVAAGTMPALAMEMEEETAFSLDEFEFDVSSFLA*

>Traes_5DL_E4A6D1889 Org_Taestivumearly-release peptide: Traes_5DL_E4A6D1889.2 (1 of 169) PF03106 - WRKY DNA -binding domain (WRKY) (PAC:31898234)

MDMKEQANAAATAAREGDLADVVARANAMAYSTGARRQAPPPPPSAAARVMIPYEEERQRRPANVACGGGEVTFEAPPSTVVVDPYLLAAAGGYGLPQQHQHQHQQLLAFQISEHACCAAADSDDPMRISPPPPQPAPHHQMITSYCGMACTHIPYCRKNDVRKVVCIPAPPVMSNRAGGGGEVIPSDLWAWRKYGQKPIKGSPYPRGYYRCSSSKGCLARKQVERSRSDPNMLVITYTAEHNHPWPMQRNVLAGYARAHTHAAAKKQQKISSSSSADNAASSSSINSFHVEQINPICVDQLPVSCKMPDSTATAGDGGGLLFEGIQPDEVFAELEELETHNNPVMTSANVYGSRGVSSNYEWHKF*

>Traes_6AL_BA4636569 Org_Taestivumearly-release peptide: Traes_6AL_BA4636569.1 (1 of 169) PF03106 - WRKY DNA -binding domain (WRKY) (PAC:31787421)

MHICMDQGSQLGMAYCLPNLSVPDHYYTTPVPLSPLQLPFHPKPLQMPFDQEEALMLSSDHCGLYPLPALPLGSGHSAGAPAIVCEKPTVGFMPNIGAEEVGTSVTARVGYEGATACNGYSSNTWWRGSTMLAGEKGKMKVRRKMREPRFCFQTRSDVDVLDDGYKWRKYGQKVVKNSLHPRSYFRCTHSNCRVKKRVERLSTDCRMVITTYEGRHTHPPCDDNSSSSGDNTTTCF*

>Traes_6AS_68775100B Org_Taestivumearly-release peptide: Traes_6AS_68775100B.1 (1 of 1) PTHR31282:SF1 - WRKY TRANSCRIPTION FACTOR 38-RELATED (PAC:31916438)

MGNIELPPDDGYTWRKYGQKDILGSRFPRSYYRCTHKNYYGCDAKKKVQRLDDDPFMYEVTYCGSHSCLTSTTPLLNFPTATATATATNSPTAATGSGLAPADHFMAPTEQAAVSTSMHLGVGWMPASFQGVVAGSGAGGGSSAGMQTSVSTAARDTDYPALDLADVMFNSGGSVGMDGIFSSHHRRDS*

>Traes_6AS_DA75BB1FD Org_Taestivumearly-release peptide: Traes_6AS_DA75BB1FD.1 (1 of 169) PF03106 - WRKY DNA -binding domain (WRKY) (PAC:31748920)

MDPWVSSQPSLSLDLHVGLPPMGHHQAAPMVALAKPKVLVEENFMQLKKDPEVAVLESELQRVSEENRRLGEMLREVASKYEALQGQFTDMVTAGAHAGGNNSHYNNQPSSASEGGSVSPSRKRKSEESLGTPPRPSQHQQQHYAGGLAYAAAPDQAECTSGEPCKRIREECKPVVSKRYVHADPSDLSLVVKDGYQWRKYGQKVTKDNPCPRAYFRCSFAPGCPVKKKVQRSAEDKTILVATYEGEHNHTQPPPSQPQQQNDGSGAGKNAGKPPQAPTATPHHPQQQHKQEAAAAAVSG

>Traes_6BL_B92FA1D38 Org_Taestivumearly-release peptide: Traes_6BL_B92FA1D38.1 (1 of 5) PTHR31429:SF1 - WRKY FAMILY TRANSCRIPTION FACTOR-RELATED (PAC:31835704)

MNDGCQWRKYGQKVAKGNPCPRAYYRCTVAPACPVRKQVQRCQEDMSILITTYEGTHN

>Traes_6BL_DD840863A Org_Taestivumearly-release peptide: Traes_6BL_DD840863A.1 (1 of 169) PF03106 - WRKY DNA -binding domain (WRKY) (PAC:31938936)

MHICMDQGSQLEMAYCLPNLSVPDHYYTTPVPLSPLQLPFHPKPLQMPFDQEEALMLSSDHCGLYPLPALPFGGHSAAAPATVCDKPTVGFMPSIGAEEVGTSVTARVGYEGATACNGYSSNTWWRGSTMLAGEKGKMKVRRKMREPRFCFQTRSDVDVLDDGYKWRKYGQKVVKNSLHPRSYFRCTHSNCRVKKRVERLSTDCRMVITTYEGRHTHPPCDDNSSSSGDNTTTCF*

>Traes_6BL_EEAA2A7E3 Org_Taestivumearly-release peptide: Traes_6BL_EEAA2A7E3.1 (1 of 9) PTHR32096:SF18 - DISEASE RESISTANCE PROTEIN-LIKE-RELATED (PAC:31766778)

MSSSPRGGGIKRRKNQARKVVCIPAPAAAVAGKTTGEVVPSDLWAWRKYGQKPIKGSPYPRGYYRCSSSKGCPARKQVERSRTDPNMLVITYTSEHNHPWPTQRNVLAGSTRSHYAKNSSSNTAAAASKNSKNCSRNQHKPVVKAE

>Traes_6DL_D4F2CDDDC Org_Taestivumearly-release peptide: Traes_6DL_D4F2CDDDC.1 (1 of 9) PTHR32096:SF18 - DISEASE RESISTANCE PROTEIN-LIKE-RELATED (PAC:31827697)

MCDYFLKRADGDQQAGDLTDIVRAGGAMPAGSTDPPSTATEWLQLPADPILFPLPQTSSSDGAGPSSADALGDPFSGLPDAFSTDYPSSSGSAAADFFDAVQDAMGVGMAKQVGFVDTTGCGGGGTTVGAGGGFLDMRNHHMFPGEMPMRVLSPYALMGGGAAKLGVPMAGHGQAAGPCAFDAVAGLQMSSSPRGGGIKRRKNQARKVVCIPAPAAAVAGKTTGEVVPSDLWAWRKYGQKPIKGSPYPRGYYRCSSSKGCPARKQVERSRTDPNMLVITYTSEHNHPWPTQRNVLAGSTRSHYAKNSSNTDAASSKNSKNSSRNQHKPVVKAESKDQSAATPAATSTTTTATTSTGNNTPPMAVKEEAEMERRIGGDTTATVGYYSDHLLQQMFSQSYRPMMPEEAGGYHHQDDFFADLTELDSDPVSLIFSTEYMEARPGKEKAAAKDDVDSLFMMDWAPASAAVTTSAGSALEQGDMGL*

>Traes_6DS_8F684013D Org_Taestivumearly-release peptide: Traes_6DS_8F684013D.1 (1 of 169) PF03106 - WRKY DNA -binding domain (WRKY) (PAC:31785825)

EGGSVSPSRKRKSEESLGTPPPSHQQHYAAGLAYAAAPDQAECTSGEPCKRIREECKPVVSKRYVHADPADLSLVVKDGYQWRKYGQKVTKDNPCPRAYFRCSFAPGCPVKKKVQRSAEDKTILVATYEGEHNHSQPPP

>Traes_7AL_48C81DE03 Org_Taestivumearly-release peptide: Traes_7AL_48C81DE03.1 (1 of 169) PF03106 - WRKY DNA -binding domain (WRKY) (PAC:31765470)

LSSFPWPTAXXXXXXXXXXXXXXXXXXXXXXXXXXXXXETKPEKKAVTETQVKEVSKSGPKEIEKEVKVKVEKENENVEIEATLRPTGAGTEAPPILAVPMLAVPCFIAPPGFAGQFAMSHQAALASVTAQAHIQLQSPASSAYSEGLPSPFPHPITPKAIRPLQQAPSVTQGSIGRPIAERPSSSESKLQHHAAVNIVGDGFNWRKYGQKQVKSSDNSRSYYRCTNSSCLAKKKVEHCPDGRVIEIIYRGAHSHEPPQKTRFVKERSLHIYVPPIGDGTLQLVNTEIVESRTPTCKLNQSAAIENSEQQLFCSSDCEGDVGNKSEDEHRSAESQPKRRIVEATTSNLTPVLRTVRERKIIVQAGKMSDGYRWRKYGQKIVKGNPNPSSNCEASN

>Traes_7AL_48C81DE031 Org_Taestivumearly-release peptide: Traes_7AL_48C81DE031.1 (1 of 169) PF03106 - WRKY DNA -binding domain (WRKY) (PAC:31765472)

LSSFPWPTAXXXXXXXXXXXXXXXXXXXXXXXXXXXXXETKPEKKAVTETQVKEVSKSGPKEIEKEVKVKVEKENENVEIEATLRPTGAGTEAPPILAVPMLAVPCFIAPPGFAGQFAMSHQAALASVTAQAHIQLQSPASSAYSEGLPSPFPHPITPKAIRPLQQAPSVTQGSIGRPIAERPSSSESKLQHHAAVNIVGDGFNWRKYGQKQVKSSDNSRSYYRCTNSSCLAKKKVEHCPDGRVIEIIYRGAHSHEPPQKTRFVKERSLHIYVPPIGDGTLQLVNTEIVESRTPTCKLNQSAAIENSEQQLFCSSDCEGDVGNKSEDEHRSAESQPKRRIVEATTSNLTPVLRTVRERKIIVQAGKMSDGYRWRKYGQKIVKGNPNPSSNCEASN

>Traes_7DL_B09854286 Org_Taestivumearly-release peptide: Traes_7DL_B09854286.1 (1 of 169) PF03106 - WRKY DNA -binding domain (WRKY) (PAC:31767242)

AVSVNVGPDQAECTSVHEPCNSKRVRADECKASRVSKLYVHADPSDLSLVVKDGYQWRKYGQKVTKDNPCPRAYFRCSFAPSCQVKKKVQRSAEDRTVLVATYEGEHNHAQPPKMQGSGGRKSAXQQQPKQQSMTEAGSAADRKNLAEQMAATLTRDPGFKAALVSALSGRILELSPSDS*

>Traes_7DL_F849918EA Org_Taestivumearly-release peptide: Traes_7DL_F849918EA.2 (1 of 169) PF03106 - WRKY DNA -binding domain (WRKY) (PAC:32005351)

LAAAGSSGRFAVSVTVGPDQAECTSVHEPCNSKRVRADECKASRVSKLYVHADPSDLSLVVKDGYQWRKYGQKVTKDNPCPRAYFRCSFAPSCHVKKKVQRSAEDKAVLVATYDGDHNHAPPPKQQGSGGRKSGXRSRRTRISCAGACPAAAEARTFDGGAGGR*

>Traes_7DS_24C563960 Org_Taestivumearly-release peptide: Traes_7DS_24C563960.1 (1 of 169) PF03106 - WRKY DNA -binding domain (WRKY) (PAC:31926597)

MLAVPCFIAPPGFAGQFAMSHQAALASVTAQAHIQLQSPAPSAYSEGLPSPFPHPITPKAIRPLQQAPSVTQGSVGRPIAERPSSSESKLQHHAAVNIVGDGFNWRKYGQKQVKSSDNSRSYYRCTNSSCLAKKKVEHCPDGRVIEIIYRGTHSHEPPQNTRFVKERSPHIYVPPIGDGTLQLVNTEIVESRTPTCKLNQSAAIENSEQQLFCSSDCEGDVGNKSEDEHRSAESQPKRRIVEATTSNLTPVLRTVREQKIIVQAGKMSDGYRWRKYGQKIVKGNPNPRCVLLLLTHGYFSESIKH*
