# Supplementary material for: Characterizing the Role of TaWRKY13 in Salt Tolerance
Source: Int J Mol Sci. 2019 Nov 14;20(22):5712. doi: 10.3390/ijms20225712 (PMC6888956; doi:10.3390/ijms20225712)
Supplement: Supplementary file 1 [file ijms-20-05712-s001.zip › Supplementary Table 3.docx]

>AT1G13960.1

MSEKEEAPSTSKSTGAPSRPTLSLPPRPFSEMFFNGGVGFSPGPMTLVSNMFPDSDEFRSFSQLLAGAMSSPATAAAAAAAATASDYQRLGEGTNSSSGDVDPRFKQNRPTGLMISQSQSPSMFTVPPGLSPAMLLDSPSFLGLFSPVQGSYGMTHQQALAQVTAQAVQANANMQPQTEYPPPSQVQSFSSGQAQIPTSAPLPAQRETSDVTIIEHRSQQPLNVDKPADDGYNWRKYGQKQVKGSEFPRSYYKCTNPGCPVKKKVERSLDGQVTEIIYKGQHNHEPPQNTKRGNKDNTANINGSSINNNRGSSELGASQFQTNSSNKTKREQHEAVSQATTTEHLSEASDGEEVGNGETDVREKDENEPDPKRRSTEVRISEPAPAASHRTVTEPRIIVQTTSEVDLLDDGYRWRKYGQKVVKGNPYPRSYYKCTTPGCGVRKHVERAATDPKAVVTTYEGKHNHDLPAAKSSSHAAAAAQLRPDNRPGGLANLNQQQQQQPVARLRLKEEQTT

>AT1G13960.2

MSEKEEAPSTSKSTGAPSRPTLSLPPRPFSEMFFNGGVGFSPGPMTLVSNMFPDSDEFRSFSQLLAGAMSSPATAAAAAAAATASDYQRLGEGTNSSSGDVDPRFKQNRPTAVLDLICNIVQGSYGMTHQQALAQVTAQAVQANANMQPQTEYPPPSQVQSFSSGQAQIPTSAPLPAQRETSDVTIIEHRSQQPLNVDKPADDGYNWRKYGQKQVKGSEFPRSYYKCTNPGCPVKKKVERSLDGQVTEIIYKGQHNHEPPQNTKRGNKDNTANINGSSINNNRGSSELGASQFQTNSSNKTKREQHEAVSQATTTEHLSEASDGEEVGNGETDVREKDENEPDPKRRSTEVRISEPAPAASHRTVTEPRIIVQTTSEVDLLDDGYRWRKYGQKVVKGNPYPRSYYKCTTPGCGVRKHVERAATDPKAVVTTYEGKHNHDLPAAKSSSHAAAAAQLRPDNRPGGLANLNQQQQQQPVARLRLKEEQTT

>AT1G18860.1

MDEAKEENRRLKSSLSKIKKDFDILQTQYNQLMAKHNEPTKFQSKGHHQDKGEDEDREKVNEREELVSLSLGRRLNSEVPSGSNKEEKNKDVEEAEGDRNYDDNEKSSIQGLSMGIEYKALSNPNEKLEIDHNQETMSLEISNNNKIRSQNSFGFKNDGDDHEDEDEILPQNLVKKTRVSVRSRCETPTMNDGCQWRKYGQKIAKGNPCPRAYYRCTIAASCPVRKQVQRCSEDMSILISTYEGTHNHPLPMSATAMASATSAAASMLLSGASSSSSAAADLHGLNFSLSGNNITPKPKTHFLQSPSSSGHPTVTLDLTTSSSSQQPFLSMLNRFSSPPSNVSRSNSYPSTNLNFSNNTNTLMNWGGGGNPSDQYRAAYGNINTHQQSPYHKIIQTRTAGSSFDPFGRSSSSHSPQINLDHIGIKNIISHQVPSLPAETIKAITTDPSFQSALATALSSIMGGDLKIDHNVTRNEAEKSP

>AT1G29280.1

MKRGLDMARSYNDHESSQETGPESPNSSTFNGMKALISSHSPKRSRRSVEKRVVNVPMKEMEGSRHKGDTTPPSDSWAWRKYGQKPIKGSPYPRGYYRCSSTKGCPARKQVERSRDDPTMILITYTSEHNHPWPLTSSTRNGPKPKPEPKPEPEPEVEPEAEEEDNKFMVLGRGIETTPSCVDEFAWFTEMETTSSTILESPIFSSEKKTAVSGADDVAVFFPMGEEDESLFADLGELPECSVVFRHRSSVVGSQVEIF

>AT1G29860.1

MDDHVEHNYNTSLEEVHFKSLSDCLQSSLVMDYNSLEKVFKFSPYSSPFQSVSPSVNNPYLNLTSNSPVVSSSSNEGEPKENTNDKSDQMEDNEGDLHGVGESSKQLTKQGKKKGEKKEREVRVAFMTKSEIDHLEDGYRWRKYGQKAVKNSPYPRSYYRCTTQKCNVKKRVERSFQDPSIVITTYEGKHNHPIPSTLRGTVAAEHLLVHRGGGGSLLHSFPRHHQDFLMMKHSPANYQSVGSLSYEHGHGTSSYNFNNNQPVVDYGLLQDIVPSMFSKNES

>AT1G30650.1

MCSVSELLDMENFQGDLTDVVRGIGGHVLSPETPPSNIWPLPLSHPTPSPSDLNINPFGDPFVSMDDPLLQELNSITNSGYFSTVGDNNNNIHNNNGFLVPKVFEEDHIKSQCSIFPRIRISHSNIIHDSSPCNSPAMSAHVVAAAAAASPRGIINVDTNSPRNCLLVDGTTFSSQIQISSPRNLGLKRRKSQAKKVVCIPAPAAMNSRSSGEVVPSDLWAWRKYGQKPIKGSPFPRGYYRCSSSKGCSARKQVERSRTDPNMLVITYTSEHNHPWPIQRNALAGSTRSSTSSSSNPNPSKPSTANVNSSSIGSQNTIYLPSSTTPPPTLSSSAIKDERGDDMELENVDDDDDNQIAPYRPELHDHQHQPDDFFADLEELEGDSLSMLLSHGCGGDGKDKTTASDGISNFFGWSGDNNYNNYDDQDSRSL

>AT1G55600.1

MSDFDENFIEMTSYWAPPSSPSPRTILAMLEQTDNGLNPISEIFPQESLPRDHTDQSGQRSGLRERLAARVGFNLPTLNTEENMSPLDAFFRSSNVPNSPVVAISPGFSPSALLHTPNMVSDSSQIIPPSSATNYGPLEMVETSGEDNAAMMMFNNDLPYQPYNVDLPSLEVFDDIATEESFYIPSYEPHVDPIGTPLVTSFESELVDDAHTDIISIEDSESEDGNKDDDDEDFQYEDEDEDQYDQDQDVDEDEEEEKDEDNVALDDPQPPPPKRRRYEVSNMIGATRTSKTQRIILQMESDEDNPNDGYRWRKYGQKVVKGNPNPRSYFKCTNIECRVKKHVERGADNIKLVVTTYDGIHNHPSPPARRSNSSSRNRSAGATIPQNQNDRTSRLGRAPPTPTPPTPPPSSYTPEEMRPFSSLATEIDLTEVYMTGISMLPNIPVYENSGFMYQNDEPTMNAMPDGSDVYDGIMERLYFKFGVDM

>AT1G62300.1

MDRGWSGLTLDSSSLDLLNPNRISHKNHRRFSNPLAMSRIDEEDDQKTRISTNGSEFRFPVSLSGIRDREDEDFSSGVAGDNDREVPGEVDFFSDKKSRVCREDDEGFRVKKEEQDDRTDVNTGLNLRTTGNTKSDESMIDDGESSEMEDKRAKNELVKLQDELKKMTMDNQKLRELLTQVSNSYTSLQMHLVSLMQQQQQQNNKVIEAAEKPEETIVPRQFIDLGPTRAVGEAEDVSNSSSEDRTRSGGSSAAERRSNGKRLGREESPETESNKIQKVNSTTPTTFDQTAEATMRKARVSVRARSEAPMISDGCQWRKYGQKMAKGNPCPRAYYRCTMATGCPVRKQVQRCAEDRSILITTYEGNHNHPLPPAAVAMASTTTAAANMLLSGSMSSHDGMMNPTNLLARAVLPCSTSMATISASAPFPTVTLDLTHSPPPPNGSNPSSSAATNNNHNSLMQRPQQQQQQMTNLPPGMLPHVIGQALYNQSKFSGLQFSGGSPSTAAFSQSHAVADTITALTADPNFTAALAAVISSMINGTNHHDGEGNNKNQ

>AT1G64000.1

MEGVDNTNPMLTLEEGENNNPFSSLDDKTLMMMAPSLIFSGDVGPSSSSCTPAGYHLSAQLENFRGGGGEMGGLVSNNSNNSDHNKNCNKGKGKRTLAMQRIAFHTRSDDDVLDDGYRWRKYGQKSVKNNAHPRSYYRCTYHTCNVKKQVQRLAKDPNVVVTTYEGVHNHPCEKLMETLSPLLRQLQFLSRVSDL

>AT1G66550.1

MVSNIDHKAMEALLRGQGCANNLKILLENGEISSVSTEPLIHTILDSFSLALSFMDSPNHPPYHESSSHNMASHMSRRSSKQVQHRRKLCVAEGLVNYNHDSRTMCPNDGFTWRKYGQKTIKASAHKRCYYRCTYAKDQNCNATKRVQKIKDNPPVYRTTYLGKHVCKAFAVHDDTYSSTMIRFDQVVPEPIMPQLTTIDHQVITVEENSAEHIMNQECDINDYLVDDDPFWASQFPPFPSSDTMFLENISAFD

>AT1G66550.2

MVSNIDHKAMEALLRGQGCANNLKILLENGEISSVSTEPLIHTILDSFSLALSFMDSPNHPPYHESSSHNMASHMSRRSSKQKLCVAEGLVNYNHDSRTMCPNDGFTWRKYGQKTIKASAHKRCYYRCTYAKDQNCNATKRVQKIKDNPPVYRTTYLGKHVCKAFAVHDDTYSSTMIRFDQVVPEPIMPQLTTIDHQVITVEENSAEHIMNQECDINDYLVDDDPFWASQFPPFPSSDTMFLENISAFD

>AT1G66560.1

MFSNIDQTAVAALLRGQGCANSLKRLLENHKLSSDSTEPLIYTILNSFSLALSFVDPPSLLPHNESSLQNMTSHVLQRSSKKKYYGAEDLEYYRDESPTPRPDDGFTWRKYGQKTIKTSPYQRCYYRCTYAKDQNCNARKRVQMIQDNPPVYRTTYLGKHVCKAVAVHDDTYGSEMIKFDQVVSESVMPQLATIDEQAITMEDEAIDHIMNQECDINDFSVDDDPFWASQFPPFSSEDIMFFDNIANLD

>AT1G66600.1

MFSNIDHKAVAALLHGQGCANILKTVLDNCKVSSVSTEPLINTILDSFSLALSSVNSPNRQPHHESSSRDMAGLVPQRSSKKKICGVKGLEIYRDDSPNPRLDDGFTWRKYGQKTIKTSLYQRCYYRCAYAKDQNCYATKRVQMIQDSPPVYRTTYLGQHTCKAFGVHDNTYGSEMINFDQVVSESVMRQLATIGEQAVLMEDEANHIMNQEYDINDYLVDDEVFWGNEFPLFSSEDLMLF

>AT1G68150.1

MGFDFSTSKSKAKRQKRIEVRFASPLMGIDLSLKLEAEEKKKEIEGSKHSRENKEDEEHDASGDEDEQMVKEDEDDSSSLGLRTREEENEREELLQLQIQMESVKEENTRLRKLVEQTLEDYRHLEMKFPVIDKTKKMDLEMFLGVQGKRCVDITSKARKRGAERSPSMEREIGLSLSLEKKQKQEESKEAVQSHHQRYNSSSLDMNMPRIISSSQGNRKARVSVRARCETATMNDGCQWRKYGQKTAKGNPCPRAYYRCTVAPGCPVRKQVQRCLEDMSILITTYEGTHNHPLPVGATAMASTASTSPFLLLDSSDNLSHPSYYQTPQAIDSSLITYPQNSSYNNRTIRSLNFDGPSRGDHVSSSQNRLNWMM

>AT1G69310.1

MNDPDNPDLSNDDSAWRELTLTAQDSDFFDRDTSNILSDFGWNLHHSSDHPHSLRFDSDLTQTTGVKPTTVTSSCSSSAAVSVAVTSTNNNPSATSSSSEDPAENSTASAEKTPPPETPVKEKKKAQKRIRQPRFAFMTKSDVDNLEDGYRWRKYGQKAVKNSPFPRSYYRCTNSRCTVKKRVERSSDDPSIVITTYEGQHCHQTIGFPRGGILTAHDPHSFTSHHHLPPPLPNPYYYQELLHQLHRDNNAPSPRLPRPTTEDTPAVSTPSEEGLLGDIVPQTMRNP

>AT1G69310.2

MNDPDNPDLSNDDSAWRELTLTAQDSDFFDRDTSNILSDFGWNLHHSSDHPHSLRFDSDLTQTTGVKPTTVTSSCSSSAAVSVAVTSTNNNPSATSSSSEDPAENSTASAEKTPPPETPVKEKKKAQKRIRQPRFAFMTKSDVDNLEDGYRWRKYGQKAVKNSPFPRSYYRCTNSRCTVKKRVERSSDDPSIVITTYEGQHCHQTIGFPRGGILTAHDPHSFTSHHHLPPPLPNPYYYQELLHQLHRDNNAPSPRLPRPTTEDTPAVSTPSEEGLLGDIVPQTMRNP

>AT1G69810.1

MIKEETVSYFQTFDGVMAESDKEEELDATKAKVEKVREENEKLKLLLSTILNNYNSLQMQVSKVLGQQQGASSMELDHIDRQDENNDYDVDISLRLGRSEQKISKKEENKVDKISTKNVEESKDKRSALGFGFQIQSYEASKLDDLCRQVKLANAENKCVSSRKDVKSVRNENHQDVLEEHEQTGLKKTRVCVKASCEDPSINDGCQWRKYGQKTAKTNPLPRAYYRCSMSSNCPVRKQVQRCGEEETSAFMTTYEGNHDHPLPMEASHMAAGTSAAASLLQSGSSSSSSSTSASLSYFFPFHHFSISTTNSHPTVTLDLTRPNYPNQLPDDYPLSSSSFSLNFSSPDPPPPSSHDHTLNFSGLRTQAPLSTDSLLARYRTRLSGQQ

>AT1G80590.1

MSLEIDAKAVSALLLGQGCANNLKTLLKNHETGSVSTEPLINSILDSFSFALSSQNIPRHVSQRSSKKKMCGIQGMEDSPTPAHIDGFIWRKYGQKTIKTSPHQRWYYRCAYAKDQNCDATKRVQKIQDNPPVYRNTYVGQHACEAPAYAVNNGGTYGSKMIKFDYVIPESVMPQPLSIDSQEITMEDKDTDDHILNYINEHLMEDEAYDVFPDVLGERCCFGLEPFPGLNINKS

>AT1G80840.1

MDQYSSSLVDTSLDLTIGVTRMRVEEDPPTSALVEELNRVSAENKKLSEMLTLMCDNYNVLRKQLMEYVNKSNITERDQISPPKKRKSPAREDAFSCAVIGGVSESSSTDQDEYLCKKQREETVVKEKVSRVYYKTEASDTTLVVKDGYQWRKYGQKVTRDNPSPRAYFKCACAPSCSVKKKVQRSVEDQSVLVATYEGEHNHPMPSQIDSNNGLNRHISHGGSASTPVAANRRSSLTVPVTTVDMIESKKVTSPTSRIDFPQVQKLLVEQMASSLTKDPNFTAALAAAVTGKLYQQNHTEK

>AT2G03340.1

MAEKEEKEPSKLKSSTGVSRPTISLPPRPFGEMFFSGGVGFSPGPMTLVSNLFSDPDEFKSFSQLLAGAMASPAAAAVAAAAVVATAHHQTPVSSVGDGGGSGGDVDPRFKQSRPTGLMITQPPGMFTVPPGLSPATLLDSPSFFGLFSPLQGTFGMTHQQALAQVTAQAVQGNNVHMQQSQQSEYPSSTQQQQQQQQQASLTEIPSFSSAPRSQIRASVQETSQGQRETSEISVFEHRSQPQNADKPADDGYNWRKYGQKQVKGSDFPRSYYKCTHPACPVKKKVERSLDGQVTEIIYKGQHNHELPQKRGNNNGSCKSSDIANQFQTSNSSLNKSKRDQETSQVTTTEQMSEASDSEEVGNAETSVGERHEDEPDPKRRNTEVRVSEPVASSHRTVTEPRIIVQTTSEVDLLDDGYRWRKYGQKVVKGNPYPRSYYKCTTPDCGVRKHVERAATDPKAVVTTYEGKHNHDVPAARTSSHQLRPNNQHNTSTVNFNHQQPVARLRLKEEQIT

>AT2G04880.1

MAEVGKVLASDMELDHSNETKAVDDVVATTDKAEVIPVAVTRTETVVESLESTDCKELEKLVPHTVASQSEVDVASPVSEKAPKVSESSGALSLQSGSEGNSPFIREKVMEDGYNWRKYGQKLVKGNEFVRSYYRCTHPNCKAKKQLERSAGGQVVDTVYFGEHDHPKPLAGAVPINQDKRSDVFTAVSKGEQRIDIVSLIYKLCIVSYDIMFVEKTSGSSVQTLRQTEPPKIHGGLHVSVIPPADDVKTDISQSSRITGDNTHKDYNSPTAKRRKKGGNIELSPVERSTNDSRIVVHTQTLFDIVNDGYRWRKYGQKSVKGSPYPRSYYRCSSPGCPVKKHVERSSHDTKLLITTYEGKHDHDMPPGRVVTHNNMLDSEVDDKEGDANKTPQSSTLQSITKDQHVEDHLRKKTKTNGFEKSLDQGPVLDEKLKEEIKERSDANKDHAANHAKPEAKSDDKTTVCQEKAVGTLESEEQKPKTEPAQS

>AT2G04880.2

MAEVGKVLASDMELDHSNETKAVDDVVATTDKAEVIPVAVTRTETVVESLESTDCKELEKLVPHTVASQSEVDVASPVSEKAPKVSESSGALSLQSGSEGNSPFIREKVMEDGYNWRKYGQKLVKGNEFVRSYYRCTHPNCKAKKQLERSAGGQVVDTVYFGEHDHPKPLAGAVPINQDKRSDVFTAVSKEKTSGSSVQTLRQTEPPKIHGGLHVSVIPPADDVKTDISQSSRITGDNTHKDYNSPTAKRRKKGGNIELSPVERSTNDSRIVVHTQTLFDIVNDGYRWRKYGQKSVKGSPYPRSYYRCSSPGCPVKKHVERSSHDTKLLITTYEGKHDHDMPPGRVVTHNNMLDSEVDDKEGDANKTPQSSTLQSITKDQHVEDHLRKKTKTNGFEKSLDQGPVLDEKLKEEIKERSDANKDHAANHAKPEAKSDDKTTVCQEKAVGTLESEEQKPKTEPAQS

>AT2G21900.1

MNYPSNPNPSSTDFTEFFKFDDFDDTFEKIMEEIGREDHSSSPTLSWSSSEKLVAAEITSPLQTSLATSPMSFEIGDKDEIKKRKRHKEDPIIHVFKTKSSIDEKVALDDGYKWRKYGKKPITGSPFPRHYHKCSSPDCNVKKKIERDTNNPDYILTTYEGRHNHPSPSVVYCDSDDFDLNSLNNWSFQTANTYSFSHSAPY

>AT2G23320.1

MAVELMTRNYISGVGADSFAVQEAAASGLKSIENFIGLMSRDSFNSDQPSSSSASASASAAADLESARNTTADAAVSKFKRVISLLDRTRTGHARFRRAPVHVISPVLLQEEPKTTPFQSPLPPPPQMIRKGSFSSSMKTIDFSSLSSVTTESDNQKKIHHHQRPSETAPFASQTQSLSTTVSSFSKSTKRKCNSENLLTGKCASASSSGRCHCSKKRKIKQRRIIRVPAISAKMSDVPPDDYSWRKYGQKPIKGSPHPRGYYKCSSVRGCPARKHVERAADDSSMLIVTYEGDHNHSLSAADLAGAAVADLILESS

>AT2G23320.2

MAVELMTRNYISGVGADSFAVQEAAASGLKSIENFIGLMSRDSFNSDQPSSSSASASASAAADLESARNTTADAAVSKFKRVISLLDRTRTGHARFRRAPVHVISPVLLQEEPKTTPFQSPLPPPPQMIRKGSFSSSMKTIDFSSLSSVTTESDNQKKIHHHQRPSETAPFASQTQSLSTTVSSFSKSTKRKCNSENLLTGKCASASSSGRCHCSKKRKIKQRRIIRVPAISAKMSDVPPDDYSWRKYGQKPIKGSPHPRIL

>AT2G24570.1

MTVDIMRLPKMEDQTAIQEAASQGLKSMEHLIRVLSNRPEERNVDCSEITDFTVSKFKKVISLLNRSGHARFRRGPVHSPPSSSVPPPVKVTTPAPTQISAPAPVSFVQANQQSVTLDFTRPSVFGAKTKSSEVVEFAKESFSVSSNSSFMSSAITGDGSVSKGSSIFLAPAPAVPVTSSGKPPLSGLPYRKRCFEHDHSEGFSGKISGSGNGKCHCKKSRKNRMKRTVRVPAVSAKIADIPPDEYSWRKYGQKPIKGSPHPRGYYKCSTFRGCPARKHVERALDDSTMLIVTYEGEHRHHQSTMQEHVTPSVSGLVFGSA

>AT2G25000.1

MDYDPNTNPFDLHFSGKLPKREVSASASKVVEKKWLVKDEKRNMLQDEINRVNSENKKLTEMLARVCEKYYALNNLMEELQSRKSPESVNFQNKQLTGKRKQELDEFVSSPIGLSLGPIENITNDKATVSTAYFAAEKSDTSLTVKDGYQWRKYGQKITRDNPSPRAYFRCSFSPSCLVKKKVQRSAEDPSFLVATYEGTHNHTGPHASVSRTVKLDLVQGGLEPVEEKKERGTIQEVLVQQMASSLTKDPKFTAALATAISGRLIEHSRT

>AT2G30250.1

MSSTSFTDLLGSSGVDCYEDDEDLRVSGSSFGGYYPERTGSGLPKFKTAQPPPLPISQSSHNFTFSDYLDSPLLLSSSHSLISPTTGTFPLQGFNGTTNNHSDFPWQLQSQPSNASSALQETYGVQDHEKKQEMIPNEIATQNNNQSFGTERQIKIPAYMVSRNSNDGYGWRKYGQKQVKKSENPRSYFKCTYPDCVSKKIVETASDGQITEIIYKGGHNHPKPEFTKRPSQSSLPSSVNGRRLFNPASVVSEPHDQSENSSISFDYSDLEQKSFKSEYGEIDEEEEQPEMKRMKREGEDEGMSIEVSKGVKEPRVVVQTISDIDVLIDGFRWRKYGQKVVKGNTNPRSYYKCTFQGCGVKKQVERSAADERAVLTTYEGRHNHDIPTALRRS

>AT2G30590.1

MEEIEGTNRAAVESCHRVLNLLHRSQQQDHVGFEKNLVSETREAVIRFKRVGSLLSSSVGHARFRRAKKLQSHVSQSLLLDPCQQRTTEVPSSSSQKTPVLRSGFQELSLRQPSDSLTLGTRSFSLNSNAKAPLLQLNQQTMPPSNYPTLFPVQQQQQQQQQQQQQEQQQQQQQQQQQFHERLQAHHLHQQQQLQKHQAELMLRKCNGGISLSFDNSSCTPTMSSTRSFVSSLSIDGSVANIEGKNSFHFGVPSSTDQNSLHSKRKCPLKGDEHGSLKCGSSSRCHCAKKRKHRVRRSIRVPAISNKVADIPPDDYSWRKYGQKPIKGSPYPRGYYKCSSMRGCPARKHVERCLEDPAMLIVTYEAEHNHPKLPSQAITT

>AT2G34830.1

MDNFQGDLTDVVRGIGSGHVSPSPGPPEGPSPSSMSPPPTSDLHVEFPSAATSASCLANPFGDPFVSMKDPLIHLPASYISGAGDNKSNKSFAIFPKIFEDDHIKSQCSVFPRIKISQSNNIHDASTCNSPAITVSSAAVAASPWGMINVNTTNSPRNCLLVDNNNNTSSCSQVQISSSPRNLGIKRRKSQAKKVVCIPAPAAMNSRSSGEVVPSDLWAWRKYGQKPIKGSPYPRGYYRCSSSKGCSARKQVERSRTDPNMLVITYTSEHNHPWPTQRNALAGSTRSSSSSSLNPSSKSSTAAATTSPSSRVFQNNSSKDEPNNSNLPSSSTHPPFDAAAIKEENVEERQEKMEFDYNDVENTYRPELLQEFQHQPEDFFADLDELEGDSLTMLLSHSSGGGNMENKTTIPDVFSDFFDDDESSRSL

>AT2G37260.1

MEVNDGERVVIAKPVASRPSSSSGFRTFTELLTDSVTVSPQTTCHEIVDAAIRPKTLRFNQPVAASVSCPRAEVKGIGNGMSCDDDSDSRNYVVYKPKAKLVSKATVSALANMLQGNRQQTWRQSEAVSYGKSVSQGTHRAGPNLVQKVPSFTESETSTGDRSSVDGYNWRKYGQKQVKGSECPRSYYKCTHPKCPVKKKVERSVEGQVSEIVYQGEHNHSKPSCPLPRRASSSISSGFQKPPKSIASEGSMGQDPNNNLYSPLWNNQSNDSTQNRTEKMSEGCVITPFEFAVPRSTNSNPGTSDSGCKSSQCDEGELDDPSRSKRRKNEKQSSEAGVSQGSVESDSLEDGFRWRKYGQKVVGGNAYPRSYYRCTSANCRARKHVERASDDPRAFITTYEGKHNHHLLLSPPSSSTLPFNSPQLSKQTI

>AT2G37260.2

MSCDDDSDSRNYVVYKPKAKLVSKATVSALANMGNRQQTWRQSEAVSYGKSVSQGTHRAGPNLVQKVPSFTESETSTGDRSSVDGYNWRKYGQKQVKGSECPRSYYKCTHPKCPVKKKVERSVEGQVSEIVYQGEHNHSKPSCPLPRRASSSISSGFQKPPKSIASEGSMGQDPNNNLYSPLWNNQSNDSTQNRTEKMSEGCVITPFEFAVPRSTNSNPGTSDSGCKSSQCDEGELDDPSRSKRRKNEKQSSEAGVSQGSVESDSLEDGFRWRKYGQKVVGGNAYPRSYYRCTSANCRARKHVERASDDPRAFITTYEGKHNHHLLLSPPSSSTLPFNSPQLSKQTI

>AT2G38470.1

MAASFLTMDNSRTRQNMNGSANWSQQSGRTSTSSLEDLEIPKFRSFAPSSISISPSLVSPSTCFSPSLFLDSPAFVSSSANVLASPTTGALITNVTNQKGINEGDKSNNNNFNLFDFSFHTQSSGVSAPTTTTTTTTTTTTTNSSIFQSQEQQKKNQSEQWSQTETRPNNQAVSYNGREQRKGEDGYNWRKYGQKQVKGSENPRSYYKCTFPNCPTKKKVERSLEGQITEIVYKGSHNHPKPQSTRRSSSSSSTFHSAVYNASLDHNRQASSDQPNSNNSFHQSDSFGMQQEDNTTSDSVGDDEFEQGSSIVSRDEEDCGSEPEAKRWKGDNETNGGNGGGSKTVREPRIVVQTTSDIDILDDGYRWRKYGQKVVKGNPNPRSYYKCTTIGCPVRKHVERASHDMRAVITTYEGKHNHDVPAARGSGYATNRAPQDSSSVPIRPAAIAGHSNYTTSSQAPYTLQMLHNNNTNTGPFGYAMNNNNNNSNLQTQQNFVGGGFSRAKEEPNEETSFFDSFMP

>AT2G40740.1

MYSYKKISYQMEEVMSMIFHGMKLVKSLESSLPEKPPESLLTSLDEIVKTFSDANERLKMLLEIKNSETALNKTKPVIVSVANQMLMQMEPGLMQEYWLRYGGSTSSQGTEAMFQTQLMAVDGGGERNLTAAVERSGASGSSTPRQRRRKDEGEEQTVLVAALRTGNTDLPPDDNHTWRKYGQKEILGSRFPRAYYRCTHQKLYNCPAKKQVQRLNDDPFTFRVTYRGSHTCYNSTAPTASSATPSTIPISSVTTGHSVDYGLAVVDMADVMFGSGGVGTNMDFIFPKNDPS

>AT2G40740.2

MYSYKKISYQMEEVMSMIFHGMKLVKSLESSLPEKPPESLLTSLDEIVKTFSDANERLKMLLEIKNSETALNKTKPVIVSVANQMLMQMEPGLMQEYWLRYGGSTSSQGTEAMFQTQLMAVDGGGERNLTAAVERSGASGSSTPRQRRRAYYRCTHQKLYNCPAKKQVQRLNDDPFTFRVTYRGSHTCYNSTAPTASSATPSTIPISSVTTGHSVDYGLAVVDMADVMFGSGGVGTNMDFIFPKNDPS

>AT2G40750.1

MDSNSNNTKSIKRKVVDQLVEGYEFATQLQLLLSHQHSNQYHIDETRLVSGSGSVSGGPDPVDELMSKILGSFHKTISVLDSFDPVAVSVPIAVEGSWNASCGDDSATPVSCNGGDSGESKKKRLGVGKGKRGCYTRKTRSHTRIVEAKSSEDRYAWRKYGQKEILNTTFPRSYFRCTHKPTQGCKATKQVQKQDQDSEMFQITYIGYHTCTANDQTHAKTEPFDQEIIMDSEKTLAASTAQNHVNAMVQEQENNTSSVTAIDAGMVKEEQNNNGDQSKDYYEGSSTGEDLSLVWQETMMFDDHQNHYYCGETSTTSHQFGFIDNDDQFSSFFDSYCADYERTSAM

>AT2G44745.1

MEGGGRRVFSNYDLQQVTSSSTTIQENMNFLVPFEETNVLTFFSSSSSSSLSSPSFPIHNSSSTTTTHAPLGFSNNLQGGGPLGSKVVNDDQENFGGGTNNDAHSNSWWRSNSGSGDMKNKVKIRRKLREPRFCFQTKSDVDVLDDGYKWRKYGQKVVKNSLHPRSYYRCTHNNCRVKKRVERLSEDCRMVITTYEGRHNHIPSDDSTSPDHDCLSSF

>AT2G46130.1

MNGLVDSSRDKKMKNPRFSFRTKSDADILDDGYRWRKYGQKSVKNSLYPRSYYRCTQHMCNVKKQVQRLSKETSIVETTYEGIHNHPCEELMQTLTPLLHQLQFLSKFT

>AT2G46130.2

MKNPRFSFRTKSDADILDDGYRWRKYGQKSVKNSLYPRCTQHMCNVKKQVQRLSKETSIVETTYEGIHNHPCEELMQTLTPLLHQLQFLSKFT

>AT2G46400.1

MMMEEKLVINELELGKELANRLMNNLKHTSSVDSNKTLISDILRIYQNAIFMLSFNQDKNILKRSLEIDGKDSKNVFKKRKVSEKNTEKVKVFVATEQENGSIDDGHCWRKYGQKEIHGSKNPRAYYRCTHRFTQDCLAVKQVQKSDTDPSLFEVKYLGNHTCNNITSPKTTTNFSVSLTNTNIFEGNRVHVTEQSEDMKPTKSEEVMISLEDLENKKNIFRTFSFSNHEIENGVWKSNLFLGNFVEDLSPATSGSAITSEVLSAPAAVENSETADSYFSSLDNIIDFGQDWLWS

>AT2G47260.1

MEFTDFSKTSFYYPSSQSVWDFGDLAAAERHSLGFMELLSSQQHQDFATVSPHSFLLQTSQPQTQTQPSAKLSSSIIQAPPSEQLVTSKVESLCSDHLLINPPATPNSSSISSASSEALNEEKPKTEDNEEEGGEDQQEKSHTKKQLKAKKNNQKRQREARVAFMTKSEVDHLEDGYRWRKYGQKAVKNSPFPRSYYRCTTASCNVKKRVERSFRDPSTVVTTYEGQHTHISPLTSRPISTGGFFGSSGAASSLGNGCFGFPIDGSTLISPQFQQLVQYHHQQQQQELMSCFGGVNEYLNSHANEYGDDNRVKKSRVLVKDNGLLQDVVPSHMLKEE

>AT3G01080.1

MAVEDDVSLIRTTTLVAPTRPTITVPHRPPAIETAAYFFGGGDGLSLSPGPLSFVSSLFVDNFPDVLTPDNQRTTSFTHLLTSPMFFPPQSSAHTGFIQPRQQSQPQPQRPDTFPHHMPPSTSVAVHGRQSLDVSQVDQRARNHYNNPGNNNNNRSYNVVNVDKPADDGYNWRKYGQKPIKGCEYPRSYYKCTHVNCPVKKKVERSSDGQITQIIYKGQHDHERPQNRRGGGGRDSTEVGGAGQMMESSDDSGYRKDHDDDDDDDEDDEDLPASKIRRIDGVSTTHRTVTEPKIIVQTKSEVDLLDDGYRWRKYGQKVVKGNPHPRSYYKCTTPNCTVRKHVERASTDAKAVITTYEGKHNHDVPAARNGTAAATAAAVGPSDHHRMRSMSGNNMQQHMSFGNNNNTGQSPVLLRLKEEKITI

>AT3G01970.1

MEDRRCDVLFPCSSSVDPRLTEFHGVDNSAQPTTSSEEKPRSKKKKKEREARYAFQTRSQVDILDDGYRWRKYGQKAVKNNPFPRSYYKCTEEGCRVKKQVQRQWGDEGVVVTTYQGVHTHAVDKPSDNFHHILTQMHIFPPFCLKE

>AT3G04670.1

MEEVEAANRSAIESCHGVLNLLSQRTSDPKSLTVETGEVVSKFKRVASLLTRGLGHGKFRSTNKFRSSFPQHIFLESPICCGNDLSGDYTQVLAPEPLQMVPASAVYNEMEPKHQLGHPSLMLSHKMCVDKSFLELKPPPFRAPYQLIHNHQQIAYSRSNSGVNLKFDGSGSSCYTPSVSNGSRSFVSSLSMDASVTDYDRNSFHLTGLSRGSDQQHTRKMCSGSLKCGSRSKCHCSKKRKLRVKRSIKVPAISNKIADIPPDEYSWRKYGQKPIKGSPHPRGYYKCSSVRGCPARKHVERCIDETSMLIVTYEGEHNHSRILSSQSAHT

>AT3G04670.2

MEEVEAANRSAIESCHGVLNLLSQRTSDPKSLTVETGEVVSKFKRVASLLTRGLGHGKFRSTNKFRSSFPQHIFLESPICCGNDLSGDYTQVLAPEPLQMVPASAVYNEMEPKHQLGHPSLMLSHKMCVDKSFLELKPPPFRAPYQLIHNHQQIAYSRSNSGVNLKFDGSGSSCYTPSVSNGSRSFVSSLSMDASVTDYDRNSFHLTGLSRGSDQQHTRKMCSGSLKCGSRSKCHCSKKRKLRVKRSIKVPAISNKIADIPPDEYSWRKYGQKPIKGSPHPRYIYKHLLV

>AT3G56400.1

MDTNKAKKLKVMNQLVEGHDLTTQLQQLLSQPGSGLEDLVAKILVCFNNTISVLDTFEPISSSSSLAAVEGSQNASCDNDGKFEDSGDSRKRLGPVKGKRGCYKRKKRSETCTIESTILEDAFSWRKYGQKEILNAKFPRSYFRCTHKYTQGCKATKQVQKVELEPKMFSITYIGNHTCNTNAETPKSKTCDHHDEIFMDSEDHKSPSLSTSMKEEDNPHRHHGSSTENDLSLVWPEMVFEEDYHHQASYVNGKTSTSIDVLGSQDLMVFGGGGDFEFSENEHFSIFSSCSNLS

>AT3G58710.1

MHRRAAIQESDDEEDETYNDVVPESPSSCEDSKISKPTPKKSRRNVEKRVVSVPIADVEGSKSRGEVYPPSDSWAWRKYGQKPIKGSPYPRGYYRCSSSKGCPARKQVERSRVDPSKLMITYACDHNHPFPSSSANTKSHHRSSVVLKTAKKEEEYEEEEEELTVTAAEEPPAGLDLSHVDSPLLLGGCYSEIGEFGWFYDASISSSSGSSNFLDVTLERGFSVGQEEDESLFGDLGDLPDCASVFRRGTVATEEQHRRCDFGAIPFCDSSR

>AT3G58710.2

MHRRAAIQESDDEEDETYNDVVPESPSSCEDSKISKPTPKKRRNVEKRVVSVPIADVEGSKSRGEVYPPSDSWAWRKYGQKPIKGSPYPRGYYRCSSSKGCPARKQVERSRVDPSKLMITYACDHNHPFPSSSANTKSHHRSSVVLKTAKKEEEYEEEEEELTVTAAEEPPAGLDLSHVDSPLLLGGCYSEIGEFGWFYDASISSSSGSSNFLDVTLERGFSVGQEEDESLFGDLGDLPDCASVFRRGTVATEEQHRRCDFGAIPFCDSSR

>AT3G62340.1

MENVGVGMPFYDLGQTRVYPLLSDFHDLSAERYPVGFMDLLGVHRHTPTHTPLMHFPTTPNSSSSEAVNGDDEEEEDGEEQQHKTKKRFKFTKMSRKQTKKKVPKVSFITRSEVLHLDDGYKWRKYGQKPVKDSPFPRNYYRCTTTWCDVKKRVERSFSDPSSVITTYEGQHTHPRPLLIMPKEGSSPSNGSASRAHIGLPTLPPQLLDYNNQQQQAPSSFGTEYINRQEKGINHDDDDDHVVKKSRTRDLLDGAGLVKDHGLLQDVVPSHIIKEEY

>AT4G01250.1

MADDWDLHAVVRGCSAVSSSATTTVYSPGVSSHTNPIFTVGRQSNAVSFGEIRDLYTPFTQESVVSSFSCINYPEEPRKPQNQKRPLSLSASSGSVTSKPSGSNTSRSKRRKIQHKKVCHVAAEALNSDVWAWRKYGQKPIKGSPYPRGYYRCSTSKGCLARKQVERNRSDPKMFIVTYTAEHNHPAPTHRNSLAGSTRQKPSDQQTSKSPTTTIATYSSSPVTSADEFVLPVEDHLAVGDLDGEEDLLSLSDTVVSDDFFDGLEEFAAGDSFSGNSAPASFDLSWVVNSAATTTGGI

>AT4G01720.1

MEEHIQDRREIAFLHSGEFLHGDSDSKDHQPNESPVERHHESSIKEVDFFAAKSQPFDLGHVRTTTIVGSSGFNDGLGLVNSCHGTSSNDGDDKTKTQISRLKLELERLHEENHKLKHLLDEVSESYNDLQRRVLLARQTQVEGLHHKQHEDVPQAGSSQALENRRPKDMNHETPATTLKRRSPDDVDGRDMHRGSPKTPRIDQNKSTNHEEQQNPHDQLPYRKARVSVRARSDATTVNDGCQWRKYGQKMAKGNPCPRAYYRCTMAVGCPVRKQVQRCAEDTTILTTTYEGNHNHPLPPSATAMAATTSAAAAMLLSGSSSSNLHQTLSSPSATSSSSFYHNFPYTSTIATLSASAPFPTITLDLTNPPRPLQPPPQFLSQYGPAAFLPNANQIRSMNNNNQQLLIPNLFGPQAPPREMVDSVRAAIAMDPNFTAALAAAISNIIGGGNNDNNNNTDINDNKVDAKSGGSSNGDSPQLPQSCTTFSTN

>AT4G04450.1

MFRFPVSLGGGPRENLKPSDEQHQRAVVNEVDFFRSAEKRDRVSREEQNIIADETHRVHVKRENSRVDDHDDRSTDHINIGLNLLTANTGSDESMVDDGLSVDMEEKRTKCENAQLREELKKASEDNQRLKQMLSQTTNNFNSLQMQLVAVMRQQEDHHHLATTENNDNVKNRHEVPEMVPRQFIDLGPHSDEVSSEERTTVRSGSPPSLLEKSSSRQNGKRVLVREESPETESNGWRNPNKVPKHHASSSICGGNGSENASSKVIEQAAAEATMRKARVSVRARSEAPMLSDGCQWRKYGQKMAKGNPCPRAYYRCTMAVGCPVRKQVQRCAEDRTILITTYEGNHNHPLPPAAMNMASTTTAAASMLLSGSTMSNQDGLMNPTNLLARTILPCSSSMATISASAPFPTITLDLTESPNGNNPTNNPLMQFSQRSGLVELNQSVLPHMMGQALYYNQQSKFSGLHMPSQPLNAGESVSAATAAIASNPNFAAALAAAITSIINGSNNQQNGNNNNSNVTTSNVDNRQ

>AT4G11070.1

MEMMNWERRSLLNELIHGLKAAKQLQGSSSPSLSASSSYLTTEIKENLLHNIVSSFKKAILMLNGSTTQHNPTIELAPDPLAHPGKVPGSPASITGNPRSEEFFNVRSKEFNLSSKKRKMLPKWTEQVRISPERGLEGPHDDIFSWRKYGQKDILGAKFPRSYYRCTFRNTQYCWATKQVQRSDGDPTIFEVTYRGTHTCSQGIPLPEKRETKPKHTVAVNYQNLRASLTVRTGGLGSEAFSFPVTSPLYTYESINGGGTFYHHVGSSGPSDFTGLISTNTSTGSSPIFDVNFQFDPTAEINTGFPTFFHNSI

>AT4G11070.2

MEMMNWERRSLLNELIHGLKAAKQLQAILMLNGSTTQHNPTIELAPDPLAHPGKVPGSPASITGNPRSEEFFNVRSKEFNLSSKKRKMLPKWTEQVRISPERGLEGPHDDIFSWRKYGQKDILGAKFPRSYYRCTFRNTQYCWATKQVQRSDGDPTIFEVTYRGTHTCSQGIPLPEKRETKPKHTVAVNYQNLRASLTVRTGGLGSEAFSFPVTSPLYTYESINGGGTFYHHVGSSGPSDFTGLISTNTSTGSSPIFDVNFQFDPTAEINTGFPTFFHNSI

>AT4G12020.1

MSEKEELPLTLTSIGAATATSDYHQRVGSSGEGISSSSSDVDPRFMQNSPTGLMISQSSSMCTVPPGMAATPPISSGSGLSQQLNNSSSSKLCQVEGCQKGARDASGRCISHGGGRRCQKPDCQKGAEGKTVYCKAHGGGRRCEYLGCTKGAEGSTDFCIAHGGGRRCNHEDCTRSAWGRTEFCVKHGGGARCKTYGCGKSASGPLPFCRAHGGGKKCSHEDCTGFARGRSGLCLMHGGGKRCQRENCTKSAEGLSGLCISHGGGRRCQSIGCTKGAKGSKMFCKACITKRPLTIDGGGNMGGVTTGDALNYLKAVKDKFEDSEKYDTFLEVLNDCKHQGVDTSGVIARLKDLFKGHDDLLLGFNTYLSKEYQITILPEDDFPIDFLDKVEGPYEMTYQQAQTVQANANMQPQTEYPSSSAVQSFSSGQPQIPTSAPDSSLLAKSNTSGITIIEHMSQQPLNVDKQVNDGYNWQKYGQKKVKGSKFPLSYYKCTYLGCPSKRKVERSLDGQVAEIVYKDRHNHEPPNQGKDGSTTYLSGSSTHINCMSSELTASQFSSNKTKIEQQEAASLATTIEYMSEASDNEEDSNGETSEGEKDEDEPEPKRRITEVQVSELADASDRTVREPRVIFQTTSEVDNLDDGYRWRKYGQKVVKGNPYPRFSSSKDYDVVIRYGRADISNEDFISHLRASLCRRGISVYEKFNEVDALPKCRVLIIVLTSTYVPSNLLNILEHQHTEDRVVYPIFYRLSPYDFVCNSKNYERFYLQDEPKKWQAALKEITQMPGYTLTDKSESELIDEIVRDALKVLCSADKVNMIGMDMQVEEILSLLCIESLDVRSIGIWGTVGIGKTTIAEEIFRKISVQYETCVVLKDLHKEVEVKGHDAVRENFLSEVLEVEPHVIRISDIKTSFLRSRLQRKRILVILDDVNDYRDVDTFLGTLNYFGPGSRIIMTSRNRRVFVLCKIDHVYEVKPLDIPKSLLLLDRGTCQIVLSPEVYKTLSLELVKFSNGNPQVLQFLSSIDREWNKLSQEVKTTSPIYIPGIFEKSCCGLDDNERGIFLDIACFFNRIDKDNVAMLLDGCGFSAHVGFRGLVDKSLLTISQHNLVDMLSFIQATGREIVRQESADRPGDRSRLWNADYIRHVFINDTGTSAIEGIFLDMLNLKFDANPNVFEKMCNLRLLKLYCSKAEEKHGVSFPQGLEYLPSKLRLLHWEYYPLSSLPKSFNPENLVELNLPSSCAKKLWKGKKARFCTTNSSLEKLKKMRLSYSDQLTKIPRLSSATNLEHIDLEGCNSLLSLSQSISYLKKLVFLNLKGCSKLENIPSMVDLESLEVLNLSGCSKLGNFPEISPNVKELYMGGTMIQEIPSSIKNLVLLEKLDLENSRHLKNLPTSIYKLKHLETLNLSGCISLERFPDSSRRMKCLRFLDLSRTDIKELPSSISYLTALDELLFVDSRRNSPVVTNPNANSTELMPSESSKLEILGTPADNEVVVGGTVEKTRGIERTPTILVKSREYLIPDDVVAVGGDIKGLRPPVLQLQPAMKLSHIPRGSTWDFVTHFAPPETVAPPSSSSEAREEEVETEETGAMFIPLGDKETCSFTVNKGDSSRTISNTSPIYASEGSFITCWQKGQLLGRGSLGSVYEGISADGDFFAFKEVSLLDQGSQAHEWIQQVEGGIALLSQLQHQNIVRYRGTTKDESNLYIFLELVTQGSLRKLYQRNQLGDSVVSLYTRQILDGLKYLHDKGFIHRNIKCANVLVDANGTVKLADFGLAKVMSLWRTPYWNWMAPEVIVLKSFPLF

>AT4G12020.2

MSEKEELPLTLTSIGAATATSDYHQRVGSSGEGISSSSSDVDPRFMQNSPTGLMISQSSSMCTVPPGMAATPPISSGSGLSQQLNNSSSSKLCQVEGCQKGARDASGRCISHGGGRRCQKPDCQKGAEGKTVYCKAHGGGRRCEYLGCTKGAEGSTDFCIAHGGGRRCNHEDCTRSAWGRTEFCVKHGGGARCKTYGCGKSASGPLPFCRAHGGGKKCSHEDCTGFARGRSGLCLMHGGGKRCQRENCTKSAEGLSGLCISHGGGRRCQSIGCTKGAKGSKMFCKACITKRPLTIDGGGNMGGVTTGDALNYLKAVKDKFEDSEKYDTFLEVLNDCKHQGVDTSGVIARLKDLFKGHDDLLLGFNTYLSKEYQITILPEDDFPIDFLDKVEGPYEMTYQQAQTVQANANMQPQTEYPSSSAVQSFSSGQPQIPTSAPDSSLLAKSNTSGITIIEHMSQQPLNVDKQVNDGYNWQKYGQKKVKGSKFPLSYYKCTYLGCPSKRKVERSLDGQVAEIVYKDRHNHEPPNQGKDGSTTYLSGSSTHINCMSSELTASQFSSNKTKIEQQEAASLATTIEYMSEASDNEEDSNGETSEGEKDEDEPEPKRRITEVQVSELADASDRTVREPRVIFQTTSEVDNLDDGYRWRKYGQKVVKGNPYPRFSSSKDYDVVIRYGRADISNEDFISHLRASLCRRGISVYEKFNEVDALPKCRVLIIVLTSTYVPSNLLNILEHQHTEDRVVYPIFYRLSPYDFVCNSKNYERFYLQDEPKKWQAALKEITQMPGYTLTDKSESELIDEIVRDALKVLCSADKVNMIGMDMQVEEILSLLCIESLDVRSIGIWGTVGIGKTTIAEEIFRKISVQYETCVVLKDLHKEVEVKGHDAVRENFLSEVLEVEPHVIRISDIKTSFLRSRLQRKRILVILDDVNDYRDVDTFLGTLNYFGPGSRIIMTSRNRRVFVLCKIDHVYEVKPLDIPKSLLLLDRGTCQIVLSPEVYKTLSLELVKFSNGNPQVLQFLSSIDREWNKLSQEVKTTSPIYIPGIFEKSCCGLDDNERGIFLDIACFFNRIDKDNVAMLLDGCGFSAHVGFRGLVDKSLLTISQHNLVDMLSFIQATGREIVRQESADRPGDRSRLWNADYIRHVFINDTGTSAIEGIFLDMLNLKFDANPNVFEKMCNLRLLKLYCSKAEEKHGVSFPQGLEYLPSKLRLLHWEYYPLSSLPKSFNPENLVELNLPSSCAKKLWKGKKARFCTTNSSLEKLKKMRLSYSDQLTKIPRLSSATNLEHIDLEGCNSLLSLSQSISYLKKLVFLNLKGCSKLENIPSMVDLESLEVLNLSGCSKLGNFPEISPNVKELYMGGTMIQEIPSSIKNLVLLEKLDLENSRHLKNLPTSIYKLKHLETLNLSGCISLERFPDSSRRMKCLRFLDLSRTDIKELPSSISYLTALDELLFVDSRRNSPVVTNPNANSTELMPSESSKLEILGTPADNEVVVGGTVEKTRGIERTPTILVKSREYLIPDDVVAVGGDIKGLRPPVLQLQPAMKLSHIPRGSTWDFVTHFAPPETVAPPSSSSEAREEEVETEETGAMFIPLGDKETCSFTVNKGDSSRTISNTSPIYASEGSFITCWQKGQLLGRGSLGSVYEGISADGDFFAFKEVSLLDQGSQAHEWIQQVEGGIALLSQLQHQNIVRYRGTTKDESNLYIFLELVTQGSLRKLYQRNQLGDSVVSLYTRQILDGLKYLHDKGFIHRNIKCANVLVDANGTVKLADFGLAKVMSLWRTPYWNWMAPEVILNPKDYDGYGTPADIWSLGCTVLEMLTGQIPYSDLEIGTALYNIGTGKLPKIPDILSLDARDFILTCLKVNPEERPTAAELLNHPFVNMPLPSSGSGSVSSLLRG

>AT4G12020.3

MSEKEELPLTLTSIGAATATSDYHQRVGSSGEGISSSSSDVDPRFMQNSPTGLMISQSSSMCTVPPGMAATPPISSGSGLSQQLNNSSSSKLCQVEGCQKGARDASGRCISHGGGRRCQKPDCQKGAEGKTVYCKAHGGGRRCEYLGCTKGAEGSTDFCIAHGGGRRCNHEDCTRSAWGRTEFCVKHGGGARCKTYGCGKSASGPLPFCRAHGGGKKCSHEDCTGFARGRSGLCLMHGGGKRCQRENCTKSAEGLSGLCISHGGGRRCQSIGCTKGAKGSKMFCKACITKRPLTIDGGGNMGGVTTGDALNYLKAVKDKFEDSEKYDTFLEVLNDCKHQGVDTSGVIARLKDLFKGHDDLLLGFNTYLSKEYQITILPEDDFPIDFLDKVEGPYEMTYQQAQTVQANANMQPQTEYPSSSAVQSFSSGQPQIPTSAPDSSLLAKSNTSGITIIEHMSQQPLNVDKQVNDGYNWQKYGQKKVKGSKFPLSYYKCTYLGCPSKRKVERSLDGQVAEIVYKDRHNHEPPNQGKDGSTTYLSGSSTHINCMSSELTASQFSSNKTKIEQQEAASLATTIEYMSEASDNEEDSNGETSEGEKDEDEPEPKRRITEVQVSELADASDRTVREPRVIFQTTSEVDNLDDGYRWRKYGQKVVKGNPYPRFSSSKDYDVVIRYGRADISNEDFISHLRASLCRRGISVYEKFNEVDALPKCRVLIIVLTSTYVPSNLLNILEHQHTEDRVVYPIFYRLSPYDFVCNSKNYERFYLQDEPKKWQAALKEITQMPGYTLTDKSESELIDEIVRDALKVLCSADKVNMIGMDMQVEEILSLLCIESLDVRSIGIWGTVGIGKTTIAEEIFRKISVQYETCVVLKDLHKEVEVKGHDAVRENFLSEVLEVEPHVIRISDIKTSFLRSRLQRKRILVILDDVNDYRDVDTFLGTLNYFGPGSRIIMTSRNRRVFVLCKIDHVYEVKPLDIPKSLLLLDRGTCQIVLSPEVYKTLSLELVKFSNGNPQVLQFLSSIDREWNKLSQEVKTTSPIYIPGIFEKSCCGLDDNERGIFLDIACFFNRIDKDNVAMLLDGCGFSAHVGFRGLVDKSLLTISQHNLVDMLSFIQATGREIVRQESADRPGDRSRLWNADYIRHVFINDTGTSAIEGIFLDMLNLKFDANPNVFEKMCNLRLLKLYCSKAEEKHGVSFPQGLEYLPSKLRLLHWEYYPLSSLPKSFNPENLVELNLPSSCAKKLWKGKKARFCTTNSSLEKLKKMRLSYSDQLTKIPRLSSATNLEHIDLEGCNSLLSLSQSISYLKKLVFLNLKGCSKLENIPSMVDLESLEVLNLSGCSKLGNFPEISPNVKELYMGGTMIQEIPSSIKNLVLLEKLDLENSRHLKNLPTSIYKLKHLETLNLSGCISLERFPDSSRRMKCLRFLDLSRTDIKELPSSISYLTALDELLFVDSRRNSPVVTNPNANSTELMPSESSKLEILGTPADNEVVVGGTVEKTRGIERTPTILVKSREYLIPDDVVAVGGDIKGLRPPVLQLQPAMKLSHIPRGSTWDFVTHFAPPETVAPPSSSSEAREEEVETEETGAMFIPLGDKETCSFTVNKGDSSRTISNTSPIYASEGSFITCWQKGQLLGRGSLGSVYEGISADGDFFAFKEVSLLDQGSQAHEWIQQVEGGIALLSQLQHQNIVRYRGTTKDESNLYIFLELVTQGSLRKLYQRNQLGDSVVSLYTRQILDGLKYLHDKGFIHRNIKCANVLVDANGTVKLADFGLAKVILNPKDYDGYGTPADIWSLGCTVLEMLTGQIPYSDLEIGTALYNIGTGKLPKIPDILSLDARDFILTCLKVNPEERPTAAELLNHPFVNMPLPSSGSGSVSSLLRG

>AT4G18170.1

MSNETRDLYNYQYPSSFSLHEMMNLPTSNPSSYGNLPSQNGFNPSTYSFTDCLQSSPAAYESLLQKTFGLSPSSSEVFNSSIDQEPNRDVTNDVINGGACNETETRVSPSNSSSSEADHPGEDSGKSRRKRELVGEEDQISKKVGKTKKTEVKKQREPRVSFMTKSEVDHLEDGYRWRKYGQKAVKNSPYPRSYYRCTTQKCNVKKRVERSFQDPTVVITTYEGQHNHPIPTNLRGSSAAAAMFSADLMTPRSFAHDMFRTAAYTNGGSVAAALDYGYGQSGYGSVNSNPSSHQVYHQGGEYELLREIFPSIFFKQEP

>AT4G22070.1

MFRFPVSLGGSRDEDRHDQITPLDDHRVVVDEVDFFSEKRDRVSRENINDDDDEGNKVLIKMEGSRVEENDRSRDVNIGLNLLTANTGSDESTVDDGLSMDMEDKRAKIENAQLQEELKKMKIENQRLRDMLSQATTNFNALQMQLVAVMRQQEQRNSSQDHLLAQESKAEGRKRQELQIMVPRQFMDLGPSSGAAEHGAEVSSEERTTVRSGSPPSLLESSNPRENGKRLLGREESSEESESNAWGNPNKVPKHNPSSSNSNGNRNGNVIDQSAAEATMRKARVSVRARSEAAMISDGCQWRKYGQKMAKGNPCPRAYYRCTMAGGCPVRKQVQRCAEDRSILITTYEGNHNHPLPPAATAMASTTTAAASMLLSGSMSSQDGLMNPTNLLARAILPCSSSMATISASAPFPTITLDLTNSPNGNNPNMTTNNPLMQFAQRPGFNPAVLPQVVGQAMYNNQQQSKFSGLQLPAQPLQIAATSSVAESVSAASAAIASDPNFAAALAAAITSIMNGSSHQNNNTNNNNVATSNNDSRQ

>AT4G23550.1

MDEGDLEAIVRGYSGSGDAFSGESSGTFSPSFCLPMETSSFYEPEMETSGLDELGELYKPFYPFSTQTILTSSVSLPEDSKPFRDDKKQRSHGCLLSNGSRADHIRISESKSKKSKKNQQKRVVEQVKEENLLSDAWAWRKYGQKPIKGSPYPRSYYRCSSSKGCLARKQVERNPQNPEKFTITYTNEHNHELPTRRNSLAGSTRAKTSQPKPTLTKKSEKEVVSSPTSNPMIPSADESSVAVQEMSVAETSTHQAAGAIEGRRLSNGLPSDLMSGSGTFPSFTGDFDELLNSQEFFSGYLWNY

>AT4G23810.1

MEGRDMLSWEQKTLLSELINGFDAAKKLQARLREAPSPSSSFSSPATAVAETNEILVKQIVSSYERSLLLLNWSSSPSVQLIPTPVTVVPVANPGSVPESPASINGSPRSEEFADGGGSSESHHRQDYIFNSKKRKMLPKWSEKVRISPERGLEGPQDDVFSWRKYGQKDILGAKFPRSYYRCTHRSTQNCWATKQVQRSDGDATVFEVTYRGTHTCSQAITRTPPLASPEKRQDTRVKPAITQKPKDILESLKSNLTVRTDGLDDGKDVFSFPDTPPFYNYGTINGEFGHVESSPIFDVVDWFNPTVEIDTTFPAFLHESIYY

>AT4G24240.1

MTVELMMSSYSGGGGGGDGFPAIAAAAKMEDTALREAASAGIHGVEEFLKLIGQSQQPTEKSQTEITAVTDVAVNSFKKVISLLGRSRTGHARFRRAPASTQTPFKQTPVVEEEVEVEEKKPETSSVLTKQKTEQYHGGGSAFRVYCPTPIHRRPPLSHNNNNNQNQTKNGSSSSSPPMLANGAPSTINFAPSPPVSATNSFMSSHRCDTDSTHMSSGFEFTNPSQLSGSRGKPPLSSASLKRRCNSSPSSRCHCSKKRKSRVKRVIRVPAVSSKMADIPSDEFSWRKYGQKPIKGSPHPRGYYKCSSVRGCPARKHVERALDDAMMLIVTYEGDHNHALVLETTTMNHDKTL

>AT4G26440.1

MAGIDNKAAVMGEWFDCSTTNHRKRSKAELGREFSLNYIKNEDSLQTTFQESSRGALRERIAARSGFNAPWLNTEDILQSKSLTISSPGLSPATLLESPVFLSNPLLSPTTGKLSSVPSDKAKAELFDDITTSLAFQTISGSGLDPTNIALEPDDSQDYEERQLGGLGDSMACCAPADDGYNWRKYGQKLVKGSEYPRSYYKCTHPNCEAKKKVERSREGHIIEIIYTGDHIHSKPPPNRRSGIGSSGTGQDMQIDATEYEGFAGTNENIEWTSPVSAELEYGSHSGSMQVQNGTHQFGYGDAAADALYRDENEDDRTSHMSVSLTYDGEVEESESKRRKLEAYATETSGSTRASREPRVVVQTTSDIDILDDGYRWRKYGQKVVKGNPNPRSYYKCTANGCTVTKHVERASDDFKSVLTTYIGKHTHVVPAARNSSHVGAGSSGTLQGSLATQTHNHNVHYPMPHSRSEGLATANSSLFDFQSHLRHPTGFSVYIGQSELSDLSMPGLTIGQEKLTSLQAPDIGDPTGLMLQLAAQPKVEPVSPQQGLDLSASSLICREMLSRLRQI

>AT4G26640.1

MILLPEPSPTTGSLFKPRPVHISASSSSYTGRGFHQNTFTEQKSSEFEFRPPASNMVYAELGKIRSEPPVHFQGQGHGSSHSPSSISDAAGSSSELSRPTPPCQMTPTSSDIPAGSDQEESIQTSQNDSRGSTPSILADDGYNWRKYGQKHVKGSEFPRSYYKCTHPNCEVKKLFERSHDGQITDIIYKGTHDHPKPQPGRRNSGGMAAQEERLDKYPSSTGRDEKGSGVYNLSNPNEQTGNPEVPPISASDDGGEAAASNRNKDEPDDDDPFSKRRRMEGAMEITPLVKPIREPRVVVQTLSEVDILDDGYRWRKYGQKVVRGNPNPRSYYKCTAHGCPVRKHVERASHDPKAVITTYEGKHDHDVPTSKSSSNHEIQPRFRPDETDTISLNLGVGISSDGPNHASNEHQHQNQQLVNQTHPNGVNFRFVHASPMSSYYASLNSGMNQYGQRETKNETQNGDISSLNNSSYPYPPNMGRVQSGP

>AT4G26640.2

MNPQANDRKEFQGDCSATGDLTAKHDSAGGNGGGGARYKLMSPAKLPISRSTDITIPPGLSPTSFLESPVFISNIKPEPSPTTGSLFKPRPVHISASSSSYTGRGFHQNTFTEQKSSEFEFRPPASNMVYAELGKIRSEPPVHFQGQGHGSSHSPSSISDAAGSSSELSRPTPPCQMTPTSSDIPAGSDQEESIQTSQNDSRGSTPSILADDGYNWRKYGQKHVKGSEFPRSYYKCTHPNCEVKKLFERSHDGQITDIIYKGTHDHPKPQPGRRNSGGMAAQEERLDKYPSSTGRDEKGSGVYNLSNPNEQTGNPEVPPISASDDGGEAAASNRNKDEPDDDDPFSKRRRMEGAMEITPLVKPIREPRVVVQTLSEVDILDDGYRWRKYGQKVVRGNPNPRSYYKCTAHGCPVRKHVERASHDPKAVITTYEGKHDHDVPTSKSSSNHEIQPRFRPDETDTISLNLGVGISSDGPNHASNEHQHQNQQLVNQTHPNGVNFRFVHASPMSSYYASLNSGMNQYGQRETKNETQNGDISSLNNSSYPYPPNMGRVQSGP

>AT4G30935.1

MEEDTGIDEAKTYTVEKSEKVEPEKDGLSQFRDEEKSLGADMEDLHDETVRETLGKDQVQGVRENSSVEPNVEDVLEVNETDSVKETVVSAIVPVDEVEENRQVETSPSLAASSDSLTVTPCLSLDPATASTAQDLPLVSVPTKQEQRSDSPVVNRLSVTPVPRTPARDGYNWRKYGQKQVKSPKGSRSYYRCTYTECCAKKIECSNDSGNVVEIVNKGLHTHEPPRKTSFSPREIRVTTAIRPVSEDDTVVEELSIVPSGSDPSASTKEYICESQTLVDRKRHCENEAVEEPEPKRRLKKDNSQSSDSVSKPGKKNKFVVHAAGDVGICGDGYRWRKYGQKMVKGNPHPRNYYRCTSAGCPVRKHIETAVENTKAVIITYKGVHNHDMPVPKKRHGPPSSMLVAAAAPTSMRTRTDDQVNIPTSSQCSVGRESEKQSKEALDVGGEKVMESARTLLSIGFEIKQC

>AT4G31550.1

MAVDLMRFPKIDDQTAIQEAASQGLQSMEHLIRVLSNRPEQQHNVDCSEITDFTVSKFKTVISLLNRTGHARFRRGPVHSTSSAASQKLQSQIVKNTQPEAPIVRTTTNHPQIVPPPSSVTLDFSKPSIFGTKAKSAELEFSKENFSVSLNSSFMSSAITGDGSVSNGKIFLASAPLQPVNSSGKPPLAGHPYRKRCLEHEHSESFSGKVSGSAYGKCHCKKSRKNRMKRTVRVPAISAKIADIPPDEYSWRKYGQKPIKGSPHPRGYYKCSTFRGCPARKHVERALDDPAMLIVTYEGEHRHNQSAMQENISSSGINDLVFASA

>AT4G31550.2

MAVDLMRFPKIDDQTAIQEAASQGLQSMEHLIRVLSNRPEQQHNVDCSEITDFTVSKFKTVISLLNRTGHARFRRGPVHSTSSAASQKLQSQIVKNTQPEAPIVRTTTNHPQIVPPPSSVTLDFSKPSIFGTKAKSAELEFSKENFSVSLNSSFMSSAITGDGSVSNGKIFLASAPLQPVNSSGKPPLAGHPYRKRCLEHEHSESFSGKVSGSAYGKCHCKKRKNRMKRTVRVPAISAKIADIPPDEYSWRKYGQKPIKGSPHPRGYYKCSTFRGCPARKHVERALDDPAMLIVTYEGEHRHNQSAMQENISSSGINDLVFASA

>AT4G31800.1

MDGSSFLDISLDLNTNPFSAKLPKKEVSVLASTHLKRKWLEQDESASELREELNRVNSENKKLTEMLARVCESYNELHNHLEKLQSRQSPEIEQTDIPIKKRKQDPDEFLGFPIGLSSGKTENSSSNEDHHHHHQQHEQKNQLLSCKRPVTDSFNKAKVSTVYVPTETSDTSLTVKDGFQWRKYGQKVTRDNPSPRAYFRCSFAPSCPVKKKVQRSAEDPSLLVATYEGTHNHLGPNASEGDATSQGGSSTVTLDLVNGCHRLALEKNERDNTMQEVLIQQMASSLTKDSKFTAALAAAISGRLMEQSRT

>AT4G31800.2

MDGSSFLDISLDLNTNPFSAKLPKEVSVLASTHLKRKWLEQDESASELREELNRVNSENKKLTEMLARVCESYNELHNHLEKLQSRQSPEIEQTDIPIKKRKQDPDEFLGFPIGLSSGKTENSSSNEDHHHHHQQHEQKNQLLSCKRPVTDSFNKAKVSTVYVPTETSDTSLTVKDGFQWRKYGQKVTRDNPSPRAYFRCSFAPSCPVKKKVQRSAEDPSLLVATYEGTHNHLGPNASEGDATSQGGSSTVTLDLVNGCHRLALEKNERDNTMQEVLIQQMASSLTKDSKFTAALAAAISGRLMEQSRT

>AT4G39410.1

MGAINQGISLFDESQTVINPINTNHLGFFFSFPSHSTLSSSSSSSSSSPSSLVSPFLGHNSLNSFLHNNPSSFISHPQDSINLMTNLPETLISSLSSSKQRDDHDGFLNLDHHRLTGSISSQRPLSNPWAWSCQAGYGSSQKNNHGSEIDVDDNDDEVGDGGGINDDDNGRHHHHDTPSRHDKHNTASLGVVSSLKMKKLKTRRKVREPRFCFKTLSEVDVLDDGYRWRKYGQKVVKNTQHPRSYYRCTQDKCRVKKRVERLADDPRMVITTYEGRHLHSPSNHLDDDSLSTSHLHPPLSNFFW

>AT5G01900.1

MNSCQQKAMEKLLHGHGCANQLLIMDQTESDSSMEREDLAKSVLHCFSDALSILIDTNDHQDDQSNNSSPQDSSPVLESSRKPLHKRGRKTSMAESSDYHRHESSTPIYHDGFLWRKYGQKQIKESEYQRSYYKCAYTKDQNCEAKKQVQKIQHNPPLYSTTYFGQHICQLHQAYATFPIDTSDFEEHEGSHMIRFGHPNISFSSSTSNLRQHQNHQDRIKDEYMKPVIAEDWSPSQWMSSEVALAVEAFEFNPFWTSHDLSS

>AT5G07100.1

MGSFDRQRAVPKFKTATPSPLPLSPSPYFTMPPGLTPADFLDSPLLFTSSNILPSPTTGTFPAQSLNYNNNGLLIDKNEIKYEDTTPPLFLPSMVTQPLPQLDLFKSEIMSSNKTSDDGYNWRKYGQKQVKGSENPRSYFKCTYPNCLTKKKVETSLVKGQMIEIVYKGSHNHPKPQSTKRSSSTAIAAHQNSSNGDGKDIGEDETEAKRWKREENVKEPRVVVQTTSDIDILDDGYRWRKYGQKVVKGNPNPRSYYKCTFTGCFVRKHVERAFQDPKSVITTYEGKHKHQIPTPRRGPVLRLLGKTET

>AT5G07100.2

MVTQPLPQLDLFKSEIMSSNKTSDDGYNWRKYGQKQVKGSENPRSYFKCTYPNCLTKKKVETSLVKGQMIEIVYKGSHNHPKPQSTKRSSSTAIAAHQNSSNGDGKDIGEDETEAKRWKREENVKEPRVVVQTTSDIDILDDGYRWRKYGQKVVKGNPNPRSYYKCTFTGCFVRKHVERAFQDPKSVITTYEGKHKHQIPTPRRGPVLRLLGKTET

>AT5G13080.1

MEGYDNGSLYAPFLSLKSHSKPELHQGEEESSKVRSEGCSKSVESSKKKGKKQRYAFQTRSQVDILDDGYRWRKYGQKAVKNNKFPRSYYRCTYGGCNVKKQVQRLTVDQEVVVTTYEGVHSHPIEKSTENFEHILTQMQIYSSF

>AT5G15130.1

MEVLLKLPSSESPLKDKFGSVQIHEANKGDGDHQELESAKAEMSEVKEENEKLKGMLERIESDYKSLKLRFFDIIQQEPSNTATKNQNMVDHPKPTTTDLSSFDQERELVSLSLGRRSSSPSDSVPKKEEKTDAISAEVNADEELTKAGLTLGINNGNGGEPKEGLSMENRANSGSEEAWAPGKVTGKRSSPAPASGGDADGEAGQQNHVKRARVCVRARCDTPTMNDGCQWRKYGQKIAKGNPCPRAYYRCTVAPGCPVRKQVQRCADDMSILITTYEGTHSHSLPLSATTMASTTSAAASMLLSGSSSSPAAEMIGNNLYDNSRFNNNNKSFYSPTLHSPLHPTVTLDLTAPQHSSSSSSSLLSLNFNKFSNSFQRFPSTSLNFSSTSSTSSNPSTLNLPAIWGNGYSSYTPYPYNNVQFGTSNLGKTVQNSQSLTETLTKALTSDPSFHSVIAAAISTMVGSNGEQQIVGPRHSISNNIQQTNTTNNNKGCGGYFSSLLMSNIMASNQTGASLDQPSSQLPPFSMFKNSSSSSSTTNFVNKEEKS

>AT5G22570.1

MEMNSPHEKAVQAIRYGHSCAMRLKRRLNHPMADGGPLSSYDLAKSIVESFSNAISILSAKPETEDDQFSDLSSRDSSPPPQGSPSKKRKIDSTNSSENWRDDSPDPIYYDGYLWRKYGQKSIKKSNHQRSYYRCSYNKDHNCEARKHEQKIKDNPPVYRTTYFGHHTCKTEHNLDAIFIAGQDPLDDFKSTQMIRFGKDQDQEKESRSNGFSLSVKHEEDIIKEQAIDQYREITSNDQDCQDVIEEYLSSPSGSYPPSSSSGSESADFNSDLLFDNPDSWDRYDQFYF

>AT5G24110.1

MEKNHSSGEWEKMKNEINELMIEGRDYAHQFGSASSQETREHLAKKILQSYHKSLTIMNYSGELDQVSQGGGSPKSDDSDQEPLVIKSSKKSMPRWSSKVRIAPGAGVDRTLDDGFSWRKYGQKDILGAKFPRGYYRCTYRKSQGCEATKQVQRSDENQMLLEISYRGIHSCSQAANVGTTMPIQNLEPNQTQEHGNLDMVKESVDNYNHQAHLHHNLHYPLSSTPNLENNNAYMLQMRDQNIEYFGSTSFSSDLGTSINYNFPASGSASHSASNSPSTVPLESPFESYDPNHPYGGFGGFYS

>AT5G26170.1

MNDADTNLGSSFSDDTHSVFEFPELDLSDEWMDDDLVSAVSGMNQSYGYQTSDVAGALFSGSSSCFSHPESPSTKTYVAATATASADNQNKKEKKKIKGRVAFKTRSEVEVLDDGFKWRKYGKKMVKNSPHPRNYYKCSVDGCPVKKRVERDRDDPSFVITTYEGSHNHSSMN

>AT5G28650.1

MEEVEAANKAAVESCHGVLNLLSQQTNDSKSIMVETREAVCKFKRVSSLLSRGLGQRKIKKLNNNNYKFSSSLLPQHMFLESPVCSNNAISGCIPILAPKPLQIVPAGPPPLMLFNQNMCLDKSFLELKPPSSRAVDPKPYQFIHTHQQGVYSRSKSGLNLKFDGSIGASCYSPSISNGSRSFVSSLSMDGSVTDYDRNSFHLIGLPQGSDHISQHSRRTSCSGSLKCGSKSKCHCSKKRKLRVKRSIKVPAISNKIADIPPDEYSWRKYGQKPIKGSPHPRGYYKCSSVRGCPARKHVERCVEETSMLIVTYEGEHNHSRILSSQSAHT

>AT5G41570.1

MDREDINPMLSRLDVENNNTFSSFVDKTLMMMPPSTFSGEVEPSSSSSWYPESFHVHAPPLPPENDQIGEKGKELKEKRSRKVPRIAFHTRSDDDVLDDGYRWRKYGQKSVKHNAHPRSYYRCTYHTCNVKKQVQRLAKDPNVVVTTYEGVHNHPCEKLMETLNPLLRQLQFLSSFSNL

>AT5G43290.1

MEEEGYQWARRCGNNAVEDPFVYEPPLFFLPQDQHHMHGLMPNEDFIANKFVTSTLYSGPRIQDIANALALVEPLTHPVREISKSTVPLLERSTLSKVDRYTLKVKNNSNGMCDDGYKWRKYGQKSIKNSPNPRSYYKCTNPICNAKKQVERSIDESNTYIITYEGFHFHYTYPFFLPDKTRQWPNKKTKIHKHNAQDMNKKSQTQEESKEAQLGELTNQNHPVNKAQENTPANLEEGLFFPVDQCRPQQGLLEDVVAPAMKNIPTRDSVLTAS

>AT5G45050.1

MTESEQIVYISCIEEVRYSFVSHLSKALQRKGVNDVFIDSDDSLSNESQSMVERARVSVMILPGNRTVSLDKLVKVLDCQKNKDQVVVPVLYGVRSSETEWLSALDSKGFSSVHHSRKECSDSQLVKETVRDVYEKLFYMERIGIYSKLLEIEKMINKQPLDIRCVGIWGMPGIGKTTLAKAVFDQMSGEFDAHCFIEDYTKAIQEKGVYCLLEEQFLKENAGASGTVTKLSLLRDRLNNKRVLVVLDDVRSPLVVESFLGGFDWFGPKSLIIITSKDKSVFRLCRVNQIYEVQGLNEKEALQLFSLCASIDDMAEQNLHEVSMKVIKYANGHPLALNLYGRELMGKKRPPEMEIAFLKLKECPPAIFVDAIKSSYDTLNDREKNIFLDIACFFQGENVDYVMQLLEGCGFFPHVGIDVLVEKSLVTISENRVRMHNLIQDVGRQIINRETRQTKRRSRLWEPCSIKYLLEDKEQNENEEQKTTFERAQVPEEIEGMFLDTSNLSFDIKHVAFDNMLNLRLFKIYSSNPEVHHVNNFLKGSLSSLPNVLRLLHWENYPLQFLPQNFDPIHLVEINMPYSQLKKLWGGTKDLEMLKTIRLCHSQQLVDIDDLLKAQNLEVVDLQGCTRLQSFPATGQLLHLRVVNLSGCTEIKSFPEIPPNIETLNLQGTGIIELPLSIVKPNYRELLNLLAEIPGLSGVSNLEQSDLKPLTSLMKISTSYQNPGKLSCLELNDCSRLRSLPNMVNLELLKALDLSGCSELETIQGFPRNLKELYLVGTAVRQVPQLPQSLEFFNAHGCVSLKSIRLDFKKLPVHYTFSNCFDLSPQVVNDFLVQAMANVIAKHIPRERHVTGFSQKTVQRSSRDSQQELNKTLAFSFCAPSHANQNSKLDLQPGSSSMTRLDPSWRNTLVGFAMLVQVAFSEGYCDDTDFGISCVCKWKNKEGHSHRREINLHCWALGKAVERDHTFVFFDVNMRPDTDEGNDPDIWADLVVFEFFPVNKQRKPLNDSCTVTRCGVRLITAVNCNTSIENISPVLSLDPMEVSGNEDEEVLRVRYAGLQEIYKALFLYIAGLFNDEDVGLVAPLIANIIDMDVSYGLKVLAYRSLIRVSSNGEIVMHYLLRQMGKEILHTESKKTDKLVDNIQSSMIATKEIEITRSKSRRKNNKEKRVVCVVDRGSRSSDLWVWRKYGQKPIKSSPYPRSYYRCASSKGCFARKQVERSRTDPNVSVITYISEHNHPFPTLRNTLAGSTRSSSSKCSDVTTSASSTVSQDKEGPDKSHLPSSPASPPYAAMVVKEEDMEQWDNMEFDVDVEEDTFIPELFPEDTFADMDKLEENSQTMFLSRRSSGGNMEAQGKNSSDDREVNLPSKILNR

>AT5G45050.2

MTESEQIVYISCIEEVRYSFVSHLSKALQRKGVNDVFIDSDDSLSNESQSMVERARVSVMILPGNRTVSLDKLVKVLDCQKNKDQVVVPVLYGVRSSETEWLSALDSKGFSSVHHSRKECSDSQLVKETVRDVYEKLFYMERIGIYSKLLEIEKMINKQPLDIRCVGIWGMPGIGKTTLAKAVFDQMSGEFDAHCFIEDYTKAIQEKGVYCLLEEQFLKENAGASGTVTKLSLLRDRLNNKRVLVVLDDVRSPLVVESFLGGFDWFGPKSLIIITSKDKSVFRLCRVNQIYEVQGLNEKEALQLFSLCASIDDMAEQNLHEVSMKVIKYANGHPLALNLYGRELMGKKRPPEMEIAFLKLKECPPAIFVDAIKSSYDTLNDREKNIFLDIACFFQGENVDYVMQLLEGCGFFPHVGIDVLVEKSLVTISENRVRMHNLIQDVGRQIINRETRQTKRRSRLWEPCSIKYLLEDKEQNENEEQKTTFERAQVPEEIEGMFLDTSNLSFDIKHVAFDNMLNLRLFKIYSSNPEVHHVNNFLKGSLSSLPNVLRLLHWENYPLQFLPQNFDPIHLVEINMPYSQLKKLWGGTKDLEMLKTIRLCHSQQLVDIDDLLKAQNLEVVDLQGCTRLQSFPATGQLLHLRVVNLSGCTEIKSFPEIPPNIETLNLQGTGVSNLEQSDLKPLTSLMKISTSYQNPGKLSCLELNDCSRLRSLPNMVNLELLKALDLSGCSELETIQGFPRNLKELYLVGTAVRQVPQLPQSLEFFNAHGCVSLKSIRLDFKKLPVHYTFSNCFDLSPQVVNDFLVQAMANVIAKHIPRERHVTGFSQKTVQRSSRDSQQELNKTLAFSFCAPSHANQNSKLDLQPGSSSMTRLDPSWRNTLVGFAMLVQVAFSEGYCDDTDFGISCVCKWKNKEGHSHRREINLHCWALGKAVERDHTFVFFDVNMRPDTDEGNDPDIWADLVVFEFFPVNKQRKPLNDSCTVTRCGVRLITAVNCNTSIENISPVLSLDPMEVSGNEDEEVLRVRYAGLQEIYKALFLYIAGLFNDEDVGLVAPLIANIIDMDVSYGLKVLAYRSLIRVSSNGEIVMHYLLRQMGKEILHTESKKTDKLVDNIQSSMIATKEIEITRSKSRRKNNKEKRVVCVVDRGSRSSDLWVWRKYGQKPIKSSPYPRSYYRCASSKGCFARKQVERSRTDPNVSVITYISEHNHPFPTLRNTLAGSTRSSSSKCSDVTTSASSTVSQDKEGPDKSHLPSSPASPPYAAMVVKEEDMEQWDNMEFDVDVEEDTFIPELFPEDTFADMDKLEENSQTMFLSRRSSGGNMEAQGKNSSDDREVNLPSKILNR

>AT5G45260.1

MTNCEKDEEFVCISCVEEVRYSFVSHLSEALRRKGINNVVVDVDIDDLLFKESQAKIEKAGVSVMVLPGNCDPSEVWLDKFAKVLECQRNNKDQAVVSVLYGDSLLRDQWLSELDFRGLSRIHQSRKECSDSILVEEIVRDVYETHFYVGRIGIYSKLLEIENMVNKQPIGIRCVGIWGMPGIGKTTLAKAVFDQMSSAFDASCFIEDYDKSIHEKGLYCLLEEQLLPGNDATIMKLSSLRDRLNSKRVLVVLDDVRNALVGESFLEGFDWLGPGSLIIITSRDKQVFCLCGINQIYEVQGLNEKEARQLFLLSASIKEDMGEQNLQELSVRVINYANGNPLAISVYGRELKGKKKLSEMETAFLKLKRRPPFKIVDAFKSTYDTLSDNEKNIFLDIACFFQGENVNYVIQLLEGCGFFPHVEIDVLVDKCLVTISENRVWLHKLTQDIGREIINGETVQIERRRRLWEPWSIKYLLEYNEHKANGEPKTTFKRAQGSEEIEGLFLDTSNLRFDLQPSAFKNMLNLRLLKIYCSNPEVHPVINFPTGSLHSLPNELRLLHWENYPLKSLPQNFDPRHLVEINMPYSQLQKLWGGTKNLEMLRTIRLCHSHHLVDIDDLLKAENLEVIDLQGCTRLQNFPAAGRLLRLRVVNLSGCIKIKSVLEIPPNIEKLHLQGTGILALPVSTVKPNHRELVNFLTEIPGLSEELERLTSLLESNSSCQDLGKLICLELKDCSCLQSLPNMANLDLNVLDLSGCSSLNSIQGFPRFLKQLYLGGTAIREVPQLPQSLEILNAHGSCLRSLPNMANLEFLKVLDLSGCSELETIQGFPRNLKELYFAGTTLREVPQLPLSLEVLNAHGSDSEKLPMHYKFNNFFDLSQQVVNDFLLKTLTYVKHIPRGYTQELINKAPTFSFSAPSHTNQNATFDLQSGSSVMTRLNHSWRNTLVGFGMLVEVAFPEDYCDATDVGISCVCRWSNKEGRSCRIERKFHCWAPWQVVPKVRKDHTFVFSDVNMRPSTGEGNDPDIWAGLVVFEFFPINQQTKCLNDRFTVRRCGVRVINVATGNTSLENIALVLSLDPVEVSGYEVLRVSYDDLQEMDKVLFLYIASLFNDEDVDFVAPLIAGIDLDVSSGLKVLADVSLISVSSNGEIVMHSLQRQMGKEILHGQSMLLSDCESSMTENLSDVPKKKKKHSESRVKKVVSIPAIDEGDLWTWRKYGQKDILGSRFPRGYYRCAYKFTHGCKATKQVQRSETDSNMLAITYLSEHNHPRPTKRKALADSTRSTSSSIC

>AT5G46350.1

MSHEIKDLNNYHYTSSYNHYNINNQNMINLPYVSGPSAYNANMISSSQVGFDLPSKNLSPQGAFELGFELSPSSSDFFNPSLDQENGLYNAYNYNSSQKSHEVVGDGCATIKSEVRVSASPSSSEADHHPGEDSGKIRKKREVRDGGEDDQRSQKVVKTKKKEEKKKEPRVSFMTKTEVDHLEDGYRWRKYGQKAVKNSPYPRSYYRCTTQKCNVKKRVERSYQDPTVVITTYESQHNHPIPTNRRTAMFSGTTASDYNPSSSPIFSDLIINTPRSFSNDDLFRVPYASVNVNPSYHQQQHGFHQQESEFELLKEMFPSVFFKQEP

>AT5G49520.1

MEKKKEEDHHHQQQQQQQKEIKNTETKIEQEQEQEQKQEISQASSSSNMANLVTSSDHHPLELAGNLSSIFDTSSLPFPYSYFEDHSSNNPNSFLDLLRQDHQFASSSNSSSFSFDAFPLPNNNNNTSFFTDLPLPQAESSEVVNTTPTSPNSTSVSSSSNEAANDNNSGKEVTVKDQEEGDQQQEQKGTKPQLKAKKKNQKKAREARFAFLTKSDIDNLDDGYRWRKYGQKAVKNSPYPRSYYRCTTVGCGVKKRVERSSDDPSIVMTTYEGQHTHPFPMTPRGHIGMLTSPILDHGATTASSSSFSIPQPRYLLTQHHQPYNMYNNNSLSMINRRSSDGTFVNPGPSSSFPGFGYDMSQASTSTSSSIRDHGLLQDILPSQIRSDTINTQTNEENKK

>AT5G52830.1

MSSEDWDLFAVVRSCSSSVSTTNSCAGHEDDIGNCKQQQDPPPPPLFQASSSCNELQDSCKPFLPVTTTTTTTWSPPPLLPPPKASSPSPNILLKQEQVLLESQDQKPPLSVRVFPPSTSSSVFVFRGQRDQLLQQQSQPPLRSRKRKNQQKRTICHVTQENLSSDLWAWRKYGQKPIKGSPYPRNYYRCSSSKGCLARKQVERSNLDPNIFIVTYTGEHTHPRPTHRNSLAGSTRNKSQPVNPVPKPDTSPLSDTVKEEIHLSPTTPLKGNDDVQETNGDEDMVGQEVNMEEEEEEEEVEEDDEEEEDDDDVDDLLIPNLAVRDRDDLFFAGSFPSWSAGSAGDGGG

>AT5G56270.1

MAGFDENVAVMGEWVPRSPSPGTLFSSAIGEEKSSKRVLERELSLNHGQVIGLEEDTSSNHNKDSSQSNVFRGGLSERIAARAGFNAPRLNTENIRTNTDFSIDSNLRSPCLTISSPGLSPATLLESPVFLSNPLAQPSPTTGKFPFLPGVNGNALSSEKAKDEFFDDIGASFSFHPVSRSSSSFFQGTTEMMSVDYGNYNNRSSSHQSAEEVKPGSENIESSNLYGIETDNQNGQNKTSDVTTNTSLETVDHQEEEEEQRRGDSMAGGAPAEDGYNWRKYGQKLVKGSEYPRSYYKCTNPNCQVKKKVERSREGHITEIIYKGAHNHLKPPPNRRSGMQVDGTEQVEQQQQQRDSAATWVSCNNTQQQGGSNENNVEEGSTRFEYGNQSGSIQAQTGGQYESGDPVVVVDASSTFSNDEDEDDRGTHGSVSLGYDGGGGGGGGEGDESESKRRKLEAFAAEMSGSTRAIREPRVVVQTTSDVDILDDGYRWRKYGQKVVKGNPNPRSYYKCTAPGCTVRKHVERASHDLKSVITTYEGKHNHDVPAARNSSHGGGGDSGNGNSGGSAAVSHHYHNGHHSEPPRGRFDRQVTTNNQSPFSRPFSFQPHLGPPSGFSFGLGQTGLVNLSMPGLAYGQGKMPGLPHPYMTQPVGMSEAMMQRGMEPKVEPVSDSGQSVYNQIMSRLPQI

>AT5G64810.1

MNISQNPSPNFTYFSDENFINPFMDNNDFSNLMFFDIDEGGNNGLIEEEISSPTSIVSSETFTGESGGSGSATTLSKKESTNRGSKESDQTKETGHRVAFRTRSKIDVMDDGFKWRKYGKKSVKNNINKRNYYKCSSEGCSVKKRVERDGDDAAYVITTYEGVHNHESLSNVYYNEMVLSYDHDNWNQHSLLRS

>LOC_Os01g08710.1

MALGHHGAARQPPTTMAAAASSSTTSAAAAPATATTTVAFSFQHPTPTPSCEEGDHRGRPQMGNKGEAAAAMGAMGINDAGNNTAAAAAAQHHLGVGAVRMKKVGGGGGGGGKARRKVREPRFCFKTMSDVDVLDDGYKWRKYGQKVVKNTQHPRSYYRCTQDNCRVKKRVERLAEDPRMVITTYEGRHVHSPSRDDDDAARASAEMTFICPLNRRNVPYGALLNSYGMPHFMIIVQIAFSTIYVVLVLLVAVTHVDSSLQGVYFGGNSSSAAAAAASQMSNQILIEFNQIYYFRFEFLKNHLNSSLMLTNFELARNQKTSSQISMGFPRNCISWLDTLLQPSPSTHASHLPPRGQLVASVDACGPSDADRIFPLRSTARETRSRGNINSSDLMDLSAIAIAHCISTKQPNHKVGASRERKKER*

>LOC_Os01g08710.2

MFPSPGRAVMALGHHGAARQPPTTMAAAASSSTTSAAAAPATATTTVAFSFQHPTPTPSHHHHHHGVLGYSPLVLDHHHPTTAAASSHAPSPPTLHHHHGGGLHAAAPPPRSSPPHPWSCEEGDHRGRPQMGNKGEAAAAMGAMGINDAGNNTAAAAAAQHHLGVGAVRMKKVGGGGGGGGKARRKVREPRFCFKTMSDVDVLDDGYKWRKYGQKVVKNTQHPRSYYRCTQDNCRVKKRVERLAEDPRMVITTYEGRHVHSPSRDDDDAARASAEMTFIW*

>LOC_Os01g09080.1

MDVVVESPPVRDEKKVDVAAIGGAPPIVFESFAPSTQRDSTIIKKEEKMEAAKAEMGEVREENERLKTMLTRIVSDYKSLHTHFLDVVKVKEQTAAELSGDDDDDEPDDLVSLSLCTRPNAAATRRKGHERTPSSGGGGDDGRLSLGLSCARGGVASDDDDDKQASRRALPPMPVLNLSSDSSGDAAGAGAGEPTQPNKASRSSSGGGDGADDEVLQQQQAKKARVSVRVKCDTPTMNDGCQWRKYGQKISKGNPCPRAYYRCTVAPNCPVRKQVQRCADDMSILITTYEGTHSHPLPPAAAAMASTTSAAAAMLTSGSTNSTMHGSGGVHHHLPFASAVGGGGGVGLLGPTTISTATSCPTVTLDLTAPHSLLHPSSASPYAAAAAGYESSRALPAAWSSGYLAYGGAAAAQPYYAKGVAPSPFGHHFGMMGMAAAAARPAPEQLFGGQTTSPYLQRAIGGGGVAPAAVTDTIAKAITSDPSFQSVLAAAITSYMGRGGGAAAPNK*

>LOC_Os01g09100.1

MAASLGLCHETSYAYSYPASNTSSSLCFPPLMADHIVDGGGGGGCSFGEFLELGHSVYSLPLPPPPSQPVVVAGGNNDQYGVSSSSSAAATTSRIGFRTRSEVEVLDDGFKWRKYGKKAVKSSPNPRNYYRCSAAGCGVKKRVERDGDDPRYVVTTYDGVHNHATPGCVGGGGHLPYPTSAAPPWSVPAAAASPPPAHAQAWGAPLHAAAAAHSSESSF*

>LOC_Os01g14440.1

MEADDSGGGGGRARRSVEVDFFSDEKKNMKKSRVSGGVAAEADDAKGPAAAGLAIKKEDLTINVSRRRLLRSSIARHLFNSRRSNACARPCSLQLLPAGNNARSDRSMVVDDDAASRPDHEEKSRSSNELAAMQAELGRMNEENQRLRGMLTQVTTSYQALQMHLVALMQQRPQMMQPPTQPEPPPPHQDGKAEGAVVPRQFLDLGPSSGAGGEAAEEPSNSSTEAGSPRRSSSTGNKDQERGDSPDAPSTAAAWLPGRAMAPQMGAAGAAGKSHDQQAQDANMRKARVSVRARSEAPIIADGCQWRKYGQKMAKGNPCPRAYYRCTMATGCPVRKQVQRCAEDRSILITTYEGTHNHPLPPAAMAMASTTSAAASMLLSGSMPSADGAAGLMSSNFLARTVLPCSSSMATISASAPFPTVTLDLTHAPPGAPNAVPLNAARPGAPAPQFQVPLPGGGMAPAFAVPPQVLYNQSKFSGLQMSSDSAEAAAAAAAAAQFAQPRPPIGQLPGPLSDTVSAAAAAITADPNFTVALAAAITSIIGGQHAAAAGNSNANNTNTNTTSNTNNTSSNNTTSNNTNSETQ*

>LOC_Os01g18584.1

MELRPPPPKHHHHRRRRGGGGEDGGEEEEEETGRLSLRGGGFWRRHDGEEEEEKGGGRRGEIKEVDFFLGASGRDVVVASRRHDDGFRGTTHGGGGGGDVNIGLDLLTTTTAGAAAGGAAAGAGEEDTGKNHRKEATTAAVDVELRRVVEENRRLRGMLDELNRSYSALYHQYLQVTQQQNHRHPDHHLIMNNNNNRPSLAQTHRTAATTTATTQQFLEPRASSTAQATADADMAASDDEAGRGGGDGDASSPSLSNAAGGGGGGNKMRRVGGQDETAAAAPARENGEQQAAAAAELPCRKPRVSVRARSEAPMISDGCQWRKYGQKMAKGNPCPRAYYRCTMAIGCPVRKQVQRCAEDKTVLITTYEGNHNHQLPPAATTMANTTSAAAAMLLSGPAASRDGAAAALLGHHHHHHPAAMFHQSFPYASTMATLSASAPFPTITLDLTQTPAGGAGAASLLHALHRPPVIHPGAAAQAMPFAVPPQLAMYLPQQRAAAAGLGGAGAARQPSVMETVTAALAADPNFTTALAAAISSVVAGGAHHQALSTTPRGSAAGAGDGNGNGSSAAAVATGAASPAATAEAPAASGSPPRLATQSCTTSN*

>LOC_Os01g40260.1

MSSLYPSLLSLSESPAEYRQVGGGRYAGEDVVDDDDDMAAVADAVSSYLSFDMDDVEYYTPEVGFHSKQHNPPPVAAAPLEAGGGREQSRREAAVNLGKMDRGPAPVSGGAATGGVPRSKNGSKIAFKTRSEVDVLDDGYRWRKYGKKMVKNSPNPRNYYRCSSEGCRVKKRVERARDDARFVVTTYDGVHNHPAPLHLRPQLPPPGGYSIAGAPAVVAPHGRLGLEEAEVIALFRGTTATSLLLP*

>LOC_Os01g40430.1

MNTFTLFRDEVQKASEKVHRHHANDDEAGLFLSLGLSLGSSPDACQCHASKKDEADAGNGGGDGYLALALRCAPAAGEPMVHPKRQRATTNSSSSSSICGEYGGGAAAAAVPAGHDDDDRSCMITAASTANRPGRVVLRTRCSAPTVKDGCQWRKYGQKTAKGNPWPRGYYRCTGAPGCPVKKQVQRCNHDTSVLVTTYDGVHNHPITPYAAALPPSSSSSSSAAVAMLASSSSSSTWSELQRAMPAAQSSWSQRNYPIQADVVAKAIWDPKFQATVAAAVASYVRDREQSARVAGGKGAGELFNLAPPC*

>LOC_Os01g43550.1

MRGPLLLRAVVVVAMEHFNDWDLQAVVRSCSFPQSEPPRVGVGVPAAPGAGGAPVVVAPPARAPDGPDQMARASASALYDLEYLDLDHKPFLLPGSSSSSSSSRAVARARGEDDGKGRHEVMISFPAAAAASTSGAQPRSPSGRKPGIRTPRPKRRSSKKSQLKKVVYEVPVADGGVSSDLWAWRKYGQKPIKGSPYPRGYYKCSSMKGCMARKMVERSPAKPGMLVVTYMAEHCHPVPTQLNALAGTTRHKSAPTGDDDKPTSPGPAAGRAAAGEGVVKCEDVDGNELSAMAADGGAEDTAAAADDDGELWPEGMGLELDEFLGPMDDDVFEFDHVLEDDGVLGRRLSL*

>LOC_Os01g43550.2

MRGPLLLRAVVVVAMEHFNDWDLQAVVRSCSFPQSEPPRVGVGVPAAPGAGGAPVVVAPPARAPDGPDQMARASASALYDLEYLDLDHKPFLLPGSSSSSSSSRAVARARGEDDGKGRHEVMISFPAAAAASTSGAQPRSPSGRKPGIRTPRPKRSKKSQLKKVVYEVPVADGGVSSDLWAWRKYGQKPIKGSPYPRGYYKCSSMKGCMARKMVERSPAKPGMLVVTYMAEHCHPVPTQLNALAGTTRHKSAPTGDDDKPTSPGPAAGRAAAGEGVVKCEDVDGNELSAMAADGGAEDTAAAADDDGELWPEGMGLELDEFLGPMDDDVFEFDHVLEDDGVLGRRLSL*

>LOC_Os01g43650.1

MSSGGGGGGGGDRHGPYHQHGHLGRGEGADYVYSSSDMESFFFSQPGGVGIGGGGGGVVGAGGADEIMPYSSITDYLQGLLDPSGLARHLDVACPSSQDTVVKQELSVDVTSHDSQGTGGVAGEGVAQATPNSSASFSSSDGEAEGGKSSRRCKKGQAKAEEEDDKDEEDGENSKKPNKPKKKAEKRQRQPRVAFLTKSEVDHLEDGYRWRKYGQKAVKNSPYPRSYYRCTTPKCGVKKRVERSYQDPSTVITTYEGQHTHHSPASLRGGGGGVGIVGGHHHHHLFMPGVHGLPPSHLMPAGFHPELMGLMHHHPAMAAAAANPSMYFPGVAASAPPPPAVAGGGAMPPNDHPPLQQHHFTDYALLQDLFPSTMPSSNP*

>LOC_Os01g43650.2

MSSGGGGGGGGDRHGPYHQHGHLGRGEGADYVYSSSDMESFFFSQPGGVGIGGGGGGVVGAGGADEIMPYSSITDYLQGLLDPSGLARHLDVACPSSQDTVVKQELSVDVTSHDSQGTGGVAGEGVAQATPNSSASFSSSDGEAEGGKSSRRCKKGQAKAEEEDDKDEEDGENSKKPNKPKKKAEKRQRQPRVAFLTKSEVDHLEDGYRWRKYGQKAVKNSPYPRSYYRCTTPKCGVKKRVERSYQDPSTVITTYEGQHTHHSPASLRGGGGGVGIVGGHHHHHLFMPGVHGLPPSHLMPAGFHPELMGLMHHHPAMAAAAANPSMYFPGVAASAPPPPAVAGGGAMPPNDHPPLQQHHFTDYALLQDLFPSTMPSSNP*

>LOC_Os01g46800.1

MAAGADERCRALVSGLLSSIDRSISIARSCCTEAAAAGRLTQQAGAAPESPPSADGSAGSDLGADSRCRANAAGPCKKRKTLPKWSKQVKVRSVQDVGPLDDGFSWRKYGQKDILGAKYPRAYFRCTHRHTQGCHASKQVQRADGDPLLFDVVYHGDHTCAHGVRSAAAAIDGQAAASAEQKHQPTPPQEQNAVSVAFTSMAVVNASTSSPFVSPAMSDCQISYELGGGSMAGVRNVPDVELASKTNSSMGDDMEFMFSLDSDFLDTYKYSSYF*

>LOC_Os01g47560.1

MGVAVHWRRAGDSLHMGGEPRARAEAAWAAALPAALVALVRDTATYTRMMHRLRMETPRGPTCQLDPCCCCCTAHVIPPPPPVSRTHATRRDAEASVPPPPASAAVSSRSDGTGQMAAGVTLACAAPPPLRAPRASDGGRRRGVVKGGAGTDTCRSPQRLNVRPRERERVRACVRARAKNHEHGQRRREAAVDPAMSGEYQFQDELAPLFARPGGGAGEMQMLPSSWFADYLQAGTPMQMDYDLMCRALELPVGEDVKREVGVVDVVAAGGGGAPPLTPNTTSSMSTSSSEGVGGGGGGGAGAGAGEEESPARCKKEEDENKEEGKGEEDEGHKNKKGSAAKGGKAGKGEKRARQPRFAFMTKSEVDHLEDGYRWRKYGQKAVKNSPYPRSYYRCTTQKCPVKKRVERSYQDPAVVITTYEGKHTHPIPATLRGSTHLLAAHAQAAAAAAAAHQLHHHHGHHGHHGMAPPLPLGSGAAAQFGRSSGIDVLSSFLPRAAAAHHGMTTMGGAAATTTTSHGLNSAISGGGGVSSETTSAVTVAASAQPSSPAALQMQHFMAQDLGLLQDMLLPSFIHGTNQP*

>LOC_Os01g51690.1

MYMAAAAAGASTPFNFCRHGSHAEYDAVFSGSWMARRPSAAPHGGGASGSGSGSGYGAASYVAPTFGAAFRQQHLDLLDYLSDDQGVPAPPPAAVPSASYVTPAPAMAPAEPVVPDAVAAAGGYPRSVAAAAAAVAGEGRDRTTTDKIAFRTRSDDEILDDGYKWRKYGKKSVKNSPNPRNYYRCSTEGCNVKKRVERDKNDPRYVVTMYEGIHNHVCPGTVYYAAQDAASGRFFVAGISHPDLN*

>LOC_Os01g53040.1

MDGEWSDGAAVSSPTMSGGGGREQMKGGEDVAAADCPGSPVSPSPAAAQRSAAGAAASPSGRSRRSAQKRVVTVPLADVTGPRPKGVGEGNTPTDSWAWRKYGQKPIKGSPFPRAYYRCSSSKGCPARKQVERSRNDPDTVIVTYSFEHNHSATVPRAQNRQAAPQKPKAQACSPPEPVVEVEPEETHQYGVTAGPATGGGGGAAAIEVRDEFRWLYDVVSVPATSTSPSDIDAADEMQLYDQPMFFGGAVVGTAALLPDEFGDVGGLGGEGLGEEEALFEGLGELPECAMVFRRRAGDGLEMGGGVKIEQPAESTAMT*

>LOC_Os01g53260.1

MENLQLQGDDHDDEALPHFPYFAVPSPPPLAVAPAASATTSDGHQHGPLEVLEQPPCSNNLHPDGLVDGPQLAATTAVPMMLPAMTSLDWQSLLQTCLQVPPPVLEQQQPAAAAQADQYSGENDHGDLQAAESSGAGNKEKQVMAKGGAGRPSGTKKKASRPRFAFQTRSDNDILDDGYRWRKYGQKAVKNSKHPRSYYRCTHHTCNVKKQVQRLAKDTSIVVTTYEGVHNHPCEKLMEALTPILKQLQFLSQF*

>LOC_Os01g54600.1

MAAGEEVMDRSTSAEDGYCSAGTDSPRAESVDEQGAAEESSPRGGQKRELPSPSASPSSPLPPAAKRSRRSVEKRVVSVPIAECGDRPKGAGEGPPPSDSWAWRKYGQKPIKGSPYPRGYYRCSSSKGCPARKQVERSRADPTVLLVTYSFEHNHPWPQPKSSSCHASKSSPRSTAPKPEPVADGQHPEPAENESSASAELEVPEPEPEQESEPVVKQEEEQKEEQKAVVEPAAVTTTVAPAPAVEEEDENFDFGWIDQYHPTWHRSYAPLLPPEEWERELQGDDALFAGLGELPECAVVFGRRRELGLAATAPCS*

>LOC_Os01g60490.1

MVKRSDNMDSSSECSRGAHKRLLQDSRSYDQENAMKKVCIGTRTEYTYAPYHDGYQWRKYGQKMIRGNSFPRCYYRCTYHQDHGCPASKHVEQHNSEDPPLFRVIYTNEHTCGTSNSASDYMASSMQIQQIADASLRKAQAAERLRKAEVETPRLMHSPPPRCSGGYNMAMKEEKDVIVSSLLTVIRGCHIAESAGNNSAAALPVNRPPPAVARSDHYSCSYAISPELLPASDDLTLDFMLDSVLDPHWVEPLDLAWLKESTHTG*

>LOC_Os01g60520.1

MASPRLKREQSFDFEEASAQEAVGSASASYSPPGGGGVFGISPPESSPRDGRKRRKDRPSLVKHTFTPHFDGHLWRKYGQKNIKDSAFPRLYYRCSYREDRQCLASKLVQQENDDDPPLYRVTYTYEHTCNTTPVPTPDVVAEQPPPGAAGDAYLLRFGSSAGGGGGGAHQQQTERERQQQNTARRRPFMMLSFDSSSSHQLHEQPHAFPPDGQLPATAAAASPSSFTAAEALAAPPLTTTMNDGGDLFSTWDALRYGLDYDHGHLGNHVYLPDDCNGGDDNY*

>LOC_Os01g60540.1

MAFGQDTIEQLYRELAGGRRLSAKLQALLEGPLDSRGQKEAVDVSRELGRVFMVSLYMLKPCSNSSRRPEGVTRTAPETRTDDSICLHTPARVKRVRSEEVLVRNGREEVVTRTEIITPSPYKDGYQWRKYGQKNIQDSNYLRLYFKCTFSRERSCAAKKQVQQRDAGEPPMFLVTYLNEHTCQQPQAVPGTPNTAGSSPTTTSRQRQSSSSPPAEMLDLTMNGAGLFSRLLLPHAVGGGGSAAEEEAAIVTCLAAVISGGGAAAAPPPLIWPTSAPEAAFVASAAGHSPSAADESVADEAAAAQMADMDYCFGQYDQSTFGAAAAADHRVLIGDDGDVQRIVAARIADTVWPRYTRDTSAWETAGTSSMRGSID*

>LOC_Os01g60600.1

MQAQSRLAAAASGGSGSGISGSGGISRLGGGAGEEHEAVVRELTRGHELTARLRAEALRALRGQGQAEATATFILGEVSRAFTVCLSIMASASPSASPPQPDETPPADSAVSPPPPRAAREDNVPRKRLLTASPYDDGYQWRKYGQKKINNTNFPRSYYRCSYHRERRCPAQKHVQQRDGDDVPALHVVVYTHEHTCLQGAPAELPDAATNGGAAAAASPDYFPAGGETPSSLRRLRGVGGGGLQPQFVDHRAAMEERERQVLVSSLARVLQGRQCYDDDDDDDTDVASLGAVHARAPAAAAPVAASSSSSGPVDAAGEELDVMDYDMTDALFWGPFGTDSNSYDGNLTSTRCFDLIN*

>LOC_Os01g60640.1

MAMLGSSSAVVLELMTMGYQSAAYLGELLRAASPAQAGDEQQELAAEILRCCDRVIAKLNRGGATGATTGKKRKAAESAAAAAVTSPSLPVTPTKRRARGAEAVREVRSGTTTDGFIWRKYGQKEINGCKHPRLYYRCAFRGQGCLATRRVQQSQSQDDPAAAFVIAYYGEHTCGGDAAAAAACRDGELMPPAVINSGASSFAAAWNMASREPASSLAVERRSCDGDAPSETSQGWSPSFSSEVELDVVGFDLAGADSSASPVWEFLNGSFDWEFVINSL*

>LOC_Os01g61080.1

MTTSSSGSVETSANSRLGTFSFASASFTDLLGGNAGAGGGGVSRYKAMTPPSLPLSPPPVSPSSFFNSPIGMNQADFLGSPVLLTSSIFPSPTTGAFASQHFDWRPEVAAAQSADQGGKDEQRNSYSDFSFQTAPASEEAVRTTTFQPPVPPAPLGDEAYRSQQQQQPWGYQQQPAGMDAGANAASFGAAPFQATSSEMAPQVQGGGGYSQPQSQRRSSDDGYNWRKYGQKQVKGSENPRSYYKCTFPNCPTKKKVERSLDGQITEIVYKGTHNHAKPQNTRRNSGSSAAQVLQSGGDMSEHSFGGMSGTAATPENSSASFGDDEIRVGSPRAGNGGGDEFDDDEPDSKRWRKDGDGEGISMAGNRTVREPRVVVQTMSDIDILDDGYRWRKYGQKVVKGNPNPRSYYKCTTAGCPVRKHVERASHDLRAVITTYEGKHNHDVPAARGSAALYRPAPPAAAATSSHPYLPNQPPPMSYQPTGPQPYALRPDGFGGQGPFGGVVGGSSFGGFSGFDDARGSYMSQHQQQQRQNDAMHASRAKEEPGDDMFFQNSLY*

>LOC_Os01g62510.1

MEEDDGLALMTGANQSLWSYYKCTSSRCSAKKHVEKSTDDPEMLIVTYEGSHHHGPQPLFPPHIAQPPPPTSVVGFSAASGAGPPPSSPAAAARKRKNYVRAAFSPTTSEDDGDGAGRLRPEWPQDDGTSCDVAELRRRGDAEHAAPRRVATDRSCDDGGGGGSTSASSSVARADAATALSSDSPPTIWSCLDWPWSQETLFL*

>LOC_Os01g62514.1

MYGQSPHKQRRRFPGPYLMGDVLRAQATASAEEVAGGVWPCELDDHLIGELLGDDGLFVPAAEHPTLYYSFGAGSSAAAAAAPCNGGGSADHERRPRPAPAVSRDLCSVYSGPTIRDIEKALSSSASPRPPYPSGRRYSSLYFRRVEAESKYTSKVRSCGGKMPADGYKWRKYGQKSIKNNPHPRCATRSIIDPI*

>LOC_Os01g74140.1

MDGAMQESREYWRDGGDVVGEELLREILDETAAVHSNSNSNSNSNSNSKEAEEEDEREYFAAAAADEQLQVEAPCGRRRRESMVNKLISTVYSGPTISDIESALSFTAAGDHQLLADGHNFAASSCSPVVFSPEKTLSKTMENKYTLKMKSCGNNGGLADDGYKWRKYGQKSIKNSPNPRSYYRCTNPRCNAKKQVERAVDEPDTLIVTYEGLHLHYTYSHFLHSTSSSSSSTTTQQQLQPQPQMMTNCKKKPKLHLHPLLHDDPPPPPPPPEMTTMMIMQSFSIQQQQHDDDQLLQPAADDHLMVQAPPDDCYNINGSSSSGLMMSLDDDEQAAGAGGLLEDVVPLLVRRPPPPICNNNNYYYSPATTCTSDNEYGSSASASPSSSVSVSSWTTPMSPCIDMAILSNIF*

>LOC_Os02g08440.1

MDPWISTQPSLSLDLRVGLPATAAVAMVKPKVLVEEDFFHQQPLKKDPEVAALEAELKRMGAENRQLSEMLAAVAAKYEALQSQFSDMVTASANNGGGGGNNPSSTSEGGSVSPSRKRKSESLDDSPPPPPPPHPHAAPHHMHVMPGAAAAGYADQTECTSGEPCKRIREECKPKISKLYVHADPSDLSLVVKDGYQWRKYGQKVTKDNPCPRAYFRCSFAPACPVKKKVQRSAEDNTILVATYEGEHNHGQPPPPLQSAAQNSDGSGKSAGKPPHAPAAAPPAPVVPHRQHEPVVVNGEQQAAAASEMIRRNLAEQMAMTLTRDPSFKAALVTALSGRILELSPTKD*

>LOC_Os02g08440.2

MGAENRQLSEMLAAVAAKYEALQSQFSDMVTASANNGGGGGNNPSSTSEGGSVSPSRKRKSESLDDSPPPPPPPHPHAAPHHMHVMPGAAAAGYADQTECTSGEPCKRIREECKPKISKLYVHADPSDLSLVVKDGYQWRKYGQKVTKDNPCPRAYFRCSFAPACPVKKKVQRSAEDNTILVATYEGEHNHGQPPPPLQSAAQNSDGSGKSAGKPPHAPAAAPPAPVVPHRQHEPVVVNGEQQAAAASEMIRRNLAEQMAMTLTRDPSFKAALVTALSGRILELSPTKD*

>LOC_Os02g08440.3

MDPWISTQPSLSLDLRVGLPATAAVAMVKPKVLVEEDFFHQQPLKKDPEVAALEAELKRMGAENRQLSEMLAAVAAKYEALQSQFSDMVTASANNGGGGGNNPSSTSEGGSVSPSRKRKSESLDDSPPPPPPPHPHAAPHHMHVMPGAAAAGYADQTECTSGEPCKRIREECKPKISKLYVHADPSDLSLVVKDGYQWRKYGQKVTKDNPCPRAYFRCSFAPACPVKKKVT*

>LOC_Os02g08440.4

MDPWISTQPSLSLDLRVGLPATAAVAMVKPKPCKRIREECKPKISKLYVHADPSDLSLVVKDGYQWRKYGQKVTKDNPCPRAYFRCSFAPACPVKKKVQRSAEDNTILVATYEGEHNHGQPPPPLQSAAQNSDGSGKSAGKPPHAPAAAPPAPVVPHRQHEPVVVNGEQQAAAASEMIRRNLAEQMAMTLTRDPSFKAALVTALSGRILELSPTKD*

>LOC_Os02g16540.1

MEEELCGNNWDLDAVVRLGCCRRRISPAAVAQQVDPFASFLQQGVAMEVAAEKEVGVEAAWSFPELTVRDGGGGGLGRDADELLKAFCAAFPSSSSSKSSPLPTPPPPPPTQPQPEQQKPVTVQENLPAPTTAPARASQPAAARQVPAGGVPRSKRRKNQQKKVVRHVPADGVSADVWAWRKYGQKPIKGSPYPRGYYRCSSSKGCPARKQVERSRSDPNTFILTYTGEHNHSAPTHRNSLAGTTRNKLPSSSAASAASAQPQPPPPSVVVVGAGGGGAEAAGLSPTTPLRTPSMEEDEEEEEEEELLVEDMEMAGEDELLFLNGGDDNAALDGTPMSSLFDIADEPFLPSPWTEPTAAGS*

>LOC_Os02g26430.1

MADPFPAAARGGEQGGGTAGQLVSTPSRLRTAVASMLNRTGHARFRRAAPVVVQEEEDEAAAAARDAVVRCDGLSASASSSFPSSVTGVTGDGSVSNARAVLPAAGDGDKPPPMQSASDYASDGRLKRSSDDDGERCHCSKKKRKASWRARRRIRVPAISSRNADIPADDYSWRKYGQKPIKGSPYPRGYYKCSTVRGCPARKHVERDPGEPAMLIVTYDGDHRHGEPGHRRPDEAATTTEHRTTDQTTGRLL*

>LOC_Os02g43560.1

MHTCMEGGGQLGTCLPNFYLLPDHHGMPLPPPLQLPCHPKLLQMPFDQEDQPGIHGVMLSSDHCGLYPLPALPLSNSAAAAAATVALGKHSAAAGSMPNIGGAEEVATTVTKAGNESTTCNGSTTWWRGSTMAAMGEKGKMKIRRKMREPRFCFQTRSEVDVLDDGYKWRKYGQKVVKNSLHPRYICST*

>LOC_Os02g47060.1

MCDYFLQRMEGEQAAGDLADIVLRAGGAAAAAVAGGGIPSTEWQLPPAEEEEEEPGLFPLPPSSSDGSGMSGADAFGDPFAGLPDPFGGDYPSSGGAAAAADFFDAVVAKAGFVDVGVLGGGGGGGCDGGGVDGGGGGSSLLGMSKPILPRAAMQLPSVSPRAIRPYPVMAGDTVKLGAPMAGGPCAFDGAAAAGLHMSSSPRGAVGGIKRRKNQARKVVCIPAPAAAGGRTSGEVVPSDLWAWRKYGQKPIKGSPYPRGYYRCSSSKGCSARKQVERSRTDPNMLVITYTSEHNHPWPTQRNALAGSTRSHHAKNSSSNSSSSGASSASKNNSSHSGYHHHHHQKPLVKAEPNDQSAAATTAATVPVKEEAAMVGTSSEALAKTTQKSMEDAAAAASATAAAVEHSDLMQQMFSQSYRPMIPEAAAGGHHDDFFADLAELESDPMSLIFSKEYMATNYKPAGDPAGKEMNAVDKGLDPAYMLDWSSTTVVTRAGGSSFMQGEGGL*

>LOC_Os02g53100.1

MSSKKKRAAIDLSLEAERRRPEERGGGSDREASDGAAAAAEEDGDVKQREGPKEETGGEEEKVVEVVVDQGEDGSNEEIKYRTQQAEMIEEDKQPAAAANVDDDGGDSDGVGASAEEKHMVTEATGGEGDDGGDSRTPMAQDELSEMQEEMERMKEENRMLRRVVDKTVRDYYELQMKLAAYQQQPAAADEPKETEVFLSLGATAAASAGCGGGFPEAKSKEQAAWRRRSVGSDDSDCGKEDLGLSLSLGASSSYDDDQKAVEARPHDVDGAAAAAMIGGDGSRPAPRGYALLESSKVQGGAAPAAGELAAAGGITSQSVNPANRKTRVSVRVRCQGPTMNDGCQWRKYGQKVAKGNPCPRAYYRCTVAPGCPVRKQVQRCLEDMSILVTTYEGTHNHPLPVGATAMASTTSAAATFMLLSSTTSSSSVSDASAAPSSSYLSPYLLNSASPLLMPGATGGGGGMQHLNLFGNSPSSSSLLAPQAPGSSKYPWSPNHPPLAGAGGNKRPFWSAGGDGDKPAPAALAENVGAVMSDPNKFSAAIAAAINNFMGKDGESSSGKSSTFAWTRSMDCTATPASVPAPARPRREPVHGPGPRRGEQAAKL*

>LOC_Os03g20550.1

MSPVPSPHQSHHLGHGSRKEKRMRKVDTFAPHNDGHQWRKYGEKKINNCNFPRYYYRCTYKDNMNCPATKQIQQKDYSDPPLYSVTYYNEHTCNSAFLPLSPSEFQLQTASGKAVSICFESSGAQEPMTNASSPSSSAARRSTPSENKNQPLPRHSEAYSWGVGVVEQKPSCTELQSCSTECQDAFSAGTIPEETVDAGRFGSIRFFHFL*

>LOC_Os03g20550.2

MFLKKTCRKEKRMRKVDTFAPHNDGHQWRKYGEKKINNCNFPRYYYRCTYKDNMNCPATKQIQQKDYSDPPLYSVTYYNEHTCNSAFLPLSPSEFQLQTASGKAVSICFESSGAQEPMTNASSPSSSAARRSTPSENKNQPLPRHSEAYSWGVGVVEQKPSCTELQSCSTECQDAFSAGTIPEETVDAGRFGSIRFFHFL*

>LOC_Os03g21710.1

MAFSSEGGVPAERVAAAVNDLVEVRDGLVRLRGFLPPPPQAEQSSSRPPCAAELMDATMSKLMSAMATLGGSGDIAGEVDAAGRWTSVAESADPMVVRREGESSAGRTRRRRGGGSRSGRGRSSNKRVAATLEDGHVWRKYGQKDIQNSPYPRSYYRCTHKLDQGCGARRQTQRCEADPSNYDITYYGEHTCRDPSTIIPTAIANAAGAASDGPNNNIISFATGGVVVANSSRLAREGTTATTTSAATQLSSSWGTSGGGGGGDDVFSSSGERFMQWDELAAAVGHVSSVGVTSSTVGSAPAAENDGGNGDTAAGGGGDGGGAGSFPSSPSAGSLGFVVGPLGSIEDVDDFFPFDP*

>LOC_Os03g33012.1

MAAHEGGGNGARRPPAPPLLPTLSLPPRSAAGSLFSAESSPGALTLAASLFPDAPSPAFQGSFTQLLVGAMGYPAASAPAPPSPFPVPHGLSPTAFLGGSPGLFSPTGNFEMSHQQALAQVTAEAVHSPYSMINQSDFSLPFSSTTTSVLASQHVNSSANVSSPREIPTLPSHTDNSNIESTEVSHGFQTTALTEDKPADDGYNWRKYGQKAVKGGEYPRSYYKCTHLSCPVKKKVERSSDGQITQILYRGQHNHQRPPKRRSKDGGALLNEADVSPEKEDASTRSEQGSQDYSGKFKASNDGGPSSSRRGDRGEQISGSSDSNDQGEEEVKVEGRATSDGNANKRHVPAPAQRIIVQTTSEVDLLDDGYRWRKYGQKVVKGNPHPRSYYKCTYQGCDVKKHIERSSQDPKAVITTYEGKHSHDVPAARNSSHSSANANVSSSSNLPHKDRGQRSSCRDGLRNASSVSSLQLKEESG*

>LOC_Os03g45450.1

MASNSQTTTTGAGGGRGQGDDEEPTPTPPPAPPPETAPSTVGGGGDGVQLVMPEDGYEWKKYGQKFIKNIQKNRSYFRCRDQRCGAKKKVEWHPHDPGLNLRVVYDGAHHHGSPSSAAGEGGTSAAAAANQYDLSTQYFGGAGGPRSQ*

>LOC_Os03g53050.1

MEGMEEANREAVQSCHRVLTLLSSPHSQLVPNKDLAAATGEAVAKFCSVASRLNNGNGLQGHARVRKIKKPLPIFDSNLFLESPALAVAMAAKTPNSSPITSLQLFPRYHQMEGSSSKDPVRIPAQFPKRLLLDNPAVDSDGPSRGPPLQLIQPVSVAPPAGTPHPALPSAHLHFIQQHQSYQRFQLMQQMKMQSEMIKRSGLGEQGGSNGGGKGVNLKFDSSNCTASSSRSFLSSLSMEGSIASLDGSRSSRPFQLVSGSQTSSTPELGLMQRRRCTGREDGSGRCTTGSRCHCAKKRKLRIRRSIKVPAISNKVADIPADEFSWRKYGQKPIKGSPHPRGYYKCSSVRGCPARKHVERCVDDPSMLIVTYEGDHNHNRVLAAQPA*

>LOC_Os03g55080.1

MAGAEWSPFDGDAAFAEYSSAVLAELGGWAAPGEEGGAGMMVPAALDLPVDVVGAAAREEEEEEEEAPARSGDGAAAAASSSSSGEPAAPDKRPAAAEAAPAAAATATATAKKGQKRARQPRFAFMTKSEIDHLEDGYRWRKYGQKAVKNSPFPRSYYRCTNSKCTVKKRVERSSDDPSVVITTYEGQHCHHTASFQRGVGGAAVAAHIHGAAAVALAEQMSAFVSPPPQPHMLYGLPRLHPPSSETAVSCSMPTTTSLQELNNSEGLQRPGYNNSPQAAVTIAQRPPSPSVPPAVSFDKGLLDDIVPPGVRLG*

>LOC_Os03g55164.1

MAEALVAVLRLAASAAATARPQSRSGRHGSCAARVPCPGPSPFRRGRLCARAAVAGPPEVDDDDAMTIDNLRRFFDVNVGKWNGAFYFFFFFFFFFFFFFSGFVVCKTETLSVLVRVPVCLCSSRQQFDAHGRVLQGISTRLSVSTYGEDDLISLLQSLYIKQASSQISFVDEEDSEEWVEYKIKETNMFTVDKYQQVGFFQEEKAFALRYQTAGMLETVLRAGVLGEDDTGEESPKNLKIPSRKPSIVCENCLYSREGNGRVRAFHIMDPKGVLDMLIIFHEKQGSEVPLMYSSDDADITNSDRIAPLLGRWEGRSVTKRSGVYGATLSEADTVVLLEKDRNGQLILDNMSTKSGSSTTTTVHWTGSANNNLLQFDGGYEMTLLPGGMYMGYPTDIGKIVNDMDSFHLEFCWMESPGKRQRLVRTYDSAGLAVSSTYFFETKRLSSLKPHHHLRRRVGDHGRTPDLSNANRVAGEGEGGDRFRRRRVAAVTAAAIDRAKSPEEGDSFRWFWKYSAQAVGASQSNPNTSRSDPLSWSAAVVVIDREALPLHKQRKLTRAAAVAMADRRRSDGGGGMQQQPFTSPGQERVFDGGGVPGQVAAPYGSDFDQSSYMALLAAGAVGVGVGVQPTAAPWAVEEDVAAAPPGISLAPQFSMANYAPPPSYQHPATLVSPPLAAGLHPYPPYLHGVDAPPPQWPPRPAPPPSFSVLDLAAAAAPHEQRHSMQQLLLRAAAFGGGMHAAAAPAPAAAAAIEQPAKDGYNWRKYGQKQLKDAESPRSYYKCTRDGCPVKKIVERSSDGCIKEITYKGRHSHPRPVEPRRGGAASSSSSAMAAGTDHNAGAAADDAAAADEDDPSDDDDTLLHEDDDDGEEGHDRGVDGEVGQRVVRKPKIILQTRSEVDLLDDGYRWRKYGQKVVKGNPRPRSYYKCTADGCNVRKQIERASADPKCVLTTYTGRHNHDPPGRPPAAANLQMPGPAAMRLAGGGTAHQQPSGGAHQMKEET*

>LOC_Os03g55164.2

MADRRRSDGGGGMQQQPFTSPGQERVFDGGGVPGQVAAPYGSDFDQSSYMALLAAGAVGVGVGVQPTAAPWAVEEDVAAAPPGISLAPQFSMANYAPPPSYQHPATLVSPPLAAGLHPYPPYLHGVDAPPPQWPPRPAPPPSFSVLDLAAAAAPHEQRHSMQQLLLRAAAFGGGMHAAAAPAPAAAAAIEQPAKDGYNWRKYGQKQLKDAESPRSYYKCTRDGCPVKKIVERSSDGCIKEITYKGRHSHPRPVEPRRGGAASSSSSAMAAGTDHNAGAAADDAAAADEDDPSDDDDTLLHEDDDDGEEGHDRGVDGEVGQRVVRKPKIILQTRSEVDLLDDGYRWRKYGQKVVKGNPRPRSYYKCTADGCNVRKQIERASADPKCVLTTYTGRHNHDPPGRPPAAANLQMPGPAAMRLAGGGTAHQQPSGGAHQMKEET*

>LOC_Os03g58420.1

MDGVEEANMAAVESSKKLVAILSKSGDPFRLMAAVAETDEAVSRFGKVVTILSNRVGHARARLGKRRSSPPVDPGCLMDHPLAAAASSPAPSNGRLHFSSSAATASPSPATAAAASSAANVTPAVVDRSLFLETTLLDLNSRGAPAPAASMAAAAKNSSKLAPAPMVNSSSSANHIQFQQPMKSFQFEQTPISDKFHIEMPRGVGGGGGKEVISFSFDNSVCTSSAATSFFTSISSQLISMSDAATNSAAAAAAPTTKKPSSCARKATADDDAGGKCHCPKKKKPREKKVVTVPAISDKVADIPSDNYSWRKYGQKPIKGSPHPRGYYRCSSKKDCPARKHVERCRSDPAMLLVTYENEHNHAQPLDLSVVQQATANPQT*

>LOC_Os03g58420.2

MDGVEEANMAAVESSKKLVAILSKSGDPFRLMAAVAETDEAVSRFGKVVTILSNRVGHARARLGKRRSSPPVDPGCLMDHPLAAAASSPAPSNGRLHFSSSAATASPSPATAAAASSAANVTPAVVDRSLFLETTLLDLNSRGAPAPAASMAAAAKNSSKLAPAPMVNSSSSANHIQFQQPMKSFQFEQTPISDKFHIEMPRGVGGGGGKEVISFSFDNSVCTSSAATSFFTSISSQLISMSDAATNSAAAAAAPTTKKPSSCARKATADDDAGGKCHCPKKKKPREKKVVTVPAISDKVADIPSDNYSWRKYGQKPIKGSPHPRGYYRCSSKKDCPARKHVERCRSDPAMLLVTYENEHNHAQPLDLSVVQQATANPQT*

>LOC_Os03g63810.1

MDMMEEEAANAATAQAAAAGDLADVVARANARAFLVSTPHHHPSPLHPLPPPPMPQAPHQYYPAPQITIPYHHHHHGELRRPTTIAYTDAPVPFETAGPPSTVVDSYHHLTPGDAGYGMPRPLALQISQHALCGGGDVVMGGGGAGAADDGEEAIRISPLTPSAHHQMMKRKNEVKKVVCIPAPPATSSRGGGGEVIPSDLWAWRKYGQKPIKGSPYPRGYYRCSSSKGCMARKQVERSRSDPNMLVITYAAEHNHPWPMQRNVLAGYARSHHSTHATASSSRHKQQQQQQTNQLQPALITSSSSSSSSPFNLYADVVLGGQQANMMMTTEGAGAGLGIQPSAADEVFAELEELEPDNPTMINANMQVYSTTSRPGVSSYDHQWHKF*

>LOC_Os04g04300.1

MAKGNPCPRAYYRCTVAVGCPAAVAKSSSANAATTEMLELLRDFSDYSSFNSDISSELERLAAAVTPRSDAPQVAAVDLNGGSSSSSRLTTTTPPPLLQLGCRQALPFIVPTGRCASLAAVVEEISGRRASPPWMSAAGDAVAVIPSYENHVTEVSTCVKHSSSHTDIDRLRLSITTPFHDYDVITLIDKIINPLFWC*

>LOC_Os04g21950.1

MITMDLMGGYGRVDEQVAIQEAAAAGLRGMEHLILQLSQTGTSERSPAPAQEQQQQVDCREITDMTVSKFKKVISMLNRTGHARFRRGPVVAQSSGPAASEPAPVRSSPSAVSRPMTLDFTKAASGYGKDAGFSVSGISAASSSFLSSVTGDGSVSNGRGGGSSSLMLPPPPATSCGKPPLSSAAAAMSAGAGHKRKCHDHAHSENVAGGKYGSTGGRCHCSKRRKHRVKRTIRVPAISSKVADIPADDFSWRKYGQKPIKGSPFPRGYYKCSTLRGCPARKHVERDPTDPSMLIVTYEGEHRHSPSAAGQDHPPAPPPPLALPLA*

>LOC_Os04g39570.1

MTMTTATLDPPPPPLLIAGSLLDDDRDAGSAASSSPRWLPRARGSTVGSPPRRGGEERGGEAATRRKGNSSCPDDDDDCRNDEDDCRNDDDYRDDDVGATTDGGGDAAALLPMAMMTATTMAPMPVEAAVDGGGELSSAGEGGQRRVNLLPTKILSAYENHLFSQALACREDEWIIEKLAVIAARRSSFVSEESLCSVPAAALVSLAIAHLAFSPMLVYGVLDRLCSVCHLGEMEGDIAMEEWKDSNHRGADYLMTMPMQNFLADAFPPPELLEGEGGFEKHGLSVAVGSPPPTPPPPEDGCSPLPLTPQFGQKFGSGGGGGGSLADRRARGGFSNVARISVPYNQPAADVSSAGAPSPYVTIPPGLSPTTLLESPVFSNAMGQASPTTGKLHMLGGANDSNPIRFESPRIEEGSGAFSFKPLNLASSHYAAEEKTKSLPNNQHQSLPISVKTEATSIQTAQDEAAANQLMQPQFNGGKRSRAAPDNGGDGEGQPAEGDAKADSSSGAAAVAVVAAAAAAVAEDGYSWRKYGQKQVKHSEYPRSYYKCTHASCAVKKKVERSHEGHVTEIIYKGTHNHPKPAASRRPPVHPPPPSPATTTTTPLPPGDAQADHAPDGGGGSTPVGAGQAGAEWHNGGVVGGEGLVDATSSPSVPGELCESTASMQVHEGAAAAQLGESPEGVDVTSAVSDEVDRDDKATHVLPLAAAAADGESDELERKRRKLDSCATMDMSTASRAVREPRVVIQTTSEVDILDDGYRWRKYGQKVVKGNPNPRSYYKCTHPGCLVRKHVERASHDLKSVITTYEGKHNHEVPAARNSGHPAGSASPGGGAGSSSQPHGVGVGGRRPEVPSVQESLMRLGGGCGAAPFPPHFGLHLPPPPPRDPLAPMSNFPYSLGHAPSPALRGLPPPPPPPPSASALAVAGLGGVVEGLKYPMLAPPSVHSLLRHRQGGGMEAVVVPKAEVKQEAMRPAAAVAGAGRGAAVYQQAMSRVSLGNQL*

>LOC_Os04g39570.2

MEGDIAMEEWKDSNHRGADYLMTMPMQNFLADAFPPPELLEGEGGFEKHGLSVAVGSPPPTPPPPEDGCSPLPLTPQFGQKFGSGGGGGGSLADRRARGGFSNVARISVPYNQPAADVSSAGAPSPYVTIPPGLSPTTLLESPVFSNAMGQASPTTGKLHMLGGANDSNPIRFESPRIEEGSGAFSFKPLNLASSHYAAEEKTKSLPNNQHQSLPISVKTEATSIQTAQDEAAANQLMQPQFNGGKRSRAAPDNGGDGEGQPAEGDAKADSSSGAAAVAVVAAAAAAVAEDGYSWRKYGQKQVKHSEYPRSYYKCTHASCAVKKKVERSHEGHVTEIIYKGTHNHPKPAASRRPPVHPPPPSPATTTTTPLPPGDAQADHAPDGGGGSTPVGAGQAGAEWHNGGVVGGEGLVDATSSPSVPGELCESTASMQVHEGAAAAQLGESPEGVDVTSAVSDEVDRDDKATHVLPLAAAAADGESDELERKRRKLDSCATMDMSTASRAVREPRVVIQTTSEVDILDDGYRWRKYGQKVVKGNPNPSSSSSMDADRSLVVVVVIRSYYKCTHPGCLVRKHVERASHDLKSVITTYEGKHNHEVPAARNSGHPAGSASPGGGAGSSSQPHGVGVGGRRPEVPSVQESLMRLGGGCGAAPFPPHFGLHLPPPPPRDPLAPMSNFPYSLGHAPSPALRGLPPPPPPPPSASALAVAGLGGVVEGLKYPMLAPPSVHSLLRHRQGGGMEAVVVPKAEVKQEAMRPAAAVAGAGRGAAVYQQAMSRVSLGNQL*

>LOC_Os04g46060.1

MYACMEGSQLETACLPAALYAPLCPYTPPSPPSFLAPLPSLQHKLPQLPQLVHDHAAATGTNHGVMFSSDHGCLYPLLPGIPFCLDSGCGAAACDDDKPAGFAHLGSAEADTSAAAARVDSEIAAAATATTCHGPNSWWKGTEKGKMKVRRKMREPRFCFQTRSDVDVLDDGYKWRKYGQKVVKNSLHPRSYYRCTHNNCRVKKRVERLSEDCRMVITTYEGRHTHTPCSDDATTGAAGDHTASCAFTSF*

>LOC_Os04g50920.1

MPPCVWGSHTPLSLLSPPAAWAGERMEGDQAGGDLTDIVRAGGGAMPGSVVVDLPSTAAEWQLPAEPMLFPPPPSLSSTTDGCGAGGAAGADIFGGGGGDLFSGLVDPFSSDYSSGADFLDAMPDAMAKVGFDTAVGGGCGGGGGGGGGSGGHLLDMSRKPLLPRGMPMAAVGGLAAPRVMPSPLSPRAIRPYPPISAGDMMKLGITAGQAAGCAIDAAVAGMQMSSPRSGGIKRRKNQARKVVCIPAPTAAGGRPSGEVVPSDLWAWRKYGQKPIKGSPYPRGYYRCSSSKGCSARKQVERSRTDPNMLVITYTSEHNHPWPTQRNALAGSTRSHHSKNSGGGGGSGSKGSQNDKSQQQPSVKEEQKDQATTATTTTTSTITTTNSASPVVVKEEEAALAGSSEALELERVMDTTAAGVVDHSELMDHVFSESYKPMIPETGQPDDFFADLAELESDPMSLIFSKEYMEAKPSGGDHAQEKAMAKELDPFDMLDWSTTTNSSAGSSFEQGKRG*

>LOC_Os04g51560.1

MAVDLMGCYAPRRADDQLAIQEAATAGLRSLEMLVSSLSSSSQAAGAHKASPQQQPFGEIADQAVSKFRKVISILDRTGHARFRRGPVESSAPAAPVAAAPPPPPPPPAPVAAALAPTSSQPQTLTLDFTKPNLTMSAATSVTSTSFFSSVTAGEGSVSKGRSLLSSGKPPLSGHKRKPCAGGHSEATANGGRCHCSKRRKNRVKRTIRVPAISSKIADIPPDEYSWRKYGQKPIKGSPYPRGYYKCSTVRGCPARKHVERATDDPAMLVVTYEGEHRHTPGPLPAPPAAAAVAAMPVSVAVSTGNGHV*

>LOC_Os05g03900.1

MTKMSSMKADGSVPKRRRQDVQKVVVSLKDHKVEQGPPADSWSWRKYGQKPIKGSPHPRYHHLALIPHTYTAPNVLVISQSFACALILYITDNFTRIRLSDQLLVRFFSLYQSIIFALFVDHAVSRGYYKCSSYRGCPARKQVDKCRNDASLLIITYTSDHNHDNYAATTTNSVQEQAHNPDTSDPLSNGMSLAEVVTVASSKLSGEEESCDFFDELEELPVSASPLPSLSFMVQECSFSDARTLL*

>LOC_Os05g03900.2

MTKMSSMKADGSVPKRRRQDVQKVVVSLKDHKVEQGPPADSWSWRKYGQKPIKGSPHPRYHHLALIPHTYTAPNVLVISQSFACALILYITDNFTRIRLSDQLLVRFFSLYQSIIFALFVDHAVSRGYYKCSSYRGCPARKQVDKCRNDASLLIITYTSDHNHDNYAATTTNSVQEQAHNPDTSDPLSNGMSLAEVVTVASSKLSGEEESCDFFDELEELPVSASPLPSLSFMVQECSFSDARTLL*

>LOC_Os05g04640.1

MEMMVQKQRHEEGEEERGGLCAREIKELDFFSAAGAGAGRRDDDDVLRADGISSSHAGFMVSTALDLLTAVNDGDHHEEKKGQSNIHQSKQMDAAATTVEGELRQAGEENRRLRRRLEELTSSYGALYHQLVQAQQLHTKHQQQAPIAGVQLLDALAAASPASHRRRAAAAVDGDRTADSDGGEGDENVSPSLGSKRPAAAATLTRLTPESGSGGENNGGGEQAPAAEMAPCRKARVSVRARSEAPMISDGCQWRKYGQKMAKGNPCPRAYYRCTMASQCPVRKQVQRCAEDKSILITTYEGTHSHPLPPAAAAMAKTTSAAAAMLLSGPAVSRDALFAAHHHVVAPPPFFHHPYAGSTMATLSASAPFPTITLDLTQPPPTTTTTAAAAMLQLHRPYAFSSLPFSMYGAGGGSHRPPVVLPPPSSVVETMTAAITRDPNFTTAVAAALSSIMAGGGAQARTPPRGGSDAAGDINGGGGADHATAGARAAAAATQPCGTSPT*

>LOC_Os05g09020.1

MAASVGLNPEAFFFSNSYSYSSSPFMASYTPEFSAAAIDANLFSGELDFDCSLPAPAQEYPENENTMMRYESEEKMRARVNGRIGFRTRSEVEILDDGFKWRKYGKKAVKNSPNPRNYYRCSTEGCNVKKRVERDREDHRYVITTYDGVHNHASPAAAAAALQYAAAAGDYYSPPLSSAGSPPAAYSAGGSLLF*

>LOC_Os05g09020.2

MRYESEEKMRARVNGRIGFRTRSEVEILDDGFKWRKYGKKAVKNSPNPRNYYRCSTEGCNVKKRVERDREDHRYVITTYDGVHNHASPAAAAAALQYAAAAGDYYSPPLSSAGSPPAAYSAGGSLLF*

>LOC_Os05g14370.1

MKNKGSSCRYLPHSSAPCATDGARCFRCDHRPAIDEIVREQSLVTQLRAVVLPALETKADDGRAEIVAQLFGSILDCSRKVISALNSRYVGESPPDDDEIVDKRRAKRKNSEGKKGDDQVKVKPHEHKRSRRYTNSTSQITAVPHYDGHQWRKYGQKNINNSNHQRSYYRCSYKHEQNCKATKTVQQLDSAGETIMYTVVYYGQHTCKTNMSNAPLHVVETSTTQSISTTCCSDDLGDYSQKMENMHTPELAEVCSDELGSYHAIIGAEHSALGLEDEHMHKLLDTFACGALDLDSWEIDAIVRSGFC*

>LOC_Os05g25770.1

MTSSMSPAPAPAYAQVMEDMEKGKELAAQLQGLLRDSPEAGRFVDQILHTFSRAMRALDKAAVSAAGGEGSEVQSEVTCGGGASAGGKRKAPAADRKANCRRRTQQSSGNSVVVKNLDDGQAWRKYGQKEIQNSKHPKAYFRCTHKYDQLCTAQRQVQRCDDDPASYRVTYIGEHTCRDPATAPIIAAHVIHQVAAGDNDDGCGGLQAGSRLISFVAAPAAPVDAAAAPTTSTITTVTAPGPLLQPLKVEGGVGSSDQEEVLSSLTPGSSAARGGGGGGGVAGPFGPDQGDVTSSLHWSYDAVAGMEFFKNDEVVFDLDDIMGLSF*

>LOC_Os05g27730.1

MASSTGGLDHGFTFTPPPFITSFTELLSGGGGDLLGAGGEERSPRGFSRGGARVGGGVPKFKSAQPPSLPLSPPPVSPSSYFAIPPGLSPTELLDSPVLLSSSHILASPTTGAIPAQRYDWKASADLIASQQDDSRGDFSFHTNSDAMAAQPASFPSFKEQEQQVVESSKNGAAAASSNKSGGGGNNKLEDGYNWRKYGQKQVKGSENPRSYYKCTYNGCSMKKKVERSLADGRITQIVYKGAHNHPKPLSTRRNASSCATAAACADDLAAPGAGADQYSAATPENSSVTFGDDEADNASHRSEGDEPEAKRWKEDADNEGSSGGMGGGAGGKPVREPRLVVQTLSDIDILDDGFRWRKYGQKVVKGNPNPRSYYKCTTVGCPVRKHVERASHDTRAVITTYEGKHNHDVPVGRGGGGGRAPAPAPPTSGAIRPSAVAAAQQGPYTLEMLPNPAGLYGGYGAGAGGAAFPRTKDERRDDLFVESLLC*

>LOC_Os05g39720.1

MTAAPGSLPLVNSRPVSLSLAASRSSFSSLLSGGAGSSLNLMTPPSSLPPSSPSSYFGGVSSSGFLDSPILLTPSLFPSPTTTGALFSWITTATATAAIAPESQVQGGVKDEQQQYSDFTFLPTASTAPATTMAGATATTSNSFMQDSMLMAPLGGDPYNGEQQQPWSYQEPTMDADTRPAEFTSSAAAGDVAGNGSYSQVAAPAAAGGFRQQSRRSSDDGYNWRKYGQKQMKGSENPRSYYKCTFPGCPTKKKVEQSPDGQVTEIVYKGAHSHPKPPQNGRGRGGSGYALHGGAASDAYSSADALSGTPVATPENSSASFGDDEAVNGVSSSLRVASSVGGGEDLDDDEPDSKRWRRDGGDGEGVSLVAGNRTVREPRVVVQTMSDIDILDDGYRWRKYGQKVVKGNPNPRSYYKCTTAGCPVRKHVERASNDLRAVITTYEGKHNHDVPAARGSAAAALYRATPPPQASNAGMMPTTAQPSSYLQGGGGVLPAGGYGASYGGAPTTTQPANGGGFAALSGRFDDDATGASYSYTSQQQQQPNDAVYYASRAKDEPRDDGIMSFFEQPLLF*

>LOC_Os05g40060.1

MALIATGATATATAAPVASPAASSMASELMAQGRESAAVLEALLHGASLPPAHGGAHALAAEILRCCDRALAALRAGGDAESSSADTKRKPATAQPSTRRRRRATASGGGAAAAAEPARVEKARTSEDGFLWRKYGQKEIKNSKHPRLYYRCSYKDDHGCTATKQVQQSEEDPSLYVITYFGDHTCSCQTAAAAAMDDDDDDENSQHFVINFGPATASRSGSPPLLYDDGDDGDVWRETAATPPSSRQSRCSPEGDGEESGVKMSKEEPVDSCPGPSAVSSPADVVSCSSPAMEPDLLGCLNWDDDFGDSSFVDADEFMNFDEIDLFQIYS*

>LOC_Os05g40070.1

MARRLPKSERSPSPPPPPPGDQRDAAIQELSKGSELATQLMAQLELIPERELDGRRDDALANVRSLSMSLSSSLYALRSERREHYYCGSSSSSGGAGPAAVTSVSGAGGERKTKRRRGKHGEELIETVFITTTPENDGFHWRKYGEKNILNSEFRKLYYRCGYSDERKCQAKKYVQQENNKHPPEFRVTLTNEHTCNTVFQDQPSSSSTNSQVLDFTKASISSSLMDSHVGAPILKEEEEEEVPSIDESTRIMSTIMRNYGSYGDYDESSPQPWNGAGWK*

>LOC_Os05g40080.1

MALTMVSLAAIAGKPTAASAQLVAEGRESAARLYALLVGSSALHGPVGLAEQILLCFDRALAKLHGVNLAGAEDDDDAAGNGNGRKRKPGRGRGLTAASAAASSKRMRVSNAGGNGARIERKATMDDKFLWRKYGQKEIKNSKHPRFYYRCSYKDDHGCTATKQVQQSETADDDTASPVYIITYFGEHTCRHGDDAAAMVVDGGEEEDQLSPAQMVISFASSNGGDASVSWPCSGDDAQNNSETSHESSPPEAPAGEEERLRPCTAAGVSDEPIMESTPPAPELLADLKPMDGCLLDGESLFGMDELVYFHELSAALGLLDRDWGAPV*

>LOC_Os05g45230.1

MDGLEAAAGDQQHGRLLIPQLPAAYLASSSMAALSPAGDDWAASLILPDGGSAAAGVGEDDLGGGVMAAAAAESSCGGSSTVTSSGVTEAAAAAATTTRRGRGNGKKAGGGGRTPRFAFHTRSENDILDDGYRWRKYGQKAVKNSDFPSDDELLLFSDVDNTQTATENLRFIPLGRVYITG*

>LOC_Os05g46020.1

MAAVGAHAAVYHHPVSGLSAPAGDAAYSMSSYFSHGGSSTSSSASSFSAALAAATTPPLPDPSGSQFDISEFFFDDAPPAAVFNGAPTAALPDGAAANATRSAAEAVPAPAPAAVERPRTERIAFRTKSEIEILDDGYKWRKYGKKSVKNSPNPRNYYRCSTEGCNVKKRVERDKDDPSYVVTTYEGTHNHVSPSTVYYASQDAASGRFFVAGTQPPGSLN*

>LOC_Os05g49100.1

MSGGGGGGEGFPFHDELASLFAERPPNGAMPGMLQQQQPWSFIDYHHHLMQESAPTTPPLDYEAFAGEFDDDVAPLEEVKRELVVDGVGLFPGGGASAAAAAAAVAGPMTPNSMSVSSTSSEACGVGGGAGGDEESAGKCKKEEEGDGGDDDGKEGSSTTKGDGDGEDKNKKGGKGKGKGEKRPRQPRFAFMTKSEVDHLEDGYRWRKYGQKAVKNSPFPRSYYRCTTQKCPVKKRVERSYQDAAVVITTYEGKHTHPIPATLRGTAHLLGAAAAAHHHGGLQYHHPGHFAAAVGHRLPPQPHDALGGGLLAPPHAQHLHAMQHQMQLAAAAAASGGSLHAAAMQQMPQPDHAGLVAIIASTTGASTTPPPPPATGSAAAATTPLRMQHFMAQDYGLLQDMFIPSPFLHNDDANNNNHR*

>LOC_Os05g49210.1

MESYVGVKGKNVVGGGDVGREMPVAPPSSSSAAVGMVEFPAAAAGLGYAGMTAKEAGGGYQERRVVVGEMDFFKTAEKRGERKEPPPATATAAASGHAGASPDDLSLNKDDLTINMGLLVGRRRNSGSEESIVDDGGVSSNDEEHREAKAALAVTKAEIGRLSEENKRLKNMLSNVTTKYNSLQMQFVTLMQQRRSVLAAPIHQQELLDPEKKEQEGSQQQQQQLIPRQFISLGSASLQPDVEAPHSVVVVGGDVCAPSSSNPDAAVPAMMPLPHFDHHNHHHPIHGGRERGSSPAEADHHRHHQQEQPPPPPQQQQQLPPSWLPADKVPRFLPGKGPEPVPEAATMRKARVSVRARSDAPMISDGCQWRKYGQKMAKGNPCPRAYYRCTMAAGCPVRKQVQRCAEDRTVLITTYEGNHNHPLPPAAMAMASTTAAAASMLLSGSMPSADGSLMAGSNFLARAVLPCSSTVATISASAPFPTVTLDLTQTAPPPPPASSTQPQPPRPEPAQLQAALAEAARPVALPQLFGQKLYDQSKLSAVQAVAGTKGSDGGALADTVNAATAAIASDPNFTAVLAAALTSYIGSRSGSGGAGAGGSSGTVQPLMSGGGDSCSRDDKIGEQNS*

>LOC_Os05g49620.1

MVELCGGEGEGQIMLATELAQLRAMARELEAKMDPDRVAARELCRALASSVDRSIRLAASCFPPPEHPPPAAGNAGRDAAFKKRKGMAKVRRQVRVTSVQDTASLDDGLSWRKYGQKDILGAKYPRAYFRCTHRHTQGCNATKQVQRADGDPLLFDVVYLGDHTCGQAAVAAAAQSAPPEHAGQEQQRQSSLLAAGTEGIHQQVVAEPMAAPFLFTSTAAGGVDDGYFSFISPANSDCQFSSDFSAGSVGVDMDHEARFEDLFSSTLEFFQSEIQNL*

>LOC_Os05g50610.1

MSGPGGGGHGLYEDHPAAAGFLPFDHDDDVVASFFFGRSAASGGGAGAGAGAGDDDGVGLITPYSSITDYLQGFLQDPVYASSPLGGDAAVKHETVVDHPSQAGGVAAAPATPNSSVLSSSSEAAGGDDLRRCKKGRRPEDEEEEEIDDEGSAVQSCKTNKMKNKKGAKKEREPRVAFMTKSEVDHLEDGYRWRKYGQKAVKNSSYPSYYRCTAPRCGVKKRVERSEQDPSMVITTYEGQHTHPSPVSYHMHRQQGLMHVSARGVMPGAAGAYQFGAPPPPLLGFDEALAARSQDTVLVSQEDRLSQQQRSLYYRVDVQSCWAADRSRAHNCITKGKRKPGLGPNNTGATWELGRNPKTHRSRLAPGRTPHQQDPLRLLQRLGGDYLYRARRGVHFTHTTLLTRITQPVGISHVGRPPSFHDKAFPKPIQVYHMPTRGVPDQQQESVLKCESGKTVHPAQDVQRWLALLEIWSLILEILALRVFGLRNYTRNGTTQQTAKMKP*

>LOC_Os05g50610.2

MSGPGGGGHGLYEDHPAAAGFLPFDHDDDVVASFFFGRSAASGGGAGAGAGAGDDDGVGLITPYSSITDYLQGFLQDPVYASSPLGGDAAVKHETVVDHPSQAGGVAAAPATPNSSVLSSSSEAAGGDDLRRCKKGRRPEDEEEEEIDDEGSAVQSCKTNKMKNKKGAKKEREPRVAFMTKSEVDHLEDGYRWRKYGQKAVKNSSYPRSYYRCTAPRCGVKKRVERSEQDPSMVITTYEGQHTHPSPVSYHMHRQQGLMHVSARGVMPGAAGAYQFGAPPPPLLGFDEALAARVRMTMNQQQQQQQLGFVPSIHAAAARPTMPPLHLYTAQQDLFLP*

>LOC_Os05g50700.1

MTITHASSLSRFHPLLKKLIMEYSNDWDLQALVRSCGTAVADSEPEPPAAPSTTRRAEAETVFVGRAGGVPEFVGQPVRSSAASFYDLEYLDLYHERPRAPFLVTAPSTSRERGEGGEHEVLISFPAIASTSGQGRKQPGRKPGVRTARPKRSKKSQLKKVVCEVPVADGGVSTDLWAWRKYGQKPIKGSPYPRGYYKCSSLKACMARKMVERSPEKPGVLVITYIAEHCHAVPTQLNSLAGTTRNNKPASPDQQQQQQPSPGGASTDEAAAAAAKTEDSADTTCSMADDENDLWAPVEMDMNDFFGPFDDDLDHFLDDDAVLGRRLSL*

>LOC_Os06g05380.1

MGEVREENERLKTLLSRISHDYRSLQTHFYDVLQQGRAKKLPDSPATDIEEPELVSLRLGTSTSKCKKEDKSTTSSEVKGSTEDFLKIKGGLSLGLSDCRVDANNSEKVQPDVMTLSPEGSFEDARDDTAETTEQWPPSKMLKNLRSVGAEAEDDIAPQPQVKKARVSVRARCDAPTMNDGCQWRKYGQKIAKGNPCPRAYYRCTVAAGCPVRKQVQRCADDMSILITTYEGTHNHPLSVSATAMASTTSAAASMLISGSSSTSLAAYPAAAASPALAFDASSKPPLIGGRPFFLPTAAAAAITSTPSYPTITLDLTSPAAAATSSHAAFSLSNRFSHTRYPSTGFTFSGSGPSSAPWPGYLSYGASLSAHPYNAGGGKSSSSFEAALSSINGSRQQGGGGGGGSAPPLYQMQQKAAAAAPPPPSVITDTIAKAITADPSFHTALAAAITSYVGKKGSPPASGGEDSKVGLKWGEHLGLGLTHSSLSTAAAAAASSSSQMFLQPSLGLSGSTTSASTSPVANREQAH*

>LOC_Os06g06360.1

MDGGDIHLLLSILADGEEQARQLGEPAAAADDEYHGGGRGEEYYRGVARQLQGTLARAMGIARAIEAAAFAGGGGGGGASGSRGTTGDRSDSPRSADESSGRTARDAAVAQQERHHDTIKRRKGLPRWTEKFRVPDASLEATPDDGFSWRKYGQKDILGAKFPRGYYRCTYRNAQGCPATKQVQRSDADLAVFDVTYQGAHTCHQKQRRAAAAGDQPPPPPPQADPSVELLVNFRHGLKVETNGLAPPPPPPPTTTTNFHDDQHFCFPSMPPFHAGVGPPPPPDDALGGGCNNFSSPPFVSPAGSAAGESYFSMEHSYEPRGGGGHFVMSRGDSSELHEVVSAAASSSAVVDPAAAAGGFDYPLYHGEVDPHLPFPPLFGHASMYGQYRDA*

>LOC_Os06g30860.1

MDGDAWWYPGGGGGGGSNNWDLGAVVRFGCGGGRVSPAAALLGEAWEYDDDPFSSFLAPPMTAQQAALPAVWEEGDDGDAAWMAPLPGLQTGGGWGDQAPMVVDELCGALVVAPPPPPKQQEVLQVQQQPPPADNTQPTTYQQGSGGDGESTRAGGSRKKQTRKEVVRVAASGPAPDLWAWRKYGQKPIKGSPYPRGYYRCSSNKNCAARKQVERCRFDPSFLLLTYTGAHSGHDVPLHRNSLAGTTRHKPPPPPPLPSAADKSPATAAEAATASQSPGLSPTTPLRASSMELHGEDDAEAELQVEEDDMAIDDEDDDDVADETISTVPWGTPISDAIIAASYEWR*

>LOC_Os06g44010.1

MAKMLPPPSQSVPSRPPSWLYIPPRRRHGTFTSSCAFRLSPSSPSSPPPPVLDFQYIQFMDSWIEQTSLSLDLNVGLPSTARRSSAPAAPIKVLVEENFLSFKKDHEVEALEAELRRASEENKKLTEMLRAVVAKYTELQGQVNDMMSAAAAAAVNAGNHQSSTSEGGSVSPSRKRIRSVDSLDDAAHHRKPSPPFVAAAAAAAYASPDQMECTSAAAAAAAKRVVREDCKPKVSKRFVHADPSDLSLVVKDGYQWRKYGQKVTKDNPCPRAYFRCSFAPACPVKKKVQRSADDNTVLVATYEGEHNHAQPPHHDAGSKTAAAAKHSQHQPPPSAAAAVVRQQQEQAAAAGPSTEVAARKNLAEQMAATLTRDPGFKAALVTALSGRILELSPTKN*

>LOC_Os07g02060.1

MAMAGAGDWPFAADEAYADSSAIFAELGWANGLAVVDAVGELLPPLDPPGELATPPPPPLDLPETPAGSSADGAASSCSTDDADGGKPAAASTEAASKSLTPGKKRARQPRFAFMTKSEIDHLEDGYRWRKYGQKAVKNSPFPRSYYRCTNSKCTVKKRVERSSDDPSVVITTYEGQHSHHTVTFPRAAATAAGFSHIHAMAALAAAPFSAHQQLYSNLQPPPPTMPLAATTPASSSSLLQLPLHCNHELQVVASCGGYPSSSSSPPASVLPVDKGLLDDMVPRAMRHDG*

>LOC_Os07g27670.1

MTYLEFLQKNQWGQEASVGADGVQVDDVCAGSLGGHATAGVWPGRWREIGGRACAWTAAEGLENGGQESRADGSSRRIILELGDRDDSYPWRKYGQKDILGARFARSYYRCAQMLGCTARKQVQQSDDDPSRLEITYIGLHTCGGDRPSSPAPTNPADGPRCDAATSSHRLLPSALQQKLEEHVPAASDDMMMACTPSWLFIPSPACSQSELLSEGEVPELRVVRQEPYDPVELVEEHKKPSDADEDSLALHNSVVPDFM*

>LOC_Os07g39480.1

MADSPNPSSGDHPAGVGGSPEKQPPVDRRVAALAAGAAGAGARYKAMSPARLPISREPCLTIPAGFSPSALLESPVLLTNFKVEPSPTTGTLSMAAIMNKSANPDILPSPRDKTSGSTHEDGGSRDFEFKPHLNSSSQSTASAINDPKKHETSMKNESLNTALSSDDMMIDNIPLCSRESTLAVNISSAPSQLVGMVGLTDSSPAEVGTSELHQMNSSGNAMQESQPESVAEKSAEDGYNWRKYGQKHVKGSENPRSYYKCTHPNCDVKKLLERSLDGQITEVVYKGRHNHPKPQPNRRLSAGAVPPIQGEERYDGVATTDDKSSNVLSILGNAVHTAGMIEPVPGSASDDDNDAGGGRPYPGDDAVEDDDLESKRRKMESAAIDAALMGKPNREPRVVVQTVSEVDILDDGYRWRKYGQKVVKGNPNPRSYYKCTNTGCPVRKHVERASHDPKSVITTYEGKHNHEVPASRNASHEMSTPPMKPVVHPINSNMQGLGGMMRACEPRTFPNQYSQAAESDTISLDLGVGISPNHSDATNQLQSSVSDQMQYQMQPMGSVYSNMGLPAMAMPTMAGNAASNIYGSREEKPSEGFTFKATPMDHSANLCYSTAGNLVMGP*

>LOC_Os07g40570.1

MAAARRVAGGGGGSLWGPPQPPPSTGGGIPQLPAAAAAPVEGLLDAPFSSSSGGGGGGWPPPPPPLSGTAVLIGYPQGNFETFPQQDLVPLTAQEVHSKCITFGRAENLPFIPLATSALVSQHTGSSSVNVTPLQEILTSPSQISNVNTESIGVLQGLPASSIVLDRPTDDGYNWRKYGQKAVKGGEYPKSYYKCTHLNCLVRKNVEHSADGRIVQIIYRGQHTHERPSKRRFKDCGGISDDLDDFSGTTGTSVRSQPDYDDYCRKPIIPSGTMVAPLVKKIEDGDDQLSGSSDNQDEHDDEVRTSDGASGDASANERNVPAPGQKIIVSTTSEIDLLDDGYRWRKYGQKVVKGNPYPRSYYKCTYLGCDVKKQVERSVEEPNAVITTYEGKHIHDVPAARNKSHVVANASLLQNTKSNTYCTEQSYTTITC*

>LOC_Os07g48260.1

MASPDGGVGDGGAEPHEVMDDLLEMREQAAMLHSMLHGTSPSSCAAAASTRQLNQLIDGVMSRLQSSSLSVMSPGGGGGRRGSGGRKKKGAKAVAGPHRRSSSGRRRSKSPFVRMVTTKELEDGRQWRKYGQKHIQDSPNNPRSYYRCTHRPDQGCMATKQVQTSESNSSEFVISYYGEHTCSDPSTIPFVVEAEAPAADYANLISFGSSGGASTSRVDPLRQSRHRLMAEAVDPTPSCSFANCHSPVLSSECASEAAALSSSLPLSAVVGSAVTTPSTSIVGSAPADYDWPSGLAGGDMAGSFPSSPSSLGFMTGSFGNLPGDDDDMFGFDP*

>LOC_Os08g09800.1

MENQSGQAQDGMADQRFRSWLTEQFNTQPYAGSSSNLAVMQMQVLPSHTTSQIIDTHPPEYDGYNWRICGQKVVQGGCHQKFYYECSQANCGAEKSVTRSADGQIKKTVCKGSHNHPLSSERVFGEGSATLDAIPVGEILQAAGVIRPSVAMPMNEEEDELQSGLGDSEEDDANEARVDGDGAAADANAIERHGAAQDITAQTATEVDVTGNGCQQRKNYCRSENRRSKSKVWKEFTAVLSVGKIQSAECKHCKKCLSGKSSGGTSHLRRHLKICPGQCRDTRIQQKWSSSRLDSSDANNWEFDQETSLELLTRALVSNLCPFSVTTSANFRKFFAGICPTYNIVPQAAIEEKFLSIFQNEKMKLKEEIALKPGGVFLSVTRWAPECKQFLCFTVHFIDKEWKLNRKIIRFQFSGDEALEAEHYVSILSNWKSFSNIRNASYEYGTEKTNKALIKAAVQDWNLEKKLLGIALPTNIGNEVILDLEETMTAAGQNFLLAKYKLLIVPCMINALHGLFGYTLERYVLEASREWFEYMTCSAIRLEKYKEILLRLHLSQPSFGSQKWHLTYYLFEAALQFVKEFPNPDAAHLKMFLRKPFPERLEATKNFCDLARPIYHAIDVLSRQNVAFNSHFHVIWSLGTVLKESSKKINIKRIIDIDDMLKKFDNLWRKCYVWLSLAVVLDPRFKLRYLEQCFKQAFGTGAKLCILEVRGKIYELFLQYSCNADQQSGELVNHWNNDLQMDRDGNDSLHGTDQNDIGQSALGEFRELTLYLEGGLCPQNEQFDILKWWKDNALTYPTLARLARDILAIPGSAVSAESAFDETDERVSLFNRKLSPEIVEALICTQDWIKSSETGDENGGS*

>LOC_Os08g09810.1

MENNQSGQAQDGMADQRFRSWLTEQLNMQQQNAGCSSNPAPAVMQMPALPSHTDYGGIYPNPTEYDGYNWRMCGQKLVQGGCHQKFYYECSQANCGAEKSVTRSADGQIKKTVCKGSHNHPRSSERVFGDGSATLDAILVGEILQAAGVIRPSVAMPRNEEEDGLQSGSSDSGDDDASEARAAGDDNAIRHVPAAAAQDTTAHNTIDVDVLGNSSQQLMGSFLTASQPLDSFLSSEKRKSKSKVWEDFTAVFSGGKVQSAECKHCKKCLSGKTSGGTSHLRRHLKICPAQFRTTRLQQEGSSSILDSSAANNRKFDQETSLELLIRGLVSNHCSYLVPSSANFRQFLVAICPDYNMVPQAAFEEKFLSFFHNEKMKLKEKIELTPGGVFLSVTKQYVEFKTFVCITVHFIDNEWKMNRKIISYGYGGYPDGADYYVGILTNWKSYLDIRDSLDYNFEEIDSSLIKEAVQDWNLEHKLLGLALHKNFRNNVTSDLEECMAGEVQNYLLAKYKLLTVPCMIDALHDFFGYDVGNFVKEISKEWFEYMTCSALCLEKYKEILSRMHLNKPSLGSQKWHLTFYLFEAALQFNKEFPNPEEMDSQMDIRKPSPQRLEATKNFCDLVRPIYHAIDLLSRQYVTSNSHFHALWRVGIALGESSRKLNMKCIINVDYMKKRFDILWRKCYVWLSLAVFLDPRFKLRYLEQCFTQVSSSGCAKLFVLEVRAKIYELFLQYSCNVDWQTGELLNHRSNDLQMDRHGNDSLHGTDKNDIEQGSNGEFRELTSYIEGELYPQNDQFDILKWWKDNASTYPTLARLARDILAIPGSAVSAEYAFNKTGERVILFNQKMSPEIVEALICTQDWIKSSETGDKNGGS*

>LOC_Os08g09840.1

MADQGFRPFSALMSAPSTAQQHVGSSFSTAVVQVAAAQQSHTDHGNICLADDDGYYWRMTGQSTTQGESSPTILSHYQCAQANCVVQKTVAYTADVETFYRGRHNHLRQSDRLEPMSQVGVLVEASDAAGAAAGPSVPETENGDDQSSGSSDRNEDDAGDVEMDEDAAAGDPNAMQRRKLKSKVWKEFKLVFKDGKLHTAICNHCKLRLVAETRNGTSHLRRHLKICPEKAGTSRVQKKRRSSTSQSQPDLPVSENLENGQENPSQNLENGQENPLEEFMRATVLKLCPFPAMYRASFASFLAGRNPAPNMVPQTTVEDKFISVYEKEKLKLKEKIIATPGGVFLSVNKWYSGSYETGIVCLTVHFIDEEWKINRKTIRCCLSESDGLDLNLFPHWQSEIANYEDDDKMVLKKVVRDWCLEPKLLGVTLEGSVDKKATISLEDDLTTGKNYLVAKCKLLTIPCMVDGLDDLMQYTVGREVRSMWSRYMTNTPERKLKCQEVVSQLQLDRPSFGSKYRYLTFYWCEAALQFIKSFPLSNGSERPSLDDLEATENFCKIARAIYHATKAFYEPYNLTFNSYFHVIWSLRATLQELPRIKNIERVIKVKNMQEKFDNHWKKWYLWLSIAVVLDPRYKLAFIELRFREAFSQDAGTYISEVRAKFYELYIQYSHVNEQSNEILNQGNNGSGTQISAPLHKQRTNYTIAQAALEEFKELFEYLGGGLCPQNDSFDILKWWKDNSAAYPSLAKMARDILAIPGCAVSAESAFNDDSDHRAELFNGKLGPETTEALICAQSWIIKSSGTADADNGNNITLS*

>LOC_Os08g09900.1

MENQSGQPQYAMADQGFHPFSPFMLAPSTTMQQHVGSSSSTPVIQVAALPSHAYYGNIDVADDGFHWRMCGQSTIQGGLCPTVFSYQCALPNCGVRKSITRSADGQTIETVCKGCHNHPRQSLRWLGDGSERLEPISQEIVLLEASDASGAAGGPSVPGTGNGHGQSSGSSDSCRDDDGDLGIDGNASVGDANAVKSGQVPAPAKEITVHSACEVDILNNSVRHENPQPRKKVRSKSTVWEEFEVVLIDGKVQTAECKHCKKGLSAKTSGGTSHLIRHLKICPAQHGTSRVQKKCSSLADLPIVKSWKDDQESSLDEIIRSIVSNLCPFSAMYSASFAQFLAGRNPVLNMVQQATVEEKFLSVFHNEKMKLKEKITATPGGVFLSLGEWQRLFYIQVRVACLTVHFIDEDWKINRKTIRCSLSVFGKSDILSLYPHWQSDIVLAEKVLKEVVQDWGLLDKLLGVTLQRSVDKKAPLHLEDDITGRNYLLSKCRLLSIPCMVDALHELMDSTVLDMESTWSHYMTSSPERKQKYQEILSQLHLDRPSLGSKGWYFTFYFSEAALQFIKSFPLPDAKPNCQSGPWEPSFDDLEATENYCKIARSAYRVIKVVSGPHNMTFNSYFHVIWSLRAAIQELPSIKNIGRVFDVAYMQKKFDRNWKKWYLWLSIAVVLDPRYKLGFIELCFRQAFSHVAGMYFSEVRAKLHELYIQYSYVNEQSKEILDHKNNCSDIQISAPLHNKGQNSTTAQAAVEEFKELYEYLGGGLCTQDDSFDILKWWRGNSSAYPTLAMMARDILAIPGCAVSTESAFDQCDQRAELFDGKLRPETTEALICAQSWIKSSGTADADDGNKNTSF*

>LOC_Os08g13840.1

MAVDLMGFSPRGGCRPSVETEQLAFQEAAAAGLRSLELLVSSLSAGGEHHHRRRPQEKQSSPPLGEIADQAVSRFRKVISILDRTGHARFRRGPVVGAAAAEAAAAAASASPSSSPVSPPLPPVTTQPAAAVKSLTLDFTNPAKVAAASVTSTSFFSSVTAGGDGSVSKGRSLVSSGKPPLAGGVKRKHPHPPCAAAGDGHGHGAGHAHAHGGCHCSKKRKQRVRRTVRVAAASARVADIPADEYSWRKYGQKPIKGSPYPRGYYRCSTVKGCPARKHVERAADDPATLVVTYEGDHRHSPPPPPLV*

>LOC_Os08g13840.2

MAVDLMGFSPRGGCRPSVETEQLAFQEAAAAGLRSLELLVSSLSAGGEHHHRRRPQEKQSSPPLGEIADQAVSRFRKVISILDRTGHARFRRGPVVGAAAAEAAAAAASASPSSSPVSPPLPPVTTQPAAAVKSLTLDFTNPAKVAAASVTSTSFFSSVTAGGDGSVSKGRSLVSSGKPPLAGGVKRKHPHPPCAAAGDGHGHGAGHAHAHGGCHCSKKRKQRVRRTVRVAAASARVADIPADEYSWRKYGQKPIKGSPYPRGYYRCSTVKGCPARKHVERAADDPATLVVTYEGDHRHSPPPPPLV*

>LOC_Os08g17400.1

MPDGYPAAPPGMERPAPEAAAAAAEPPGAKAEEEEEGPKDVGVKGTGKEKETEKVEAEKEMEGKGKGKESMEVEEGKEKEGKGKAKEKEKETKVKVKEEGGEEKEKGKVEVVEAKRRPAGVGAETPILAVPMVAVPCFLASPAFAGHFSMSHQAALASVTAQAQIQLQSPTTPYSEGLPSPFPITPKAVMPLQRSPSGTEGSVRRSVLEKSASFQSRPHNHVSVNMVGDGFNWRKYGQKQVKSSENSRSYYRCTNSNCLAKKKVEHCPDGRVVEIIYRGTHNHEPPQKTRFVKERVAHITASSGDDETLGLVNNEIIESPSPGCKLEPGAVSEASEQQLFCSSDCEGDAGNKSEDDHPSTEPQPKRSRIIETSTPLTPVLRTVREQKIIVQAGKTSDGYRWRKYGQKIVKGNPNPRSYYRCTHDGCPVRKHVEKAPDDDNNIVVTYEGKHNHDQPFRNNSESKDGPVPMIIPAETTSEQPSTMTSTSEQKQPISLLKDGGDEPMKGKTSEIGGEKAVESAQTLISIKTNPDDMKNTLLKDTSAVVPVQNN*

>LOC_Os08g29660.1

MEGVVDGSGAQLVVAELVRVQGLLRQLEAHLSAPCSVELCRGLVAQIVALTDRSIGIATRSFSSASGGGAHFADTAPPMPALTSCTPSPLSDGSDHQPFRTTNAKKRKTTARWTSQVRVSAAGGAEGPADDGHSWRKYGQKDILGAKHPRGYYRCTHRNTQGCTATKQVQRTDDDASLFDVVYHGEHTCRPGAASAAAAKRPHAQTLLQSLSASLTVNTDTNTPLTPENRAPAPPLQQQQQQQSVSASPVASDSYGLGGAGYGDWRCCDGDLQEVVSALATVTSAPDHAAMDAADFMSYCFDFDPAVYGGIVGTPSFFL*

>LOC_Os08g38990.1

MDGTNNHGALMDDWMLPSPSPRTLMSSFLNEEFSSGPFSDIFCDNGSNKHQDGLGKSKAFIDSSREETAQLAKKFESNLFGANQKSSSNGCLSERMAARTGFGVLKIDTSRVGYSTPIRSPVTIPPGVSPRELLESPVFLPNAIAQPSPTTGKLPFLMHSNVKPSIPKKTEDETRHDRVFFFQPILGSKPPTCPVAEKGFSVNHQNQPSVTDNHQELSLQSSSTAAKDFTSATIVKPKTSDSMLDNDDHPSPANDQEENATNKNEEYSSDLIITPAEDGYNWRKYGQKQVKNSEHPRSYYKCTFTNCAVKKVERSQDGQITEIVYKGSHNHPLPPSNRRPNVPFSHFNDLRDDHSEKFGSKSGQATATSWENAANGHLQDVGSEVLTKLSASLTTTEHAEKSVMDKQEAVDISSTLSNEEDDRVTHRAPLSLGFDANDDYVEHKRRKMDVYAATSTSTNAIDIGAVASRAIREPRVVVQTTSEVDILDDGYRWRKYGQKVVKGNPNPRSYYKCTHPGCSVRKHVERSSHDLKSVITTYEGKHNHEVPAARNSGHPSSGSAAAPQATNGLLHRRPEPAQGGGGGSLAQFGYGSAGHRPAEQFGAAAAGFSFGMLPRSIATPAPSPAIAVPAMQGYPGLVLPRGEMKVNLLPQSGNAGAAASQQLMGRLPKQHPQM*

>LOC_Os08g38990.2

MDGTNNHGALMDDWMLPSPSPRTLMSSFLNEEFSSGPFSDIFCDNGSNKHQDGLGKSKAFIDSSREETAQLAKKFESNLFGANQKSSSNGCLSERMAARTGFGVLKIDTSRVGYSTPIRSPVTIPPGVSPRELLESPVFLPNAIAQPSPTTGKLPFLMHSNVKPSIPKKTEDETRHDRVFFFQPILGSKPPTCPVAEKGFSVNHQNQPSVTDNHQELSLQSSSTAAKDFTSATIVKPKTSDSMLDNDDHPSPANDQEENATNKNEEYSSDLIITPAEDGYNWRKYGQKQVKNSEHPRSYYKCTFTNCAVKKVERSQDGQITEIVYKGSHNHPLPPSNRRPNVPFSHFNDLRDDHSEKFGSKSGQATATSWENAANGHLQDVGSEVLTKLSASLTTTEHAEKSVMDKQEAVDISSTLSNEEDDRVTHRAPLSLGFDANDDYVEHKRRKMDVYAATSTSTNAIDIGAVASRAIREPRVVVQTTSEVDILDDGYRWRKYGQKVVKGNPNPRSYYKCTHPGCSVRKHVERSSHDLKSVITTYEGKHNHEVPAARNSGHPSSGSAAAPQATNGLLHRRPEPAQGGGGGSLAQFGYGSAGHRPAEQFGAAAAGFSFGMLPRSIATPAPSPAIAVPAMQGYPGLVLPRGEMKVNLLPQSGNAGAAASQQLMGRLPKQHPQM*

>LOC_Os08g38990.3

MDGTNNHGALMDDWMLPSPSPRTLMSSFLNEEFSSGPFSDIFCDNGSNKHQDGLGKSKAFIDSSREETAQLAKKFESNLFGANQKSSSNGCLSERMAARTGFGVLKIDTSRVGYSTPIRSPVTIPPGVSPRELLESPVFLPNAIAQPSPTTGKLPFLMHSNVKPSIPKKTEDETRHDRVFFFQPILGSKPPTCPVAEKGFSVNHQNQPSVTDNHQELSLQSSSTAAKDFTSATIVKPKTSDSMLDNDDHPSPANDQEENATNKNEEYSSDLIITPAEDGYNWRKYGQKQVKNSEHPRSYYKCTFTNCAVKKVERSQDGQITEIVYKGSHNHPLPPSNRRPNVPFSHFNDLRDDHSEKFGSKSGQATATSWENAANGHLQDVGSEVLTKLSASLTTTEHAEKSVMDKQEAVDISSTLSNEEDDRVTHRAPLSLGFDANDDYVEHKRRKMDVYAATSTSTNAIDIGAVASRAIREPRVVVQTTSEVDILDDGYRWRKYGQKVVKGNPNPRSYYKCTHPGCSVRKHVERSSHDLKSVITTYEGKHNHEVPAARNSGHPSSGSAAAPQATNGLLHRRPEPAQGGGGGSLAQFGYGSAGHRPAEQFGAAAAGFSFGMLPRSIATPAPSPAIAVPAMQGYPGLVLPRGEMKVNLLPQSGNAGAAASQQLMGRLPKQHPQM*

>LOC_Os08g38990.4

MDGTNNHGALMDDWMLPSPSPRTLMSSFLNEEFSSGPFSDIFCDNGSNKHQDGLGKSKAFIDSSREETAQLAKKFESNLFGANQKSSSNGCLSERMAARTGFGVLKIDTSRVGYSTPIRSPVTIPPGVSPRELLESPVFLPNAIAQPSPTTGKLPFLMHSNVKPSIPKKTEDETRHDRVFFFQPILGSKPPTCPVAEKGFSVNHQNQPSVTDNHQELSLQSSSTAAKDFTSATIVKPKTSDSMLDNDDHPSPANDQEENATNKNEEYSSDLIITPAEDGYNWRKYGQKQVKNSEHPRSYYKCTFTNCAVKKVERSQDGQITEIVYKGSHNHPLPPSNRRPNVPFSHFNDLRDDHSEKFGSKSGQATATSWENAANGHLQDVGSEVLTKLSASLTTTEHAEKSVMDKQEAVDISSTLSNEEDDRVTHRAPLSLGFDANDDYVEHKRRKMDVYAATSTSTNAIDIGAVASRAIREPRVVVQTTSEVDILDDGYRWRKYGQKVVKGNPNPRSYYKCTHPGCSVRKHVERSSHDLKSVITTYEGKHNHEVPAARNSGHPSSGSAAAPQATNGLLHRRPEPAQGGGGGSLAQFGYGSAGHRPAEQFGAAAAGFSFGMLPRSIATPAPSPAIAVPAMQGYPGLVLPRGEMKVNLLPQSGNAGAAASQQLMGRLPKQHPQM*

>LOC_Os09g09630.1

MSANIESFCCIDHRMIIQEMKREHGLLIDLKNHIIPILRFNNVQADHIVHAFDDILCCSNGIISKIQAEVCDGGNSDPGIDKGNGRNALDNMKVFIEDGTVTKNKRRKNAQHTGSVVTATPDYDGYEWRKYGQKSISKTKHSRSYYRCTNQKGQGCMATKTVQQIENDNSSNSVVKLYNVDYFGKHTCKFGNDMVCPDIVETDSPKYSSINDKYASTRLTNHSDDHQPKNDMKPENLFAVPDMSLFSENMWDIIFEDVTMNSTFSLEQEAKDSWIKHQQESTIHLWADELC*

>LOC_Os09g16510.1

MESMEGNGGGRLVVTELSHIKELVRQLEGHLGGSGSPDLCKHLASQIFSVTERSIGMIRSGHFDGHRKRSAAAVAAGDLDSATPSPLSDVSDLPFKATKKRKTSTEKKRHQIRVSSTGGVENPPVDDGHSWRKYGQKEILGAKHPRGYYRCTHRHSQGCMATKQVQRTDEDATVFDVIYHGEHTCVHKAVAAGAGKPETETDTNAAAESRLHDLSSGLTVKIEGLTAPPQQQQGGGGWNAMPPFCLSSPVSGLAPPDQHNPFSAPSTPENRLAAAASSAASPATSDSMAAAPFHQAAAGGGDEAWRDAELQEVVSALVAATTTTATAQPAPATAMVDADLSALDAFEFDPGFTIDITSFFA*

>LOC_Os09g25060.1

MDAAWRGGVGCSPVCLDLCVGLSPVREPSAARHELLDRPAGCRGGGDSKSMTNDEAKIVEAKVTQMSEENRRLTEVIARLYGGQIPRLGLDGSASPPRPVSPLSGKKRSRESMETANSCDANSNRHQGGDADHAESFAADDGTCRRIKVSRVCRRIDPSDTSLVVKDGYQWRKYGQKVTRDNPSPRAYFRCAFAPSCPVKKKVQRSAEDSSLLVATYEGEHNHPHPSPRAGELPAAAGGAGGSLPCSISINSSGPTITLDLTKNGGAVQVVEAAHPPPPPDLKEVCREVASPEFRTALVEQMASALTSDPKFTGALAAAILQKLPEF*

>LOC_Os09g25070.1

MDDDGDGSSSPTDDSAAAGLLPLFSRSPAEDLEEKLRRAMEENARLTRALDAILAGHHAHQRALLAPSLSPPPPSATARAPSVSTSCAAREDAAPAVAAAAASTACPSRQQPPTAEPRPKVRTVRVRADAADATDANSMAETVKDGYQWRKYGQKVTRDNPYPRAYFRCAFAPSCPVKKKLQRCAEDRSMLVATYEGEHNHALSTQTTEFVASGCTTSQHAGGSSSSPLPCSISINSSGRTITLDLTNQAGSGSIASCGVEAAAVSGELVTVLSPELRRHLVEEVVQVLKNDAEFVEAVTNAVAARVVDQIPHIPVHL*

>LOC_Os09g25070.2

MEENARLTRALDAILAGHHAHQRALLAPSLSPPPPSATARAPSVSTSCAAREDAAPAVAAAAASTACPSRQQPPTAEPRPKVRTVRVRADAADATDANSMAETVKDGYQWRKYGQKVTRDNPYPRAYFRCAFAPSCPVKKKLQRCAEDRSMLVATYEGEHNHALSTQTTEFVASGCTTSQHAGGSSSSPLPCSISINSSGRTITLDLTNQAGSGSIASCGVEAAAVSGELVTVLSPELRRHLVEEVVQVLKNDAEFVEAVTNAVAARVVDQIPHIPVHL*

>LOC_Os09g30400.1

MASSSDHGSLMEDWMPPPTPSPRTLMSSFLNEDFVSGSFSNIFSDHESNKPQDQFERNRELVDLSKEVPSQFARPAFQRDASLDHSLVSPTQRSNSHGGLAERRAARAGFSVPKIDTSRGGSSTVIRSPVAIPPGLSPTTLLESPVFLYNAMAQPSPTTGTLPFLMASNAKSTIPSATKMDEDCTFGNDTFSFQPHVGSRRPNFSAAEKGPNACHQNQSLSNIHQRESSLQSSFTAVKDITDEKNIKTKTSDSMFGDNHSSDEQDDETNQNGENSMPPPNHRSGVPLSHTNDPEVNVLENRGSQTCHNSASLWDNAKNDCLQDVQSEVIETRTAACLPVSTNCDTSIMESQDAVDVSSTLSNEEDDRATHGTASIECNGDGDETDSKRRKLDALTAATAAITTTSNIDMGAAASRGVREPRVVVQTTSEVDILDDGYRWRKYGQKVVKGNPNPRSYYKCTHQGCSVRKHVERASHDLKSVITTYEGKHNHEVPAARNSGHGSSGSGNAPSAPQSNGSQRRQEQGQASFSQFGGAAPFSSFVLPPRNQFGPSASNFPFGMVPPGMAIPMPSLGSLAPAKMAGHPSTMQGYQGLMIPEGEMKTEPMSQLGFPAVNQSSSSFQQMMNRPPSFGPQM*

>LOC_Os09g30400.2

MASSSDHGSLMEDWMPPPTPSPRTLMSSFLNEDFVSGSFSNIFSDHESNKPQDQFERNRELVDLSKEVPSQFARPAFQRDASLDHSLVSPTQRSNSHGGLAERRAARAGFSVPKIDTSRGGSSTVIRSPVAIPPGLSPTTLLESPVFLYNAMAQPSPTTGTLPFLMASNAKSTIPSATKMDEDCTFGNDTFSFQPHVGSRRPNFSAAEKGPNACHQNQSLSNIHQRESSLQSSFTAVKDITDEKNIKTKTSDSMFGDNHSSDEQDDETNQNGENSMPPPNHRSGVPLSHTNDPEVNVLENRGSQTCHNSASLWDNAKNDCLQDVQSEVIETRTAACLPVSTNCDTSIMESQDAVDVSSTLSNEEDDRATHGTASIECNGDGDETDSKRRKLDALTAATAAITTTSNIDMGAAASRGVREPRVVVQTTSEVDILDDGYRWRKYGQKVVKGNPNPRSYYKCTHQGCSVRKHVERASHDLKSVITTYEGKHNHEVPAARNSGHGSSGSGNAPSAPQSNGSQRRQEQGQASFSQFGGAAPFSSFVLPPRNQFGPSASNFPFGMVPPGMAIPMPSLGSLAPAKMAGHPSTMQGYQGLMIPEGEMKTEPMSQLGFPAVNQSSSSFQQMMNRPPSFGPQM*

>LOC_Os09g30400.3

MASSSDHGSLMEDWMPPPTPSPRTLMSSFLNEDFVSGSFSNIFSDHESNKPQDQFERNRELVDLSKEVPSQFARPAFQRDASLDHSLVSPTQRSNSHGGLAERRAARAGFSVPKIDTSRGGSSTVIRSPVAIPPGLSPTTLLESPVFLYNAMAQPSPTTGTLPFLMASNAKSTIPSATKMDEDCTFGNDTFSFQPHVGSRRPNFSAAEKGPNACHQNQSLSNIHQRESSLQSSFTAVKDITDEKNIKTKTSDSMFGDNHSSDEQDDETNQNGENSMPPPNHRSGVPLSHTNDPEVNVLENRGSQTCHNSASLWDNAKNDCLQDVQSEVIETRTAACLPVSTNCDTSIMESQDAVDVSSTLSNEEDDRATHGTASIECNGDGDETDSKRRKLDALTAATAAITTTSNIDMGAAASRGVREPRVVVQTTSEVDILDDGYRWRKYGQKVVKGNPNPRSYYKCTHQGCSVRKHVERASHDLKSVITTYEGKHNHEVPAARNSGHGSSGSGNAPSAPQSNGSQRRQEQGQASFSQFGGAAPFSSFVLPPRNQFGPSASNFPFGMVPPGMAIPMPSLGSLAPAKMAGHPSTMQGYQGLMIPEGEMKTEPMSQLGFPAVNQSSSSFQQMMNRPPSFGPQM*

>LOC_Os10g18099.1

MASRHQLQTMQFTDPASRSPRPVGGGVHGQPPPTPMSSPFSSRKPRMQEGHPTCVNLTPIPHTDGHLWRKYGEKKIKNSSFPRLYYRCSYRDDRNCMATKVVQQENDADPPLYRVTYIHPHTCNPSPPAPTPAHVFTEPPPAKAEVHHAVLFRFSSTAGGHTANNAVHRQQWQPAAATMAAGAQAQLSMTMSDDEREQPPAAIRSAPPARRLSMFRAVVDGLRQMRSSAPPTPSSSMVVDDGWDTFSSFDLDTCEFSVDDELLCGDHMYFPDSMQQ*

>LOC_Os10g42850.1

MCDSLFWQSSADQGDLSDVVRASLQLQTAPRHQAASPPYVHLLGGGGGGGEDQLAAVSQHAEQQQQSMVDASAACDLLHALLPPPPVVQVQQQGASRTRTTIEEDTTGDGEELFAGAHYVVPPIKRRKSQTKKVVCIPAGASGGGGGEVVPSDLWAWRKYGQKPIKGSPYPRGYYRCSSSKGCSARKQVERSRADPTMLVVTYTSDHNHPWPTHRNALAGSTRPSSSNSSNIRLQDSTPVHHQSQTGHDRLTTTHLKQEDVIISPSLLQPDHHQLCTIIDTKHHLLFHQDYPHSFGLFD*

>LOC_Os11g02470.1

MAVTESACLSYEQEAVAVREVAQVYELIKTQQPLLLVHQQPQQLAHGLLNHALRALNVALSVMNQPHASSSAAAAAVPVMSLIKAEAATPANSSSPAADVAADNHVVGKPRRSSSAAKRRRINGEEYKSSSWSQFTPVPHEDGFQWRKYGEKKIQGTHFTRSYFRCTYRDDRGCQATKQIQQKDKNDPPMFQVTYSNEHTCTTTRLINNINNPAALHNLTANPNGHHDSDDDDTIFTKMIKQEEQAAWLPPPPPADLATISNNFDETPGLHVCQEVPPSSSNSSVISHYADEFDHHQMLETTVMEEALGLGADLDDPYFYDPNLLLIYESLMNCY*

>LOC_Os11g02480.1

MALDSVPSYPSDLGSSRARTPQQQRVSPRKEERTWTTDTYAPYDDGHQWRKYGEKKLSNSNFPRFYYRCTYKNDMKCPATKQVQQKDTNDPPLFSVTYFNHHTCNSSSKIVGSTPDSTVQSRKAISICFNSHGQTGEQPTFLSSSASLLSPSMQSYSSNQQPDMNTYSRQFQWADTSSSTSNAPVKMEADDYAEASASPSTTGALSRTLLPIGQSRCIEYFHFL*

>LOC_Os11g02480.2

MALDSVPSYPSDLGSSRARTPQQQRVSPRKEERTWTTDTYAPYDDGHQWRKYGEKKLSNSNFPRFYYRCTYKNDMKCPATKQVQQKDTNDPPLFSVTYFNHHTCNSSSKIVGSTPDSTVQSRKAISICFNSHGQTGEQPTFLSSSASLLSPSMQSYSSNQQPDMNTYSRQFQWADTSSSTSNAPVKMEADDYAEASASPSTTGALSRTLLPIGQSRCIEYFHFL*

>LOC_Os11g02520.1

MKILESFGHSDCQVVINMIEHQKALMVELRGMVMPLLPSDNEQAKLALQLLGDILSCSDKAISMLELGGDTKKLTNLVGGKRKGDKHSMDNHNLEEEAKESVSKRRKNAEHTGSTVAQAPHNDGHQWRKYGQKWISRAKHSRSYYRCANSKVQGCPATKTVQQMDSSGNGTSKLFNVDYYGQHTCRGDGIADPYVVDTAHHSMEPINQNECNSPTLEHEAHEVQDERFENLCMVQNMPEYLIDFELERAFEFIVNSPLGSEHWTFDDSIRCEHSPICIWG*

>LOC_Os11g02530.1

MKNSSNKRSLVADQWHPSSVCCDHRAALREIAKGQSLVTQLRAIVLPALHSDERCDLAAQMLEGILDCSRKAVSQLQLLLSSPHDDDDHHHVDDKRRVRKIISSSDDDDHCSSKAAEDDNAKPLRQHKRRRFGDSVSLETPVPHYDGHQWRKYGQKHINNSKHPRSYYRCTYRQEEKCKATKTVQQREDLHHANSYNGDHPIMYTVVYYGQHTCCKGPAALADDHVVVEASQISTDSHCQSPSSSSDLQAAEVHAGNSSQCSNISVTCSPSVVVEDCNKLLDMMPAADELTADVLLFDMTAYAPLDLDINWEMDTNALWV*

>LOC_Os11g02540.1

MEEAYCMMMVGRERELVAELRHLLFPSPSPTPTTPASHSTTALAGDGECCLPPGLTTTTTVSGGGRRRGRKRVNRDNDNVKLLLQADDDQEAVIADHGDANAKPLPNFTKTRRRKQQATTSTMVTTVPDFDGYQWRKYGQKQIEGALYPRSYYRCTNSTNQGCLAKKTVQRNGGGGAAGYTVAYISEHTCKSIEPSLPPVILDTTVRTTNNHQQPAAAESPAATSSSSSNMVMTSSETGNWSGQHGAYACRQMIAADEEYCCWDTPATTTTTSGSNGGNSTCAEDIELLSRPIRSPMHIAAEGNWMDDLLLVTDGLIVISNSSITHFLT*

>LOC_Os11g29870.1

MENFPILFATQPTSSSTSSSYHFMSSSSGSHDHRHHHGLQAGGNGGGGGGSLSHGLFMGSSSSSIRMEELSNSKQAGDVVVDGGATRSPHGGDGDGAAGDDGGDAQAAAAGGRKKGEKKERRPRFAFQTRSQVDILDDGYRWRKYGQKAVKNNKFPRSYYRCTHQGCNVKKQVQRLSRDETVVVTTYEGTHTHPIEKSNDNFEHILTQMHIYSGLTPSSAAHASSSSPLFPSAAAAASHMFQ*

>LOC_Os11g45850.1

MGVLKVGAYINLCILHNRKEPQMERRKVIRMSYTEDDGFSWRKYGQKDVEGAMHPTTQSNYFRCAHKMTTGCKARKKVQRTDGDPLMVDVVYKGVHSCAGVHSDSQRSSAASSKSNLRPTKSMQVRASSKDVGPPDDGYSWKRYGQKNIFGANYPRCYYRCIHKTTTGCTATKNAQATDGDPLLFDVVYHGEHTCDLQSTHSNDVEPIRPQSGLDDDMCTDDTTTVSTRHDSNTDASSISFQLDWTNCKDESDGPPTTL*

>LOC_Os11g45920.1

MWHVRSNTEDDGLSWSKYEQKEILGAKFPRAYFRCTHWNTKKGCMATKEVQRDDGDPLMFDIVYHGEHTCTQTAESNVDEQIRLTRTRDKKVKRTKRKRQVRVTSVPADDGYSWRKYGQKNVLGFSYLRGYYRCATKGCQASKQVQRHDDGLLFDVTYFGEHTCADQPQAAHSSDQSTILERPTFKTRLQGQVDAMEKQHEGFRKMLSSIQEAIGAMSVKQGEVQETMNKMEKSISSWRPQVDAAVQSLQRDMELQRNQVGAVERHQAEADKSSNTSQALEEREEIARRTSQLPTSPTMSLPVSQALEEREEIARRAPLLPTPPTASMPASTGEIGLDGHRISTQFRGRASGVVTTLVPPPGKGFVTVTSVKTTDSMDGEDVASRPGPGAGRWHWAPGS*

>LOC_Os12g01180.1

MAHGGGEEEEERVLSHGDVVLLRCDLTILRGPHFLNDRIIAFYLAHLAADHHDDDLLLLPPSVPYLLSNLPDPASVAAVADPLRLASRRLVLLPVNDNPDVSHAEGGSHWTLLVLDNSNAVSGPRFVHHDSLPPTNLPSARRLAAVLRPLLPASAIPLIEGPTPRQTNGYDCGVFVLAVARAICNWWPTRARHSNSDSDWLEAVKREVNADSVKAMRTQLLQLIHTLIQNNTTTNQHSPSTQLPSHPSIASTSLPVTAQDFGTWLEDSGTHTAYDQKKADTGKGACWDNLTVSQSVRKPNVSAKNSLSYDGYSWRKYGQKQVKGSEFPRSYYKCTHPTCPVKRKVEMTPDGRIAEIVYNGEHNHPKPHPPRKPTLSTSVETLVATNDAGLENKLEGCDQAIGSDAVVEALRGGCHCLDGFRNGNEISDCKKRYAYAVIFIQNLLMFFCAKNVQVVA*

>LOC_Os12g02400.1

MAVTESVCLSDEQQAVAVREVAQVYELIKTQQPLLLVHQQPQQLAHGLLNHAMRALNVALSVMNQPHASSSSSAAAAAGGHHFPVMTMIKAESTPANSPAADVSDNHVAGKARRSSPAKRRRINCEDKSSWVYHTVVPHEDGYQWRKYGEKKIQGTHFTRSYFRCTYRDDRGCQATKQIQQEDKNDPPMFQVTYSNEHTCTTTRLINNTNNNPAALHSLTANPNGHPDDDSDDTILTKMIKQEQQAAWLPSPPPDLTTISNNFDETPGLHVSQEVPPCSSNSSAISHYADEFDHHQMGQQLETTVMEEALGLGADLDDPYFYDPNLLLIYENLMNCY*

>LOC_Os12g02420.1

MALDSVPSYPSDLGSSRARTPQQQRVSPRKEERTWTTDTYAPYDDGHQWRKYGEKKLSNSNFPRFYYRCTYKNDMKCPATKQVQQKDTNDPPLFSVTYFNHHTCNSSPKIVGSTPDSTVQSRKAISICFNSHGKTGELPTFLSPSASLLSPSMQPYSSNQQPDMNTYSRQFHWADTSSSTSYAPVKMEADDYAEASASPSTTGALSRTLLPIGQSRCIEYFHFL*

>LOC_Os12g02420.2

MALDSVPSYPSDLGSSRARTPQQQRVRKEERTWTTDTYAPYDDGHQWRKYGEKKLSNSNFPRFYYRCTYKNDMKCPATKQVQQKDTNDPPLFSVTYFNHHTCNSSPKIVGSTPDSTVQSRKAISICFNSHGKTGELPTFLSPSASLLSPSMQPYSSNQQPDMNTYSRQFHWADTSSSTSYAPVKMEADDYAEASASPSTTGALSRTLLPIGQSRCIEYFHFL*

>LOC_Os12g02440.1

MDNHNLEEEAKESVSKRRKNAEHTGSTVAQAPHNDGHQWRKYGQKWISRAKHSRSYYRCANSKVQGCPATKTVQQMDSSGNGTSKLFNVDYYGQHTCRGDGIANPYVVDTAHHSMEPINQNECNSPTLEHEAHEVQDERFENLCMVQNMPEYLIDFELERAFEFIVNSPLGSEHWTFDDSIRCEHSPICIWG*

>LOC_Os12g02440.2

MARRRVSAGAASTPIHQIGIQGRTTKALHGCSVVIKSSTSVVGPYAWVRSGGGRNKLSVLTIGKRKGDKHIMDNHNLEEEAKESVSKRRKNAEHTGSTVAQAPHNDGHQWRKYGQKWISRAKHSRSYYRCANSKVQGCPATKTVQQMDSSGNGTSKLFNVDYYGQHTCRGDGIANPYVVDTAHHSMEPINQNECNSPTLEHEAHEVQDERFENLCMVQNMPEYLIDFELERAFEFIVNSPLGSEHWTFDDSIRCEHSPICIWG*

>LOC_Os12g02440.3

MLELGGDTNKLTNLVGGKRKGDKHIMDNHNLEEEAKESVSKRRKNAEHTGSTVAQAPHNDGHQWRKYGQKWISRAKHSRSYYRCANSKVQGCPATKTVQQMDSSGNGTSKLFNVDYYGQHTCRGDGIANPYVVDTAHHSMEPINQNECNSPTLEHEAHEVQDERFENLCMVQNMPEYLIDFELERAFEFIVNSPLGSEHWTFDDSIRCEHSPICIWG*

>LOC_Os12g02440.4

MARRRVSAGAASTPIHQIGIQGRTTKQALHGCSVVIKSSTSVVGPYAWVRSGGGRNKLSVLTIGKRKGDKHIMDNHNLEEEAKESVSKRRKNAEHTGSTVAQAPHNDGHQWRKYGQKWISRAKHSRSYYRCANSKVQGCPATKTVQQMDSSGNGTSKLFNVDYYGQHTCRGDGIANPYVVDTAHHSMEPINQNECNSPTLEHEAHEVQDERFENLCMVQNMPEYLIDFELERAFEFIVNSPLGSEHWTFDDSIRCEHSPICIWG*

>LOC_Os12g02450.1

MKNSSNKRPLVADQWHPSSVCCDHRAALREIARGQSLVTQLRAIVLPALHSDERGDLAAQMLEGILDCSRKAISELQLQLSSDSPHDDDGHLDDKRRVRKIVSSSSDDDDHSSSKAAEDHNAKPLRQHKRRRFGDSVSLETPVPHYDGHQWRKYGQKHINNSKHPRSYYRCTYRQEEKCKATKTVQQREDLHHANSYNGDHPVMYTVVYYGQHTCCKGPAASADDHVVVEASQISTDSHCQSPGSSSSELQAAAHAGDSSQCSNISVTCSSSVVVEDCNKLLDMLPAADELTTDVLLFDMTAYAPLDLDINWEMDTNALWA*

>LOC_Os12g02470.1

MEEAYCMMMVGRERELVAELRHLLFPSPSPTTPASHSTTALTGDDECLPPGLTTTTTVSGGGGRRRGRKRVRRDNDNLKLLQADDDQEVLAAAVHGDANAKPLPNFTKTSRRKLQTTTSTMVTTVPDFDGYQWRKYGQKQIEGAMYPRSYYRCTNSTNQGCLAKKTVQRNGGGGAAGYTVAYISEHTCKSIEPSLPPVILDTTVRATNNHHPPAASSSCAAQSPAAAATSSSSDMMMTSTSSTSSETGNWSGQHGAYACRRQMIAADEEYCCWDTPATTTTSGSDGGNSSTCADQVIDLMSGPIRSPLHIAADGNWVDDLLLVGDGLIDISSANITHLFSF*

>LOC_Os12g32250.1

MSARPPPPPRPRLALPPRSAAESLFTGAGDASPGPLTLASALFPSDPDGGGGGGGVMTSSSSSAAGATSFTQLLIGNLSAPPPPPPPPQQQQQREAARGGGVARAGPALSVAPPPTAGSVFTVPPGLSPSGLLDSPGLLFSPAMFDAFDVLDMFIPDKILPKRATRIKLDIYFVKTSPEGGFGMSHREALAQVTAQASHSPLRMFDHTEQPSFSAAPTSSEAMQHMNAAVNMTGISDMVMGPTNNENVAFQPAEASQRYQVNAPVDKPADDGYNWRKYGQKVVKGSDCPRSYYKCTHPNCPVKKKVEHAEDGQISEIIYKGKHNHQRPPNKRAKDGSSSAADQNEQSNDTVSGLSGIKRDQEAIYGMSEQLSGLSEGDDMDDGESRPHEADDKESDSKKRNIQISSQRTSAEAKIIVQTTSEVDLLDDGYRWRKYGQKVVKGNPHPRSYYKCTYAGCNVRKHIERASSDPKAVITTYEGKHNHEPPVGRGNNQNAGNAAPSSSAQQNMQNLSSNQASLTMADFNNINQRPIGVLQFKSEE*

>LOC_Os12g40570.1

MEEEVEAANRAAVESCHRVLALLSQQQDPALLRSIASETGEACAKFRKVVSLLGNGGGGGGGGGGGGHARGRMAGRSRPSAVLREKGFLESSSGGGQLGMMMSGAATPSTSSAAHLRNRIGGGSGVPPDSLRGLDLVSSSSKGGAHQFDPPKLVQPLSVQFQFGATAHRYPFQQHQHQQKLQAEMFKRSNSGISLKFDSPSATGTMSSAFMSSLSMDGSVASLEGKPPFHLISGPVASDPVNAHHVPKRRCTGRGEDGSGKCATTGRCHCSKRRKLRIKRSIKVPAISNKIADIPPDEYSWRKYGQKPIKGSPHPRGYYKCSSVRGCPARKHVERCVDDPAMLIVTYEENAEAPSFCSGTVGPVM*

>LOC_Os12g40570.2

MEEEVEAANRAAVESCHRVLALLSQQQDPALLRSIASETGEACAKFRKVVSLLGNGGGGGGGGGGGGHARGRMAGRSRPSAVLREKGFLESSSGGGQLGMMMSGAATPSTSSAAHLRNRIGGGSGVPPDSLRGLDLVSSSSKGGAHQFDPPKLVQPLSVQFQFGATAHRYPFQQHQHQQKLQAEMFKRSNSGISLKFDSPSATGTMSSAFMSSLSMDGSVASLEGKPPFHLISGPVASDPVNAHHVPKRRCTGRGEDGSGKCATTGRCHCSKRRKLRIKRSIKVPAISNKIADIPPDEYSWRKYGQKPIKGSPHPRGYYKCSSVRGCPARKHVERCVDDPAMLIVTYEGEHNHTRLPTQSAQT*

>Traes_1AL_0404BC790.1

MVSGAAPPPPPESGAGSSSGVGREETKGKGSARGRGSRKASRPRFAFQTKSENDVLDDGYRWRKYGQKAVKNSAFPRSYYRCTHHTCNVKKQVQRLAKDTSIVVTTYEGVHNHPCEKLMEALNPILRQLQFLSQL

>Traes_1AL_180B9CF01.1

AGAESHGCKRGSPVPEEGDEDGSADHHNHRSDEKEQKKKRKGEKKARGSRVAFATKSEVDHLDDGYRWRKYGQKAVKNSSFPRSYYRCTAAQCGVKKLVERSQQDPSTVVTTYEGRHGHPSPLAAHRGSRMIMATGADTAYSLAALQHQQHGFFPAGADVYGRMCIQPTTAVAPSLAHRLSEYGGMEVHADRLPDAVVHYQSRHH

>Traes_1AL_309623B48.1

MAMRPKSEMSPPPAPPSPSDQRDAVIEELRKGSQLAEFLRQQVELIPEDSRRDAALANVTDITTALASSLSVLQSEREQYYCSSSSDAGHASGASGGGGVRNGAVARTRNRKAKHRRGTYGEELPIKEILTEAPENDRFHWRKYGEKKILHADFPRLYYRCGYSDEHKCPAKKYVQQQNSSDPPMFLVTLINDHTCDTLFPDEDQDQPPSSSSSANNSQVLDFSKASLSSAVGISRLKKEEDADMSVTVPSYNYTYDELSSSSLPFLSPKQWEMEMEVKSLFRRHSGDGN

>Traes_1AL_4E924201A.1

MRDLFWLSPGEQGDLSDVVRASLHPPHQLPTPAADEEEEDEYSSLLLEGGGGGGGLVVGHGDEQLGMVAMMMGGNNSSRPPSSDHHVISLHSPPATTYTRPHPEPLAGMLRRPGFEREGDMVVGPPPEIGDRLQHMSIAHHPRVPTAMKPRKSQSKKVVCIPAPTAAPGASGRHSTSGEVVPSDLWAWRKYGQKPIKGSPYPRGYYRCSSSKGCPARKQVERSRTDPNMLVITYTSDHNHPWPTQRNALAGSTRPSSSSAAAAKIAASSSSSLAAAAARNSSNTNVDVDCAGAHHQLKQESDLDLFADMDALSVFSSIDKIQEDDSKQQLFDPFSSGFCDYI

>Traes_1AL_9ADA7A031.1

MAAVGAAPLLYQQQAQAAGDGCYFSSMSSHFSHGGISSTSSSPASSFSAALGATPPAAPAIAADPAAQFDISEYLYDEGTFAAPLPPVVSVPAVGAAAASSASAVTARSAESAERPRTERIAFRTRSEVEILDDGYKWRKYGKKAVKNSPNPRNYYRCSTEGCSVKKRVERDRDDPAYVVTTYEGTHSHVSPSTVYYASQDAASGRFFVAGTHPPPGSLN

>Traes_1AL_B24F28600.1

MAMRPKSDMSPPPSDQRGAVIEELRKGAQLADRLRQQLELIPELGRRSAALASVSNISTALVSSASMLESNREQYGCSSPDPGAAAYAAGASGNGGGIGARNGAIARTRKAKHRRGPHGEELPIKGRILTETPENDGFHWRKYGEKKILNAVFPRSYYRCGYSDEHRCPAKKLVQQQNNIDPPVFMVTLINDHTCSSLFPANDQPPSSSNSATVNSQVLDFTKASLSSAVGLSRLKKEEDAGMSVTVPSYTYEELASYSSLPLLSPKEWEMEMEIKSLFRHHSG

>Traes_1AL_F64E07A92.1

MKEGKREKKPRGSRVAFATKSAVDHLDDGYRWRKYGQKAVKNSSFPRSYYRCTAAQCGVKKLVERSQQDPSTVVTTYEGRHAHPSPIATHRGSRMLMATGVDTVYSLDVLQHQHHGFFPAGTDVYGRMYALPSTDASVVAHRSSEYGGMQVHAGVLPDAVMSYEHVHR

>Traes_1AS_1432A2F79.1

GAGDDEHRGEKKIKISARVSSGRIGFRTRSEVEILDDGFKWRKYGKKAVKNSPNPRNYYRCSAEGCGVKKRVERDRDDPRYVVTTYDGVHNHATPGAAAQYYCYSPPRSSPPAAYSAAGLLQF

>Traes_1AS_F3EAEC435.1

MSSSTGSLDHAGFTFTPPPFITSFTELLSGSGAGDAERSPRGFNRGGRAGAPKFKSAQPPSLPISSPFSCFSVPAGLSPAELLDSPVLLNYSHILASPTTGAIPAQRCDWQASADLNTFQQDELGLSGFSFHAVKSNATVNAQVNYLPLFKEQQEQQQEEVVQVSNKSSSSSGNNKQVEDGYNWRKYGQKQVKGSENPRSYYKCTYNNCSMKKKVERSLADGRITQIVYKGAHDHPKPLSTRRNSSGCAAVVAEDHTNGSEHSGPTPENSSVTFGDDEADKPETKRRKEHGDNEGSSGGTGGCGKPVREPRLVVQTLSDIDILDDGFRWRKYGQKVVKGNPNPRSYYKCTTVGCPVRKHVERASHDNRAVITTYEGKHSHDVPIGRGRALPASSSSDSSAVIWPAAAVQAPCTLEMLAGHPGYAAKDEPRDDMFVESLLC

>Traes_1BL_1D865A8CC.1

YDPYIFQSESYYRCTHHTCNVKKQVQRLAKDTSIVVTTYEGVHNHPCEKLMEALNPILRQLQFLSQL

>Traes_1BL_46340D685.1

MAMRPMSEMSPPPAPSDQRDAVIEELRKGSQLAEFLRQQVELIPEDSRRDAALANVSDITTALASSLSVLQSEREQYYCSSSSSDAGHASGASGGGGVRNGVVARTRNRKAKHRRGTYGEELPIKEILTKAPENDRFHWRKYGEKKILHADFPRLYYRCGYSDEHKCPAKKYVQQQNSSDPPMFLVTLINDHTCDTLFPDEDQDQPPSSSSSANNSQMLDFSKASLSSAVGVSRLKEEEDADMSVTVPSYNYTYDELSSSSLPFLSPKQWEMEMEVKSLFRRHSGDGN

>Traes_1BL_73811B853.1

QVRVASVTDAGPLNDGLSWRKYGQKDILGAPYPRAYFRCTHRHTQGCQATKQVQRAAADPLLFDVIYHGAHTCAQAAAQQQSPAGFEQEQESPPAXXXXXXXXXXXXXXXXXXXXXXXXXXXXXXXXXXGYAAGGGLGADMGFGGQLDEW

>Traes_1BL_794E99FF5.1

MCDLFWLSPGEQGDLSDVVRASLHPPHQLPTPATDEEEEEYSSLLLEGGSGGGGLVVGHGHGDEQLGMVTMMMGGNTRPPSSDHQVISMLHSPTATTYTRPHPEPLAGMLRRPDIERGGMVVAPLPEIGDRLQHMSIVHHPRVPAAMKPRKSQSKKVVCIPAPTAAPGASGRHSTSGEVVPSDLWAWRKYGQKPIKGSPYPRGYYRCSSSKGCPARKQVERSRTDPNMLVITYTSDHNHPWPTQRNALAGSTRPSSSAAATAKIAASSSSSLAAAAARNSSNTNVDVDCARAHHQLKQESDLDLFADMDALNVFSSIDKIQENDSKHQLFDPFSSGFCDYI

>Traes_1BL_9AFA4B870.1

MLASDGAAGAVVVPDGGPGGTAHSVSVSSTSSEAGVGGGGAVEDEAGKCKKEEGEGDDESKEAAADGEADKTKKGAAKGKGAAKAKGEKRPRQARFAFMTKSEVDHLEDGYRWRKYGQKAVKNSPFPRCRSYYRCTTQKCPVKKRVERSYQDAAVVITTYEGKHTHPIPATLRGANHLLAAHAHAHGGHGLIHPGMFRMPAPPGAFRPGDALGSFLQQQHAAMQHQQQVAAAGMAMRQANAMASSHMQQAPPADRGLAAAMAGGTTGNSTHTVSSSSGTDPLRMEHLMAQDYGLLQDMLMPPSFAHSDGATNSNNNAHNRH

>Traes_1BL_B15990028.1

TLLLVRRDEKEQKKKGKGEKKARGSRVAFATKSEVDHLDDGYRWRKYGQKAVKNSSFPRSYYRCTAPRCGVKKLVERSHQDPSTVVTTYEGRHGHPSPVATHRGQRMLMATGADTAYSLATLQHQQDGFFSPGADVYASSVAHRVSEYGGMQFHADLLPDATMGYQQGYC

>Traes_1BL_B4AFDB663.1

MAMRPKSEMSPPPSDQRDAVIEELRKGAQLADCLRQQLELIPELGRRSAALANVSNISTALVSSASMLESNREQYSCSSSDPGAAAYAAGASGSGGGTGARNGAIARTRKAKHRRGTHGEELPIREVLTETPENDGFHWRKYGEKKILNAVFPRSYYRCGYSDEHRCPAKKLVQQQNNSDPPVFMVTMINDHTCSSLFPADDQPHSSSNSATANSQVLDFTKASPSSAAGVWRLKKEEDAGMSVTVPSYTHDELASYSSLPLLSPKEWEMEMEMKSLFSHHSGGGT

>Traes_1BS_EF67E5A24.1

MSSPTGSLDHAGFTFTPPPFITSFTELLSGSGAGDVEGSPRGFNRGGRAGVPKFKSAQPPSLPISSPASPFSCFSIPAGLSPAELLHSPVLLNYSHILASPTTGAIPARRYDWQASADLNTFQQDEVGRGDSGLFGFSFHAVKPNATVNAQTNYLPLFKEHQQQQQQQVVEVSNKSSSGDNNKQVEDGYNWRKYGQKQVKGSENPRSYYKCTYNNCSMKKKVERSLADGRITQIVYKGAHDHPKPPSTRRNSSGCAAVIAEDHTNGSEHSGPTPENSSVTFGDDEADNGAEPETKRRKEHGDNEGSSGGTGACVKPVREPTLVVQTLSDIDILDDGFRWRKYGQKVVKGNPNPRSYYKCTTVGCPVRKHVERASHDNRAVITTYEGRHSHDVPVGRGAGASRALPTSSSSDSSVVVCPAAAGQAPYTLEMLANPAAGHRGYAAKDEPRDDMFVESLLC

>Traes_1DL_46428511F.1

DGSADHGSCRSNEKEKKKKGKGEKKAHGSRVAFATKSEVDHLDDGYRWRKYGQKAVKNSSFPRSYYRCTATRCGVKKLVERSQQDPSTVVTTYEGRHGHPSPVATHRGPRMLMATGANTAYALAALQHQQHCFFSAGADVYAPLVAHRLSEHGGMQFHADLLPDAVMGYQQGYR

>Traes_1DL_5BAB0B6BC.1

MVSGVTGAAAPESGAGSSSGVGREETKGKGSARARGSRKASRPRFAFQTKSENDVLDDGYRWRKYGQKAVKNSAFPRSYYRCTHHTCNVKKQVQRLAKDTSIVVTTYEGVHNHPCEKLMEALNPILRQLQFLSQL

>Traes_1DL_D1EC7DEA6.1

GEAAGVESHGCKRGSPVPEEGDEDGSADHHNHRSDEKEQKKKGKWEKKARGSRVAFATKSEVDHLDDGYRWRKYGQKAVKNSSFPRSYYRCTVARCGVKKLVERSQQDPSTV

>Traes_1DL_D550418641.2

FRTRTEIEILDDGYKWRKYGKKSVKNSPNPRNYYRCSTEGCSVKKRVERDRDDPAYVVTTYEGTHSHXXXXXXXXXXXXXXXXXXXXXXXXAHSTE

>Traes_1DL_DFE1721E0.1

IFPNGDAAQVRDVAAAGAPVALRPKRRCHRGAPEGLSACGVPPAAGGAHPGGQPXRRDAALANVSDITTALASSLSVLQSEKEQYSSSSSSYDPGHASGPSGGGMRNGPVARSRNRKAKHRRGTYGEELPIKEILTEAPENDRFHWRKYGEKKILHADFPRLYYRCGYSDEHKCPAKKYVQQQNSRDPPMFLVTLINDHTCDTLFPDEDQDQPPSSSSSPNNSQVLDFSKASLSSAVGVSRLKKEEDADMSVTVPSYNYTYDELSSSSLPFLSPKQWEMEMEVKSLFRRHSGDGS

>Traes_1DS_A6733B734.1

MSSSTGSLDHAGFTFTPPPFITSFTELLSGSGAGDAERSPRGFNRGGRAGAPKFKSAQPPSLPISSPFSCFSIPAGLSPAELLDSPVLLNYSHILASPTTGAIPARRYDWQASADLNTFQQDEPCRGDSGLFGFSFHAVKSNATVNAQANCLPLFKEQQQQQQQQVVEVSNKSSSGGGNNKQVEDGYNWRKYGQKQVKGSENPRSYYKCTYNNCSMKKKVERSLADGRITQIVYKGAHDHPKPLSTRRNSSGCAAVVAEDHANGSEHSGPTPENSSVTFGDDEADNGLQLSDGAEPVTKRRKEHADNEGSSGGTGGCGKPVREPRLVVQTLSDIDILDDGFRWRKYGQKVVKGNPNPRSYYKCTTVGCPVRKHVERASHDNRAVITTYEGKHSHDVPVGRGRALPATSSSDSSGVIWPAAVPAPYTLEMLTNPAAGHRGYAAGGAFQRTKDEPRDDMFVESLLC

>Traes_2AL_15A7BB684.1

MAVDLMGCYTPRRADDQLAIQEAATAGLRSLELLVSSLSGAAPSKAPQQHPQQPFGEIADQAVSKFRKVISILDRTGHARFRRGPVQSPTPPPPAPVAPPPPPPRPLAVVEPARPAPLTVVAPVSVAAPVPLPQPQSLTLDFTKPNLTMSGATSVTSTSFFSSVTAGEGSVSKGRSLVSAGKPPLSGHKRKPCAGAHSEANTTGSRCHCSKRRKNRVKTTVRVPAVSAKIADIPPDEYSWRKYGQKPIKGSPYPRGYYKCSTVRGCPARKHVERALDDPAMLVVTYEGEHRHSPGPMPMQMAPSPVPIPMPMGAPVAVASVSAGNGHV

>Traes_2AL_1B43EA59E.1

MEGAGASSSSTDDGATREAADADGRPAAATEAASKPAPAPAPGKTMKKQKRARHPRFAFMTKTEIDHLEDGYRWRKYGQKAVKNSPFPRSYYRCTNNKCTVKKRVERSSDDPSVVITTYEGQHCHHTVTFPRGAGAATLASQMAFSAHHHHHLMYNDLPALHSPTTQNPLFSVPAMSSSLLQPLHCNRQELQVASYTTQASSISSPGSVPAVDKGLLDDMVPPAMRHG

>Traes_2AL_409AB7647.1

MDGHTHLAVEWKDQSPGADCSMLPSFLTDPFPADPLVEDCDGGNDGSEGAGFERHGLSVAVGSPQEEGKPATPHFGQRSSSSSSLSERMQARAGFSVAKLSMPGSEYSGAQSPYLTIPPGLSPASLLESPVFLSNAMGQSSPTTGKLLMLGDTNNNNNTRLEPPSIEDRPGAFSFKPLDLKSSQYTAEGKKGSLPNSQHPSAPSRDVPVKTETNIQTTTRGAIPPGHLNQAQFNNGQDLMKCSYHDCNNKRNRLAADRTTAGGDNNDGPPVTAADSEAAKGDYPAAVATAAPAEDGYSWRKYGQKQVKHSEYPRSYYKCTHPSCQVKKKVERSHEGHVTEIIYKGTHNHPRPAAQGRRPAGGAQVHPFNDAQMDAPADNNNNGYGNAGGSQPNAEARSLWHAGVAVQDWRGDGLEATSSPSVPGELCDSSASMQVHDGAARFESPEGGVDVTSAVSDEVDGDDRVAHGSMSQGQGAADTTEGDELESKRRKLESCAIDMSTASRAVREPRVVIQTTSEVDILDDGYRWRKYGQKVVKGNPNPRSYYKCTHPGCSVRKHVERASHDLKSVITTYEGKHNHEVPAARNGGHGSSAASGGTGASQLSHARRAEPPSVQDGLMRLGGCGAPFGLPPRDPLGPMSNYPYSLGGGHAXXLPMPSGLGAVEGLKLPMLSPSLHSVFRQRQAMETAAGFRVPKGEVKDEAAGAGAGAGGGAAAAAAYPQTMMSRLPLGHRM

>Traes_2AL_434E9F101.1

MMTMDLIGGYGRADEQVAIQEAAAAGLCGMEHLILQLSRTGTSESSPVGSSEAPEQQVDCREITDMTVSKFKKVISILNHRTGHARFRRGPVVAQSQGPAVSEPAPVRASSSRSMTLDFTKASSGYGNDAGFSVSAASSSFMSSVTGDGSVSNGRGGGSSLMLPPPPSASCGKPPLASSAASTGAGAGQKRKCHDHAHSENVAGGKYGASGGRCHCSKRRKSRVRRMTRVPAISSKAAEIPADDFSWRKYGQKPIKGSPYPRGYYKCSTVRGCPARKHVERDPSDPSMLIVTYEGEHRHTPADQEPLAPLPEL

>Traes_2AL_B1270662B.1

MEGGSQLGACLPSLYALDPYASPPLLAPLPNQHKLHQMPLVLQEQPGNHGVMFSSDHGGGLYPLLPGIPFCHSAAACEKPTGFAPLGGTGEAGTSAARAANEFASTTTTTTASCHGPSSWWKGAEKGKMKVRRKMREPRFCFQTRSEVDVLDDGYKWRKYGQKVVKNSLHPRSYYRCTHSNCRVKKRVERLSEDCRMVITTYEGRHTHTPCSDDDAAGDHTGSCAFTSF

>Traes_2AS_0186B9E4F.2

MVAPSSSILKASRSLIDNFDVFHEQDLAQLAAQVAQKKELREKQGAGLHHKIGPQLAFSKYSILDQVDNSSSFSLATSVLTPQHVSSSVGAASMQGQTLPSHTGSGSVNTGPTGVLQVLQDSSTTLDSINTGSAGVLEALQGSSITLDKPADDGYNWRKYGQKAVKGGKYPKSYYKCTLNCPVRKNVEHSADGRIIKIIYRGQHCHEPPSKRFKDCGDLLNELDEFNDAKDPSTRSQLGCQGYYGKPITPNGTMVDGLLPTKEEGDEQLSSLSDIREGDGEIRTVDGDVGDADANERNAPGQKIIVSTTSDVDLLDDGYRWRKYGQKVVRGNPHPRSYYKCTYQGCDVKKHIERSSEEPHAVITTYEGKHTHDVPESRNRSQGTGQHHCKEQTYSEQPAASFCSSSEKRKYGTAILNDLAF

>Traes_2AS_1AFFE8DA6.1

DEYRGRNNDKRSRSLVTVVPHYDGHHWRKYGQKNINGREHARHY

>Traes_2AS_6269D889E.1

MESVEGNGAGRGNLQLVVSELCRVQELRQLELHLHAPDASIDLCRALTAEIFALTDRSIGFVAAAHFPDAPTTPSSTSSSLSGVSDQPFRTNTKKRKATTRWTSQVRVSAAGGAEWPGDDGHSWRKYGQKDILGARHPRAYYRCTHRNSQGCPATKQVQRADQDPALFDIVYHGQHTCRPGGGGGGXXXXXXXXXXPQPARGEPAAEPQSRPDRGRGPRRPEQHLSLTAVDGLACGVRLQRWPDHVTVPGAGRRVHGVAAGRRPPGGGVGAHSRVGPEHGLPFRVRPDLRPWCAQLLHVRTTNRASKWNYMTLPSPCLSLVRG

>Traes_2AS_C407071E4.2

MQFAGCSVVVLPILRSQVFRGLKAWWTLKIVNKNFFQVEPSPTTGSLSMAAIMHKSAHPDMPSPRDKSIRAHEDGGSRDFEFKPHLNSSSQSVAPAMSDLKKHEHSMQNQSMNPSSSSSNMVNENRPPCSRESSLTVNVSAPNQPVGMVGLTDSMPAEVGTSEPQQMNSSDNAMQEPQSENVADKSADDGYNWRKYGQKHVKGSENPRSYYKCTHPNCEVKKLLERAVDGLITEVVYKGRHNHPKPQPNRRLAGGAVPLNQGEERYDGAAAADDKSSNALSNLANAVNSPGMVEPVPVSVSDDDIDAGGGRSYPGDDGTEEEDLESKRRKMESAGIDAALMGKPNREPRVVVQTVSEVDILDDGYRWRKYGQKVVKGNPNPRSYYKCTSTGCPVRKHVERASHDPKSVITTYEGKHNHEVPAARNAIHEMSAPPMKNVVHQINSNMPSSIGGMMRACEVRNFSNQYSQAAETDNVSLDLGVGISPNHSDATNQMQSTGPDQMQYQMQPMASMYSNMRHPSMAMPTVQGNSAGRMYGSREDKGSEGFTFRATPMDHSANLCYSGAGNLVMGP

>Traes_2AS_D0C21ADB5.1

MAGASSHDHLHHHGQAAGNNTGGGGGLGQGLFSGSKQEDPSESKDGGDDRAGSSSQGGGEADVVVGKKKGEKRERRPRFAFQTRSQVDILDDGYRWRKYGQKAVKNNNFPRSVNPCPNFSYDTLTC

>Traes_2BL_2BA3A755A.1

MDGHTHLAMEWKDQSPGADCSMLPSFLTDPFPADPLVEDCDGGNDGSEGAGFERHGLSVAVGSPQEEGKPATPHFGQRSSSSSSLSERMQARAGFSVAKLSMPGSEYSGAQSPYLTIPPGLSPASLLESPVFLSNAMGQSSPTTGKLLMLGDTNNNNNARLEPPSVEDGPRAFSFKPLDLKSSQYTAEGKKASLPNSQHPSAPSRDVPVKTETNIQTTTRGANPLGHLNQAQFNNGQDLMKRSYHDCNNKRNRLPADSATAGGDNNAPPPDHGPPATAVDSEAAKGDYPAAVATAAPAEDGYSWRKYGQKQVKHSEYPRSYYKCTHPSCQVKKKVERSHEGHVTEIIYKGTHNHPRPAAQSRRPAGGAQVHPFNDAQMDAPADNNNNGYGNAGGSQPNAEARSLWHAGVAVQDWRGDGLEATSSPSVPGELCDSSASMQVHDGAARFESPEGGVDVTSAVSDEVDGDDRVAHGSMSQGQGAADTTEGDELESKRRKLESCAIDMSTASRAVREPRVVIQTTSEVDILDDGYRWRKYGQKVVKGNPNPRSYYKCTHPGCSVRKHVERASHDLKSVITTYEGKHNHEVPAARNGGHGSSAASGGTGASQLSHARRAEPPSVQDGLMRLGGCGAPFGLPPRDPLGPMGNYPWPPPSLPXXXXGAVEGLKLPMLSPSLHSVFRQRQAMETAAGFRVPKGEVKDEAAGAGAGGGAAAAAAYPQMMSRLPLGHRM

>Traes_2BL_6B75B32E3.1

MAVDLMGCYTPRRADDQLAIQEAATAGLRSLELLVSSLSGAAPSKAPQQHPQQPFGEIADQAVSKFRKVISILDRTGHARFRRGPVQSAPPPPPPPAPVAPPPPPPLTVVAPVSVAAPLPQPQSLTLDFTKPNLTMSGATSVTSTSFFSSVTAGEGSVSKGRSLVSAGKPPLSGHKRKPCAGAHSEANTTGSRCHCSKRRKNRVKTTVRVPAVSAKIADIPPDEYSWRKYGQKPIKGSPYPRGYYKCSTVRGCPARKHVERALDDPAMLVVTYEGEHRHSPGPMPMQMAPSPMPMPMGAPVAVASVSAGNGHV

>Traes_2BL_A5BFA97B9.1

MEGGSQLGACLPSLYALDPYASPPLLAPSPNQHKLHQLPPVLQEQPGVHGVMFSSDHGGGLYPLLPGIPFCHSAAACEKHTGFAPLGSTGEAGTLAARQGNEIASATTTTTASCHGPSSWWKGAEKGKMKVRRKMREPRFCFQTRSEVDVLDDGYKWRKYGQKVVKNSLHPRSYYRCTHSNCRVKKRVERLSEDCRMVITTYEGRHTHTPCSDDDVGGDHTGSCAFTSF

>Traes_2BL_A69F6C5DF.1

MAGVECGGGDWPFSAEEAYADSSALLAEIGWAAGFVDDGCAGELLPPLDPHPATPAGSMEGAGASSSSTDDGATREAADADGRPAAATEAASKTAPAPAPGKTMKKQKRARQPRFAFMTKTEIDHLEDGYRWRKYGQKAVKNSPFPRSYYRCTNNKCTVKKRVERSSEDPSVVITTYEGQHCHHTVTFPRGAGAATLASQMAFSAHHHHLMYNDLPALHSPTTQNPLFSVPAMSSSLLQPLHCNRQELQVASYTTQASSISSPGSVPAVDKGLLDDMVPPAMRHG

>Traes_2BS_380EC4D1E.1

MAAGQWSGIGDGGGLWAPPALDSLFPDDQPSPAASALGFFGGSLAQLPSPPPLCGTALLGYPQDNFDVFHEQDLAQLAAQVAQKKELREKQGAGLHHKIGPQLAFSKYSILDQVDNSSSFSLATSVLTPQHVSSSVGAASMQGQTLPSHTGSGSVNTGPTGVLQVLQDSSTTLDSINTGSTGVLEALQGSSITLDRPADDGYNWRKYGQKAVKGGKYPRSYYKCTLNCPVRKNVEHSADGRIIKIIYRGQHCHEPPSKRFKDCGDLLNELNDFNDAKEPSTKSQLGCQGYYGKPITPNGMMTDVLLPTKEEGDEQLSSLSDIREGDGEIRTVDGDDGDADANERNAPGQKIIVSTTSDVDLLDDGYRWRKYGQKVVRGNPHPRSYYKCTYQGCDVKKHIERSSEEPHAVITTYEGKHTHDVPESRNRSQATGQHHCKEQTYSEQSAASFCSSSEKRKYGTAILNDLAF

>Traes_2BS_B65714572.1

MESVEGNGTGRGNLQLVVSELCRVQELVRQLELHLHAPDASIDLCRALTAEIFALTDRSIGFVAAHFPDAPTTPSSTSSSLSGVSDQPFRTNTKKRKATARWTSQVRVSAAGGAEGPGDDGHSWRKYGQKDILGAKHPRAYYRCTHRNSQGCPATKQVQRADQDPALFDVVYHGQHTCRPTGSGGGRRPPTNQHNPHAESLLQSLRAGLTVDADHGGPNASVSPLGASPVASGSNGGLTMSPYPVPAGAYTEWPLDGDLQEVVSALTAVSAPSMDCLFEFDPTFGDGVPNFFM

>Traes_2BS_D435A8999.1

MAAIMHKSAHPDILPSPRDKSIRAHEDGGSRDFEFKPHLNSSSQSLAPAMSDLKKHEHSMQNQSMNPSSSSSNMVNENRPPCSRESSLTVNVSAPNQPVGMVGLTDNMPAEVGTSEPQQMNSSDNAMQEPQSENVADKSADDGYNWRKYGQKHVKGSENPRSYYKCTHPNCEVKKLLERAVDGLITEVVYKGRHNHPKPQPNRRLAGGAVPSNQGEERYDGAAAADDKSSNALSNLANPVNSPGMVEPVPVSVSDDDIDAGGGRPYPGDDATEEDLESKRRKMESAGIDAALMGKPNREPRVVVQTVSEVDILDDGYRWRKYGQKVVKGNPNPRSYYKCTSTGCPVRKHVERASHDPKSVITTYEGKHNHEVPAARNATHEMSAPPMKNVVHQINSSMPSSIGGMMRACEARNFSNQYSQAAETDNVSLDLGVGISPNHSDATNQMQSSGPDQMQYQMQSMASMYGNMRHPSSMAVPTVQGNSAGRMYGSREEKGNEGFTFRATPMDHSANLCYSGAGNLVMGP

>Traes_2BS_F3097F116.1

ENYPIFFGTQPSSSTSNPYHFVAGASSHDHLHHHGQAAGNNTGGGGLNQGLFLGSKQEEPSESKDGGDDGAGSSSQGGGGEADVVVGKKKGEKRERRPRFAFQTRSQVDILDDGYRWRKYGQKAVKNNNFPRSYYRCTHQGCNVKKQVQRLSRDEGVVVTTYEGTHTHPIEKSNDNFEHILTQMQVYSGINNVSQTFGNQHMFQ

>Traes_2DL_04535D371.1

MAGVECGGGDWPFSAEEAYADSSALLAEIGWAAGFVDDGCAGELLPPLDPPPATPTGSMEGAGASSSSTDDGATREAADADGRPAAATEAASKPAPALAPGKTMKKQKRARQPRFAFMTKTEIDHLEDGYRWRKYGQKAVKNSPFPRSYYRCTNNKCTVKKRVERCSDDPSVVITTYEGQHCHHTVTFPRGAGAATLASQMAFSAHHHHLMYNDLPALHSPTTQNPLFSVPAMSSSLLQPLHCNRQELQLASYTTQASSISSPGSVPAVDKGLLDDMVPPAMRHG

>Traes_2DL_362A1F535.1

NIFFLGRCRTQDSSVVTKNMKSLEDGQTWRKYGQKEIQNSKHSKAYFRCTHKYDQQCMARRQAQRCDDDP

>Traes_2DL_4F9F8F1F0.1

MEGGSQLGACLPSLYALDPYASPPLLAPLPNQHKLHQLPLVLQEQPGNHGVMFSSDHGGGLYPLLPGIPFCHSAAACEKSTGFAPLGGTGEAGTSAARAGNEFASATTTTTASCHGPSSWWKGAEKGKMKVRRKMREPRFCFQTRSEVDVLDDGYKWRKYGQKVVKNSLHPRSYYRCTHSNCRVKKRVERLSEDCRMVITTYEGRHTHTPCSDDDAGGDHTGSCAFTSF

>Traes_2DL_F600B5FDF.1

CHCSKRRKNRVKTTVRVPAVSAKIADIPPDEYSWRKYGQKPIKGSPYPRGYYKCSTVRGCPARKHVERALDDPAMLVVTYEGEHRHSPGPMPMQMAPSPMPMPMGAPVAVASVSAGNGHV

>Traes_2DS_0F2500A60.1

TSNSYHFVAGASSHDHLHHHGQAAGNNSSGGGGLNQGLFPGAKQEEPSNSKDGGGDDGAGSSSQGVGEADVVVGRKKGEKRERRPRFAFQTRSQVDILDDGYRWRKYGQKAVKNNNFPRSYYRCTHQGCNVKKQVQRLSRDEGVVVTTYEGTHTHPIEKSNDNFEHILTQMQVYSGINNVSQTFGNQHMFQ

>Traes_2DS_97E3E7CFC.1

MAAIMHKSAHPDILPSPRDKSIRAHEDGGSRDFEFKPHLNSSSQSLAPAMSDLKKHEHSMQNQSMNPSSSSSNMVNENRPPCSRESSLTVNVSAPNQPVGMVGLTDNMPAEVGTSEPQQMNSSDNAMQEPQSENVADKSADDGYNWRKYGQKHVKGSENPRSYYKCTHPNCEVKKLLERAVDGLITEVVYKGRHNHPKPQPNRRLAGGAVPSNQGEERYDGAAAADDKSSNALSNLANPVNSPGMVEPVPVSVSDDDIDAGGGRPYPGDDATEEEDLELKRRKMESAGIDAALMGKPNREPRVVVQTVSEVDILDDGYRWRKYGQKVVKGNPNPRSYYKCTSTGCPVRKHVERASHDPKSVITTYEGKHNHEVPAARNATHEMSAPPMKNVVHQINSNMPSSIGGMMRACEARNFTNQYSQAAETDTVSLDLGVGISPNHSDATNQMQSSGPDQMQYQMQSMASMYGNMRHPSSMAVPTVQGNSAGRMYGSREEKGNEGFTFRATPMDHSANLCYSGAGNLVMGP

>Traes_2DS_AD8820C42.1

MARLPASHHQSTTSSPAPRDREISLITCSLPSFSSANRTCSSVSAAMESVEGNGTGRGNLQLVVSELCRVQELVRQLELHLHAPDASIDLCRALTAEIFALTDRSIGFVAAAHFPDAPTTPSSTSSSLSGVSDQPFRTNTKKRKATARWTSQVRVSAAGGAEGPGDDGHSWRKYGQKDILGAKHPXLLPLHPPQLAGLPRHQAGAARRPGPRALRRRLPRPAHLQANGR

>Traes_2DS_F6FBC974C.2

MAAGQWSGIGDGGGLWAPPALYSLFPDEQPALGFYGGSLAQLPSPPPLLGYPQDNFDVFHEQDLAQLAAQVAQKKELRGKQGAGLHHKIGPQLAFSKYSILDQVDNSSSFSLATSVLTPQHVSSSVGAASMQGQTLPSHTGSGSVNTGPTGVLQVLQDSSTTLDSINTGSAGVLEALQGSSITLDRPADDGYNWRKYGQKAVKGGKYPKSYYKCTLNCPVRKNVEHSADGRIIKIIYRGQHCHEPPSKRFKDCGDLLNELDDFNDAKEPSTRSQLGCQGYYGKPITPNGTMVDGLLPTKEEGDEQLSSLSDIREGDCEIRIVDADVGDADANERNVPGQKIIVSTTSDVDLLDDGYRWRKYGQKVVRGNPHPR

>Traes_3AL_140B829CB.2

MSSGGGGGGDQGRHGLYHQHGHGQLTRYDGAGGYELSNDDMESFFFSQPEGVGGGVRADEIAPYSSITSYLQGFLDPTGLARHLDVPAKHELSVDVRSHDQDSQGTGSAAGESAALLTPNSSVSFSSGGGDGEGKSRRSKKGRAQEPDDQEDGKDHEDGESSKTANNKPKKKAEKRPRLPRVSFLTKSEVDHLEDGYRWRKYGQKAVKNSPYPRSYYRCTTPKCGVKKRVERSYQDPSTVITTYEGQHTHHSPASLRGSAAHLFMPPGLHGLPPPHLIPPGVFHPELMSMMRMPYPSPNMHLLSVPPPPHHHPTSHPMAGTLQQYHFTDYALLQDLSTSTMPNNP

>Traes_3AL_1B73D2C12.1

PKILFNLNFIYYHCRNYYRCSTEGCSVKKRVERDKDDANYVVTMYEGVHNHASPGTIYYAAQDPASGRFFVTGTHQLAP

>Traes_3AL_2297D6E18.1

MASPPPKGESFDFEDPRAQEAMGSASASYSAPGGVFGLSPPESSRRDSRKRRKDRPSWVKHTFTPHFDGHLWRKYGQKNIKDSVFPRLYYRCSYREDKQCLASKLVQQENHEDPPLFKVTYTYEHTCNTAPVPTPDVVAELPAPATGDALFLRFDSTGAGHRDAHRMEQERHYQQPAAPGWPSMMLSFDSNSQQHEQCTFPSELPPAASSSSFSTEGLPAPPSTTDGGGDGFSTWDSLRYGLNDHVHFGDNSYLPNSGNDGDDNY

>Traes_3AL_3160E1F30.1

MDGYRWRKYGQKFIKNNPHPRSYYKCTSARCSAKKHVEKSTDDPEMLIVTYEGSHLHGPQTT

>Traes_3AL_4769A72F1.1

RSYYRCTHPTCNVKKQVQRLAKDTAIVVTTYEGVHNHPCEKLMEALGPILKQLQFLS

>Traes_3AL_67ECA2932.1

AASGKIAFRTRSEEEILEDGYKWRKYGKKSVKNSPNPRYPKRTNSFTFL

>Traes_3AL_6E92D4E1F.1

SVEDVGPLDDGFSWRKYGQKDILGAKYPRAYFRCTHRHTQGCYASKQVQRAHGDPLLFDVVYHGNHTCAQGKHSNSQRPQ

>Traes_3AL_AB2BAE660.1

MSSYSSLLSVSPGEQIGGYADGGDHDDMAAAANYLSSFCFDFGEEYYSLAEAATASYPLHAQQQQQPPTQADSHHSGKAASTTSSSQGLDNINTSLTSSDARSKGSKIAFKTRSEVEVLDDGYRWRKYGKKMVKNSPNPRNYYRCSSEGCRVKKR

>Traes_3AL_DED8A29EC.1

SGENDGEAGGSGSGNKEKAKGSAGRSGKKKASKPRFAFQTRSENDILDDGYRWRKYGQKAVKNSSNPRYGSSN

>Traes_3AL_F326C5B8E.1

GRSKYHYSPSSPVVFSPEKVLGKMENKYTMKIKSCGNGLADDGYKWRKYGQKAIKNSPNPRSYYRCTNPRCNAKKQVERAVDEPDTLVVTYEGLHLHY

>Traes_3AS_5CD024A9E.1

MRKARVSVRARSEAPIIADGCQWRKYGQKMAKGNPCPRAYYRCTMATGCPVRKQVQRCAEDRTILITTYEGTHNHPLP

>Traes_3B_8B0D448D8.1

MRGSNMLSSSGSNKRALQQDCSGGSHAQEHTKRKSRIGMRTDYTYAPYHDGFQWRKYGQKVIRGNAFPRCYYRCTYHQDHGCSASKHVEQHNSADPPLFRVVYTNDHTCSGAAASASDYMASSMQIQQIADASLRKADTEAERPPRPQQPRSGGSYAAAIKEEKDAIVSSLLTVIRGSCDVVKSDTAHEGYSSASLATNCYAMSSPSVAGGSREGSSSSSVSPVVLPAPDDMGLGLDFMVESHWFEPLDLGWFVE

>Traes_3B_990298FF5.1

MTTSSSGSVETSANSRPGSFSFGSASFTEMLGGSAAAGGASGYKALTPPSLPLSPSLMSPSSFFSMPAGMNLADFLDSPVLLTSSIFPSPTTGAFGSQFNWRPEAPTPSAAEQGGKEEQRQPYSDFSFQTAPANSEEAARATMTTSLQPPVAVASQGEEAYTGQQQQAWGYGQQQEGMNASAANPASFSAPALQATSSEMAPAGAYRQTHSQRRSSDDGYNWRKYGQKQVKGSENPRSYYKCTFPNCPTKKKVETSLEGQITEIVYKGTHNHAKPLNTRRGSGGGAAAAQVLQIGGDASENSFGGMVTTPENSSASFGDDDNGVSSPRSGNVGRNDNDDDEPDSKRRRDSGDGEGINMAGNRTVREPRVVVQTMSDIDILDDGYRWRKYGQKVVKGNPNPRSYYKCTTVGCPVRKHVERASHDLRAVITTYEGKHNHDVPAARGSAALYRPAPRAADSTASTGHYLNPQPSAMAYQASAAPNVAGTQQYAPRPDGFGGQNPGSFGFNGNFGFSGAGFDNPTASYMSQHQEQQRQNDAMHASSAKEEPREEDMFFQNSQY

>TRAES3BF001300030CFD_t1

MALATPTAVVLELMTMGQQSAAHLGDLLRAASPPVRAEHQALAAEILRCCDRVIAAVSAGASDKKRKMTDPGATTCHPPAAAMPSKRRVRGAEAHREVHADTTADGFVWRKYGQKDINGSNHPRLYYRCAFRGEGCAATRRVQRSQEEPAAFVIAYYGEHTCGAAFSQQRAEPQPPTVVDSGSNAWGVFGAVDRNRGSPLMPSLAAEHDVRRHGEAPRDTSQRWSSPSSSSSYSEVELGASPVEGFLDGNFDWEWETVVNSLRFGDLLH

>TRAES3BF003800010CFD_t1

MDKGHLGGGGGGGGLLALDASPRQLGFLNLLSPAPFHRSMEADDGGGGGGGGRGRRSIEVDFFSDEKKNMKKSRASAGADAEDHKDQASAAGLAIKKEDLTINLLPGNNTRSDRSMVVDDDGASRADQDRNGRNTGELAVIQAELSRMNEENQRLRGMLTQVNNSYHALQMHLVALMQQRTQMPPVQPQQPPTHEDGKNESAIVPRQFLGLGPSGASADVAEEPSNSSTEVGSPRRSSSNGNEDPERGENPDGPSTAGWLPGRGMSQQQQQQLGAAAKGHDQQAQEATMRKARVSVRARSEAPIIADGCQWRKYGQKMAKGNPCPRAYYRCTMATGCPVRKQVQRCAEDRTILITTYEGTHNHPLPPAAMAMASTTSAAASMLLSGSMPSADGAGLMSSNFLARTVLPCSSSMATISASAPFPTVTLDLTHAPPGAPNAMPLNVARPHAPGQFHVPMPGGGMAPAFAMPPHMLYNQSKFSGLQMSSDSVDAGQFAQPRPPMGLPGQLSDTVSAAAAAITADPNFTVALAAAISSIMAGQHAAGNSNANNSNNNTSNNNVTTTSNNTTSNNTNSETQ

>TRAES3BF005100010CFD_t1

MALATPTAVVLQLMTMGQQSAAHLGELLRAASPPVRAEHQALATEILRCCDRVIATVSAGATDKKRKMTDPGATFCHLPAAAMPSKRRVRAAEAHREVQTDTTADGFLWRKYGQKDINGSNHPRLYYRCAFRGEGCAATRRVQRSQEEPAAFVIAYYGEHTCGAGFGDACQQGAAPVPPTVVDSGSNARGAVGDVDWNRGSLLLPSLPAEHGARRRGEAPSDTSRRLLSPSSSSYSSEVELGASPVGEFLDGSFDWEWETVVNSLRFGDLLQ

>TRAES3BF005100020CFD_t1

MQTQSRLIMNPNGGVTGYEPAATDEQHEAVLRELAHGHELTAHLQAEALRALHGQGQTEATAALILQEVSRAFTVCINIMGGSAPAATPTTPPPDAAAVVVTGAASARRPRDDGVPRKVTVTSSPYSDGYQWRKYGQKRIMRTSFPR

>TRAES3BF005100030CFD_t1

MQTQSRLIMNPNGGVTGYEPAATDEQHEAVLRELAHGHELTAHLQAEALRALHGQGQTEATAALILQEVSRAFTVCINIMGGSAPAATPTTPPPDAAGVVVTGAASARRPRDDGVPRKVTVTSSPYSDGYQWRKYGQKRIMRTSFPRVTVTSSPYSDGYQWRKYGQKRIMRTSFPRCYYRCCYHRERSCPATKLVQQQPPQQHSDGDQTMYTVTYVHEHTCHNMAPAEPEAAARSSTPDPLGFSAGMQPRQQQRGGAGLDRGSKEELERQALVSSLACVLQGHHQSYTGSGAGTPDGSPSQGRVGDGPSASGLSLDTSDDLGLDVMDYGVTDALYFAASSSYGPGGDGMIP

>TRAES3BF021100090CFD_t1

MSSGGGGGGDQGRHGLYHQHGHGQLTRYDGAGGYELSNDDMESFFFNQPEGVGGGVRDDEIAPYSSITNYLQGFLLDPAGLTRHLDVPAKHELSVDVRSHDQESQGTGSAAGESAALLTPNSSVSFSSGGGDGEGKSRRSNKGRAQEADDQEDGKDHEDGESSKTANNKPKKKAEKRPRLPRVSFLTKSEVDHLEDGYRWRKYGQKAVKNSPYPRSYYRCTTPKCGVKKQVERSYQDPSTVITTYEGQHTHHSPASLRGSAAHLFMPPGLHGLPPPHLIPPGVFHPELMSMMRMPYPSPNMHLLSVPPPPHHHPASHPMAGTLQQHHFTDYALLQDLSPSTMPNNP

>TRAES3BF021300010CFD_t1

MSSGGGGGGDQGRHGLYHQHGHGQLTRYDGAGGYELSNDDMESFFFNQPEGVGGGVRDDEIAPYSSITNYLQGFLLDPAGLTRHLDVPAKHELSVDVRSHDQESQGTGSAAGESAALLTPNSSVSFSSGGGDGEGKSRRSNKGRAQEADDQEDGKDHEDGESSKTANNKPKKKAEKRPRLPRVSFLTKSEVDHLEDGYRWRKYGQKAVKNSPYPRSYYRCTTPKCGVKKQVERSYQDPSTVITTYEGQHTHHSPASLRGSAAHLFMPPGLHGLPPPHLIPPGVFHPELMSMMRMPYPSPNMHLLSVPPPPHHHPASHPMAGTLQQHHFTDYALLQDLSPSTMPNNP

>TRAES3BF029000080CFD_t1

MSDCSTGTDSPRADSVDEQPPADAESPGGGQSKRDHPSPSSPLPPPKRSRRSVEKRVVSVPISECGERAKTNGEGPPPPDSWAWRKYGQKPIKGSPYPRGYYRCSSSKGCPARKQVERSRADPTVLLVTYSYDHNHPWPAPKSGCQPNKSSPRLVDPKPEPGTPVECQPEHGPEAPEHGPEAPEQDQEEEHEQKPVIGLADPAAITTVTAAAEEEEESFDFGWFDQYPTWHRTALYVPAFDVAPPPEDWERELQGEDALFAGLGELPECAIVFGRRRELALAATAPCS

>TRAES3BF045500040CFD_t1

MEEMGEESSRYPWQDYDLGFGEELMRELLDQTTTAPTPSPAAMAAGAASADNSSSSDKGIGDEEEGAAGRRESMENRLMSTVYSGPTLSDIESALSFTGAGAGDPLDGRSKYHYSPSSPVYVPRIYANNNHIQFSVDASSIWTSVWKFRVFSPEKVLGKMENKYTMKIKSCGNGLADDGYKWRKYGQKAIKNSPNPRSYYRCTNPRCNAKKQVERAVDEPDTLVVTYEGLHLHYTYSHFLQQQTNPPPAAAAAASSSKKPKLHPTAGAITVTDSHHGSTPVPTTSPPSAAVVPAGAGDSSGDSGGNVTADAGFLLEHAVPNCSPYLFDGGLLSDAGEERRMPSDAGGLLEDMVPLMVRRPSCNSAATTASSSTTLGSPPAPMSSPSPSTSSVSWTPASPYIDMAVLSNIF

>TRAES3BF051200110CFD_t1

MEERCALATELAQVLDTVRQLEAHMGVKGGADGGETCRTLVSSMRSSVDRSIHIAMSSCCVVLGAPESPPSAGGSPRSGGSDQAADSPCRGAHAAGQSKKRKTQPKWSTQVRVNSVEDVGPLDDGISWRKYGQKDILGAKYPRAYFRCTHRHTQGCYASKQVQRAHGDPLLFDVVYHGNHTCAQGKHYNSQRPQPVASGEHRQPQPAGGQERISVGLKTQGCAPGVAAPFSFPSKQAGADTGSDFQAGCATTAAPFMSPATSECKASTTESPMGDMEFMLQLADADFLDNSRYL

>TRAES3BF058500060CFD_t1

MSGEFQFHDELASLFTQRPGPGMQQQEQQASWLADYLQTPMDYDLLCRALELPAAEDVVKRELVVDTTPSGGALTPSGGGGTPNATSSMSSSSSEAGGGLCAGEGDSAGRCKKEDGDGEDGKGGDEGDKSKKGSSAAAKGGKAGKGEKRPRQPRFAFMTKSEVDHLEDGYRWRKYGQKAVKNSPYPRSYYRCTTQKCVVKKRVERSFQDPAVVITTYEGKHTHPIPSALRGSTHLLAAQAAHLHHQHHGHLGMLPQMGMGGRAGSPFGRSSGGGIDVLGGLLQPRAHHGMTAPMAGAGAGHQAPTQGLTGSIRSVASATATASSPPSLQMQHFMAPDFGLLQDMLPSFIHGAGGNSDNQPSSPYGKLH

>TRAES3BF066700160CFD_t1

MAASLGLTSHEACCTVHPTGSSLYNFQADDPFVAAADGAAELSFPELVEAARASDYSPLPAFGVAGGETMNMYGRSVVFPMTTSYYCDGAGMFDGDASARGRWGGIGAMTGRPSGRIGFRTRSEVEVMDDGFRWRKYGKKAVKSSPNLRNYYRCSAEGCGVKKRIERDRDDPRYVLTTYDGVHNHVTPGSTSSRAAPAYSAPTAPAWTWSELHAAAHSSESY

>TRAES3BF073300120CFD_t1

MDGEWSDGAVSGGEQKASGDGVSADCNSPGSPSPPAVPSTSGRRRSLQKRVVTVPLADLNVPRPKGVGEGNTPTDSWAWRKYGQKPIKGSPFPRAYYRCSSSKGCPARKQVERSQADPDTVLITYSYEHNHSSTVARAQSRPTPTPKPNKERPLPSPEPAKSDGTHHGTANVAGGLVTASPAPAIEVHDDFRWLYDVVSVTSSTSPSEVEAADDMLLYGPMFFGKPVVEVDTAALLPGEFGGEAVGGEGGEEDDAMFAGLGELPECAIVFRRHARDGLAAMAGGVKVEQPAEGTAMT

>TRAES3BF081400030CFD_t1

MAMGWFLWAWRWKDRPSWVKHTFTPHFDGHLWRKYGQKNIKDSVFPRCSYREDKQCLASKLVQQENHEDPPLFKVTYTYEHTCNTAPVPTPDVVAELPAPATGDALFLRFNSTGAGHRDAHRMEQERHYQQPAAPGWPSMMLSFDSNSQQHEQCTFPSELPPTASSSSFSTEGLPAPPSTTDGGGDGFSTWDSLRYGLNDHVHFGDHSYLPNSGNDGDDNY

>TRAES3BF090100100CFD_t1

MGDVLRAHDVATTAAGDEAGVWPGELDEQLIRELLSDDSLLGSMAPPDDSERHRSCDTGGAPAAAPCNSGGSIAAVHEPLPQASASSMALCSSYSGPTIRDIEKALWSRPYTSSQRYGSLYFRRYGAPGTAPESRHTTKVRSCGGGKTPMDGYRWRKYGQKFIKNNPHPRSYYKCTSARCSAKKHVEKSTDDPEMLVVTYEGSHLHGPQTTTLRRVQPPDAAADLPGAAGDAVAGAGIGCSVRPTYGTSSGDDARREGNEPLHHGAVQRVTPTDSLASSLPHSAAAVDATVLSSSSLDSPWSLEALLPVERI

>TRAES3BF111700140CFD_t1

MAVAGAGAAYRFHPHGAGSMAFPRPPGSGCPYSSGAPLSSPAFGGATGPGVLQQQLDVLDYLSDDGGVPGTVGAPLPVEAAVVPDVGYCDHTSRAAAVAASGKIAFRTRSEEEILDDGYKWRKYGKKSVKNSPNPRNYYRCSTEGCSVKKRVERDKDDANYVVTMYEGVHNHASPGTVYYASQDPASGRFFVTGTHHLAP

>TRAES3BF180700010CFD_t1

MCGLLLRMEHLNDWDLQAVVRSCATFSSSHHPQEEDRAGPPPGPAAAPPADTPVKREPRDVVRPASAAKDASSLYGLEYLDLDHKPFLLSAPSSQSWAAVDDRHEMMISFPAAASTSGVRPRVPPGRKPGIRSSTPRPKRSKKSQLKKVVCEVPVADGGVSSDLWAWRKYGQKPIKGSPYPRGYYKCSSMKGCMARKLVERSPAKPGVLVITYMADHCHPVPTQINALAGTTRHKTTPADGHATTPKSHGDAHEAVRCEDESNEMSSMAVDGTTEEAAGDDGGEFWPTELDLDELLAPVDGDLDHVFDEDGALGRRLSL

>TRAES3BF267200010CFD_t1

MSSYSSLLSVSPGEQIGGYADGGDHDDMAAAANYLSSFCFDFGEEYYSLAEAATASYPLHAQQQQPPTQADSHHSGKAASTTSSSQGLDNINTSLTSSDARSKGSKIAFKTRSEMEVLDDGYRWRKYGKKMVKNSPNPRNYYRCSSEGCRVKKRVERDRDDERFVITTYDGVHDHLAPLPPRGCAGYSLSLAQTRVDEGSSPLPVQGRRCFLDTMKMHAAGSQQGCTPVPQPRKLERDN

>Traes_3DL_2551BF2C1.1

LCEVHFSFNRVTVTSSPYSDGYQWRKYGQKRIMRTSFPRCYYRCCYHRERSC

>Traes_3DL_48F7A19D2.1

MASAPPKGESFDFEDPRAQEAMGSASASCSPAGGVFGVSPPESSRRDSRKRRKDRPSWIKHTFTPHFDGHLWRKYGQKNIKDSAFPRLYYRCSYREDRQCLASKLVQQENHEDPPLFKVTYTYEHTCNTAPVPTPDVVAELPA

>Traes_3DL_678D51EAD.1

HAAGQSKKRKTQPKWSTQVRVNSVEDVGPLDDGFSWRKYGQKDILGAKYPRAYFRCTHRHTQGCYASK

>Traes_3DL_7456F61A3.1

MRTDYTYAPYHDGFQWRKYGQKVIRGNAFPRCYYRCTYHQDHGCSASKHVEQH

>Traes_3DL_DF0D3F3FE.1

EEEILDDGYKWRKYGKKSVKNSPNPRNYYRCSTEGCYVKKRVERDKDDANYVVTMYEGVHNHASPGTVYYAAQDPASGRFFVTGTHHLAP

>Traes_3DS_9A02CF31D.1

MDELHDMERRQRLDVLTCSLEKLCDLRYLHMDYNIKVSYVLSSLFLRRLHMLCPFPKVPNAIGELHNLFDLDLTIEVLEDDIAILAQLQSLNRLKLHIEGKPETEEKVVICRKGFPVLKHFWLFCVRMSQLTFEAGTMPSLEKLEVRINSLYGAAPMGIEHLLGLKEIFVFVGGCGGEGSCSIRAALTLLRKAIDMRSSHPTANIKCVDNLLLLEHLLDDGFSWRKYGQKDILDAKHPRAYFRCTYRKSQGCSATKEVQRTDDDPELFLVLYKGKHTCIMETSQASQEQRVLVPQLGLQEKLF

>Traes_3DS_F6B1E6078.1

AKARRKVREPRFCFKTMSDVDVLDDGYKWRKYGQKVVKNTQHPRSYYRCTQDKCRVKKRVERL

>Traes_4AL_234E1CDF6.1

MHAPIFLVELKCSFIIYVIDELYLYVLVCRGSGEGERGHHGDEEEQQQAAWAEEAAGVQPLVMPEDGYQWKKYGQKFIKNIQKIRSYFRCRDKRCGAKKKVEWQPGDPSLRIVYDGAHQHGSPASNGGGQDGDGAANRYDLSTQYFGGAGAPTPQTR

>Traes_4AL_2EEECCC4B.1

MANFEMSHQQALAQVTAQAVHSQYTVGSQADYSLPFSSATTSALTSQFINSSANVTSMKETATLPLHTVNDNLKSNEVSQGFQTSALTVDKPADDGYNWRKYGQKAVKGGEYPRSYYKCTQASCPVKKKVEHSAYGQITQIIYRGQHNHQRPPKRRSKDGGNLLNEDDFPENRDALTRSEPGSQDHSGKVEVSNDGITGPSVSKRRGGGDQSSGSSDTEEDNDEAGDDNGDAGIVNANKRHVPAPAQRIIVQTTSEIDLLDDGYRWRKYGQKVVKGNPHPRSYYKCTYQGCDVKKHIERCSQDPTAVITTYEGKHSHDVPAARSSVAAAASANASSSISLLHRGQKAASSSQRVLPRAALHTSDSSLQLKEENEIT

>Traes_4AL_3E11167D9.1

MIKGDQRQLGGHEERLNNEIRDDHQASDGNFFKFLQNQSSSKKEAQEDKIASTRAEMGEVRKENERLKAMLSRMVDDHRTLQKQFDVLHQQGRGKNLAVGSAEHTSLVDGVKDPRFISLRLGTSTGTSKHNYMGEEIKGNTNNPDDIAVGARTDGGEIKVRPDVVTLSPGGSSEEEAAETTTTSVASKTEKNPRSTDSEDDVAQQPLAKKARVSVRARCDTPTMNDGCQWRKYGQKISKGNPCPRAYYRCTIATGCPVRKQV

>Traes_4AL_98B1C762B.1

MEEVEEANRMAVESCHRVLGLLAQTQDPAQLRSIALGTDEACAKFRKVVSLLGNGNGNGNEGGGTHHPRAKLVSRRQTPGFLSQKSFLDNNTPVVVLNSAHPSTSSAQVYPSSRNSILDSSQAAHPIGGPPKLVQPLSAHFQFGDSSRYNQFQQQHQHQQQKMRAEMFKRSNSGINLKFDSPSGTGTMSSARSFMSSLSMDGSVASLDAKSSSFHLIGGPAMSDPVNAQQAPRRRCSGRGEDGNGKCAATGRCHCSKRSRKLRVKRTIKVPAISNKIADIPPDEYSWRKYGQKPIKGSPHPRGYYKCSSVRGCPARKHVERCVDDPSMLIVTYEGEHNHTRMPTQSAQA

>Traes_4AL_9E0D1CFA6.1

MALDSVPSYPSDLGSSGRATRTQQRIRKEERTWTADTYAPYDDGHQWRKYGEKKLSNSNFPRFYYRCTYKTDLKCPATKQVQQKDMSDPPLFTVTYFNHHSCNTTSRPIGSAPDTTEQSSSRRAVSICFGSHATGEQPTFLTSPGTLQSPASTTNQQNDRGAYGHQFQWTDTSPSAGDAPVKMETDSLAGTGASSGAASGHALSRTLLPIGQSRCIEYFQFL

>Traes_4AL_C2A825B6D.1

MAQVSFAGAGDDKHRSEKTIKISARVSAGRIGFRTRSEVEILDDGFKWRKYGKKAVKNSPNPRNYYRCSAEGCGIKKRVER

>Traes_4AS_0DA136E0E.1

MAEDGQKEIQNSTHPRSYYRCTHKSDQGCNAKRQAQICETHPIKYDITYYGEHTCKPPSNTPMIIVAASDDRAENLVSFAPTFPHLAPGSAPALTTCSAPRPIHSCRRTSSPPSWDRPGGRPRWWGRCRTTAGVG

>Traes_4AS_70DF607CC.1

MSPVPSPNQSHLLGHGSRKEKRMRKVDTFAPHNDGHQWRKYGEKKINNCNFPRYYYRCTYKDNMNCPATKQIQQKDHSDPPLYQVTYYNEHSCNSAFLALTPTEFQLQTASGKAVSICFDSSGAQEPGANASSPSSSAAPRATPSESKNKPLALRSEALSSWAPGVVEQKTACADLQSCSTECQDAYISEDIDAGRFGSIRFFHFL

>Traes_4BL_A8C6FBEB6.1

MSPVPSPHQSQLLGHESRKEKRMRKVDTFAPHNDGHQWRKYGEKKINNCNFPRYYYRCTYKDNMNCPATKQIQQKDHSDPPLYQVTYYNEHSCNSAFLALTPTEFQLQTASGKAVSICFDSSGAQEPGANASSPSSSAAPRGTPSESKNKPLALRSEALSSWAPGVVEQKTACADLQSCSTECQDAYISEDIDAGRFGSIRFFHFL

>Traes_4BL_EFEC50B26.2

MSHQQALAQVTAQAVHSQYTVGSQADYSLPFSSATSALTSQFIKSSANVTSMKETATLPLHTVNDNLKSNEVSQGFQTLALTVDKPADDGYNWRKYGQKAVQGGEYPRSYYKCTHASCPVKKKVEHSAYGQITQIIYRGQHNHQRPPKRRSKDGGNLLNEDDFPENRDALTRSEPGSQDHSGKVEVSNDGITGLSMSKRRDGGDQSSGSSDREEDNDEAGDDNGDAGIVNANKRHVPVPAQRIIVQTTSEIDLLDDGYRWRKYGQKVVKGNPHPRMVFEKVDSSFHPGILDLSHLHTPCCILVVKGRGVGR

>Traes_4BS_A6D9EB0E5.1

MLLPAPSLFNAINKASQRDRGRPQQKRRLFSTAGPQNPKASPSSLLASLLLLRPTAMALDSVPSYPSDLGSSGRATRTQQRIRKEERTWTADTYAPYDDGHQWRKYGEKKLSNSNFPRFYYRCTYKTDLKCPATKQVQQKDMSDPPLFTVTYFNHHSCNTTSRPIGSAPDTTEQSSSRRAVSICFGSHATGEQPTFLTLPGTLQSPASTTNQQSDRGAYGHQFQWTDTSPSAGDAPVKMETDSLAGTDASSGAASGHALSRTLLPIGQSRCIEYFQFL

>Traes_4BS_CE839571B.2

MEEVEEANRMAVASCHRVLGLLTQTQDPAQLRSIALGTDEACAKFRKVVSLLGNGNEGGGTHPRAKLVSRRQTPGFLSQKSFLDNNTPVVVLNSAHPSTSSAQVYPRNSILDSQPAHPIGGLPKLVQPLSAHFQFGDSSRYNQFQQHQHQQQKMRAEMFKRSNSGINLKFDSPSGTGTMSSARSFMSSLSMDGSVASLDAKSSSFHLIGGPAMSDPVNAQQAPRRRCSGRGEDGNGKCAATGRCHCSKRRKLRVKRTIKVPAISNKIADIPPDEYSWRKYGQKPIKGSPHPRGYYKCSSVRGCPARKHVERCVDDPSMLIVTYEGNANSPALVDRLRCSPKL

>Traes_4DL_3140A8240.1

MSPVPSPNQSHLLGHGSRKEKRMRKVDTFAPHNDGHQWRKYGEKKINNCNFPRYYYRCTYKDNMNCPATKQIQQKDHSDPPLYQVTYYNEHSCNSAFLALTPTEFQLQTASGKAVSICFDSSGAQEPGANASSPSSSAAPRGTPSECKNKPLALRSEALSSWAPGVVEQKTACADLQSCSTECQDAYISEDIDAGRFGSIRFFHFL

>Traes_4DS_3BE557D5C.2

MEEVEEANRMAVASCHRVLGLLAQTQDPAQLRSIALGTDEACAKFRKVVSLLGNGNEGGGTHPRAKLVSRRQTPGFLSQKSFLDNNTPVVVLNSAHPSTSSAQVYPRNSSILDSQPAHPIGGPPKLVQPLSAHFQFGDSSRYNQFQQHQHQQQKMRAEMFKRSNSGINLKFDSPSGTGTMSSARSFMSSLSMDGSVASLDAKSSSFHLIGGPAMSDPVNAQQAPRRRCSGRGEDGNGKCAATGRCHCSKRRKLRLKRTIKVPAISNKIADIPPDEYSWRKYGQKPIKGSPHPRGYYKCSSVRGCPARKHVERCVDDPSMLIVTYEASVKWEDAKMLFSLKEV

>Traes_4DS_CFC487CE5.2

MYTMIHESYISMYRWLICRGNEGGERGHHGDEEEQQQGAWAEAAGGQPLVMPEDGYQWKKYGQKFIKNIQKIRSYFRCRDKRCGAKKKVEWQPGDPNLRVVYDGAHQHGSPSSNGGGQDADGAANRYDLSTQYFGGAGAPTPQTQ

>Traes_4DS_DC3C9DC42.2

MAAHEASAGGGEGARCTPPRPALSLPPRSAVESFFGSGATAASFAETSPGPFTLAAALFPDMPSSAFHGSFTQLLVGAMGSPAAPPSPPSPFAVPPGLSPTALVGPFPPTGNFEMSHQQALAQVTAQAVHSQYTVGSQADYSLPFSSATTSALTSQFINSSANVTSMKETATLPLHTVNDNLKSNEVSQGFQTSALTVDKPADDGYNWRKYGQKAVKGGEYPRSYYKCTQASCPVKKKVEHSAYGQITQIIYRGQHNHQRPPKRRSKDGGNLLNEDDFPENRDTLTRSEPGSQDHSGKVEVSNDGITGSSISKRRDGGDQSSGSSDREEDNDEAGDDNGDAGIVNANKRHVPAPAQRIIVQTTSEIDLLDDGYRWRKYGQKVVKGNPHPRSYYKCTYQGCDVKKHIERCSQDPTAVITTYEGKHSHDVPAARSSVAAAASANASSSISLLHRGQKAASSSQRVLPRAALHTSDSSLQLKEENEIT

>Traes_4DS_FE38A59D0.1

MALDSVPSYPSDLGSSGRATRTQQRIRKEERTWTADTYAPYDDGHQWRKYGEKKLSNSNFPRFYYRCTYKTDLKCPATKQVQQKDMSDPPLFTVTYFNHHSCNTTSRPIGSAPDTTEQSSSRRAVSICFGSHATGEQPTFLTSPGTLQSPASTTNQQNDRGAYGHQFQWTDTSPSAGDAPVKMETDSLAGTGASSGAASGHALSRTLLPIGQSRCIEYFQFL

>Traes_5AL_06A6F9328.2

MKPTTAGKKGQKRARQQRFAFVTKSEVDHLEDGYRWRKYGQKAVKNSPFPRSYYRCTNSKCTVKKRVERSSEDPSVVITTYEGQHCHHQTSFQRGXXXXXXXXXXXXXXXXXXXXXXXXXXXXXXXXXXXXXXXXXXXXPSLQQLNGGDELRRSTSYSPMASATQTPSSLVPPDVSFDMGLLGDIVPPGVRNG

>Traes_5AL_69A969FF4.1

MDEQAKPSCIVGRKRRRNDGKRSRSLVTNVPHYDGHQWRKYGQKNINGRQHARSYYRCTYTERNCSATKTVQQQDQDGGGSIYSADAGEDQGAKYTVVYYGDHTCKAGDNISNNIIDHLPNLVDIDLRRGETERVTAEISEFDMELDVPALLEVFNNSQLNWEI

>Traes_5AL_6F7D1D441.1

MSETSACAPASRKAAHKADDDGKCHCPKKKKPREKRVVRMPAVSDKVADIPSDSYSWRKYGQKPIKGSPHPRGYYRCSSIKDYPARKHVERCRGDAGMLNVTYENDHNHAQPLDLATLTANSEA

>Traes_5AL_6FDB440FB.1

MAICRNYYRCTNSTNQGCPAKRTVQRNDDDGSDDGRPKYTVVYISEHSCKATESAAVPVILETTVRTDT

>Traes_5AL_7164FEAC3.1

SNRNVGTGEAEHVDVDSPLSNGTCRRIKVKKVCTRIDPSDTSLVVKDGYQWRKYGQKVTRDNPSPRAYFRCAFAPSCPVKKKV

>Traes_5AL_A3653B781.1

MQQRRRCAGKEDGSGRCATGSRCHCAKKRKLRIRRSIKVPAISNKVADIPADEFSWRKYGQKPIKGSPHPRGYYKCSSVRGCPARKHVERCVDDPAMLIVTYEGDHNHNHNQAAAAQPA

>Traes_5AL_B4E8A3115.2

MGDSALHLAARAGNVALVQKIFADCDPELVAELTDHQNQDGETALYVSAEMGHVEVVCEILKVCDLHSAFLKAHNSFDAFHIAAKQGHLVVLQELLKAFPALAMTTNSVNATALYTAALHRHIGIVNLLLDTDPRLARIARNNGKTALHIAARLGNVEVVVLLLNKDPATVFRIDRKGQTAVHMASKGHNAEILLELLKPDVSVIHLEDNKGDRPLHVATRKGKTIIVQTLISIEEIDINAINGAGETAFAIAEKLGNEELVNILREAGGVTAEEQVNPPKSIKRFKQTHDVQSQIKQKRRTNMHFHTIRKSSQKLHTEAPVCALADAMFRLPAKLDELLISHVHMLPRGAEDEIPLIKQDLEEIMAILQEHDHPGRAEDRAMTSKCLTKEVRELSYDMEDSVDQYVHAVDTKRRIVPRRKKYKITCRRGKTTARLPEKLKWRIWMANKIREFSVRSQEALQRYSLFNHPGAHGISTSATSTRHDVCFGSWYPTPCGELVGIDGHLNTLEAWLGKDGEQQLKVVSVVGSGGVGKTTLSKELYRRIRGQFECQAFVRTSRKPDIRRLLISLLSQVRPHQTPHTWKLHSLIADIRTHLHDKRYLIVIDDVWATQTWDIINRALPAGNLCSRILITTEVEDVALKCCGYDSRHVLMVKPLGYDDSSKLFFSTAFGLQYECPPELCDAAHNIVRKCAGSPLAMVTVASLLVSQIGKPEKWDYVNEIFGHGLSTYPSSEGMKQVLNLSYNNLPHYLKACVMYLSIYEEDYIIQKDDLVKQWIAEGLILATEEKDKEEISRRYFDELISSRMILPVYTNDNDDVLSCTLHHMVLDFIKHKSLEENFVIAIDHSQTTAPLADKVRRLSLHFGNAEATPPTNMRLSQVRTLAFFGVIECLPSVIEFRLLQVLILHLFGDDESVSFDLTGISELFRLRYLHVTCNATLEVPQTQMRGLQYLETLKIDARVSAVPSDIVHLPSLLHLSLPVGTNLPNGIDHMTSLCTLEYFDINVNSMENVHSLGELTNLQDLRLTCSTVPSSYLKSKIDSMGSILANLSNLRSVTLKSSGILESEPYSMIISCDGLSSVSSPPALLQRFEWLPRICTFSSIPKWISHLNKLCILKIGLRELVSNDVAALRGLPALTVLSLYVRAKPAEKIVFTRAGFLVLKCFKFRCSVPWLEFEVDAMPNLLKLKLSFDAHGVDQHRTIPVGMVHLTGLKEISAKIWGAGANERRAAKSALIDAIKMHSGCPTSSIQCLDGMFSGKDDNNSGIQEEEHLTLQKQYNIKEEDSKKQHDLPKDYMDVAYKQTSSSNNHRKSKRITQVRMQVRVGSVQDNSALEDGFSWRKYGQKDIIGSMHPRAYFRCTHRHVKGCPVTKQVQRTSTDPLLFDVVYHGEHTCLDSVGSPATSCGHVAGVEVMSRSRPGVGFVSQSQAACSSQVMSSEVVSGSGSTAGLWGDEIDMPDPDRDDTGISADYLGGYEFDVSAFFA

>Traes_5AL_E566BD64E.1

QKVVKGNPRPRSYYKCTAENCNVRKQIERASTDPRCVLTTYTGRHNHDPPGRGAGXXXXXXXRLLL

>Traes_5AL_E644A6A0B.1

MQSQEKITPVKPVASRPFSSFTSFSKLLKDFTATGSAKITSPGETVIVRRPKVTRFAPPPSDLSAGVAASMLQDAGLDTTREKMVIDPEQVVSCDQMTTFHDINKPIHSVKTRLSYDGYNWRKYGQKQVKGSEFPRSYYKCTHPTCPVKRKVETTVDGQIAEIVYNGEHNHPQPHPPKKPASSASTEVVVPDAHGSNDAGAESQLGGCNLALVSDPVAAAFKSSCYYVDEFGNTSPVYHWNTSRKEKQSSIANGLTSGEAAPAFQSPTECGSSGDAAFRWRKYGQKAVNGNSFPRSYYRCSTARCNARKFVERSSDNSLVTTYEGKHNHVQLQ

>Traes_5AL_ED3ADED51.3

MAGTSDRGSLMEDWMAMPPTPSPRTLMSSFLNEDFGSGQFSNFFGEHVSNKPHDQSEKRGELVGFREQLPTQSATDTATPQKDFSLQPNSFNANQKSNPQGSLAERRASRAGFSIPKIDTSRVGSSTVIRSPIAIPPGLSPTTLLESPVFLYNAMAQPSPTTGKLFVAPEANSTMPPDSTFSNDVFSFQPHSGPASYSNMEKGYNVCHQNQSLSNIHQQGSSLQSSFTAAKDSADETIVKPKTSDSVFSDNHSSEEQEDDEGDQNEEYSSATNSNPAEDGYNWRKYGQKQVKSSEHPRSYYKCTHPDCPVKKKVERSQDGQITEIVYKSSHNHPLPPPNRRSGIPLSQINDQQVHVLEKPGSHAGLNTASLWENGKSECIQDMQGVEGRPAAGPPVSAYGDTSIMESQDAADVSSTLSNEIDRATQGTISLDCDVGEDETESKRRKLDALAAVTIPTATTTSSIDMVAAASRAVREPRVVVQTTSEVDILDDGYRWRKYGQKVVKGNPNPRSYYKCTHQGCSVRKHVERASHDLKSVITTYEGKHNHEVPAARNSGNAGSGSGSATASAPQANLSHRRQEQAQGSYPQFG

>Traes_5AS_433D3E526.1

MRLLGGPAASDPLNVRQCAPKRRCAGRGEDGSGKCTTGGKCHCSKRRKLRIKRSIKVPAISNKISDIPPDEYSWRKYGQKPIKGSPHPRGYYKCSTVRGCPARKHVERCVDEPAMLIVTYEGEHSHNRLPTQSAQT

>Traes_5AS_9C6171380.1

MGGFGMSHQQALAQVTAQASHSPLRMFDHTEQPSFSAAATSSGALQNMSSAANVAEMSEMATTISNNEHAAFQSAEASHRYQVPAPVDKPADDGYNWRKYGQKVVKGSDCPRSYYKCTHPSCPVKKKVEHAEDGQISEIIYKGKHNHQRPPNKRAKDGSSSAAEQNEQSNDTASGLSGVRRDQEAVYGMSEQLSGLSDGDDKDDGESRPNEVDDRENDCKRRNIQISSQKALTESKIIVQTTSEVDLLDDGYRWRKYGQKVVKGNPHPRSYYKCTFAGCNVRKHIERASSDPKAVITTYEGKHNHEPPVGRGSNQNGGNSNRSQQKGSNSMSSNQASHTRTDLGNVNQGQIGVLQFKREE

>Traes_5BL_0A3D332A8.1

MESVDENGGSRLVVTELGYIKELVRQLDVNLGGCPDHCKRLAAQIFAVTERSIGMIRSGHFDSRKRSAAGLDSPPFSATPSPLSDVSGMPFHTNNKKRKTMEKRKHQVRVSSEGGGAETPVDDGHSWRKYGQKDILGAKHPRGYYRCTHRKSQGCAATKQVQRADEDPALFDVIYHGEHTCVHKTVAAAAAMVQPAEENPDARRHLQNLSTSLTVNTEGLTAGHQGCSTTTSFCFSSQAAGVLTMPQEHYPFSMPSTPENCFGQGASLSTSLEPSPVTSDSNRFSMSPFQAEWRARSEYDEVVSALVAAGTMPALTMEMEEETAFSLDEFEFDVSCFLA

>Traes_5BL_175E7FC38.1

MQRSRGCAPGGEHGWAAADGGMQLQRRERELVAQLHELLFPSTSPSRSGASSCSGPAADLYWEHGSPQVKATASCGGGKRRGGRKRVREDERHEEGQGQRGGAAPATKATTRCRRKKQGATTTTLVTTVPDFDGYQWRKYGQKQIEAAKHPRNYYRCTNSTNQGCPAKRTVQRNDDDGSDDGRPKYTVVYISEHSCKATESAAVPVILETTVRTDTAAAPNVAVVPGSRSGAISSETQSPASSSDITWSSGGSDGGANVPPRERDDYSRMFAIEDDCWGWNASPPAPAAAAALLQEMDFDGPIRSPVHVAAADGSWINDLFVNEPPFVLNSCHLFGL

>Traes_5BL_17A712C94.1

MAGTSDRGSLMEDWMAMPPTPSPRTLMSSFLNEDFSSGQFSNFFGEHVSNKPHDQSEKRGELVDLREQVPAQSATDTATPQKDFSLQPNSFNANQKSNPQGSLAERRASRAGFSIPKIDTSRVGSSTVIRSPIAIPPGLSPTTLLESPVFLYNAMAQPSPTTGKLFVASEANSTMPPDSTFSNDVFSFQPHSGPTSYSNVEKGYTVCHQNQSLSNIHQQGSSLQSSFTAAKDSADETIVKPKTSDSVFSDNHSSEEQEDDEGDQNEEYSSATNSNPAEDGYNWRKYGQKQVKSSEHPRSYYKCTHPDCPVKKKVERSQDGQITEIVYKSSHNHPLPPPNRRSGIPSLQINDPQVHLLEKPGLHTGVNTASLWENGKSECIQDMQGVEGRPAAGPPVSAYGDTSIMESQDAADVSSTLSNEIDRATQGTISLDCDVGEDETESKRRKLDALAAVTIPTATTTSSIDMVAAASRAVREPRVVVQTTSEVDILDDGYRWRKYGQKVVKGNPNPRSYYKCTHQGCSVRKHVERASHDLKSVITTYEGKHNHEVPAARNSGNAGSAPASAPQANLSHRRQEQAQGSYSQFGGASPFGSFGLPPRGHLGAAGNFHFGMAPPGMSMPPMPAARHPSMMQGYPGLMMQEGQMMQEGQMKAEPDQQSGFAASSAYQQMMGRPPFGPQM

>Traes_5BL_8688F70C9.1

MAVDFVGRGHAPRGLALAGGQQQLAFHEAAAAGLSSLELLVSALSPRADCAPPPLGEIADQALSGFRRVIDILGRTGHARFRRGPVGGGAASLTPPPVSSPPRMPARPPAPAASQQLAPQKSLTLDFTKPSKTPAAAAAASVTSTSFFSSVTAGGEGSVSKGPSQLVSSGKPPLAAGTKRKQQQQQTPCASAAHSDAAAAAGGRCHCSKKRKHRVKYTTRVPAVSSRTADIPGDDYSWRKYGQKPIKGSPYPRCYYRCSTAKGCPARKHVERATDDPAMLIVTYEGDHRHDTLPPAAAN

>Traes_5BL_8BEF7F9CD.1

MDTARRSPVCLDLMVGLPMVCEPSSARCTGMRAQADIASSACGRATSMTSSEANKIMEAKFTEVTEENRRLTEMIGYLYANSQNFARQSPEGEGEQPASTAASPTSPVGKKRSRESMDTSDSGDVNSDKKMGTAEAEHIDVESPLSNGTCRRIKVKRVCTRIDPSDTSLVVKDGYQWRKYGQKVTRDNPSPRAYFRCAFAPSCPVKKKVQRSAEDSSVVEATYEGEHNHPRPTRAGELPSCATQGGGSVPCSISINSSGPTITLDLTKNGGGVQVVEAGEAQPDLKKVCREVASPEFRAALVEQMARELTGDRKFTDALAAAILRKLPDY

>Traes_5BL_90757F0CC.1

MCCFWTMGTAPVCLDLMVGRPMDHEPSPVRCTGVRTEADVASSACDRAPPMTNDEAKILEAKLAQVSEENRKLTEMIAYLYGNQVSRQSPDGEGQQRARTAASPTPPAGKKRSQESMDASHSCDVEISNRNVGTGEAEHVDVDSPLSNGTCRRIKVKKVCTRIDPSDTSLVVKDGYQWRKYGQKVTRDNPSPRAYFRCAFAPSCPVKKKVQRSAEDSSVVEATYEGEHNHPHPTRAGELPSYAARSGGSVPCSISTNSSGPTITLDLTKNGGGVQVLDAGEAQPDMKKVCRAVASPEFQRALVEQMARELTGDQKFTDALAAAILRKLPDY

>Traes_5BL_A522C62D1.1

MQSQEKITPVKPVASRPFSSFTSFSKLLKDFTVTGSATISSPGETVIVRRPKVTRFAPPPSDLSAGIAATMLQDAGLDTTHEKMVIDPEQVVSCDQMTAFHDINKPIHGVKNRLSYDGYNWRKYGQKQVKGSEFPRSYYKCTHPTCPVKRKVETTVDGQIAEIVYNGEHNHPQPHPPKKPASLASTEVVVPDAHGSNDAGAESQLGGCNLALVSDPVAAAFKSSCDYVDEIGSTSPVYHCNTSQKEKQTSITNGLTSGEAAPAFQSPTECRSSGDAAFRWRKYGQKAVNGNSFPRSYYRCSTARCNARKFVERSSDNSLVTTYEGKHNHVQLR

>Traes_5BL_AEF9FE805.2

MDGMVESNREAVQSCHKVLDLLSNPHGQLVPHKDLLEATGAAVAKFGSLASKIGNGNGGRQGHARFRQRIKKPMPLFDSNLFRDSPASAAAADAAAAAPPKTSSPGPSTSLQLFPRYQQMEASSSKDPVRIPAAQFPQRMVVENPSVGSNGPARGPPLHLVQPVSVAPPAGTPAPALPAAHLHFIQQQQSYQRFQLMHQMKLQSEMMKRGGHGDHQGGSTGAGKGVNLKFDGSNCTGSSSRSFLTSLSMEGSMASLDGSRSSRPFQLVSGSQTSSTPEMGLMQQRRRCIGKEDGSGRCATGSRCHCAKKRKLRIRRSIKVPAISNKVADIPADEFSWRKYGQKPIKGSPHPRGYYKCSSVRGCPARKHVERCVDDPAMLIVTYEGDHNHNRAAAAQPQPA

>Traes_5BL_B9DD3E76F.1

MDMEEQANAAATAAREGDLADVVARANSMPYSAGARRQAPPPPPPSAAARVMIPYEEERQRRPANVACGGGGGQVTFEAPPSTVVVDPYLLAAAGGYGLPQQQQHQHQQLLAFQISEHACCAAADSDDPMRISPPPPPPPPAPHHQMITSYCGMACTHIPYCRKNDVRKVVCIPAPPVMSNRAGGGGEVIPSDLWAWRKYGQKPIKGSPYPRGYYRCSSSKGCLARKQVERSRSDPNMLVITYTAEHNHPWPMQRNVLAGYARAHTHAAAKKQQKISSSSSADNAASSSSSNSFHVEQINPICGDQLPVSCKMPDSTATAGDGGGLLFEGIQPDEVFAELEELETDNNPMMTSANVYGSRGVSSNYEWHKF

>Traes_5BL_C1D6B6B74.2

MAGAAGDRAEGVGGGGDWPPFAGDAFAEXXXXXXXXXXXXXXXXXXXXXLPPLDLPAASASGPVPMARSDEIMPAASGEPAGAASSCSSGDGAAAAAENADKPQEAADAASMKPAAAAGKKGQKRARQQRFAFVTKSEVDHLEDGYRWRKYGQKAVKNSPFPRSYYRCTNSKCTVKKRVERSSEDPSVVITTYEGQHCHHQTSFQRGGMHFHGAATVALAEQMSFVSTQQLYNLPPLRRQQMNPASSESAVSSVPPSLQQLNGGDELRRSTSYSPTASAVQTPSSSLVPPDVSFDMGLLGDIVPPGVRNG

>Traes_5BL_D3C383CF5.1

MADRRGDGMRQQPPYSSGHQERVFDGGGGSSGGPAFGNDYDPGSSYMSLLGSGVNPQQLLPAPPAWAVEEVAPPTINLTPQFSMANYVPTSSYQQQQHQTAASFVAPLAANLHPYQSSSSSYFQADPLPQWPPRAMAPSPSSSLLPRNFTLHQTPAYPHHHEQQMHMQLLRAAALGGPHAAPAPPIEQPAKDGYNWRKYGQKQLKDAESPRSYYKCTRDACPVKKIVERSFDGCIKEITYKGRHTHPRPPEPRRAGAEDVAAPGSAHQEDELSDDEDDGEEGHDIGSGAGGPAGQRVVRKHKIILQTPSEVDLLDDGYRWRKYGQKVVKGNPRPRSYISGW

>Traes_5BL_E294922A9.2

MEGVEEANRAAVVSCKRLVARLSLSAGDPFRLAAVAAETEEAVSRFSKVVNILGNRVGHARARVGRRSSPAGDPIARCLLEYHPPPPVPYCPPASAPQLHGSSSSTPAPPTPLKQMAVPVAAAAAPCATDRDMFFQTPLLDLSGCSVTPASMPPCRSTAREFPQQQPAPPQKRMLEQQQRPASSDNKRFHFEPKPASEKPFHIEIPAARSGKEPEVITFSFDNSVCTSSAATSFFTNMSSQLISMSETSACAPASRKAAHKADDDGKCHCPKKKSVPVAYAIDLLKPREKRVVRMPAVSDKVADIPSDSYSWRKYGQKPIKGSPHPRGYYRCSSIKDCPARKHVERCRGDAGMLIVTYENDHNHAQPLDLATLTANSEV

>Traes_5BL_F853EA802.1

MEAVHEGNGGGSGLVVTELSHIKELVKQLDVHLGGSPDLCKLLAQQIFAVTERSIGMIKSGHFNGPKRSAAGAGLDSPPLSATPSPVSGVSNTPFKPNKKRKTSEKGRHQIRVSSAAGGADAPADDGHSWRKYGQKDILGAQHPRAYYRCTYQKTQGCAATKQVQRADEDPALFDVIYHGEHTCVHKTAAAAGMVQPAGQNPDAQSLLQSLSSSLTVKTEGLTAAGTQGWSTTTPFSFSSPAVSGMTPAEHHPFSTPSTPENCFVSMPTSLEPSPATSGSNHMCMTPFHVHSELQTMVSALVEATSMPAADTEEAAFSYWGFDDSALHVNNLDEDVNNFDISTFFA

>Traes_5BS_C46781248.1

MSHQQALAQVTAQASHSPLRMFDHTEQTSFSAAATSSGALQNMSSAANVAEMSEMATTISNNEHAAFQSAEASHRYQVPAPVDKPADDGYNWRKYGQKVVKGSDCPRSYYKCTHPSCPVKKKVEHAEDGQISEIIYKGKHNHQRPPNKRAKDGSSLAAEQNEQSNDTASGLSGVRRDQEAVYGMSEQLSGLSDGDDKDDGESRPNEADDRESDCKRRNIQISSQKALTESKIIVQTTSEVDLLDDGYRWRKYGQKVVKGNPHPRSYYKCTFAGCNVRKHIERASSDPKAVITTYEGKHNHEPPVGRGSNQNGGNSNRSQQKGPNSMSSNQASHTRTDLGNVNQGQIGVLQFKREE

>Traes_5BS_E0345D5DF.2

MEEVEAANSAAVESCHKLLALLSQQQDPALLRSIASETGEACAKFRKVVSLLSNGGGGRGGHARGRFSRRRKPVGFLSQKGFLESSSNTPLGMLMSGSAPTPSPSAASAAQLRPQVGAPPQPRSLDLVSSSSKSAHQFGPPKMVQPLSVQFQFGATAHRYPFQQQQQNLQAQMFKRSNSGISLKFDSPSGGAGTISSPRSFMSSLSMDGSVASLDGKPPMRLLGGPAASDTLNVRQCAPKRRCTGRGEDGSGKCTTGGKCHCSKRRKLRIKRSIKVPAISNKISDIPPDEYSWRKYGQKPIKGSPHPRGYYKCSTVRGCPARKHVERCVDEPVMLIVTYEGEHSHNRLPTQSAQT

>Traes_5DL_09F1F8F79.1

MQRSRGCAPGGEHGWAAADGGMQLQRRERELVAQLHELLFPSTSSGASSCSGLAADLYWEHGSPQVKATASCGGGKRRGGRKRVREDERHEEGQGQRGGAATATKATTRCRRKKQGATTTTLVTTVPDFDGYQWRKYGQKQIEAAKHPRNYYRCTNSTNQGCPAKRTVQRNDDDGSDHGRPRYTVVYISEHSCKATESAAVPVILETTVRTDTAAAPDVAVVPGSSSGAISSETQSPASSSDITWSSGGSDGGANVPPRERDDYSRLFAIEDDCWGWNASPPAPAAAAALLQEMDFDGPIRSPVHVAAADGSWISDLFVNEPPFVLNSCHLFGL

>Traes_5DL_1733FB4DA.1

MLSQVRPHQTSHTWKLHSLIADIRTHLQDKRYLIVIDDVWATQTWDIVSRALPDGNLCSGVLITTEIDDVALKCGGYDSKYVLPMKPLGHDDSSKLFFRTAFGPQYECPPELSDVANNIIRKCAGFPLAVVTVAGLLVNQMGKPEQWDFVNKSLGYGLRKNPAPEGMKQVLNLSYNNLRLHLKACLMYLSIYEEDYIIQKNDLVKQWIAEGFIHATEEKDMVEISRICFDELISSRMIEPVHINDTGDVLSCTVHHMVLDFITHKSLEENFVTAIDHCQTTARLADKVRRLSLHFGNAEATPPTNMRLSQVRTLAYFGVIKCLPSIVEFGLLQILILHLWGDDDSISFDLTGISELFRLRYLHVTCNATLEVPQTQIRGLRYLETLKIDARVSAVPSDIVHLPGLLHLSLPVEINLPNGIGRMTSLCTLECFDISVNSVENVHSLGELTNLHDLRLTCSTVHSCYLTSKMDSMCSILTKLSNLRSLTLEPSSILDVGPSSMSISCDGLSSVSSPPACLQTFEWLPRICTFSSLPKWIGRLSKLCILKIGVRKLANNDFDILRGLPALTVLSLHIRTKPAKRILFNKIGFSVLKYFKFRCRAPWLEFEVDAMPNLLKLKLRFDAHGVDQHGTIPVGIVHLTGLKEMSAKIGGAGANDPDRRAAESALIDAIKMHPACPTLSIHCLDAMFSGEDDDIKKEDSIEHMTLQKQYDVKKEDSIEHMTLQKQYGIKKEDSIEHTTLQEQYDIKNEDLIEHMTLQIQNDIKKEDSYKQHGFLQKDYRKTLPKWSTQVRVSSLQDIEGHDDGFSWRKYGQKDILGSRNPRGYYRCTHHNTRGCQALKQLQATDGDPLLFNAIYVGNHTCTQGANSQPQPGYEQSSISVGDKAEGSIQRLEKMPPRRSKRSIQVRVRSMQDDYPADDGYSWSKYGQKDILGSKHPRGYYRCVHRPEKGCEATKQVQRSDSDTQLFDVVYHGEHTCAENVHSRGESARSLPHHVSVSAGVIPPATSESQVTYEAVSSGSTAGIHFMSPATSAGSQVTYESGSRSTTTGRFISPGMSESQVAYAEFWTWPNNVDFMLNSPINERLDLNADFVDETGPSDFD

>Traes_5DL_21F7C6BF7.2

MAGTGDRGSLMEDWMAMPPTPSPRTLMSSFLNEDFGSGQFSNFFGEHVSNKPHDQSEKRGELVDLREQVPAQSDTATATPQKDFSLQPNLFHANQKSNPQGSLAERRASRAGFSIPKIDTSRVGSSTVIRSPIAIPPGLSPTTLLESPVFLYNAMAQPSPTTGKLFVASEANSTMPPDSTFSNDVFSFQPHSGPTSYSNVEKGYNVSHQNQLLSNIHQQGSSLQSSFTAVKDSADETIVKPKTSDSVFSVNHSSEEQEDDEGDQNEEYSSATNSNPAEDGYNWRKYGQKQVKSSEHPRSYYKCTHPDCPVKKKVERSQDGQITEIVYKSSHNHPLPPPNRRSGIPLSQINDQQVHVLEKPGSHTGLNTASLWENGKSECIQDMQGVEGRPAAGPPVSAYGDTSIMESQDAADVSSTLSNEIDRATQGTISLDCDVGEDETESKRRKLDALASVTIPTATTTSSIDMVAAASRAVREPRVVVQTTSEVDILDDGYRWRKYGQKVVKGNPNPRSYYKCTHQGCSVRKHVERASHDLKSVITTYEGKHNHEVPAARNSGNAGSGSVSAPASAPQ

>Traes_5DL_2553A6C33.1

MPAVSDKVADIPSDSYSWRKYGQKPIKGSPHPRGYYRCSSIKDCPARKHVERCRGDAGMLIVTYENDHNHAQPLDLATLTANSEXXXXXXXXXXXXXXXXXXWNMPFSLTKSECK

>Traes_5DL_32D78D06A.1

ISNRNVGTGEAEHVDVDSPLSNGTCRRIKVKKVCTRIDPSDTSLVVKDGYQWRKYGQKVTRDNPSPRAYFRCAFAPSCRVKKKVQRSAEDSSVVEATYEGEHNHP

>Traes_5DL_46E3AC8D6.1

MADRRGDAMRQQPPYSSGHQERVFDGGGGPAFGNDYDTAGSSYMALLGSGVNPQQSLPPQQAWGVDEVTPPTINLTPQFSMANYAPTSSYQQHQTTASFVSPLAANLHPYPSSSSSSYFQADLPPQWPPRAMAPSPSSSLLPRNFTVHQTPAYPHHHEQQMHMQLLRAAALGGPHAAPAPPIEQPAKDGYNWRKYGQKQLKDAESPRSYYKCTRDACPVKKIVERSFDGCIKEITYKGRHTHPRPPEPRRAGAEDAAAPSSAVGAHQEDELSDDEDDGEEGHDIVSGAGGPAGQRVVRKHKIILQTPSEVDLLDDGYRWRKYGQKVVKGNPRPRSYYQKTSFIWGR

>Traes_5DL_4BA2CC560.2

MQSQEKITPVKPVASRPFSSFTSFSKLLKDFTATGSAKITSPGETVIVRRPKATRFAPPPSDLSAGVAASMLQDAGLDTTREKMVIDPEQVVSCDQMTAFHDINKPIHSVKNRLSYDGYNWRKYGQKQVKGSEFPRSYYKCTHPTCPVKRKVETTVDGQIAEIVYNGEHNHPQPHPPKKPVSSASTEVVVPDAHGNNDAGAESQLGECNLALVSDPVAAAFKSSCDYVDEFGNTGPVYHCNTSPKEKQSSIANGLPSSGEAAPAFQPPTECRSSGDAAFRWRKYGQKAVNGNSFPRSYYRCSTARCNARKFVERSSDNSLVTTYEGKHNHVQLR

>Traes_5DL_5C93510D5.1

MEAVHEGNGGGSGLVVTELSHIKELVKQLDVHLGGSPDLCKLLAQQIFAVTERSIGMIRSGHFNGPKRPAAGAGLDSPPLSPTPSPLSGVSNTPFKPNKKRKTSEKGGRQIRVSSAAGGADAPADDGRSWRKYGQKDILGAQHPRAYYRCTYQKTQGCAATKQVQRADEDPALFDVIYHGEHTCVHKTAAAAVQPAGQNPGAESLLQSLSSSLTVKTEGLTAAGAQGWSATTPFSFSSPAVSGMTPPEHHPFSTPSTPENCFVSMPTSLEPSPATSGSNHMCMTPFHAQSELQTMVSALVEATSMPAAGTEEAAFYTFQSDWSFDDSALDVNNFDVSALDVNNFDVSVFLA

>Traes_5DL_7E2053226.2

MCARRSMKPTAAVKKGQKRARQQRFAFVTKSEVDHLEDGYRWRKYGQKAVKNSPFPRSYYRCTNSKCTVKKRVERSSEDPSVVITTYEGQHCHHQTSFQRGGMHFHGGATVALAEQMSFVSTQQLYNLPPLRRQQMNPASSESVVSSVPPSLQQLNGGDELRRSTSYSPMPSAAQTSSSSLVPPDVSFDMGLLGDIVPPGNYLPTGYI

>Traes_5DL_A54ED44C9.2

MDGMVESNREAVQSCHKVLDLLSNPHGQLVPHKDLVEATGAAVAKFGSLASKISSGNGRQGHARFRQRIKKPMPLFDSNLFRDSPASASAADAAAAAPKTSSPGPSTGLQLFPRYQQMEASSSKDPVRIPAAQFPQRMVVENPSVGSNGPPLQLVQPVSVAPPAGTPAPALPAAHLHFIQQQQSYQRFQLMHQMKLQSEMMKRGGHGDHQGGSTGAGKGVNLKFDGSNCTGSSSRSFLTSLSMEGSMASLDGSRSSRPFQLVSGSQTSSTPELGLMQQRRRCAGKEDGSGRCATGSRCHCAKKRKLRIRRSIKVPAISNKVADIPADEFSWRKYGQKPIKGSPHPRGYYKCSSVRGCPARKHVERCVDDPAMLIVTYEGDHNHNRAAAAQPQPA

>Traes_5DL_C93641E43.1

MESVDENGGSRLVVTELGYIKELVRQLDVNLGGCPDHCKRLAAQIFALTERSIGMIRSGHYDCRKRSAAGLDSPPFSATPSPLSDVSGMPFHNNKKRKTMEKRKHQVRVSSEGGGAETPVDDGHSWRKYGQKDILGAKHPRGYYRCTHRKSQGCAATKQVQRADEDPALFDVIYHGEHTCVHKTVATAAAAMAQPAEENPDARRHLQNLSTSLTVNTEGLTATAGHQGCGTTTSFCFSSQAAGVLTTPQEHYPFSMPSTPENCFGQRASLSTSLEPSPVTSDSNRFSMNPFQAEWRAQSEYDEVVSALVAAGTMPALAMEMEEETAFSLDEFEFDVSSFLA

>Traes_5DL_E4A6D1889.2

MDMKEQANAAATAAREGDLADVVARANAMAYSTGARRQAPPPPPSAAARVMIPYEEERQRRPANVACGGGEVTFEAPPSTVVVDPYLLAAAGGYGLPQQHQHQHQQLLAFQISEHACCAAADSDDPMRISPPPPQPAPHHQMITSYCGMACTHIPYCRKNDVRKVVCIPAPPVMSNRAGGGGEVIPSDLWAWRKYGQKPIKGSPYPRGYYRCSSSKGCLARKQVERSRSDPNMLVITYTAEHNHPWPMQRNVLAGYARAHTHAAAKKQQKISSSSSADNAASSSSINSFHVEQINPICVDQLPVSCKMPDSTATAGDGGGLLFEGIQPDEVFAELEELETHNNPVMTSANVYGSRGVSSNYEWHKF

>Traes_5DS_5DEA5C9E3.1

MSARPPPPPRPRLSLPPRSTGESLFSGTGDASPGPLTLASALFPSDADADAGGGGGGSGASSGAGPTSFTQLLIGSLSQPPPHQQQQQQQERGRGGVARAGPALSVAPPAGAAVFTVPPGLSPSGFFDSPGLIFSPAMGGFGMSHQQALAQVTAQASHSPHRMFDHTEQPSFSAAATSSGALQNMSSAANVAEMSEMATTISNNEHAAFQSAEASHRYQVPAPVDKPADDGYNWRKYGQKVVKGSDCPRSYYKCTHPSCPVKKKVEHAEDGQISEIIYKGKHNHQRPPNKRAKDGSSSAAEQNEQSNDTASGLSGVRRDQEAVYGMSEQLSGLSDGDDKDDGESRPNEVDDRENDCKRRNIQISSQKALTESKIIVQTTSEVDLLDDGYRWRKYGQKVVKGNPHPRSYYKCTFAGCNVRKHIERASSDPKAVITTYEGKHNHEPPVGRGSNQNGGNSNRSQQKGPNSMSSNQASHTGTDLGNIKQGQIGVLQFKREE

>Traes_5DS_D83DEA9B0.1

MEEVEAANSAAVESCHKLLALLSQQQDPALLRSIASETGEACAKFRKVVSLLSNGGGGRGGHARGRFSRRRKPVGFLSQKGFLESSSNTPLGMLMSGSAATPSPSAGSAGQLRPQVGAPPQPRRSLDLISSSSKSAHQFGPPKMVQPLSVQFQFGATAHRYPFQQQQQNLQAQMFKRSNSGISLKFDSPSGGTGTISSPRSFMSSLSMDGSVASLDGKPPMRLLGGPAVSDPLNVRQCAPKRRCTGRGEDGSGKCTTGGKCHCSKRRKLRIKRSIKVPAISNKISDIPPDEYSWRKYGQKPIKGSPHPRGYYKCSTVRGCPARKHVERCVDEPAMLIVTYEGEHSHNRLPTQSAQT

>Traes_6AL_0C0899C15.1

MSSSKRKRADIDLERRDDDVDDGSGDHHPGEAAPRVQKEGQIKEGEAPAKEVVEVVVDRGGDGSKEEIKYGTQQGGVIEEDKQSAAAAADNDAGDGIDDEEGDGGGTGAEDKHMVEAAPGDGYGDGDHEHTAMAQDELSTMQEEMEKMKEENKMLRRVVDRTVRDYYELQTKLAAYQKQPADQEPKETEVFLSLGGTAPAAAVAEAKSKEEQAALRPSVGSDDTDDGREGLGLSLSLRTSSYEDEARRDVVDGGAPDIVTDVGKVRGYALLESSRMSPPASGDLAAGGGIGSQQGVNAANRKTRVSVRVRCQGPTMNDGCQWRKYGQKVAKGNPCPRAYYRCTVAPACPVRKQVQRCQEDMSILITTYEGTHNHPLPVGATAMASTATAGAGAATFMLLSSTTSDAAVSGGPPPASSSYLSPYLQPNSSSQYHSSASPLMSGNMGGGGAQHLSMFGHSSGLSAQPATHLKYPWPPNPSHGGAAGLGGGKRPFWGTGGVDDVRPTALPDNAGTMASDPNQFSAAIATAISNVIGKDGQATRSKEGESSNKWGGG

>Traes_6AL_A5FB7CFA5.1

MCDYFLKRAEGHQQAGDLTDIVRAGGAMPAGSADPPSTATEWLQLPADPILFPLPQTSSSDGAGPSSADALGDPFSGLPDAFSTDYPSSSGSAAADFFDAVQDAMGVGMAKQVGFVDTTGCGGGGTTAGAGGGFLDMRNHHMFPGEMPMRGLSPYALMGGGAAKLGVPMAGHGLAAGPCAFDAVAGLQMSSSPRGGGIKRRKNQARKVVCIPAPAAAVAGKTTGEVVPSDLWAWRKYGQKPIKGSPYPRGYYRCSSSKGCPARKQVERSRTDPNMLVITYTSEHNHPWPTQRNVLAGSTRSHYAKNSSNTDAASSKNSKNSSRNQHKPVVKAESKDQSAATPAATSTTTTATTSTGNNTPPMAVKEEAEMERRIGGDTTATVGYYSDHLLQQMFSQSYRPMMPEEAGGYHQQDDFFADLTELDSDPVSLIFSTEYMEARPGKEKAAAKDDVDSLFMMDWAPASAAVTTSAGSALEQGDMGL

>Traes_6AL_BA4636569.1

MHICMDQGSQLGMAYCLPNLSVPDHYYTTPVPLSPLQLPFHPKPLQMPFDQEEALMLSSDHCGLYPLPALPLGSGHSAGAPAIVCEKPTVGFMPNIGAEEVGTSVTARVGYEGATACNGYSSNTWWRGSTMLAGEKGKMKVRRKMREPRFCFQTRSDVDVLDDGYKWRKYGQKVVKNSLHPRSYFRCTHSNCRVKKRVERLSTDCRMVITTYEGRHTHPPCDDNSSSSGDNTTTCF

>Traes_6AS_68775100B.1

MGNIELPPDDGYTWRKYGQKDILGSRFPRSYYRCTHKNYYGCDAKKKVQRLDDDPFMYEVTYCGSHSCLTSTTPLLNFPTATATATATNSPTAATGSGLAPADHFMAPTEQAAVSTSMHLGVGWMPASFQGVVAGSGAGGGSSAGMQTSVSTAARDTDYPALDLADVMFNSGGSVGMDGIFSSHHRRDS

>Traes_6AS_DA75BB1FD.1

MDPWVSSQPSLSLDLHVGLPPMGHHQAAPMVALAKPKVLVEENFMQLKKDPEVAVLESELQRVSEENRRLGEMLREVASKYEALQGQFTDMVTAGAHAGGNNSHYNNQPSSASEGGSVSPSRKRKSEESLGTPPRPSQHQQQHYAGGLAYAAAPDQAECTSGEPCKRIREECKPVVSKRYVHADPSDLSLVVKDGYQWRKYGQKVTKDNPCPRAYFRCSFAPGCPVKKKVQRSAEDKTILVATYEGEHNHTQPPPSQPQQQNDGSGAGKNAGKPPQAPTATPHHPQQQHKQEAAAAAVSG

>Traes_6BL_B92FA1D38.1

MNDGCQWRKYGQKVAKGNPCPRAYYRCTVAPACPVRKQVQRCQEDMSILITTYEGTHN

>Traes_6BL_DD840863A.1

MHICMDQGSQLEMAYCLPNLSVPDHYYTTPVPLSPLQLPFHPKPLQMPFDQEEALMLSSDHCGLYPLPALPFGGHSAAAPATVCDKPTVGFMPSIGAEEVGTSVTARVGYEGATACNGYSSNTWWRGSTMLAGEKGKMKVRRKMREPRFCFQTRSDVDVLDDGYKWRKYGQKVVKNSLHPRSYFRCTHSNCRVKKRVERLSTDCRMVITTYEGRHTHPPCDDNSSSSGDNTTTCF

>Traes_6BL_EEAA2A7E3.1

MSSSPRGGGIKRRKNQARKVVCIPAPAAAVAGKTTGEVVPSDLWAWRKYGQKPIKGSPYPRGYYRCSSSKGCPARKQVERSRTDPNMLVITYTSEHNHPWPTQRNVLAGSTRSHYAKNSSSNTAAAASKNSKNCSRNQHKPVVKAE

>Traes_6DL_AB95B0CE0.1

MSSSKRKRADIDLERRDDDVDDGSGDHQPGEAAPRVQKEGQIKEGEAQAKEVVEVVVDRGGDGSKEEIKCGTQQGGVMEEDKQSPAASAGNDGADGIDDEESGGGGTRAEEEHMVEAAPGDGDGDHDHTAMAQDELSTMQEEMEKMKEENKMLRRVVDRTVRDYYELQTKLAAYQKQPADQEPKETEVFLSLGGTAPAAAVAEAKSKEEQAALRPSVGSDDTDDGREGLGLSLSLRTSSYEDEARRDVVDGGAPDIVSDVGKARGYALLESSRMSPPASGDLAAAGGIGSQQGVNAANRKTRVSVRVRCQGPTMNDGCQWRKYGQKVAKGNPCPRAYYRCTVAPACPVRKQVQRCQEDMSILITTYEGTHNHPLPVGATAMASTATAGAGAATFMLLSSTSSDAAVSGGPPPASSSYLSPYLQLNSSSQYHSSASPLMSGNMGGGGAQHLSMFGHSSALASQPATLLKYPWPPNPSHGGAAGLGGGKRPFWGTGGVDDVRPTPLPDNAGTMASDPNQFSAAIATAISNVIGKDGQATRSKEGESSNKWGVVVESLPPHE

>Traes_6DL_D29E210A1.1

MHICMDQGSQLGMAYCLPNLSVPDHYYTTPVPLSPLQLPFHPKPLQMPFDQEEALMLSSDHCGLYPLPALPFGGGHSAAAPATVCEKPTVGFMPNIEAEEVGTSVTARVGYEGVTACNGYSSNTWWRGSTMLAGEKGKMKVRRKMREPRFCFQTRSDVDVLDDGYKWRKYGQKVVKNSLHPRSYFRCTHSNCRVKKRVERLSTDCRMVITTYEGRHTHPPCDDNSSSSGDNTTTCF

>Traes_6DL_D4F2CDDDC.1

MCDYFLKRADGDQQAGDLTDIVRAGGAMPAGSTDPPSTATEWLQLPADPILFPLPQTSSSDGAGPSSADALGDPFSGLPDAFSTDYPSSSGSAAADFFDAVQDAMGVGMAKQVGFVDTTGCGGGGTTVGAGGGFLDMRNHHMFPGEMPMRVLSPYALMGGGAAKLGVPMAGHGQAAGPCAFDAVAGLQMSSSPRGGGIKRRKNQARKVVCIPAPAAAVAGKTTGEVVPSDLWAWRKYGQKPIKGSPYPRGYYRCSSSKGCPARKQVERSRTDPNMLVITYTSEHNHPWPTQRNVLAGSTRSHYAKNSSNTDAASSKNSKNSSRNQHKPVVKAESKDQSAATPAATSTTTTATTSTGNNTPPMAVKEEAEMERRIGGDTTATVGYYSDHLLQQMFSQSYRPMMPEEAGGYHHQDDFFADLTELDSDPVSLIFSTEYMEARPGKEKAAAKDDVDSLFMMDWAPASAAVTTSAGSALEQGDMGL

>Traes_6DS_8F684013D.2

EGGSVSPSRKRKSEESLGTPPPSHQQHYAAGLAYAAAPDQAECTSGEPCKRIREECKPVVSKRYVHADPADLSLVVKDGYQWRKYGQKVTKDNPCPRAYFRCSFAPGCPVKKKVQRSAEDKTILVATYEGEHNHSQPPP

>Traes_6DS_BF71C1557.2

PGINIASWSPWTPALHLRRRLVLPDTVRALLHCITLLPRLLDLQGSAHVSNSSCYSTCSGFHLLKISSIVTSASFYLAFTDQWCCCVNLCRCVELGGGFPVKKKVQRSAEDSSVVGATYEGEHNHLRPMGAASRACATRGRGLVLCSISINSSGPTTMLDLTKNGGGVQVVEAGEAQPDLKKVCREVASPEFRAALVLRPRSQTSSAPPASASGASAQRSSGICQDGIFRSLCVVGASSFTGKLLILGSDCVLM

>Traes_7AL_48C81DE03.1

LSSFPWPTAXXXXXXXXXXXXXXXXXXXXXXXXXXXXXETKPEKKAVTETQVKEVSKSGPKEIEKEVKVKVEKENENVEIEATLRPTGAGTEAPPILAVPMLAVPCFIAPPGFAGQFAMSHQAALASVTAQAHIQLQSPASSAYSEGLPSPFPHPITPKAIRPLQQAPSVTQGSIGRPIAERPSSSESKLQHHAAVNIVGDGFNWRKYGQKQVKSSDNSRSYYRCTNSSCLAKKKVEHCPDGRVIEIIYRGAHSHEPPQKTRFVKERSLHIYVPPIGDGTLQLVNTEIVESRTPTCKLNQSAAIENSEQQLFCSSDCEGDVGNKSEDEHRSAESQPKRRIVEATTSNLTPVLRTVRERKIIVQAGKMSDGYRWRKYGQKIVKGNPNPSSNCEASN

>Traes_7AL_48C81DE031.1

LSSFPWPTAXXXXXXXXXXXXXXXXXXXXXXXXXXXXXETKPEKKAVTETQVKEVSKSGPKEIEKEVKVKVEKENENVEIEATLRPTGAGTEAPPILAVPMLAVPCFIAPPGFAGQFAMSHQAALASVTAQAHIQLQSPASSAYSEGLPSPFPHPITPKAIRPLQQAPSVTQGSIGRPIAERPSSSESKLQHHAAVNIVGDGFNWRKYGQKQVKSSDNSRSYYRCTNSSCLAKKKVEHCPDGRVIEIIYRGAHSHEPPQKTRFVKERSLHIYVPPIGDGTLQLVNTEIVESRTPTCKLNQSAAIENSEQQLFCSSDCEGDVGNKSEDEHRSAESQPKRRIVEATTSNLTPVLRTVRERKIIVQAGKMSDGYRWRKYGQKIVKGNPNPSSNCEASN

>Traes_7AL_AC56CC184.1

VSVTVGPDQAECTSVQEPCNSKRVHADEVKASRVSKLYVHADPSDLSLVVKDGYQWRKYGQKVTKDNPCPRAYFRCSFAPSCQVKKKVQRS

>Traes_7AS_C9DF68E53.2

MIKGDQRQLGGHEDRLKDQTFMLVLQMGDDHRASDGKFFRFLQNQSSAKKEAQEDKIASTRAEMGEVRKENERLKTMLSRMVEDHRSLQKQFDVLHQQGRGKNLAMGSPEHTSSADGVKDPRFVSLRLGTSASTSRQDMGEEIRTGTNNADGKCISLGLSSGKAIGAAGQSEMKVQPDVLTLSPGGSSEDDAATETTTTSSKVPKNPRSTGGGAEAEEEVAQQPLAKKARVSVRARCDTPTMNDGCQWRKYGQKISKGNPCPRAYYRCTIATGCPVRKQVQRCAEDMSILITTYEGAHNHPLSPSAAAMASTTSAAASMLMSGSSTSLGFPSVASSLHGLRFGLPAATTFDPSSHLSGRPFFLPAAAGASISATPSYPTITLDLTSQTASQQAFSLSNTNRFSSSFPDSHGHSSSAGRYPSTNFSFSGSGASSLPGATAWPAGVGSYLSYG

>Traes_7BL_53AA25AA1.1

MKSLDDGQAWRKYGQKEIHNSKHSRAYFRCTHKYDQQCAAQRQVQRCDDDEGMFR

>Traes_7BL_A46F1A830.2

MLAVPCFIAPPGFAGQFAMSHQAALASVTAQAHIQLQSPASAAYSEGLPSPFPHPITPKAIRPLQQAPSVTQGSIGRPIAERPSSSESKLQHHAAVNIVGDGFNWRKYGQKQVKSSDNSRSYYRCTNSSCLAKKKVEHCPDGRVTEIIYRGTHSHEPPQKTRFVKERSPHIYVPPIGDGTLQLVNTEIVESRTPTCKLNQSAAIENSEQQLFCSSDCEGDVGNKSEDEHRSAESQPKRRIVEATTSNLTPVLRTVREQKIIVQAGKMSDGYRWRKYGQKIVKGNPNPRCVLLLLTHGYFSESIKH

>Traes_7DL_5968FA56C.1

PLAAAGSGVFAVSVNVSPDQAECTSVHEPCDSKRVRADDVKASRVSKLYVHADPSDLSLVVKDGYQWRKYGQKVTKDNPCPRAYFRCSFAPSCQVKKKVQRSADDKTVLVATYDGDHNHAPPPKQQGSGGRKRGGAAVLHVSPAPVLVQEQRKHEASTADQVADRKNLVEQMAATLTRDPGFKAALVSALSGRIPVA

>Traes_7DL_A9EF00572.1

MEAPLAQVTDDLIKGRELATQLQGLLRDSPEAGGLIVDQILHAFSRAIHAARAAAAASTSERSSDVRSEVTDGASGGAKRKSASAAGGGNRRACRRRTQQSSVVTKSMESLDDGQAWRKYGQKEIHNSKHSRAYFRCTHKYDQQCAAQRQVQRCDDDEGMFRVTYIGVHACRDPAAAVAPHLLHHLSGAAQGLHAXXXXRARQRRYRYPWHHHQHGDGVRPAGHKA

>Traes_7DL_B09854286.1

AVSVNVGPDQAECTSVHEPCNSKRVRADECKASRVSKLYVHADPSDLSLVVKDGYQWRKYGQKVTKDNPCPRAYFRCSFAPSCQVKKKVQRSAEDRTVLVATYEGEHNHAQPPKMQGSGGRKSAXQQQPKQQSMTEAGSAADRKNLAEQMAATLTRDPGFKAALVSALSGRILELSPSDS

>Traes_7DL_B86B36F67.1

VKASRVSKLYVHADPSDLSLVVKDGYQWRKYGQKVTKDNPCPRAYFRCSFAPSCPVKKKVQRSAEDKTVLVATYDGDHNHAPPPKQQGSGGRKSGXXXXXXXPALVQQQQKQEASTAEQVADRKNLVEQMAATLTRDPGFKAALASALSGRIPVA

>Traes_7DL_F849918EA.2

LAAAGSSGRFAVSVTVGPDQAECTSVHEPCNSKRVRADECKASRVSKLYVHADPSDLSLVVKDGYQWRKYGQKVTKDNPCPRAYFRCSFAPSCHVKKKVQRSAEDKAVLVATYDGDHNHAPPPKQQGSGGRKSGXRSRRTRISCAGACPAAAEARTFDGGAGGR

>Traes_7DS_01F74C6F3.1

RFLSERKGLPRWTEKFRIPDDANLEYTPDDGFSWRKYGQKDILGAKFPRGYYRCTYRNAQGCAATRQVQRSDADLAVFDVTYQGAHTCLQ

>Traes_7DS_24C563960.1

MLAVPCFIAPPGFAGQFAMSHQAALASVTAQAHIQLQSPAPSAYSEGLPSPFPHPITPKAIRPLQQAPSVTQGSVGRPIAERPSSSESKLQHHAAVNIVGDGFNWRKYGQKQVKSSDNSRSYYRCTNSSCLAKKKVEHCPDGRVIEIIYRGTHSHEPPQNTRFVKERSPHIYVPPIGDGTLQLVNTEIVESRTPTCKLNQSAAIENSEQQLFCSSDCEGDVGNKSEDEHRSAESQPKRRIVEATTSNLTPVLRTVREQKIIVQAGKMSDGYRWRKYGQKIVKGNPNPRCVLLLLTHGYFSESIKH
