# Supplementary material for: Characterizing the Role of TaWRKY13 in Salt Tolerance
Source: Int J Mol Sci. 2019 Nov 14;20(22):5712. doi: 10.3390/ijms20225712 (PMC6888956; doi:10.3390/ijms20225712)
Supplement: Supplementary file 1 [file ijms-20-05712-s001.zip › Supplementary Table 4.docx]

| **Supplementary Table 3** Primer sequences used in this study | |
| --- | --- |
| **Primer name** | **Sequence** |
| TaActin-F  TaActin-R | CTCCCTCACAACAACCGC  TACCAGGAACTTCCATACCAAC |
| AtActin-F  AtActin-R | CGCCATCCAAGCTGTTCTC  TCACGTCCAGCAAGGTCAAG |
| qTaWRKY4-F  qTaWRKY4-R | ACCAATTGCAGAGAGGCCAT  GGCCATCAGGACAATGCTCA |
| qTaWRKY9-F  qTaWRKY9-R | CAAAAGGCAGTCAAGGGTGG  AGTTGAAGGCTCCTTGGCAT |
| qTaWRKY12-F  qTaWRKY12-R | CGATGGCTACAAGTGGAGGA  GAGGAGGAATTGTCGTCGCA |
| qTaWRKY13-F  qTaWRKY13-R | CTTGTGGTGTCGGAGCTGT  GAGCTGGACGTGCTGGAG |
| qTaWRKY15-F  qTaWRKY15-R | CCAATCCTCCTACTCCCCCT  CTCGATGTGGAACGGCTTCT |
| qTaWRKY22-F  qTaWRKY22-R | GCAGGAATTACTACCGGTGC  GGACGGTGGTCTCGAGGAT |
| qTaWRKY29-F  qTaWRKY29-R | CATCACCCTCGTGTGCCTAC  GTAGTAGCCCCTCGGGTACG |
| qTaWRKY33-F  qTaWRKY33-R | GTCTCCAAGCGCTACGTCC  CGCCACGAGTATGGTCTTGT |
| qTaWRKY34-F  qTaWRKY34-R | TATCCTCATTCGCATACCCGC  CTCTGTATGCACAAAGTCCAGT |
| qTaWRKY44-F  qTaWRKY44-R | CATCACAGGGCCTTCAGTGT  CACCGGTAGCCATCATCCAA |
| qTaWRKY53-F  qTaWRKY53-R | TACCCACGGGGCTACTACAA  CGGACACACTAGCTACGGC |
| qTaWRKY70-F  qTaWRKY70-R | TCAACGACCACACATGCGAT  ACGTGTAGTTGTAGCTGGGC |
| TaWRKY13-F  TaWRKY13-R | ATGGAGAGCGTGGAGGGAAA  CCCTCTTACCAAGGACAGGC |
| 1302-TaWRKY13-F  1302-TaWRKY13-R | GGGACTCTTGACCATGATGGAGAGCGTGGAGGGAAA  TCAGATCTACCCATGGCCCTCTTACCAAGGACAGGC |
| hGFP-TaWRKY13-F  hGFP-TaWRKY13-R | TATCTCTAGAGGATCCATGGAGAGCGTGGAGGGAAA  TGCTCACCATGGATCCCCCTCTTACCAAGGACAGGC |
| 1305-TaWRKY13-F  1305-TaWRKY13-R | TTACTTCTGCACTAGGTACCATGGAGAGCGTGGAGGGAAA  CGGACTTAAGACTAGTCCCTCTTACCAAGGACAGGC |
| 1305-pTaWRKY13-F  1305-pTaWRKY13-R | CCATGATTACGAATTCTGGTGAGGGCACAATTCCTC  CTCAGATCTACCATGGTCGAAAAGGCAGTCCATGCT |
