# Supplementary figures and images for: Characterizing the Role of TaWRKY13 in Salt Tolerance
Source: Int J Mol Sci. 2019 Nov 14;20(22):5712. doi: 10.3390/ijms20225712 (PMC6888956; doi:10.3390/ijms20225712)

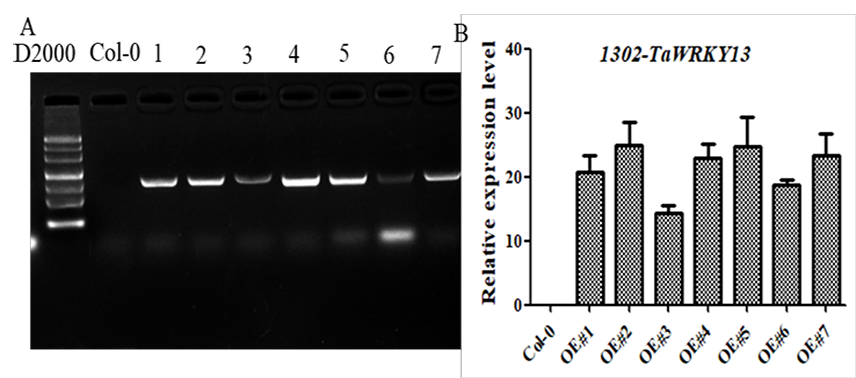

Supplement: Supplementary file 1 [file ijms-20-05712-s001.zip › Figure S1.tif]
